# Assessing radiomic feature robustness to interpolation in ^18^F-FDG PET imaging

**DOI:** 10.1038/s41598-019-46030-0

**Published:** 2019-07-04

**Authors:** Philip Whybra, Craig Parkinson, Kieran Foley, John Staffurth, Emiliano Spezi

**Affiliations:** 1 School of Engineering, Cardiff University, Cardiff, United Kingdom; 2 Velindre Cancer Centre, Cardiff, United Kingdom

**Keywords:** Cancer imaging, Image processing, Tumour biomarkers, Tumour heterogeneity

## Abstract

Radiomic studies link quantitative imaging features to patient outcomes in an effort to personalise treatment in oncology. To be clinically useful, a radiomic feature must be robust to image processing steps, which has made robustness testing a necessity for many technical aspects of feature extraction. We assessed the stability of radiomic features to interpolation processing and categorised features based on stable, systematic, or unstable responses. Here, ^18^F-fluorodeoxyglucose (^18^F-FDG) PET images for 441 oesophageal cancer patients (split: testing = 353, validation = 88) were resampled to 6 isotropic voxel sizes (1.5 mm, 1.8 mm, 2.0 mm, 2.2 mm, 2.5 mm, 2.7 mm) and 141 features were extracted from each volume of interest (VOI). Features were categorised into four groups with two statistical tests. Feature reliability was analysed using an intraclass correlation coefficient (ICC) and patient ranking consistency was assessed using a Spearman’s rank correlation coefficient (*ρ*). We categorised 93 features *robust* and 6 *limited robustness* (stable responses), 34 *potentially correctable* (systematic responses), and 8 *not robust* (unstable responses). We developed a correction technique for features with potential systematic variation that used surface fits to link voxel size and percentage change in feature value. Twenty-nine *potentially correctable* features were re-categorised to *robust* for the validation dataset, after applying corrections defined by surface fits generated on the testing dataset. Furthermore, we found the choice of interpolation algorithm alone (spline vs trilinear) resulted in large variation in values for a number of features but the response categorisations remained constant. This study attempted to quantify the diverse response of radiomics features commonly found in ^18^F-FDG PET clinical modelling to isotropic voxel size interpolation.

## Introduction

Radiomics intends to offer additional diagnostic and predictive utility in clinical practice via the extraction of imaging features from radiological scans^1^. These features aim to quantify tumour attributes such as shape, intensity, and heterogeneity, that correlate with clinical outcomes and facilitate precision-based cancer therapy^2^. The radiomics workflow can be broken down into several components, including image acquisition and reconstruction, tumour segmentation, feature extraction, and model development. For feature extraction, texture analysis is a widely adopted approach that has shown promise in both Positron Emission Tomography (PET) and Computed Tomography (CT) imaging for quantitative characterisation of tumour heterogeneity^3^. Despite encouraging studies, there are unique challenges to overcome in each stage of the radiomics workflow before we can progress from a concept to clinical use^4^. A key challenge is to ensure imaging features with prognostic or predictive value are suitably robust to necessary image processing steps along the radiomics pipeline.

One such processing step, scan interpolation, has been utilised for three main reasons in literature; the comparison of datasets spanning multiple centres with varying protocols and reconstruction parameters (that resulted in different voxel sizes)^5–8^, the resampling of registered multimodal imaging to the same voxel resolution such as in PET/CT^9^, and to acquire isotropic voxel dimensions for 3-dimensional (3D) feature extraction^10^. Several studies have focused on the robustness of radiomic features to interpolation^5–7,11^. However, these studies were primarily on CT imaging, and the resampled voxel dimensions used were often anisotropic.

In routine clinical settings, most imaging modalities produce anisotropic voxels after scan reconstruction, where the thickness between axial slices is larger than the cross-sectional resolution (i.e. ∆z > (∆x, ∆y)). To establish conservation of scale in all three directions, and remove a directional bias in 3D features, it is recommended to resample images with 3D interpolation such that ∆z = ∆x = ∆y^10^. Isotropic voxel size is thought to impact the predictive value of features^12^. Texture features quantify spatial variation in voxel intensities, and interpolation either decreases (up-sampling) or increase (down-sampling) the spatial distance between voxels. Down-sampling to a larger voxel size leads to information loss, where-as up-sampling to a smaller voxel size creates artificial information at a higher resolution. Extreme down-sampling creates a poor-quality image, extreme up-sampling creates local homogeneity and image smoothing. Clearly, image resolution is an inherent part of image texture. Yet, if potential biomarkers are heavily influenced by small variations in the resampling dimension, they are unlikely to be suitable for model development, so thorough reporting of their response to interpolation is a necessity.

The main purpose of this study was to assess the effect of interpolation on radiomic features for a range of isotropic voxel dimensions, following recent feature harmonisation guidelines^13,14^, using fludeoxyglucose (^18^F-FDG) PET imaging of a large oesophageal cancer patient cohort with the same scanning protocol. We explored two methods, linear and spline, used commonly in radiomic studies. We found common 3D texture features sensitive to interpolation, and evaluated potential correction techniques for features showing a potential systematic voxel-size dependence.

## Materials and Methods

### Patient images

This study re-uses highly curated patient data from previous studies^15,16^. The patients all had biopsy proven oesophageal cancer, either adenocarcinoma or squamous cell carcinoma (SCC), and underwent PET/CT as part of the routine diagnostic staging pathway. Patients were excluded from the original study if the primary tumour was classed as non- or poorly FDG-avid (defined as a maximum standardised uptake value (SUV_MAX_) less than 3), they had a synchronous tumour on PET/CT, an oesophageal stent was *in situ* at the time of PET/CT or a histological diagnosis was not confirmed or found to be different than adenocarcinoma or SCC. The full PET/CT protocol with image acquisition parameters were published in a previous study from our group^15^ and can be found in supplementary materials. Original PET images were acquired with a voxel size of 2.73 × 2.73 × 3.27 mm. In total, 465 patients were considered for inclusion in this study.

### Segmentation

The metabolic tumour volume (MTV) was outlined on the PET using ATLAAS (Automatic Decision Tree Learning Algorithm for Advanced Segmentation)^17,18^. Contours were rated by an expert radiologist (KF) using a binary score (acceptable = 1, unacceptable = 0). Only one expert was feasible due to limited resources and large patient numbers. A total of 441/465 tumour contours were deemed acceptable and carried forward for analysis. Segmentations were performed on the original scan dimensions.

### Voxel dimension resampling

The methodology for interpolation was applied following the detailed guidelines provided by the image biomarker standardisation initiative (IBSI)^13,14^. PET scans were resampled to 6 different voxel sizes (2.7 × 2.7 × 2.7 mm, 2.5 × 2.5 × 2.5 mm, 2.2 × 2.2 × 2.2 mm, 2.0 × 2.0 × 2.0 mm, 1.8 × 1.8 × 1.8 mm and 1.5 × 1.5 × 1.5 mm) using both (tri)-linear and spline interpolation. Trilinear interpolation is just the extension of linear interpolation in 3D that approximates the new intermediate voxel value for the new coordinate system by using the 8 closest neighbours from the previous coordinate system. A spline algorithm essentially uses a smooth third-order polynomial fit between the old voxel values to calculate the new. Following IBSI^13^ guidelines, the interpolated voxel coordinates were mapped to the original image grid by aligning the grid centres instead of grid origins. The binary mask of the contours outlining the tumours were defined on the original image dimensions and interpolated to the new grid spacing for feature extraction always using trilinear interpolation. A binary mask will become non-binary after linear interpolation to a higher resolution, as voxels at the edges of the contour become fractions. To reset the mask to binary, a threshold was used such that values ≥ 0.5 are set to 1 and the remaining set to 0.

### Feature extraction

Features were extracted from the segmented volumes using the Spaarc Pipeline for Automated Analysis and Radiomics Computing (SPAARC), an in-house software built on the MATLAB platform (MathWorks, Natick, MA, USA). SPAARC implements a wide range of solutions for processing imaging and radiotherapy data and utilises algorithms validated against a digital phantom as part of the IBSI international collaboration^13,16,19^. Full imaging feature names are found in supplementary materials. DICOM files were imported to Matlab using the Computational Environment for Radiological Research^20,21^.

Prevalent radiomic features from literature that have undergone standardisation were considered in this analysis^13^. Morphological features relate to the tumour shape. Features that measure tumour heterogeneity can be separated into first-, second-, or third-order and higher statistical descriptors. First-order features quantify the distribution of intensity values, ignoring spatial position within the volume of interest (VOI). Second order features quantify relationships between direct neighbouring voxel intensity values, and third order and higher features quantify regional relationships between voxels.

Second order and higher features are often described as texture features. We included second order features derived from a grey level cooccurrence matrix (GLCM), and third order and higher features extracted from a grey level run length matrix (GLRLM), grey level size zone matrix (GLSZM), grey level distance zone matrix (GLDZM) and neighbourhood grey tone difference matrix (NGTDM). Seventy-eight texture features were selected in total. Only 3D versions of features were considered for this work. Intensity values within an VOI need to be quantified into integer values for any second order (and higher) analysis that use grey level matrices. We selected a fixed bin width of 0.5 SUV^22^. GLCM and GLRLM were generated for each unique direction and merged before features were calculated. All mathematical definitions, feature descriptions and original references are available in the IBSI documentation^13,14^.

### Statistical analysis

Intraclass correlation coefficients (ICC) were used to assess feature reproducibility when extracted at different isotropic voxel dimensions^23,24^. ICC values lie between 0 and 1 with a threshold >0.9 often used to express high repeatability^25^. With ICC we consider each voxel dimension a rater and each patient a subject. Based on reporting guidelines by Koo and Li^24^, we selected the “2-way mixed-effects model, single rater, absolute agreement” definition of ICC. This is referred to as ICC(2,1) using the Shrout and Fleiss convention^23^. We selected “absolute agreement” over “consistency” (ICC(3,1)) for the analysis as we define a feature algorithm to be perfectly stable to interpolation if it produces the same value at each voxel size, so agreement is a larger concern. Although we utilise ICC(2,1) for our analysis, results for both definitions are reported in supplementary materials, along with the 95% confidence intervals. ICCs were calculated in R with the package *irr* (version 0.84.1)^26^.

A further test for robustness was performed using patient rankings as a proxy for feature value, similar to Leijenaar *et al*. cf.^22^. For each voxel dimension, the patient with the lowest feature value was assigned a rank of 1, the second lowest a rank of 2, and so forth. Patients with equal feature values received the same rank. Correlation between patient rankings after resampling to different voxel sizes was assessed pairwise with the 2.7 mm result, using Spearman’s rank correlation coefficient (*ρ*). The 2.7 mm voxel was chosen as the “ground truth” as it was closest to the axial plane resolution of the original reconstructed PET images. A threshold of *ρ*_*mean*_ > 0.95 was used to indicate high consistency in ranking.

**Figure 1:**
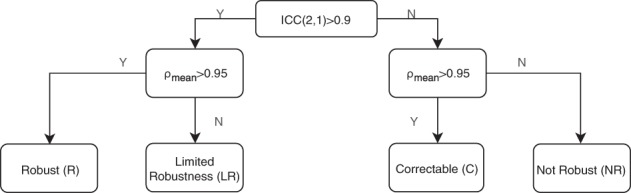
Flow chart of the criteria used to categorise feature robustness to interpolation. Feature values were grouped using a threshold value for ICC(2,1) analysis, and a threshold value for patient ranking correlations tested with spearman rank *ρ*.

The method for categorisation of feature robustness for this study, combining both statistical tests, is summarised in Fig. 1. We aimed to categorise how each feature responded as we reduced the voxel size with interpolation. Robust (R) features were considered stable to interpolation effects and passed both ICC(2,1) and ρ analysis. Limited Robustness (LR) features passed ICC(2,1) but were below the threshold set for ρ analysis; this type of response suggested limited discriminative value as the patient ranking did not remain sufficiently constant. Potentially correctable (C) features were below the threshold for ICC(2,1) robustness testing, yet retained highly correlated rankings. This response indicated a potential dependency on interpolation (and/or voxel number) that could possibly be accounted for. Not robust (NR) features scored below the set threshold for both statistical tests.

The difference between feature values from linear interpolation, compared to spline, was assessed with Bland-Altman (BA)^27^ style analysis where the resulting difference for each patient was calculated as a percentage [(Method A − Method B)/Average %]^28^. The 2.7 mm voxel size was used for this comparison. The distribution of the differences for each feature was assessed for normality and detailed box and BA style plots were generated.

### Surface fit correction factor

For those features that were deemed to be potentially correctable as outlined in Fig. 1, an attempt was made to re-scale all values to the 2.7 mm results. To determine a correction factor, models relating the change in feature value to voxel size were built. The feature variation was quantified using the percentage change between values at each voxel size compared to the 2.7 mm result. A surface was fitted to generalise the percentage change in a feature value when extracted at different voxel dimensions. This fit is used to correct the feature to the same scale as the 2.7 mm result. The resulting surface model takes the voxel dimension and feature value as inputs, and outputs a percentage change to be applied to that feature value. A surface normalised feature (*f*_*sn*_) was determined via Eq. (1),

1 $${f}_{sn}=f-(S{M}_{f}(f,{V}_{s})\times f)$$

where *f* is the current feature value, *SM*_*f*_ is the surface model developed for feature type *f*, and *V*_*s*_ is the voxel size in mm^3^ that feature *f* was extracted at. These corrected features were given their original name plus a suffix “*Surface-norm*”. For example, *glrl-rl_NonUniformity* would be referred to as *glrl-rl_NonUniformity-Surface-norm*. Surfaces were generated using MATLAB’s curve fitting toolbox.

### Dividing the dataset

The patient cohort was split randomly (80%/20%) into testing (n = 353) and validation datasets (n = 88), using MATLAB random number functionality. The robustness analysis reported here was performed on the testing dataset. For appropriate features, a surface was fitted to the testing dataset to quantify the relationship between voxel size and feature value, and applied to the unseen validation dataset using Eq. (1). The robustness of these surface normalised features was assessed using the same criteria as the testing dataset (Fig. 1), except they could no longer be categorised as correctable. If they were below the threshold for ICC(2,1) analysis they were not considered robust. For every patient, each VOI was interpolated to the same 6 voxel sizes (i.e. considering the linearly interpolated dataset, the total VOIs undergoing analysis for testing: 6 × 353 = 2118, and for validation: 6 × 88 = 528).

### Ethical statement

Ethical approval was authorised by the Wales Research Ethics Committee 1 (REC reference 14/WA/1208). The methods were carried out in accordance with the relevant guidelines and regulations. Informed consent was not deemed necessary by the REC, given that only pseudo-anonymised data was used.

## Results

### Feature robustness to interpolation

With the linearly interpolated testing dataset, we categorised each features response to interpolation using the criteria of Fig. 1 as follows; 93/141 robust, 6/141 limited robustness, 34/141 potentially correctable and 8/141 not robust. To plot these results we divided the 141 features into two groups; Morphology and first-order features (group-1, 63/141) and texture based features (group-2, 78/141). The categorisations within the two groups were: group-1 (57 R, 3 LR, 3 C, 0 NR) and group-2 (36 R, 3 LR, 31 C, 8 NR).

**Figure 2:**
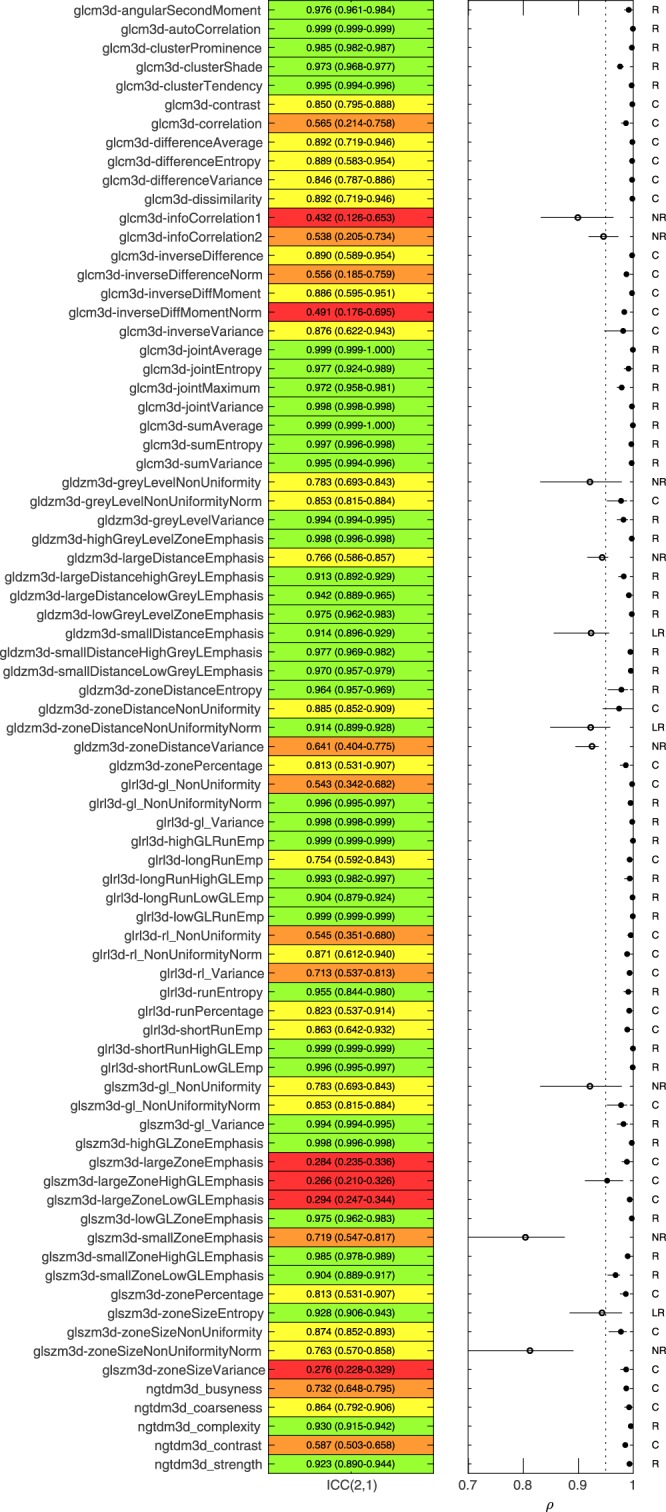
Overview of robustness analysis to linear interpolation for 78 texture features. Left. ICC(2,1) with 95% confidence intervals for each feature. ICC highlighted in green (>0.9), yellow (0.75–0.9), orange (0.5–0.75) and red (<0.5). Right. Pairwise patient ranking correlation between the 2.7 mm rankings and all other voxel sizes for each feature. *ρ*_mean_ > 0.95 is highlighted with a closed dot. Features were categorised as R (Robust), LR, (limited Robustness), C (Potentially Correctable) and NR (Not Robust) based on criteria outlined in Fig. 1.

Figure 2 contains a summary of the robustness of the texture features (group-2) analysed using ICC(2,1) and ρ analysis of patient rankings. ICC(2,1) results are reported and highlighted in green (>0.9), yellow (0.75–0.9), orange (0.5–0.75) and red (<0.5), along with 95% confidence intervals. The range of pairwise spearman rank correlations between patient rankings are also shown. The *ρ*_*mean*_ for each feature was marked with a closed dot if >0.95 and open if not. The corresponding plot for group-1 can be found in supplementary materials. The results were also generated using the spline interpolated testing dataset and can also be found in supplementary materials. The categorisation of all features remained the same when using spline instead of (tri)-linear interpolation.

### Visualising feature response to interpolation

**Figure 3:**
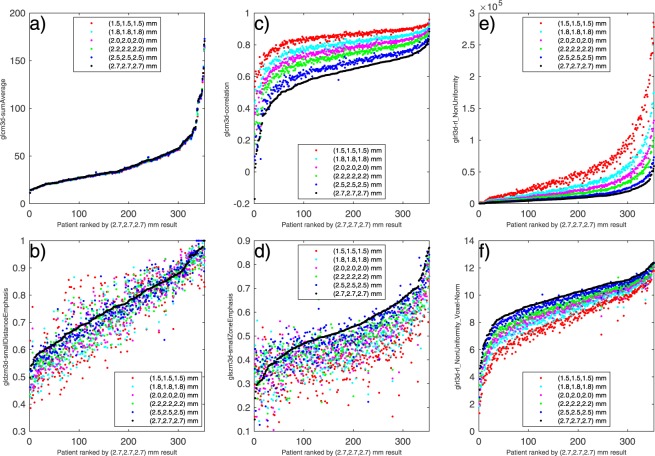
Visualisation of response to linear interpolation for 6 selected features. Patient ranking was taken for the 2.7 *mm* dimension and plotted against the extracted feature values for all voxel dimensions. (**a**) Feature *glcm3d-sumAverage*, considered robust; (**b**) *gldzm3d-smallDistanceEmphasis*, limited robustness; (**c**) *glcm3d-correlation*, considered correctable; (**d**) *glszm3d-smallZoneEmphasis*, considered not robust. (**e**) *glrl3d-rl_NonUniformity*, considered correctable, and (**f**) *glrl3d-rl_NonUniformity-Voxel-Norm*, modified as suggested in^7^ to inherently include voxel number in the feature definition.

Feature responses to interpolation were visualised for each feature by plotting the extracted feature values for every voxel size against the 2.7 mm rank assigned to the patient. Plots for all features can be found in supplementary materials for both linear and spline interpolation. Figure 3 highlights linear interpolated results for 6 features as case studies representing different categories of robustness. Figure 3a shows feature *glcm3d-sumAverage*, considered robust; Fig. 3b is of *gldzm3d-smallDistanceEmphasis*, considered to have limited robustness; Fig. 3c is *glcm3d-correlation*, categorised as correctable, and Fig. 3d is *glszm3d-smallZoneEmphasis* categorised as not robust. Figure 3e,f are of the same feature, *glrl3d-rl_NonUniformity*, as defined in the IBSI and then with the voxel normalisation method suggested by Shafiq-ul-Hassan *et al*.^7^, respectively.

### A surface fit for feature normalization

**Figure 4:**
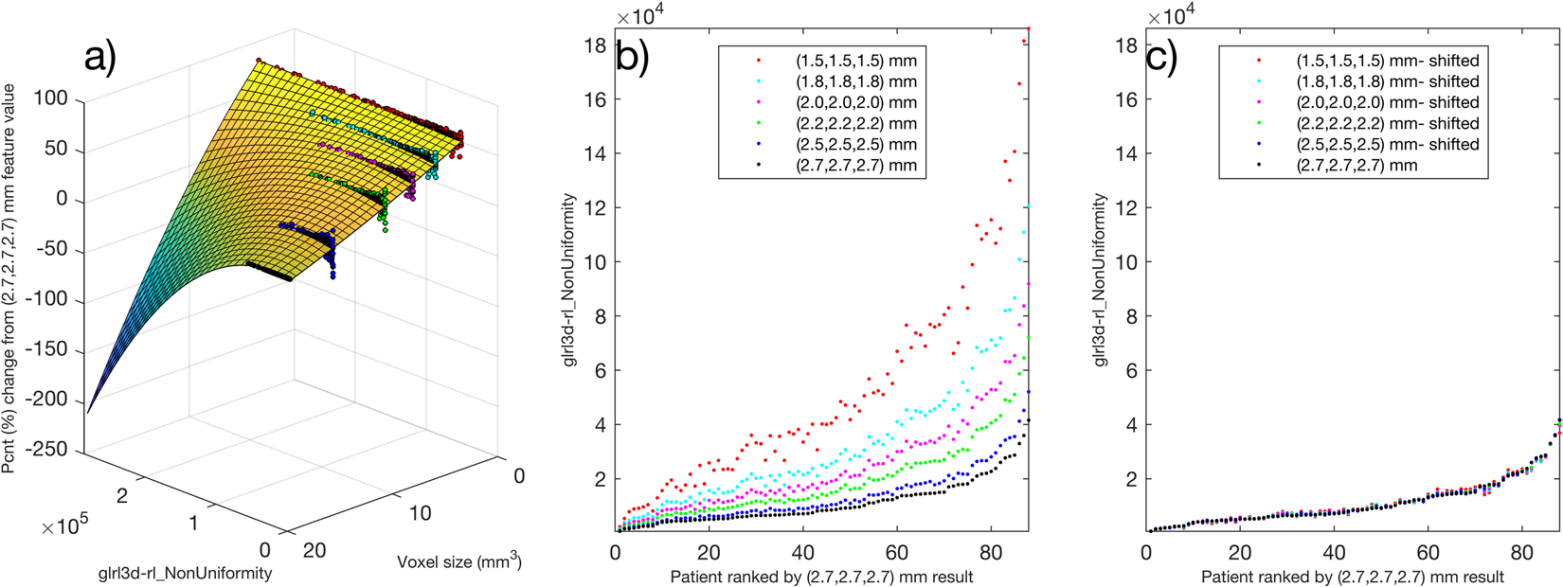
Example of correction technique for feature *glrl3d-rl_NonUniformity* using a surface fit. (**a**) With testing dataset, the percentage change in feature value from the 2.7 mm result was plotted against feature value and voxel size for each patient. (**b**) Validation dataset plotted for feature. (**c**) Applying the correction to the validation dataset using Eq. (1).

**Figure 5:**
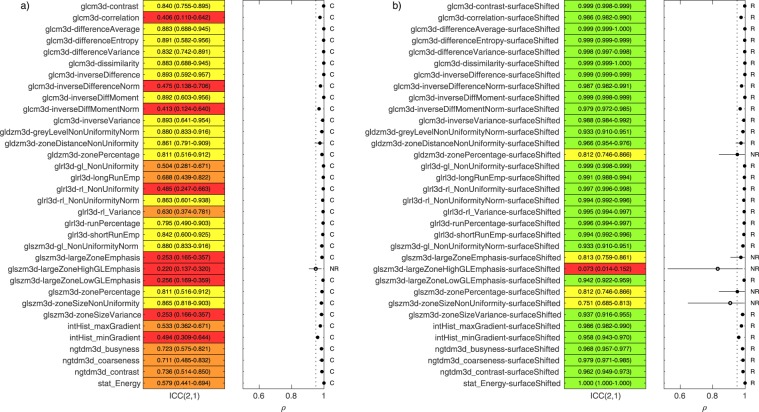
Thirty-four features identified as potentially correctable in the testing dataset were assessed in the validation dataset. The graphical representation of ICC(2,1) and *ρ* analysis is the same as found in Fig. 2. Shown is the validation set (**a**) before correction and (**b**), after correction was applied using the surfaces generated on the testing dataset.

The 34/141 features categorised as correctable due to potential systematic behaviour underwent the surface fitting method for correction described in the methods section. The surfaces (developed using the linear interpolated testing dataset) were used via Eq. (1) to rescale feature values in the validation dataset. The process is demonstrated with feature *glrl3d-rl_NonUniformity* in Fig. 4. A summary of results, before and after correction, for each of the 34 features in the validation dataset is shown in Fig. 5. Before correction, we found for the validation dataset that 33/34 of the features remained categorised as potentially correctable (Fig. 5a). When the correction surfaces were applied, 29/34 features were re-categorised as robust. Five features did not improve or meet the criteria set for robustness after the surface correction approach, and were labelled not robust (Fig. 5b). The surfaces developed for these five features did not generalise from the testing dataset to the validation dataset.

### Linear vs Spline

**Figure 6:**
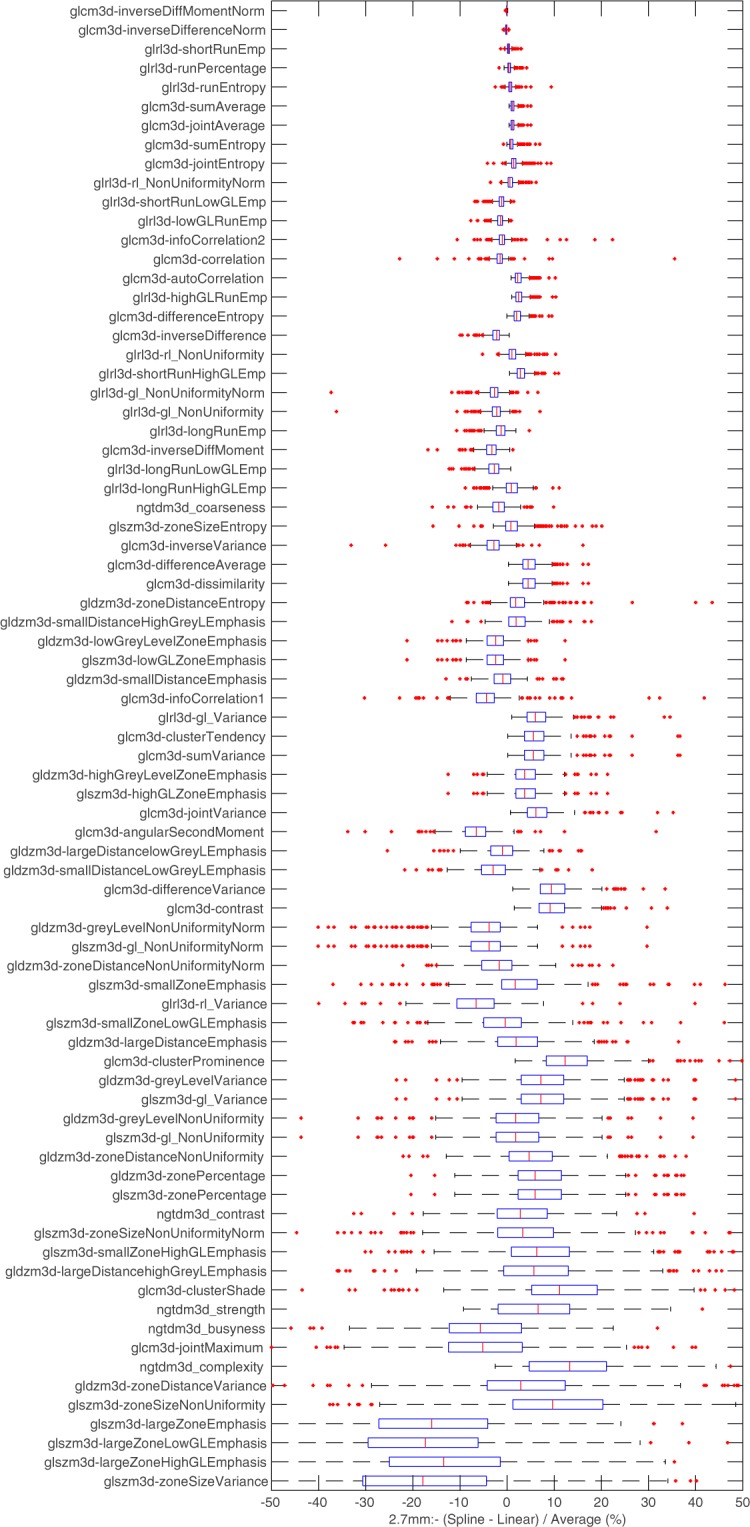
Boxplots summarising texture feature differences measured between (tri)-linear and spline interpolation. Features are ranked based on the interquartile range and the x axis has been limited between +/−50% for readability.

The difference in feature values due to scan interpolation algorithm choice (spline vs linear) was assessed for all features using the 2.7 mm results. For a number of features we found large variation in values between resampling methods. We summarised and ranked the results for the group-2, the 78 texture features in Fig. 6. The Features were ordered based on the interquartile range and the x-axis limited between +/−50% for readability. Additional plots of the distribution of feature differences between spline and linear for all 141 features can be found in supplementary materials. The robustness testing described previously was also repeated using the spline dataset. All feature responses were categorised the same when using spline instead of linear interpolation.

## Discussion

Robustness studies have emerged alongside prognostic and prediction modelling to ensure potential biomarkers are repeatable, reproducible and reliable in a clinical setting. Isotropic image interpolation is a standard processing technique in radiomics, and a necessary step for 3D analysis to reduce directional biased if the voxel sizes are not already isotropic. To the best of our knowledge, focusing entirely on feature variation due to isotropic voxel resampling using PET, this study was the first to attempt to differentiate stable features from those that varied unstably or in a potentially correctable manner.

We combined two statistical techniques, ICC of feature values and *ρ* of patient ranks, to categorise features based on the criteria shown in Fig. 1. The threshold for ICC was set to > 0.9, in-line with other radiomics studies^25,29^, and the 95% confidence levels are reported as recommended by Koo and Li^24^. We selected ICC(2,1), a “2-way mixed-effects model, single rater, absolute agreement” definition as we define features as perfectly stable to interpolation if the same result is computed at each interpolated voxel size. Previous radiomics studies have also used the ICC(3,1) definition which emphasises consistency, and we report both results in the supplementary materials for completeness. Features identified as potentially correctable often had a high ICC(3,1), as the results from each voxel size remained correlated in an additive manner^24^.

Thirty-four features scored below the threshold for ICC testing but retained reliable patient ranking based on *ρ*_mean_ > 0.95. The surface fit normalisation technique we explored is logical to use only if the patient rank remained highly consistent. The threshold could be set higher to increase confidence that a feature is stable or potentially correctable. The threshold for accepted *ρ* was set to a higher limit than in other studies such as Leijenaar *et al*. (>0.9)^22^. For analysis of the patient rankings, we chose the 2.7 mm voxel-size as the ground truth as it was the closest to the axial plane resolution of the original reconstructed PET images. Our categorisation was thus based on the interpolation response as one up-samples scans to smaller isotropic voxel sizes.

We have shown that the surface fit technique implemented in this study was successful in correcting a features dependence to interpolation with the following limitations. We only explored feature robustness for a fixed-bin-width discretisation, as it has been argued to be the most appropriate in a clinical setting^22^. We chose a bin-width of 0.5 SUV following convention from the literature, and the surfaces derived to adjust features may be limited to this discretisation method and width as a result. Additionally, the surfaces are only tested on the linearly interpolated validation data set between the voxel sizes they were generated on and correct only to the 2.7 mm voxel size, our selected ground truth. Each feature has a unique surface fit, and the corrections were only tested on the scanning reconstruction parameters used for our patient cohort.

If possible, adapting the feature algorithms to account for systematic response seen due to interpolation would be a better solution than applying a shift calculated on experimental data. If a feature already has a normalised version that we find to be stable, it would always be preferable to use over any correction shift. Including voxel size inherently in algorithms has been explored in studies that identified “voxel size dependant” features. Shafiq-ul-Hassan *et al*.^6^ assessed the impact of slice thickness and pixel size on features acquired on 116 CT phantom images with different acquisition and reconstruction parameters. Images were resampled to one voxel size ((1 × 1 × 2) mm^3^ using linear interpolation) to determine if this improved robustness. Using coefficient of variation (%COV) as an indicator, 42 out of 213 features studied improved significantly after resampling. Twenty-one features had large variations before and after resampling, and 10 improved significantly after modification to their definitions to remove so-called intrinsic voxel-size dependency. The same group recently expanded on this in another study^7^ to validate the 10 voxel size normalised features on a set of 18 non-small cell lung cancer patients, which were resampled to 4 different pixel sizes and 6 different slice thicknesses. Using the Spearman’s rank coefficient (*ρ*), eight of the features that showed high correlation (*ρ* > 0.9) with voxel numbers before normalisation had low correlation (*ρ* < 0.5) afterwards. We implemented the suggested algorithm adaptation for the feature *glrl3d-rl_NonUniformity*, and visualised the results before and after in Fig. 3e,f respectively. Apparent systematic variation can still be seen when using the adapted algorithm for the range of voxel sizes that we explored, indicating that modifying the feature algorithms to account for systematic response due to isotropic interpolation is challenging.

Our analysis finds large differences in many features extracted when interpolating using a linear method compared to spline. However, the robustness categorisations remained consistent for all features; stable features had stable responses for both interpolation methods. We note that our feature extraction implementation follows the IBSI guidelines, which states the binary mask defining the VOI should always be interpolated linearly^13^. Therefore, morphology features that depend only on the shape of the morphological mask have the same result no matter which algorithm the scan was interpolated with. As such, these features are reported as having no difference between linear as spline.

So far, no consensus has been reached on the most suitable interpolation method for ^18^F-FDG PET imaging. The potential for this choice to cause feature value differences has been previously reported by Yip^30^, who investigated the impact of experimental settings on predicting somatic mutation status, including interpolation methods, voxel sizes and bin widths. Testing 66 features, they found the predictability of 29 to be robust to the choice of experimental settings; they further stressed that the combined effects of these processing methods could be substantial and should be optimised for maximum predictive performance. Our study did not assess interpolation effect on predictive performance of the features, yet we provide a more in-depth feature by feature evaluation of the measured differences using standardised features. For each feature we evaluated the distribution of these differences for normality, and report mean difference and limits of agreement. A feature with large variability between interpolation methods may still show strong predictive significance in a developed radiomics model, it may just be that the thresholds of said model are optimised to the interpolation method that was used. As stressed previously, thorough reporting of feature extraction settings including interpolation method is a necessity for reproducibility and validation.

We have identified robust features showing stability to isotropic interpolation, but this does not necessarily correspond to any clinical application. However, clinically robust and thus relevant features are likely to be a subset of those that have a predictable interpolation response. Therefore, due to the abundance of features in radiomics and the need for reduction techniques to limit overfitting, pruning features that have not shown required interpolation stability may be one of several selection steps to consider for all radiomics studies with multi-centre datasets that requires resampling to a common voxel size.

A large scale, systematic review of repeatability and reproducibility of radiomic features by Traverso *et al*. concluded that there was still currently no emergent pattern or consensus for highly reproducible textural features^31^. Traverso *et al*. also commented that for texture features, coarseness and contrast appeared among the least reproducible. This, of course, assumes that feature definitions are consistent throughout the literature, which again highlights the importance of concepts such as the IBSI, to which we adhere. Interestingly, we also found the IBSI defined features with these names (ngtdm3d_coarseness, ngtdm3d_contrast, glcm3d-contrast) were not robust to interpolation, but they all showed a response that was potentially correctable. Of the studies included in the Traverso *et al*. review, none appear to have categorised the types of feature response to interpolation as we have presented here.

## Conclusion

In this study we assessed the effect of interpolation on radiomic features, for a range of isotropic voxel dimensions, using PET imaging from a large cohort of oesophageal cancer patients. Analysis of 141 common texture features revealed 93 that remained robust after resampling to a constrained range of isotropic voxel sizes. Thirty-four features were below the threshold for ICC analysis, but retained highly correlated patient rankings and were deemed potentially correctable. A correction model was developed, feature by feature, and performed well for 29 features in a validation dataset. Eight features were identified as not robust, behaving unstably due to interpolation, and should be used with caution in radiomics studies that require resampling to common voxel sizes. Finally, large variations were measured in many features values when selecting between linear and spline interpolation methods, yet the overall response categorisations for all features remained the same.

## Supplementary information

Supplementary Materials

Supplementary results table

## Data Availability

Original imaging data cannot be shared. Visualisations of the datasets and feature extraction results are available on request, or can be found in the Cardiff University data catalogue at 10.17035/d.2019.0078762440.
