# Supplementary material for: Assessing radiomic feature robustness to interpolation in 18F-FDG PET imaging
Source: Sci Rep. 2019 Jul 4;9:9649. doi: 10.1038/s41598-019-46030-0 (PMC6609613; doi:10.1038/s41598-019-46030-0)
Supplement: Supplementary file 1 — Supplementary Materials [file 41598_2019_46030_MOESM1_ESM.pdf]

Supplementary materials:

'Assessing radiomic feature robustness to interpolation in ^{18}F-FDG PET imaging'

Philip Whybra*[1], Craig Parkinson[1], Kieran Foley[2], John Staffurth[2], and Emiliano Spezi[1,2]

Corresponding author* (whybrap@cardiff.ac.uk)
[1] School of Engineering, Cardiff University, United Kingdom
[2] Velindre Cancer Centre, Cardiff, United Kingdom.

Contents

1 Reporting Feature Extraction Settings **6**
1.1 Imaging . . . 6
1.2 Acquisition / Reconstruction . . . 6
1.3 Software . . . 6
1.4 Data availability . . . 6
1.5 Data conversion . . . 6
1.6 Segmentation . . . 6
1.7 Interpolation . . . 6
1.8 Discretisation . . . 7
1.9 Feature calculation . . . 7
1.10 Feature list . . . 8

2 Robustness analysis with Linear interpolation **11**
2.1 Morphological & first order (Linear) . . . 11
2.2 Texture results (Linear) . . . 12

3 Repeating Robustness analysis with Spline interpolation **13**
3.1 Morphological & first order (Spline) . . . 13
3.2 Texture results (Spline) . . . 14

4 Visualising Feature Response to Interpolation **15**
4.1 Plot explanation . . . 15
4.2 Feature by feature plots . . . 15

5 Modelling Interpolation response **88**
5.1 Plot explanation . . . 88
5.2 Surface fit corrections . . . 89

Bibliography **105**

List of Figures

1 Group-1 robustness results (linear interpolation). . . . 11
2 Group-2 robustness results (linear interpolation). . . . 12
3 Group-1 robustness results (Spline interpolation). . . . 13
4 Group-2 robustness results (Spline interpolation). . . . 14
5 glcm3d-angularSecondMoment . . . 16
6 glcm3d-autoCorrelation . . . 17
7 glcm3d-clusterProminence . . . 17
8 glcm3d-clusterShade . . . 18
9 glcm3d-clusterTendency . . . 18
10 glcm3d-contrast . . . 19
11 glcm3d-correlation . . . 19
12 glcm3d-differenceAverage . . . 20
13 glcm3d-differenceEntropy . . . 20
14 glcm3d-differenceVariance . . . 21

15 glcm3d-dissimilarity . . . 21
16 glcm3d-infoCorrelation1 . . . 22
17 glcm3d-infoCorrelation2 . . . 22
18 glcm3d-inverseDifference . . . 23
19 glcm3d-inverseDifferenceNorm . . . 23
20 glcm3d-inverseDiffMoment . . . 24
21 glcm3d-inverseDiffMomentNorm . . . 24
22 glcm3d-inverseVariance . . . 25
23 glcm3d-jointAverage . . . 25
24 glcm3d-jointEntropy . . . 26
25 glcm3d-jointMaximum . . . 26
26 glcm3d-jointVariance . . . 27
27 glcm3d-sumAverage . . . 27
28 glcm3d-sumEntropy . . . 28
29 glcm3d-sumVariance . . . 28
30 gldzm3d-greyLevelNonUniformity . . . 29
31 gldzm3d-greyLevelNonUniformityNorm . . . 29
32 gldzm3d-greyLevelVariance . . . 30
33 gldzm3d-highGreyLevelZoneEmphasis . . . 30
34 gldzm3d-largeDistanceEmphasis . . . 31
35 gldzm3d-largeDistancehighGreyLEmphasis . . . 31
36 gldzm3d-largeDistancelowGreyLEmphasis . . . 32
37 gldzm3d-lowGreyLevelZoneEmphasis . . . 32
38 gldzm3d-smallDistanceEmphasis . . . 33
39 gldzm3d-smallDistanceHighGreyLEmphasis . . . 33
40 gldzm3d-smallDistanceLowGreyLEmphasis . . . 34
41 gldzm3d-zoneDistanceEntropy . . . 34
42 gldzm3d-zoneDistanceNonUniformity . . . 35
43 gldzm3d-zoneDistanceNonUniformityNorm . . . 35
44 gldzm3d-zoneDistanceVariance . . . 36
45 gldzm3d-zonePercentage . . . 36
46 glrl3d-gl-NonUniformity . . . 37
47 glrl3d-gl-NonUniformityNorm . . . 37
48 glrl3d-gl-Variance . . . 38
49 glrl3d-highGLRunEmp . . . 38
50 glrl3d-longRunEmp . . . 39
51 glrl3d-longRunHighGLEmp . . . 39
52 glrl3d-longRunLowGLEmp . . . 40
53 glrl3d-lowGLRunEmp . . . 40
54 glrl3d-rl-NonUniformity . . . 41
55 glrl3d-rl-NonUniformityNorm . . . 41
56 glrl3d-rl-Variance . . . 42
57 glrl3d-runEntropy . . . 42
58 glrl3d-runPercentage . . . 43
59 glrl3d-shortRunEmp . . . 43
60 glrl3d-shortRunHighGLEmp . . . 44
61 glrl3d-shortRunLowGLEmp . . . 44

62 glszm3d-gl-NonUniformity 45
63 glszm3d-gl-NonUniformityNorm 45
64 glszm3d-gl-Variance 46
65 glszm3d-highGLZoneEmphasis 46
66 glszm3d-largeZoneEmphasis 47
67 glszm3d-largeZoneHighGLEmphasis 47
68 glszm3d-largeZoneLowGLEmphasis 48
69 glszm3d-lowGLZoneEmphasis 48
70 glszm3d-smallZoneEmphasis 49
71 glszm3d-smallZoneHighGLEmphasis 49
72 glszm3d-smallZoneLowGLEmphasis 50
73 glszm3d-zonePercentage 50
74 glszm3d-zoneSizeEntropy 51
75 glszm3d-zoneSizeNonUniformity 51
76 glszm3d-zoneSizeNonUniformityNorm 52
77 glszm3d-zoneSizeVariance 52
78 ngtdm3d-busyness 53
79 ngtdm3d-coarseness 53
80 ngtdm3d-complexity 54
81 ngtdm3d-contrast 54
82 ngtdm3d-strength 55
83 intHist-coefficientofVariation 56
84 intHist-entropy 57
85 intHist-IQR 57
86 intHist-kurtosis 58
87 intHist-maxGradient 58
88 intHist-maxGradientGreyLevel 59
89 intHist-maxGreyLevel 59
90 intHist-mean 60
91 intHist-meanAbsoluteDeviation 60
92 intHist-median 61
93 intHist-medianAbsoluteDeviation 61
94 intHist-minGradient 62
95 intHist-minGradientGreyLevel 62
96 intHist-minGreyLevel 63
97 intHist-mode 63
98 intHist-percentile10 64
99 intHist-percentile90 64
100 intHist-quartileCoefDispersion 65
101 intHist-range 65
102 intHist-robustMeanAbsoluteDeviation 66
103 intHist-skewness 66
104 intHist-uniformity 67
105 intHist-variance 67
106 morph-areaDensity-aabb 68
107 morph-areaDensity-aee 68
108 morph-areaDensity-convexHull 69

109 morph-asphericity 69
110 morph-compactness1 70
111 morph-compactness2 70
112 morph-COMshift 71
113 morph-elongation 71
114 morph-flatness 72
115 morph-integratedIntensity 72
116 morph-leastAxisLength 73
117 morph-majorAxisLength 73
118 morph-max3Ddiameter 74
119 morph-minorAxisLength 74
120 morph-sphericalDisproportion 75
121 morph-sphericity 75
122 morph-surfaceArea 76
123 morph-surfAreaToVolumeRatio 76
124 morph-volDensity-aabb 77
125 morph-volDensity-aee 77
126 morph-volDensity-convexHull 78
127 morph-volume 78
128 stat-coefficientofVariation 79
129 stat-Energy 79
130 stat-IQR 80
131 stat-kurtosis 80
132 stat-maxGreyLevel 81
133 stat-mean 81
134 stat-meanAbsoluteDeviation 82
135 stat-median 82
136 stat-medianAbsoluteDeviation 83
137 stat-minGreyLevel 83
138 stat-percentile10 84
139 stat-percentile90 84
140 stat-quartileCoefDispersion 85
141 stat-range 85
142 stat-robustMeanAbsoluteDeviation 86
143 stat-rootMeanSquare 86
144 stat-skewness 87
145 stat-variance 87
146 Modelling response: glcm3d-contrast 89
147 Modelling response: glcm3d-correlation 89
148 Modelling response: glcm3d-differenceAverage 89
149 Modelling response: glcm3d-differenceEntropy 90
150 Modelling response: glcm3d-differenceVariance 90
151 Modelling response: glcm3d-dissimilarity 90
152 Modelling response: glcm3d-inverseDifference 91
153 Modelling response: glcm3d-inverseDifferenceNorm 91
154 Modelling response: glcm3d-inverseDiffMoment 92
155 Modelling response: glcm3d-inverseDiffMomentNorm 92

156 Modelling response: glcm3d-inverseVariance 93
157 Modelling response: gldzm3d-greyLevelNonUniformityNorm 93
158 Modelling response: gldzm3d-zoneDistanceNonUniformity 94
159 Modelling response: gldzm3d-zonePercentage 94
160 Modelling response: glrl3d-gl-NonUniformity 95
161 Modelling response: glrl3d-longRunEmp 95
162 Modelling response: glrl3d-rl-NonUniformity 96
163 Modelling response: glrl3d-rl-NonUniformityNorm 96
164 Modelling response: glrl3d-rl-Variance 97
165 Modelling response: glrl3d-runPercentage 97
166 Modelling response: glrl3d-shortRunEmp 98
167 Modelling response: glszm3d-gl-NonUniformityNorm 98
168 Modelling response: glszm3d-largeZoneEmphasis 99
169 Modelling response: glszm3d-largeZoneHighGLEmphasis 99
170 Modelling response: glszm3d-largeZoneLowGLEmphasis 100
171 Modelling response: glszm3d-zonePercentage 100
172 Modelling response: glszm3d-zoneSizeNonUniformity 101
173 Modelling response: glszm3d-zoneSizeVariance 101
174 Modelling response: intHist-maxGradient 102
175 Modelling response: intHist-minGradient 102
176 Modelling response: ngtdm3d-busyness 103
177 Modelling response: ngtdm3d-coarseness 103
178 Modelling response: ngtdm3d-contrast 104
179 Modelling response: stat-Energy 104

1 Reporting Feature Extraction Settings

1.1 Imaging

^{18}F-FDG PET / CT imaging. This work utilises the PET component.

1.2 Acquisition / Reconstruction

A GE 690 PET/CT scanner (GE Healthcare, Buckinghamshire, UK) was used for all patients. Patients were fasted for at least 6 hours prior to tracer administration. Serum glucose levels were routinely checked and confirmed as less than 7.0 mmol/L prior to imaging. Patients received a dose of 4 MBq of 18F-FDG/kg. Uptake time was 90 minutes, which is standard practice at our institution. PET images were acquired at 3 minutes per field of view. The length of the axial field of view was 15.7 cm (skull base to mid-thigh). Images were reconstructed with the ordered subset expectation maximisation algorithm, with 24 subsets and 2 iterations. Matrix size was 256 x 256 pixels, using the VUE PointTM time of flight algorithm. CT images were acquired in a helical acquisition with a pitch of 0.98 and tube rotation speed of 0.5 seconds. Tube output was 120 kVp with output modulation between 20 and 200 mA. Matrix size for the CT acquisition was 512 x 512 pixels with a 50 cm field of view. No oral or intravenous contrast was administered. Further details can be found in Foley *et al.* [1].

1.3 Software

CERR (Computational Environment for Radiological Research)[2] was used to import DICOM files into Matlab. Feature extraction was done with SPAARC (Spaarc Pipeline for Automated Analysis and Radiomics Computing), an in-house software built on the MATLAB platform (MathWorks, Natick, MA, USA).

1.4 Data availability

Original DICOM data cannot be made publically available at this time. However, visualisations of Scan VOIs are available on request.

1.5 Data conversion

PET imaging was converted from Becquerel/ml (Bq/ml) to Standardised Uptake Value (SUV) using CERR functionality that pulls the necessary information for conversion from the DICOM image tags.

1.6 Segmentation

The PET VOIs were segmented using ATLAAS [3]. Contours were rated by an expert radiologist (author KF) using a binary score (acceptable=1, unacceptable=0). Segmentations were performed on the original scan dimension 2.73 x 2.73 x 3.27 mm.

1.7 Interpolation

This study assessed isotropic interpolation to voxel dimensions of 2.7mm, 2.5mm, 2.2mm, 2.0mm, 1.8mm, and 1.5mm. Both tri-linear and spline interpolation methods were explored. The mask defining the VOI was always interpolated linearly, following IBSI guidelines [4].

1.8 Discretisation

We used a *fixed bin width* method to discretise the scan values [4]. The bin width was set to 0.5 SUV.

1.9 Feature calculation

Feature extraction parameters are detailed in Table 1. All Algorithm definitions can be found in IBSI documentation [4]. A list of the full feature names can be see in Table 2. SPAARC feature algorithms have been validated on a digital phantom and patient test data as part of the IBSI [4].

Table 1: Feature extraction parameters.

Texture Matrix	Parameters
grey level cooccurrence matrix (GLCM)	Distance = 1 voxel.<br>Directions = 13 unique directions of the 26-connected neighbours.<br>Matrix merging method = Merged GLCMs for each direction and calculated features on single matrix.
grey level run length matrix (GLRL)	Distance = 1 voxel,.<br>Directions = 13 unique direction vectors.<br>Matrix merging method = Merged GLRLs for each direction and calculated features on single matrix.
grey level size zone matrix (GLSZM)	Connectivity for all 26 neighbours in 3D.
grey level distance zone matrix (GLDZM)	Connectivity for all 26 neighbours in 3D.<br>Distance map generated with 6-connectivity.
neighbourhood grey tone difference matrix (NGTDM)	Distance = 1 voxel.

1.10 Feature list

Table 2: Feature names and corresponding code

Type	Feature Name	Code
GLCM	Angular Second Moment	glcm3d-angularSecondMoment
	Auto Correlation	glcm3d-autoCorrelation
	Cluster Prominence	glcm3d-clusterProminence
	Cluster Shade	glcm3d-clusterShade
	Cluster Tendency	glcm3d-clusterTendency
	Contrast	glcm3d-contrast
	Correlation	glcm3d-correlation
	Difference Average	glcm3d-differenceAverage
	Difference Entropy	glcm3d-differenceEntropy
	Difference Variance	glcm3d-differenceVariance
	Dissimilarity	glcm3d-dissimilarity
	First measure of information correlation	glcm3d-infoCorrelation1
	Second measure of information correlation	glcm3d-infoCorrelation2
	Inverse difference	glcm3d-inverseDifference
	Inverse difference normalised	glcm3d-inverseDifferenceNorm
	Inverse difference moment	glcm3d-inverseDiffMoment
	Inverse difference moment normalised	glcm3d-inverseDiffMomentNorm
	Inverse Variance	glcm3d-inverseVariance
	Joint Average	glcm3d-jointAverage
	Joint Entropy	glcm3d-jointEntropy
	Joint Maximum	glcm3d-jointMaximum
	Joint Variance	glcm3d-jointVariance
	Sum Average	glcm3d-sumAverage
	Sum Entropy	glcm3d-sumEntropy
	Sum Variance	glcm3d-sumVariance
GLDZM	Grey Level Non-uniformity	gldzm3d-greyLevelNonUniformity
	Grey Level Non-uniformity Normalised	gldzm3d-greyLevelNonUniformityNorm
	Grey Level Variance	gldzm3d-greyLevelVariance
	High Grey Level Zone Emphasis	gldzm3d-highGreyLevelZoneEmphasis
	Large Distance Emphasis	gldzm3d-largeDistanceEmphasis
	Large Distance High Grey Level Emphasis	gldzm3d-largeDistancehighGreyLEmphasis
	Large Distance Low Grey Level Emphasis	gldzm3d-largeDistancelowGreyLEmphasis
	Low Grey Level Zone Emphasis	gldzm3d-lowGreyLevelZoneEmphasis
	Small Distance Emphasis	gldzm3d-smallDistanceEmphasis
	Small Distance High Grey Level Emphasis	gldzm3d-smallDistanceHighGreyLEmphasis
	Small Distance Low Grey Level Emphasis	gldzm3d-smallDistanceLowGreyLEmphasis
	Zone Distance Entropy	gldzm3d-zoneDistanceEntropy
	Zone Distance Non-Uniformity	gldzm3d-zoneDistanceNonUniformity
	Zone Distance Non-Uniformity Normalised	gldzm3d-zoneDistanceNonUniformityNorm
	Zone Distance Variance	gldzm3d-zoneDistanceVariance
	Zone Percentage	gldzm3d-zonePercentage
GLRL	Grey Level Non-uniformity	glrl3d-gl_NonUniformity
	Grey Level Non-uniformity Normalised	glrl3d-gl_NonUniformityNorm
	Grey Level Variance	glrl3d-gl_Variance
	High Grey Level Run Emphasis	glrl3d-highGLRunEmp
	Long Runs Emphasis	glrl3d-longRunEmp
	Long Run High Grey Level Emphasis	glrl3d-longRunHighGLEmp
	Long Run Low Grey Level Emphasis	glrl3d-longRunLowGLEmp
	Low Grey Level Run Emphasis	glrl3d-lowGLRunEmp
	Run Length Non-uniformity	glrl3d-rl_NonUniformity
	Run Length Non-uniformity Normalised	glrl3d-rl_NonUniformityNorm
	Run Length Variance	glrl3d-rl_Variance
	Run Entropy	glrl3d-runEntropy
	Run Percentage	glrl3d-runPercentage
	Short Run Emphasis	glrl3d-shortRunEmp
	Short Run High Grey Level Emphasis	glrl3d-shortRunHighGLEmp
	Short Run Low Grey Level Emphasis	glrl3d-shortRunLowGLEmp
GLSZM	Grey Level Non-uniformity	glszm3d-gl_NonUniformity
	Grey Level Non-uniformity Normalised	glszm3d-gl_NonUniformityNorm
	Grey Level Variance	glszm3d-gl_Variance
	High Grey Level Zone Emphasis	glszm3d-highGLZoneEmphasis
	Large Zone Emphasis	glszm3d-largeZoneEmphasis
	Large Zone High Grey Level Emphasis	glszm3d-largeZoneHighGLEmphasis
	Large Zone Low Grey Level Emphasis	glszm3d-largeZoneLowGLEmphasis

	Low Grey Level Zone Emphasis	glszm3d-lowGLZoneEmphasis
	Small Zone Emphasis	glszm3d-smallZoneEmphasis
	Small Zone High Grey Level Emphasis	glszm3d-smallZoneHighGLEmphasis
	Small Zone Low Grey Level Emphasis	glszm3d-smallZoneLowGLEmphasis
	Zone Percentage	glszm3d-zonePercentage
	Zone Size Entropy	glszm3d-zoneSizeEntropy
	Zone Size Non-uniformity	glszm3d-zoneSizeNonUniformity
	Zone Size Non-uniformity Normalised	glszm3d-zoneSizeNonUniformityNorm
	Zone Size Variance	glszm3d-zoneSizeVariance
NGTDM	Busyness	ngtdm3d_busyness
	Coarseness	ngtdm3d_coarseness
	Complexity	ngtdm3d_complexity
	Contrast	ngtdm3d_contrast
	Strength	ngtdm3d_strength
Intensity Histogram	Coefficient of Variation	intHist_coefficientofVariation
	Entropy	intHist_entropy
	Interquartile range	intHist_IQR
	Kurtosis	intHist_kurtosis
	Maximum histogram gradient	intHist_maxGradient
	Maximum gradient grey level	intHist_maxGradientGreyLevel
	Maximum	intHist_maxGreyLevel
	Mean	intHist_mean
	Mean absolute deviation	intHist_meanAbsoluteDeviation
	Median	intHist_median
	Median absolute deviation	intHist_medianAbsoluteDeviation
	Minimum histogram gradient	intHist_minGradient
	Minimum gradient grey level	intHist_minGradientGreyLevel
	Minimum	intHist_minGreyLevel
	Mode	intHist_mode
	10th percentile	intHist_percentile10
	90th percentile	intHist_percentile90
	Quartile coefficient of dispersion	intHist_quartileCoefDispersion
	Range	intHist_range
	Robust mean absolute deviation	intHist_robustMeanAbsoluteDeviation
	Skewness	intHist_skewness
	Uniformity	intHist_uniformity
	Variance	intHist_variance
Morphology	Area density (AABB)	morph_areaDensity_aabb
	Area density (AEE)	morph_areaDensity_aee
	Area density (convex hull)	morph_areaDensity_convexHull
	Asphericity	morph_asphericity
	Compactness 1	morph_compactness1
	Compactness 2	morph_compactness2
	Centre of mass shift	morph_COMshift
	Elongation	morph_elongation
	Flatness	morph_flatness
	Integrated intensity	morph_integratedIntensity
	Least axis length	morph_leastAxisLength
	Major axis length	morph_majorAxisLength
	Maximum 3D diameter	morph_max3Ddiameter
	Minor axis length	morph_minorAxisLength
	Spherical disproportion	morph_sphericalDisproportion
	Sphericity	morph_sphericity
	Surface area	morph_surfaceArea
	Surface to volume ratio	morph_surfAreaToVolumeRatio
	Volume density (AABB)	morph_volDensity_aabb
	Volume density (AEE)	morph_volDensity_aee
	Volume density (convex hull)	morph_volDensity_convexHull
	Volume (mesh-based)	morph_volume
Statistical	Coefficient of Variation	stat_coefficientofVariation
	Entropy	stat_Energy
	Interquartile range	stat_IQR
	Kurtosis	stat_kurtosis
	Maximum	stat_maxGreyLevel
	Mean	stat_mean
	Mean absolute deviation	stat_meanAbsoluteDeviation
	Median	stat_median
	Median absolute deviation	stat_medianAbsoluteDeviation
	Minimum	stat_minGreyLevel

10th percentile	stat_percentile10
90th percentile	stat_percentile90
Quartile coefficient of dispersion	stat_quartileCoefDispersion
Range	stat_range
Robust mean absolute deviation	stat_robustMeanAbsoluteDeviation
Root mean square	stat_rootMeanSquare
Skewness	stat_skewness
Variance	stat_variance

2 Robustness analysis with Linear interpolation

2.1 Morphological & first order (Linear)

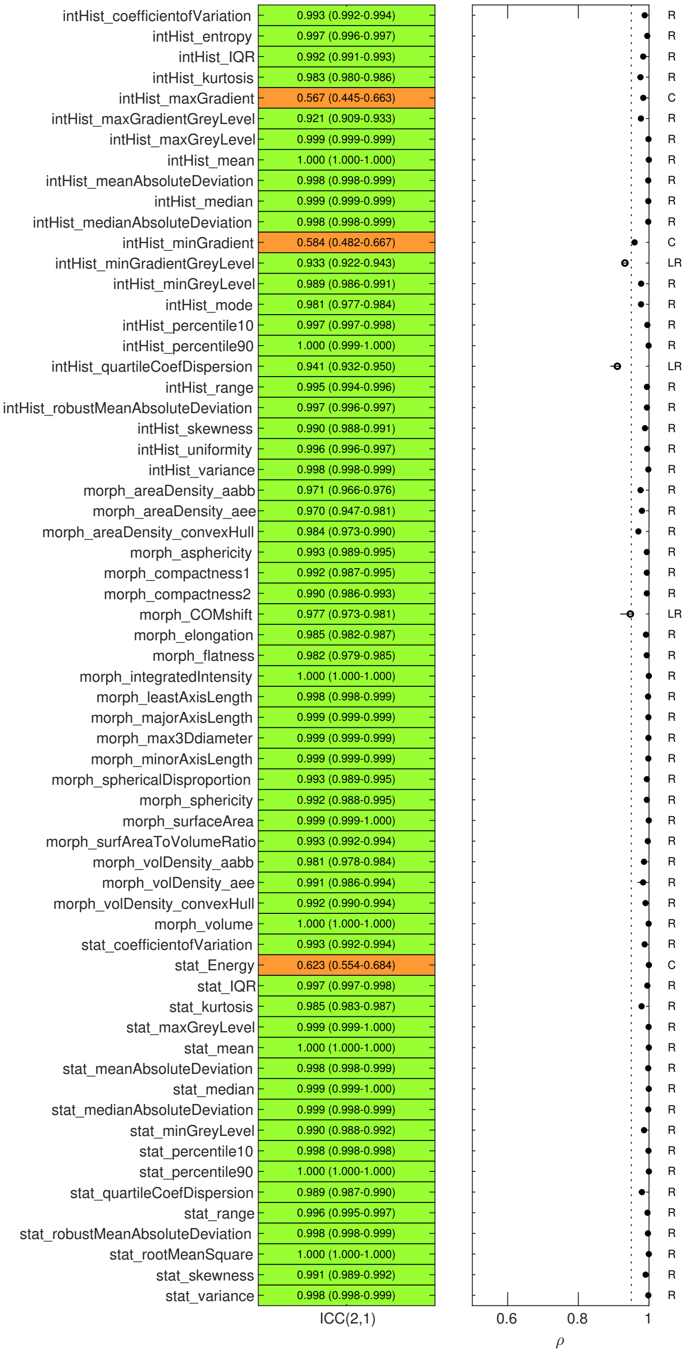

Figure 1: Group-1 robustness results (linear interpolation). Analysis of morphological and first order features. These results are in the same style as Fig.2 in the main manuscript.

2.2 Texture results (Linear)

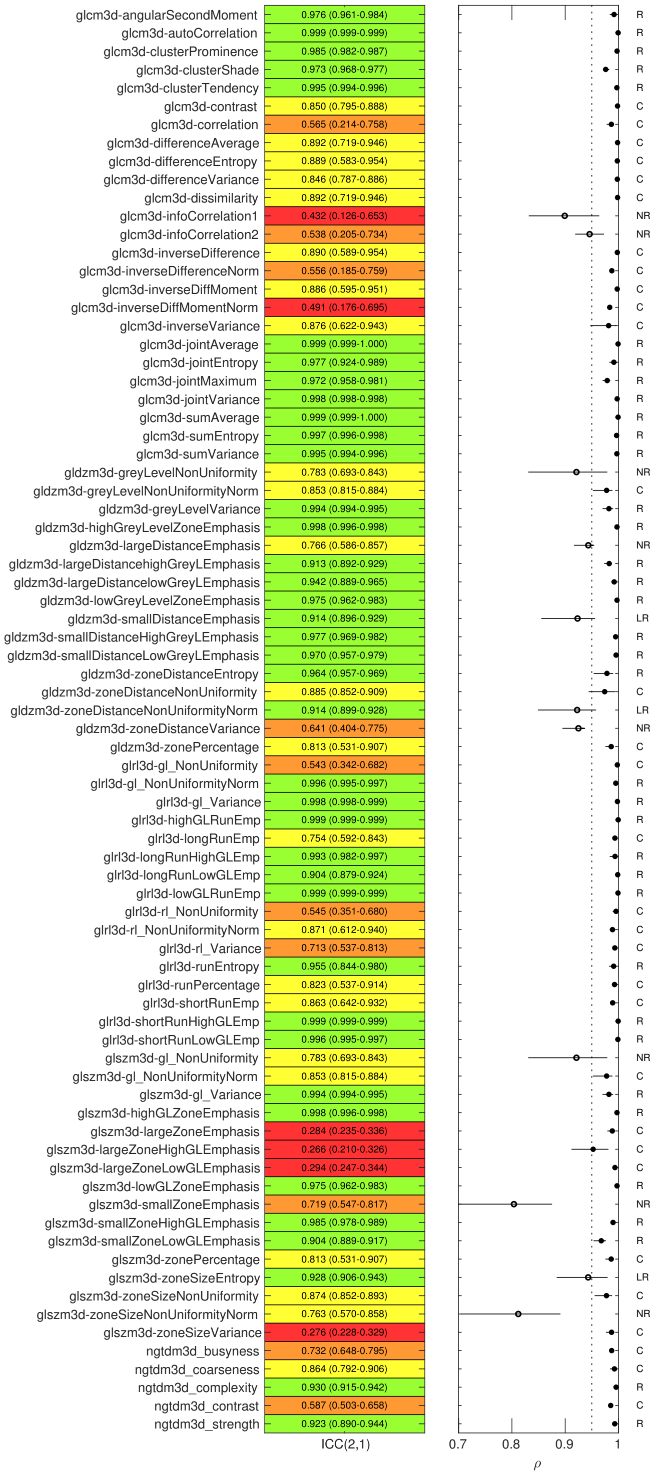

Figure 2: Group-2 robustness results (Linear interpolation). Same as Fig.2 in main manuscript, put here for completeness.

3 Repeating Robustness analysis with Spline interpolation

3.1 Morphological & first order (Spline)

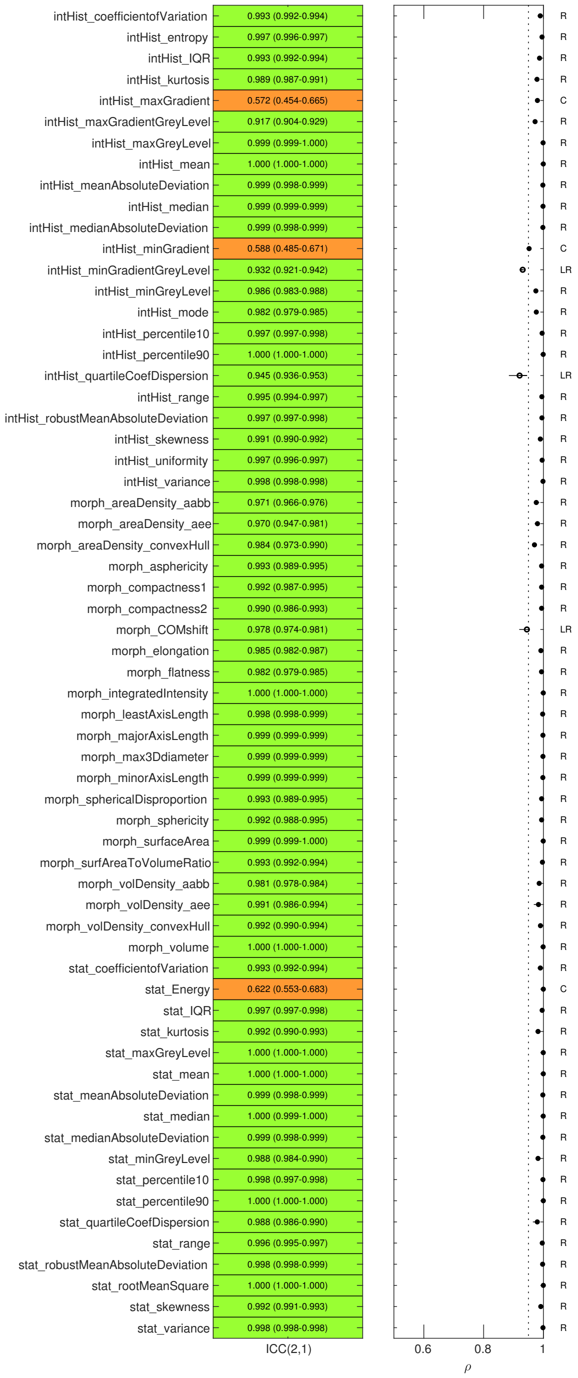

Figure 3: Group-1 robustness results (Spline interpolation). Morphological and first order features.

NOTE: Morphological features that depend only on the mask and not the scan values will always have exactly the same results between linear and spline because the binary mask defining the VOI is always interpolated linearly in our implementation (as per IBSI recommendations).

3.2 Texture results (Spline)

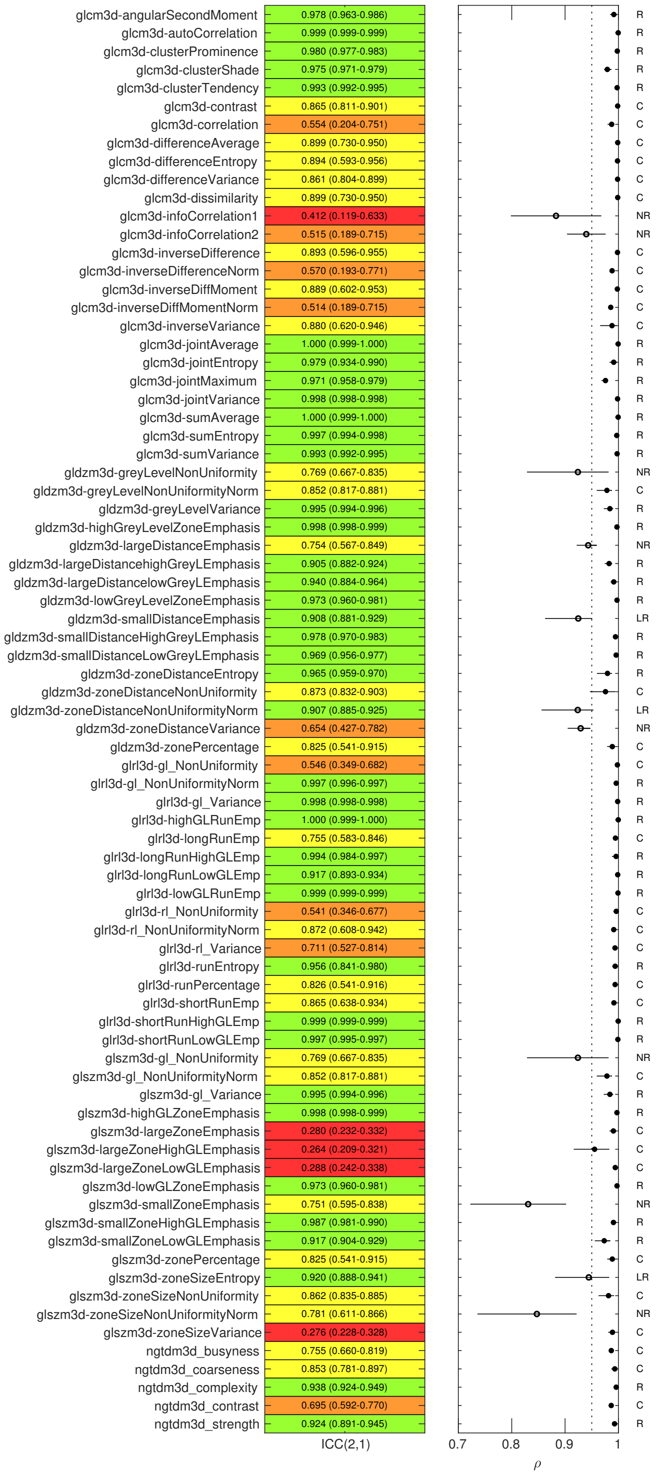

Figure 4: Group-2 robustness results (Spline interpolation). Texture features. These results are generated in the same was as Fig.2 in the main manuscript but with Spline interpolation used.

4 Visualising Feature Response to Interpolation

For all 141 features we visualised the change in feature values when resampling to different isotropic voxel sizes, using both linear and spline methods (examples of this are used in Figure 3 of main manuscript). These results were generated using the testing datasets. We also compared the difference in feature values between linear and spline methods for the 2.7mm voxel size results.

4.1 Plot explanation

Each of the 141 features has 6 plots, labelled a to f, that are summarised as follows:

a) (Linear interpolation) – Visualisation of feature response to voxel-size resampling. Each patient was given a rank based on the results of 2.7mm dimension which was plotted against the extracted feature values for all voxel dimensions.

b) (Spline interpolation) – Visualisation of feature response to voxel-size resampling. Patient ranking was kept from the linear results to enable a direct comparison between plots a) and b).

c) Spline Vs Linear – For the 2.7mm voxel-size, the spline results were plotted against the linear results.

d) Spline Vs Linear – Differences in feature values for 2.7mm voxel-size, [(Spline - Linear) / Average %], plotted as a histogram. An Anderson-Darling test was used to assess normality (Matlab function adtest). Null hypothesis is that sample is drawn from a normal distribution.

e) Spline Vs Linear – Boxplot representation of the difference in feature values between linear and spline for 2.7mm voxel-size.

f) Spline Vs Linear – Bland-Altman style analysis for 2.7mm voxel-size: The difference in feature value is plotted against the average value. Limits of agreement are set based on a normality test. If the distribution is normal, we used $mean \pm 1.96 \times sd$. If the distribution is not normal, we used 2.5 and 97.5 percentile limits.

4.2 Feature by feature plots

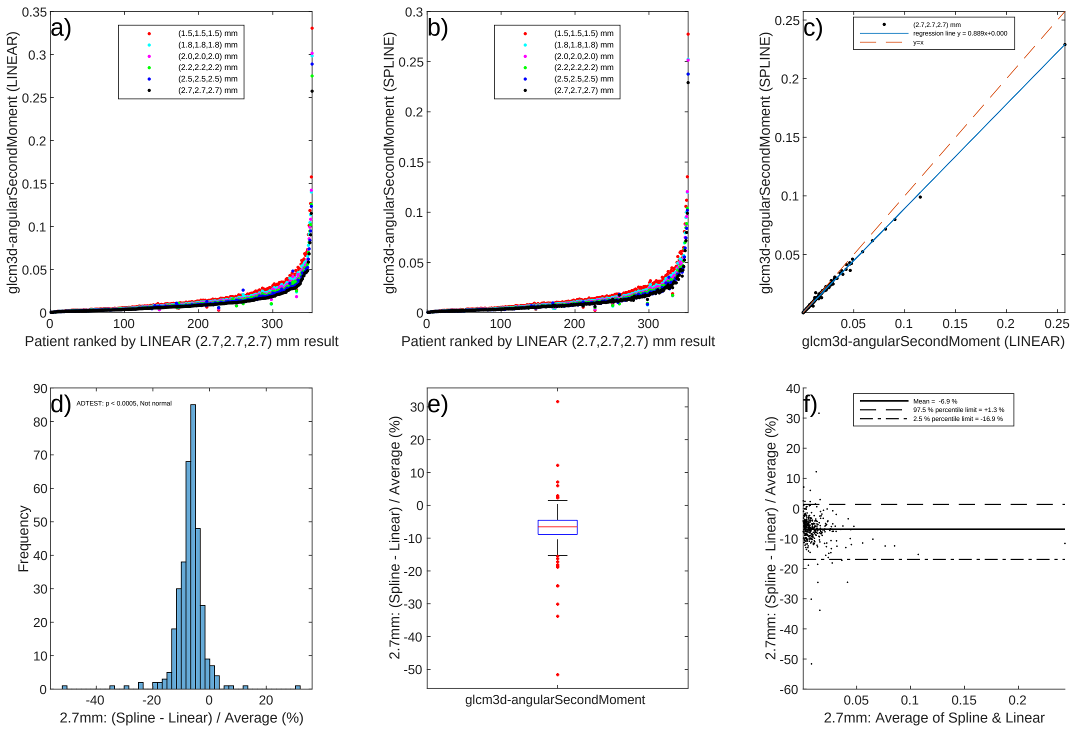

Figure 5: glcm3d-angularSecondMoment

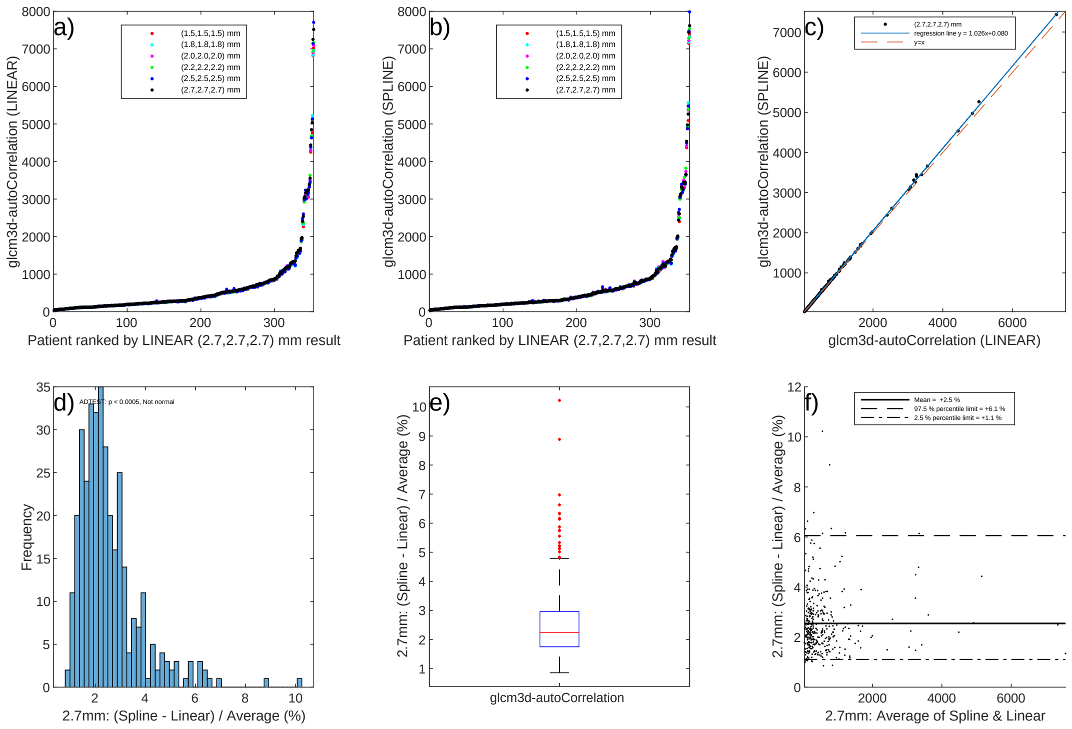

Figure 6: glcm3d-autoCorrelation

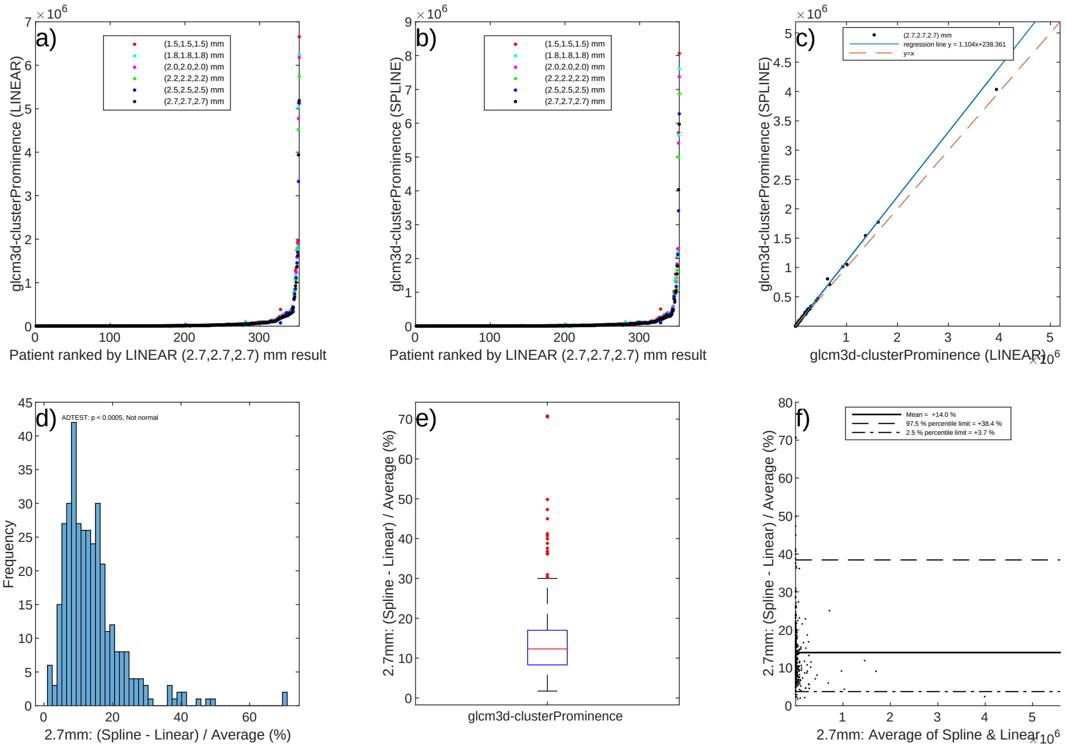

Figure 7: glcm3d-clusterProminence

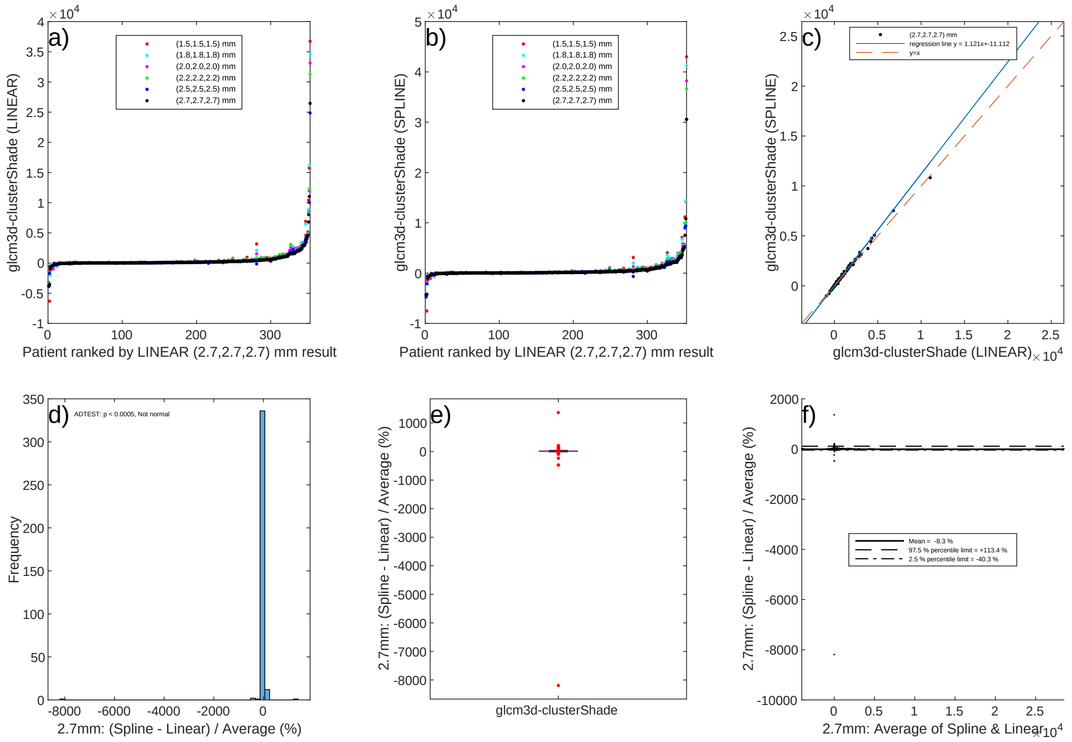

Figure 8: glcm3d-clusterShade

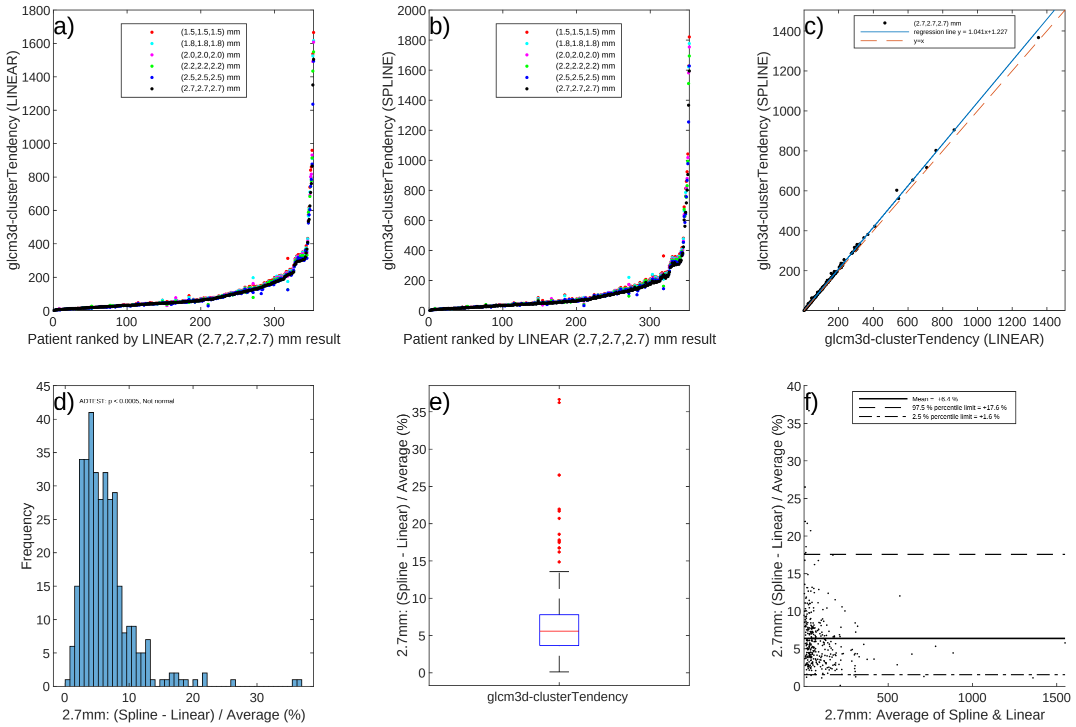

Figure 9: glcm3d-clusterTendency

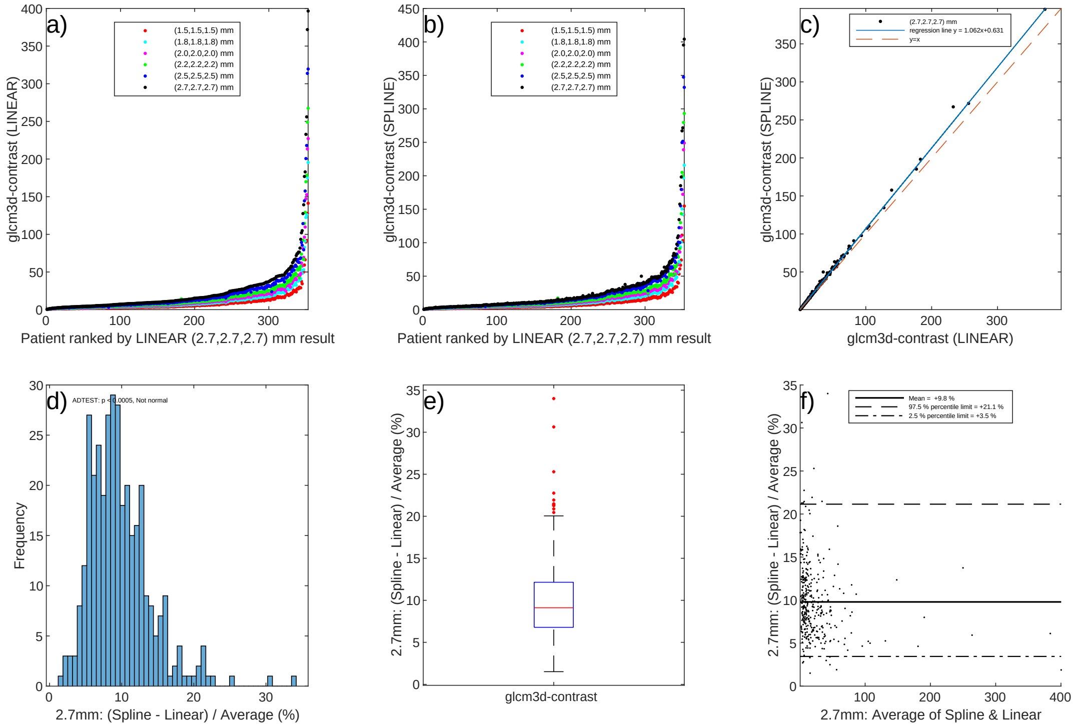

Figure 10: glcm3d-contrast

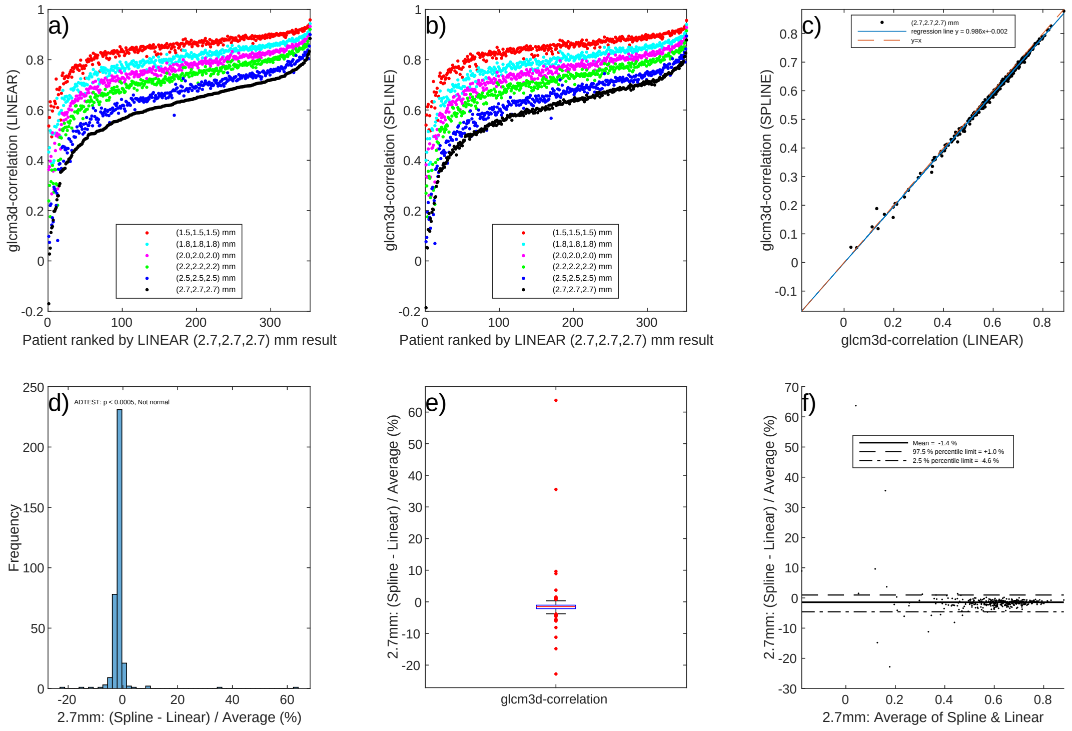

Figure 11: glcm3d-correlation

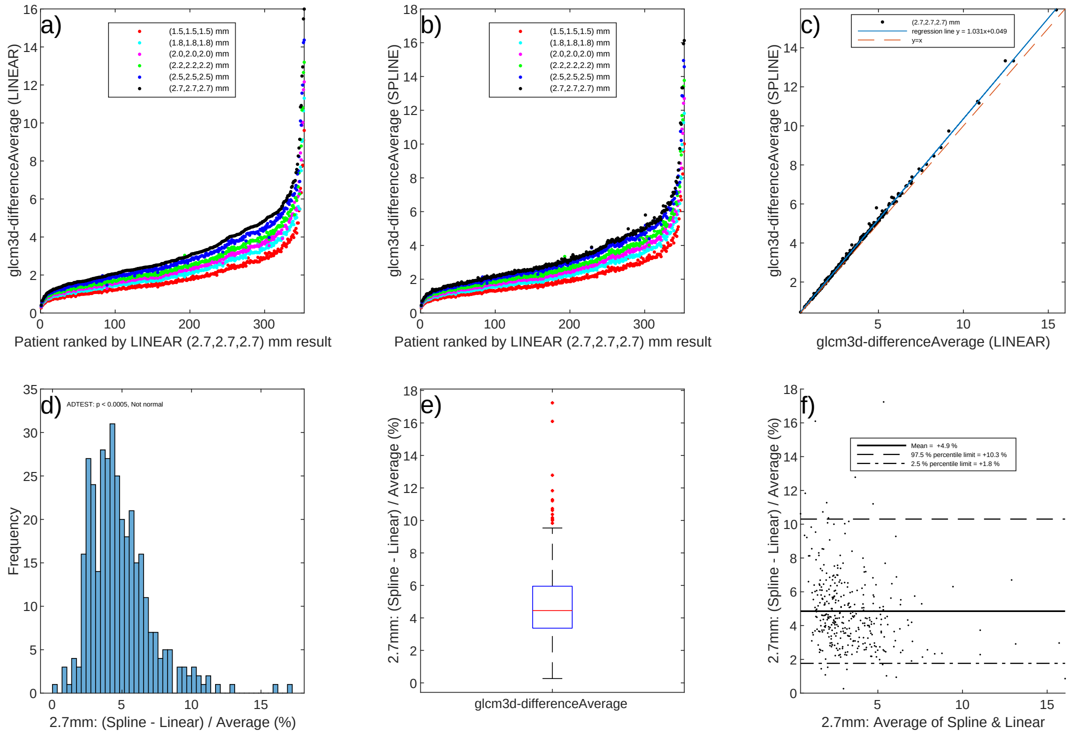

Figure 12: glcm3d-differenceAverage

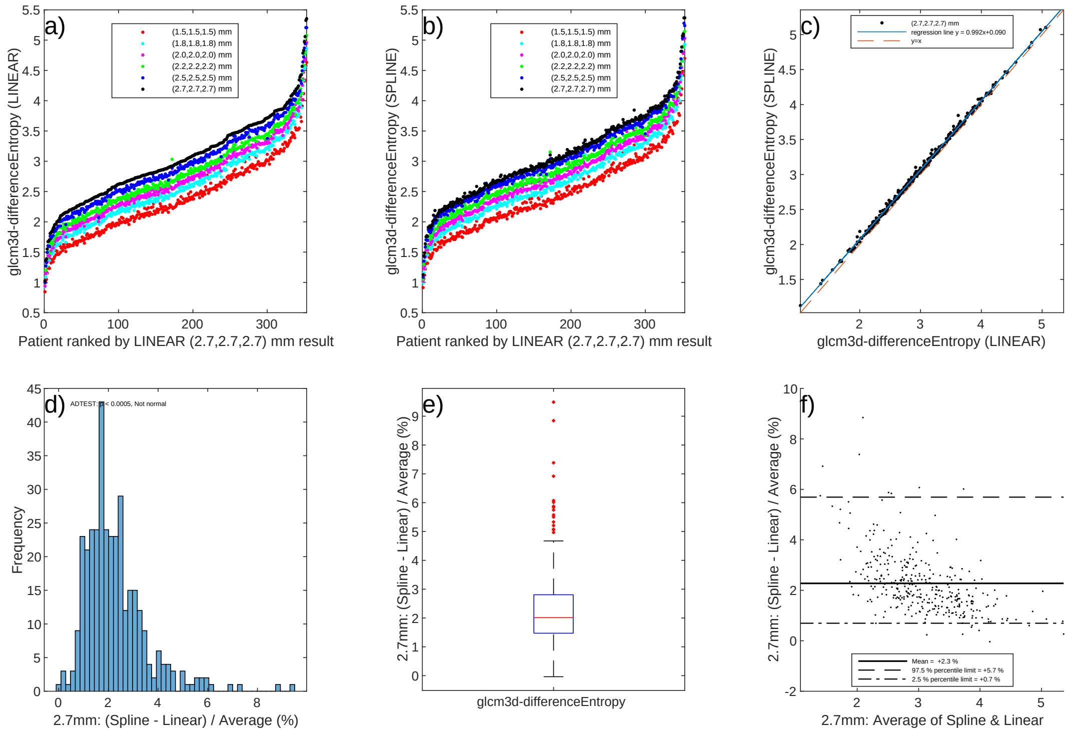

Figure 13: glcm3d-differenceEntropy

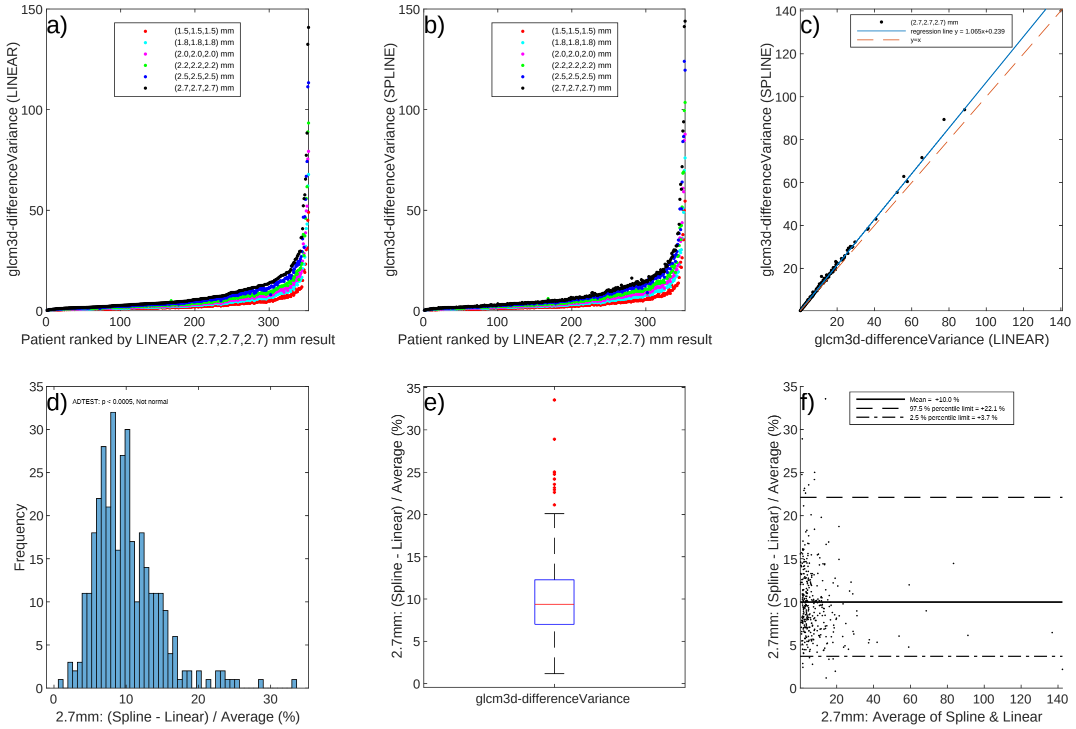

Figure 14: glcm3d-differenceVariance

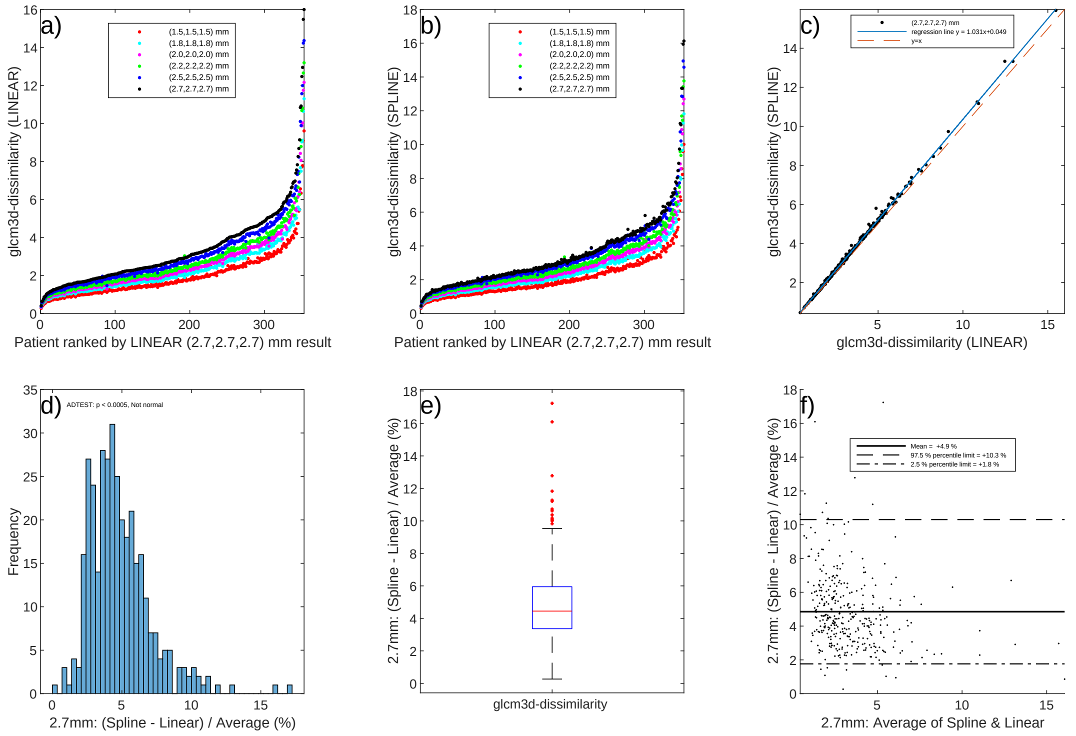

Figure 15: glcm3d-dissimilarity

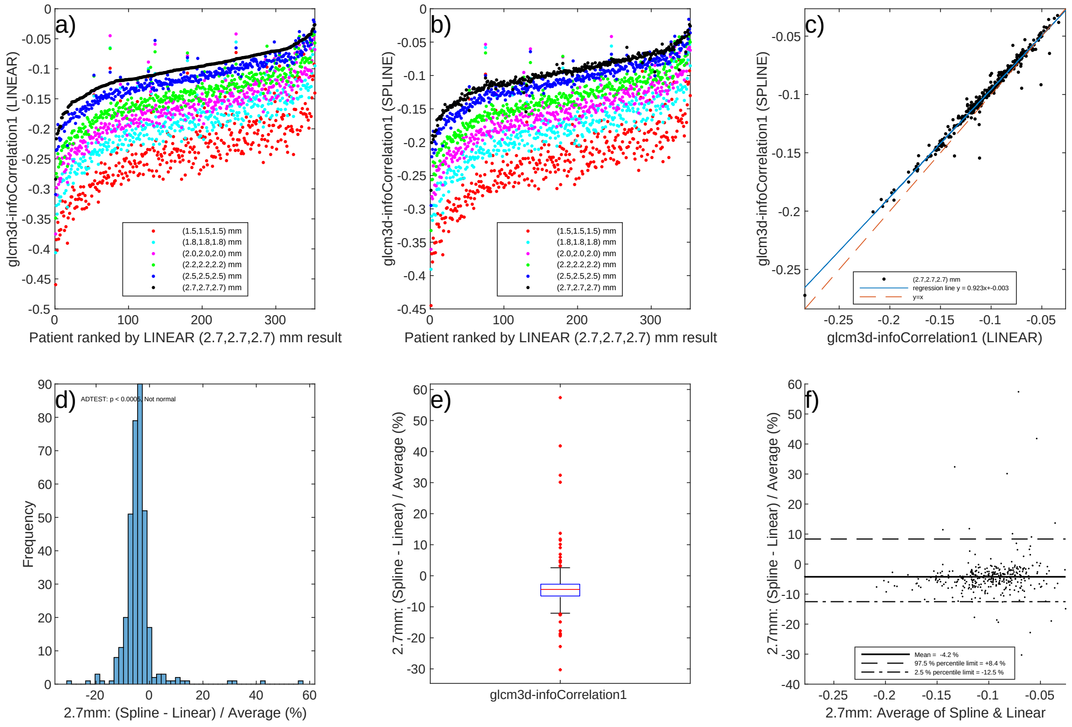

Figure 16: glcm3d-infoCorrelation1

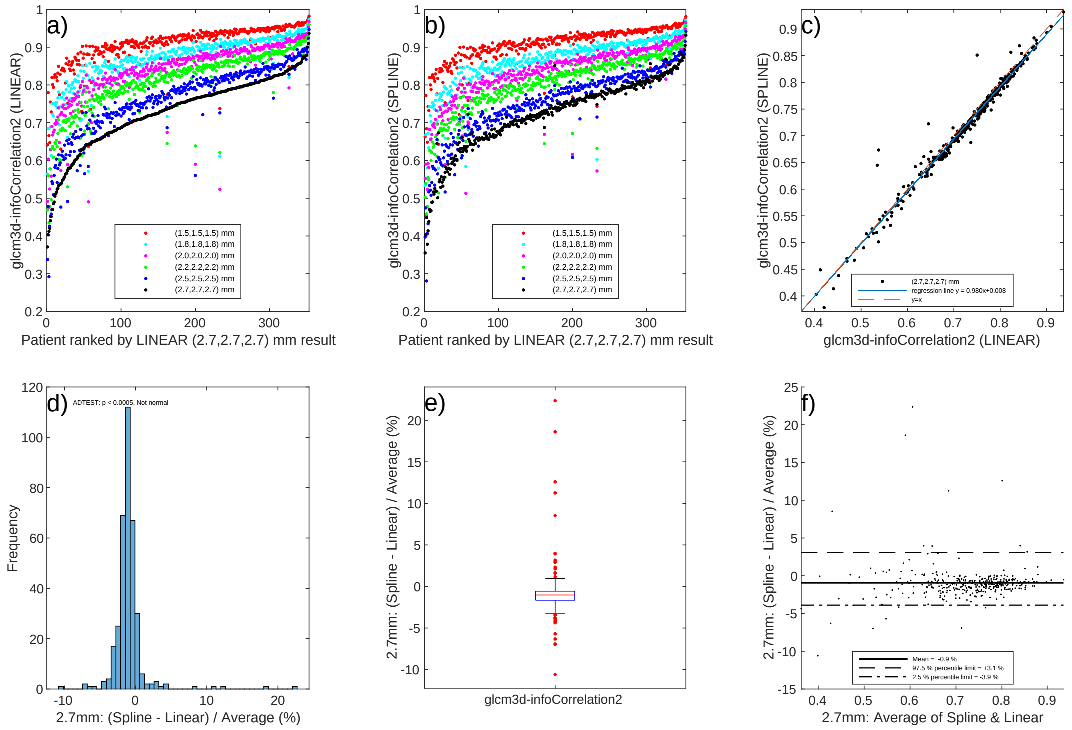

Figure 17: glcm3d-infoCorrelation2

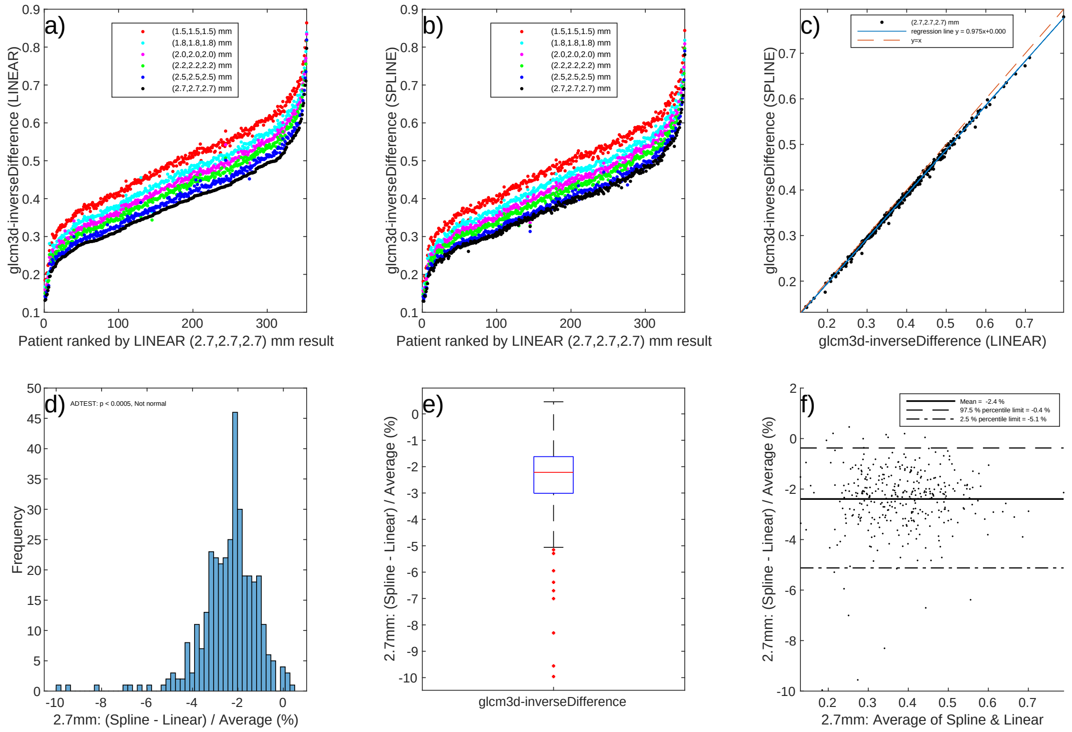

Figure 18: glcm3d-inverseDifference

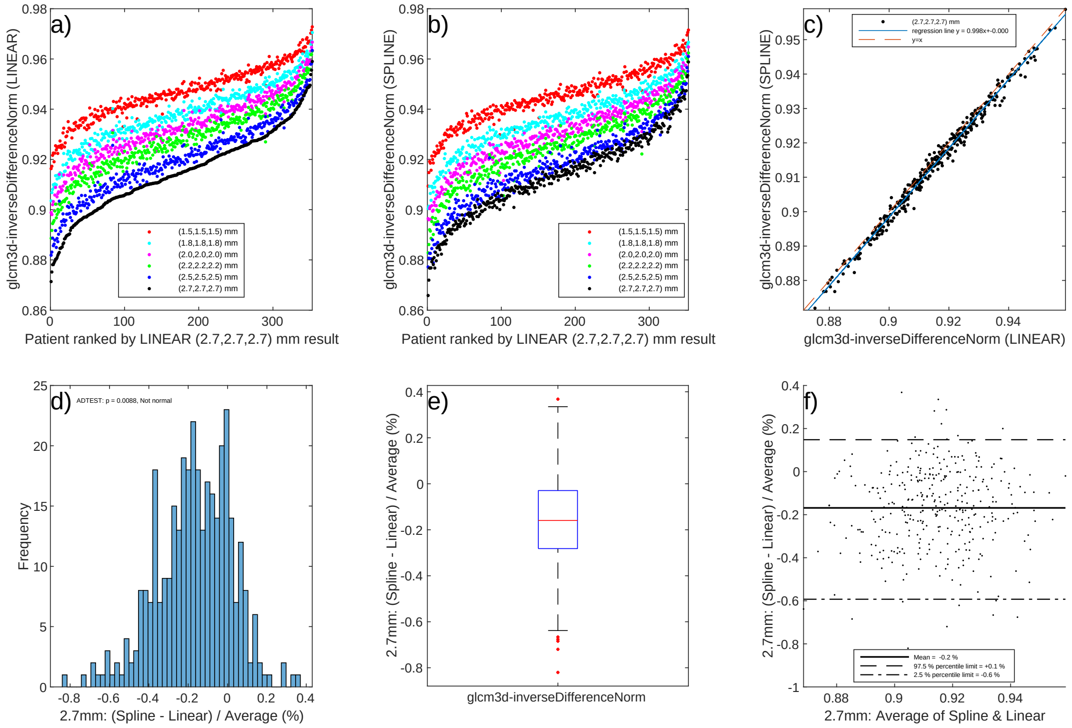

Figure 19: glcm3d-inverseDifferenceNorm

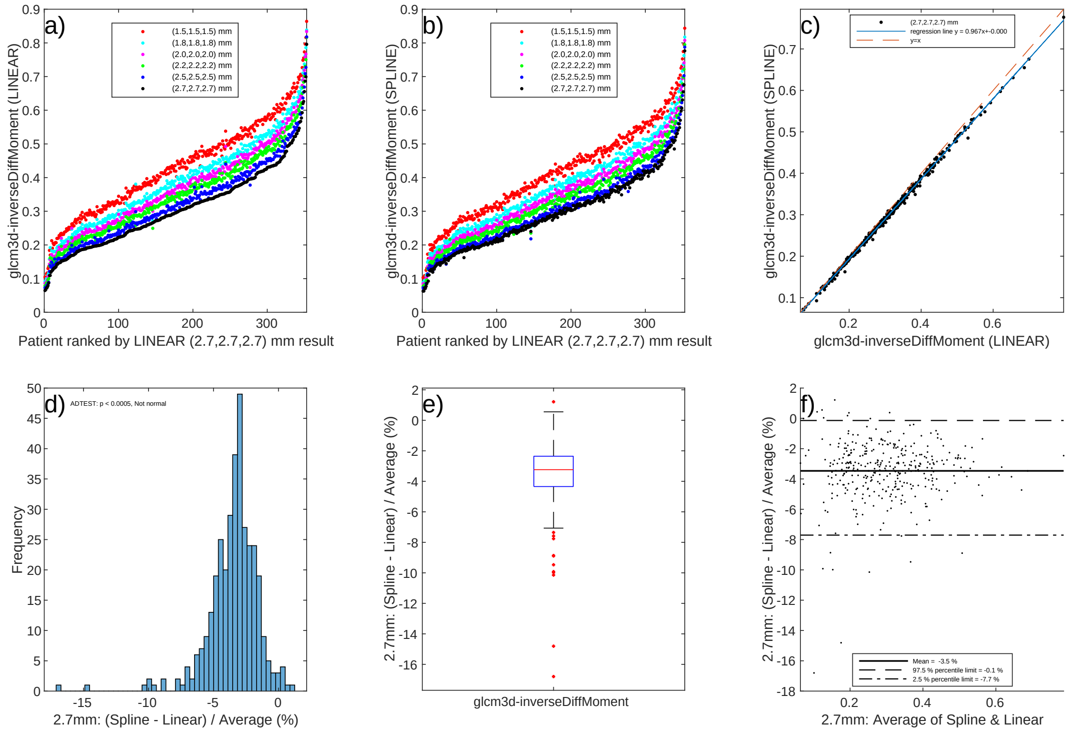

Figure 20: glcm3d-inverseDiffMoment

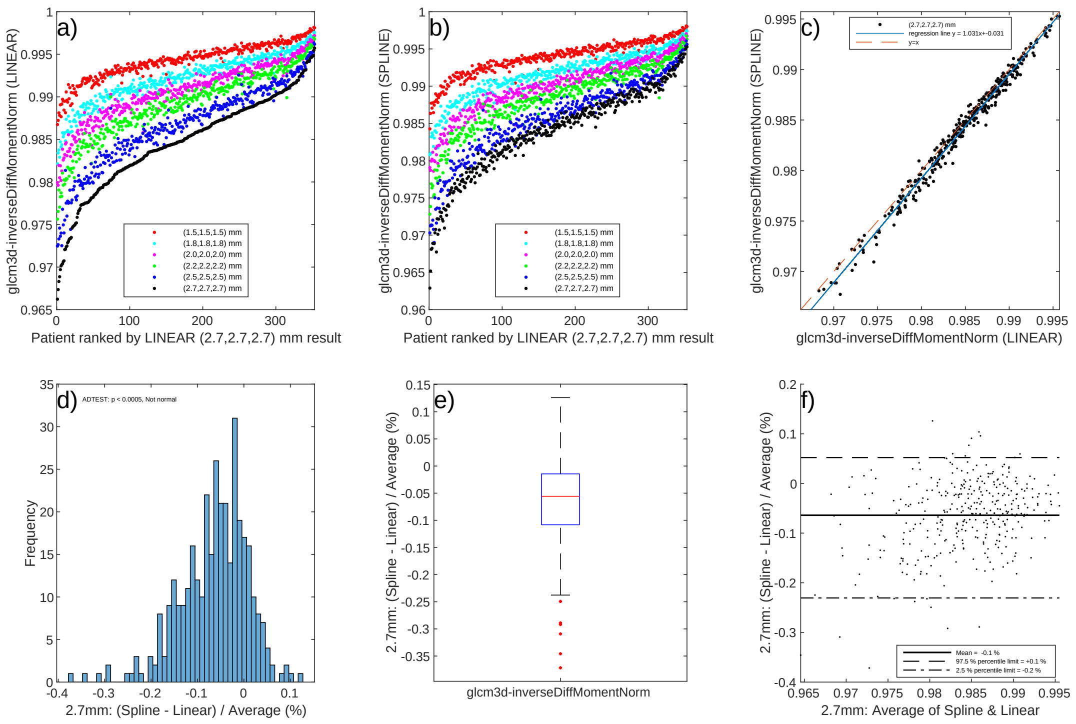

Figure 21: glcm3d-inverseDiffMomentNorm

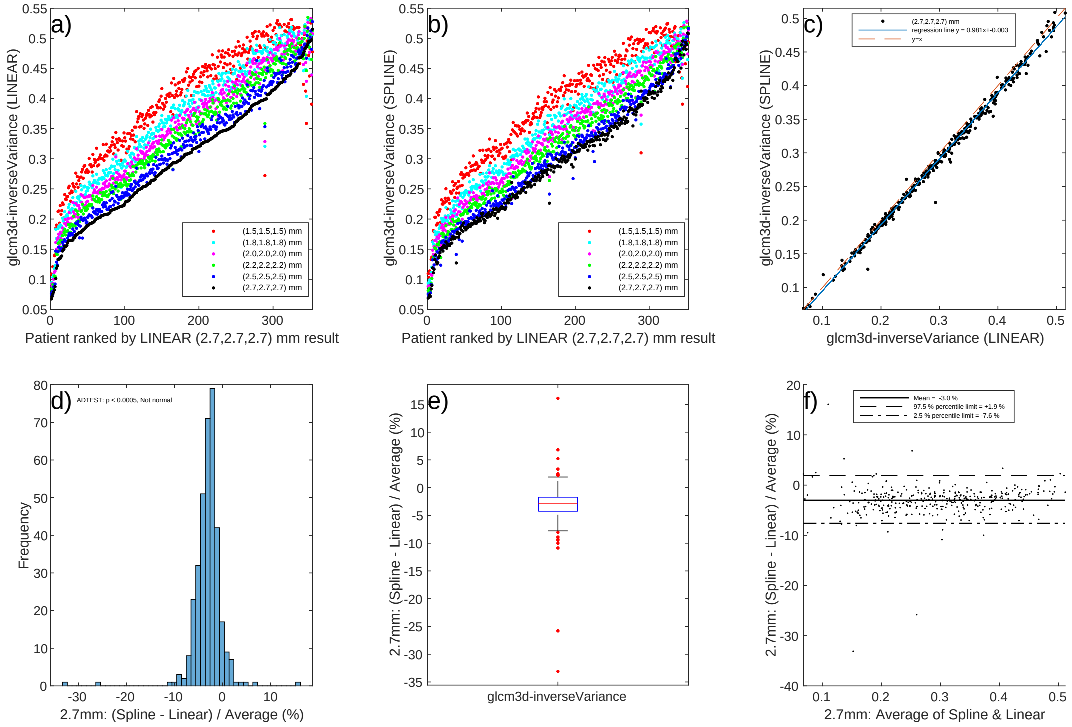

Figure 22: glcm3d-inverseVariance

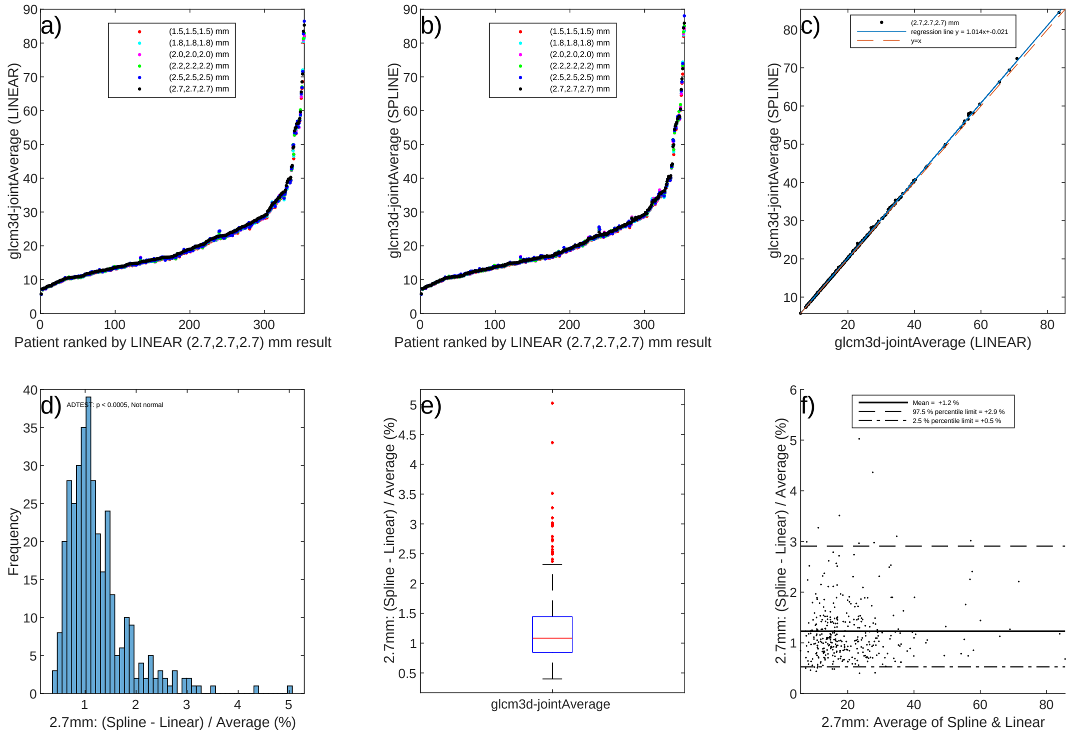

Figure 23: glcm3d-jointAverage

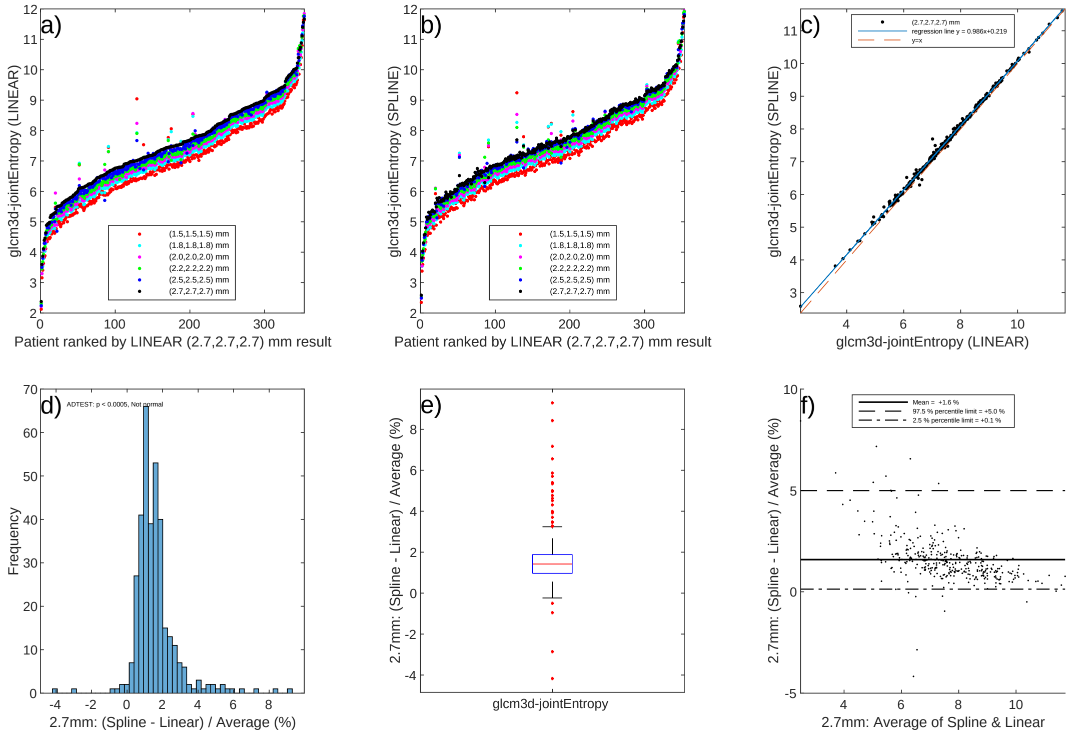

Figure 24: glcm3d-jointEntropy

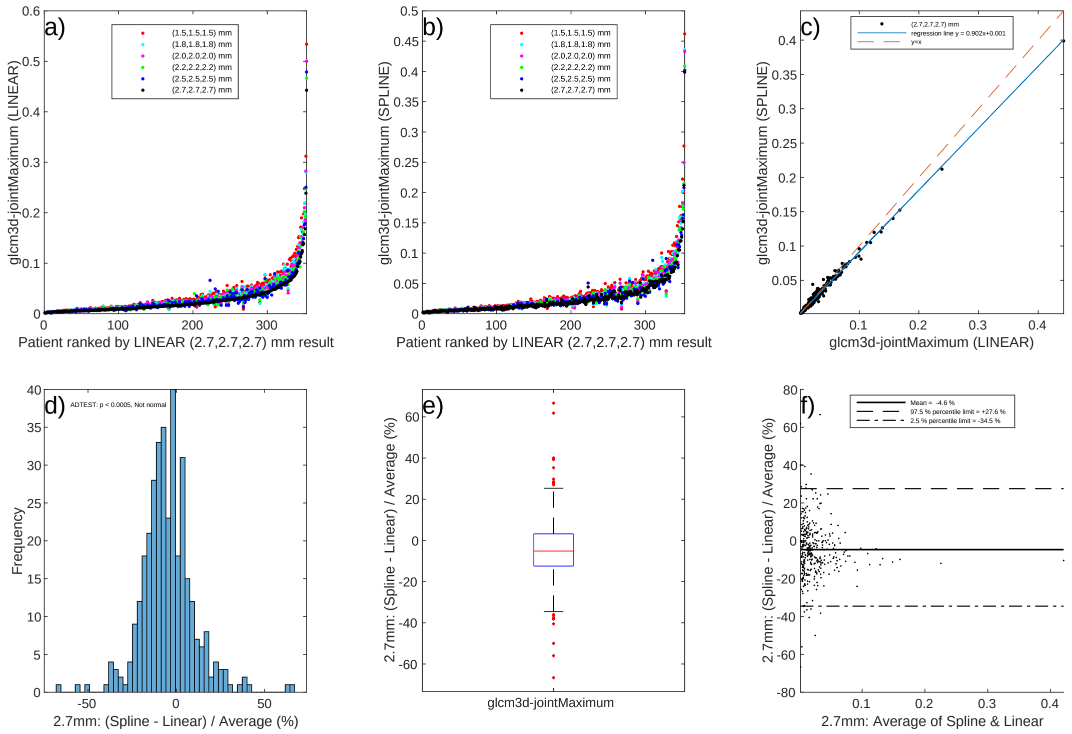

Figure 25: glcm3d-jointMaximum

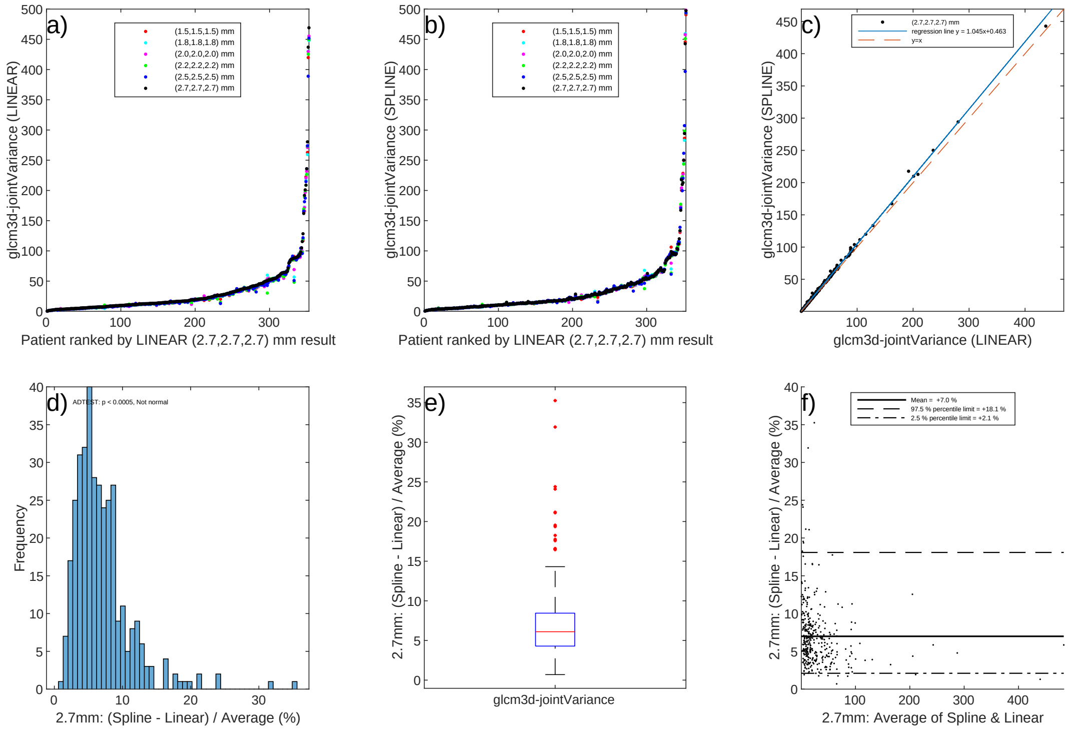

Figure 26: glcm3d-jointVariance

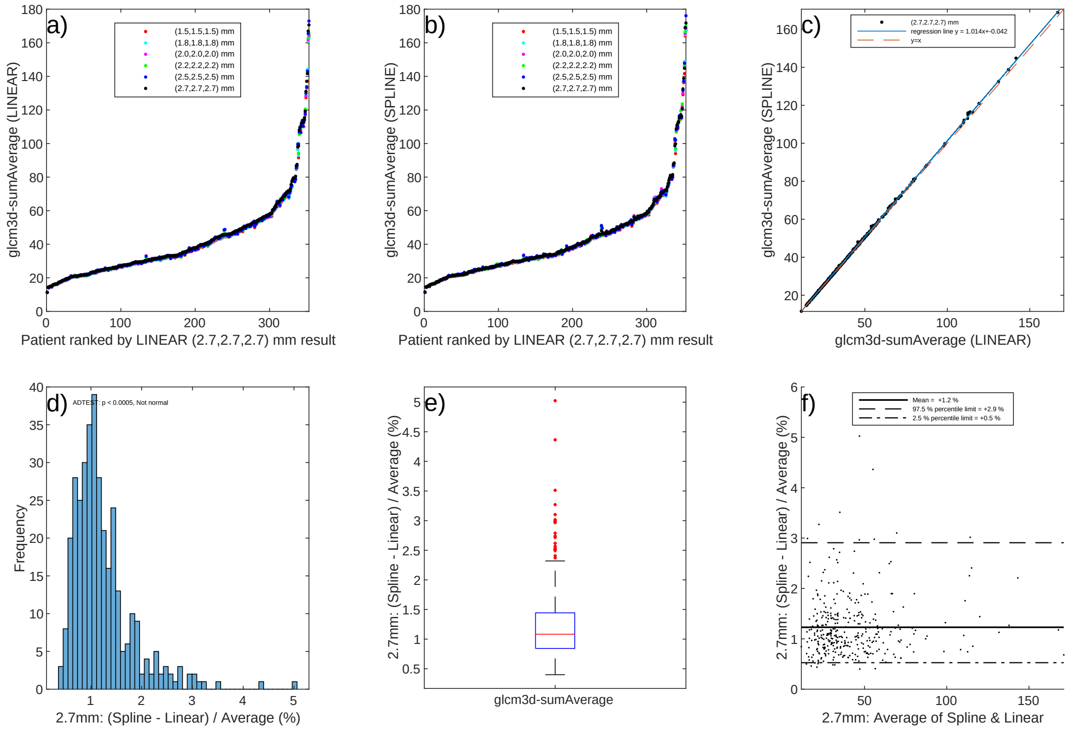

Figure 27: glcm3d-sumAverage

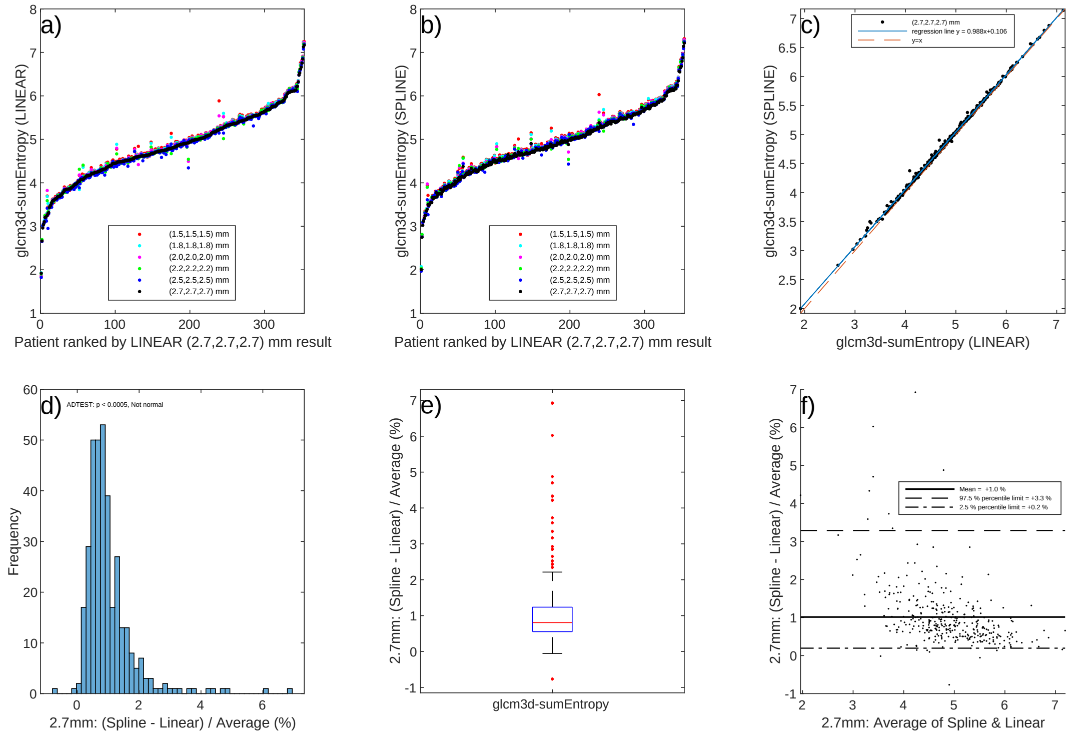

Figure 28: glcm3d-sumEntropy

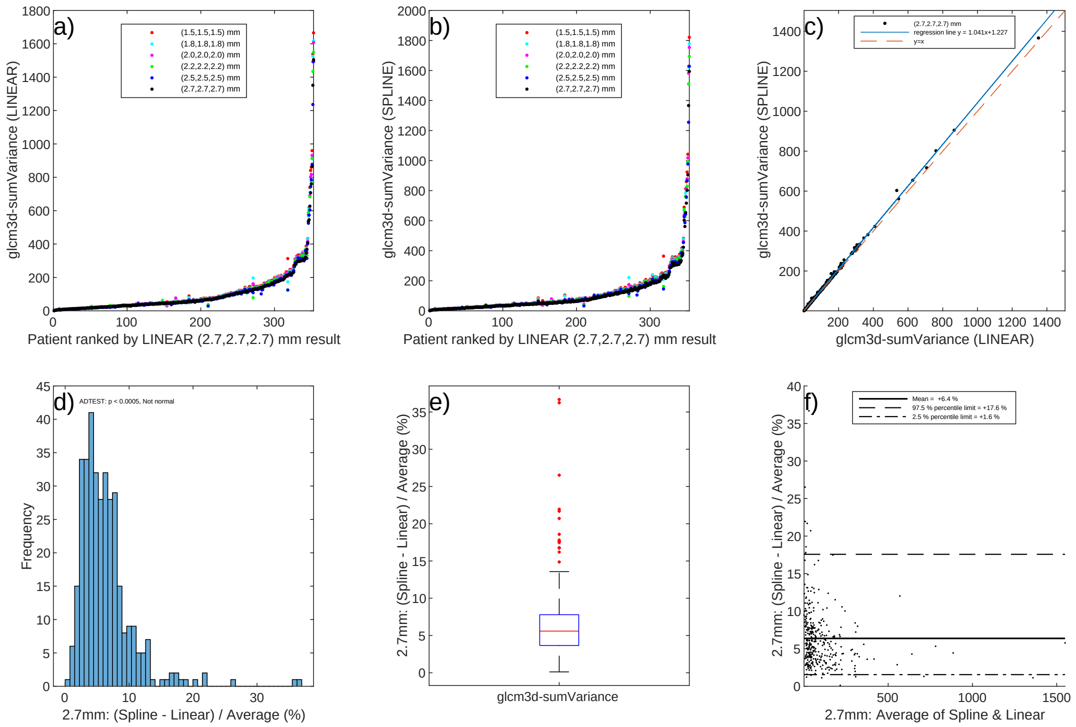

Figure 29: glcm3d-sumVariance

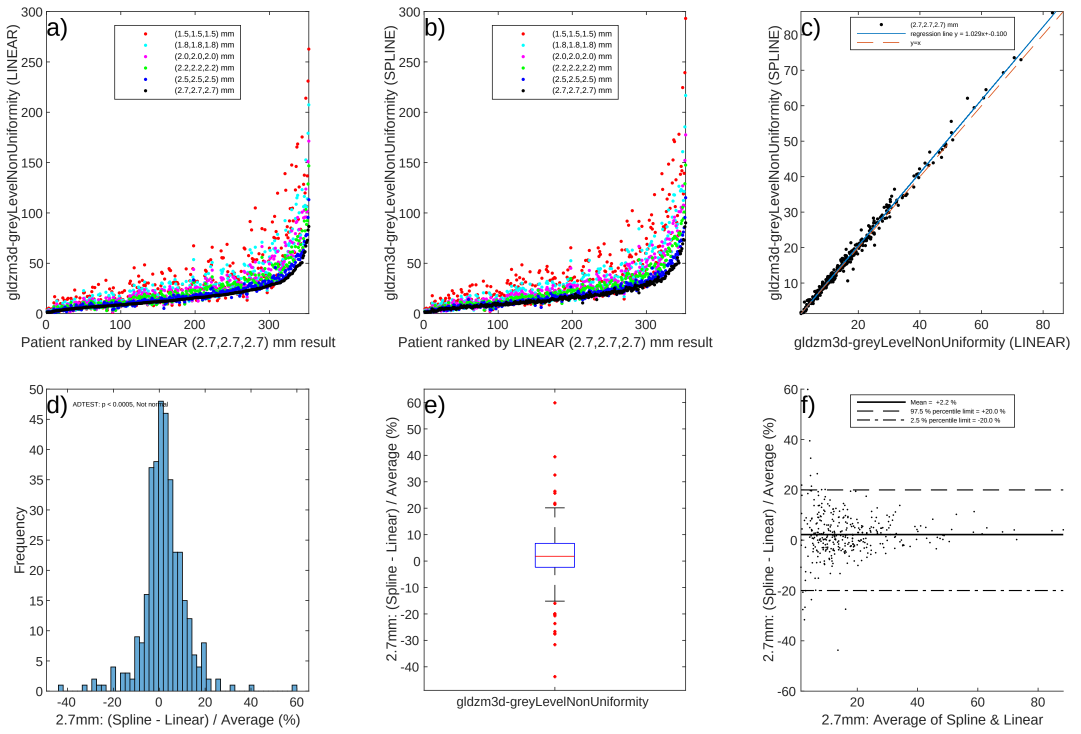

Figure 30: gldzm3d-greyLevelNonUniformity

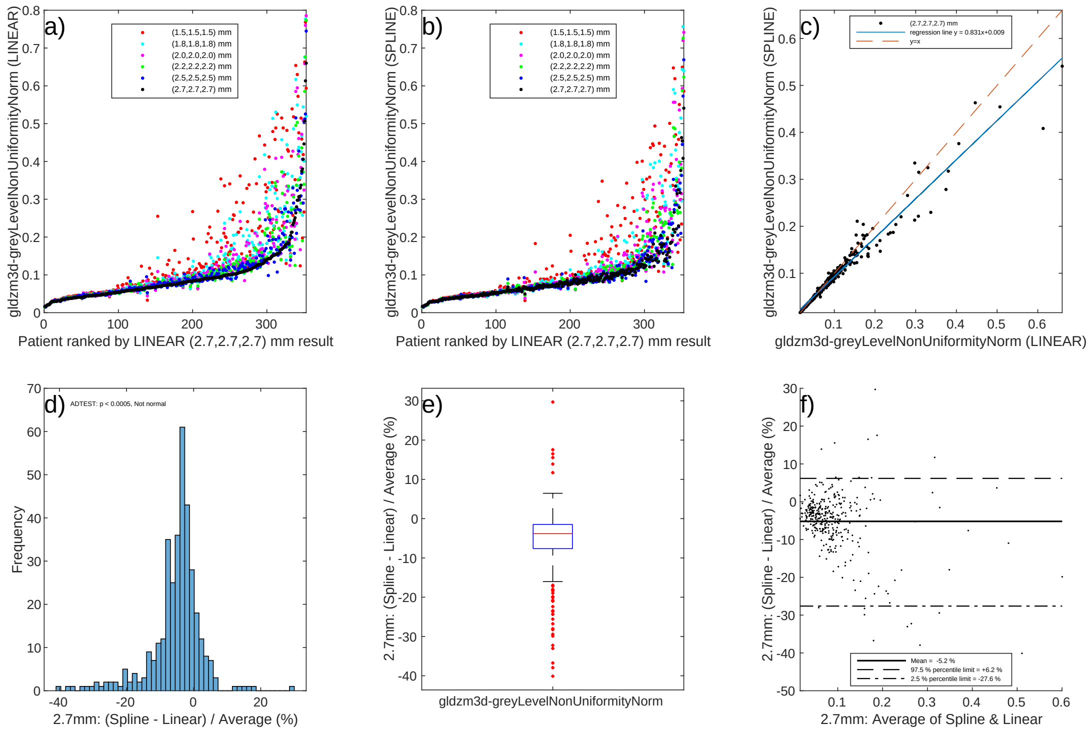

Figure 31: gldzm3d-greyLevelNonUniformityNorm

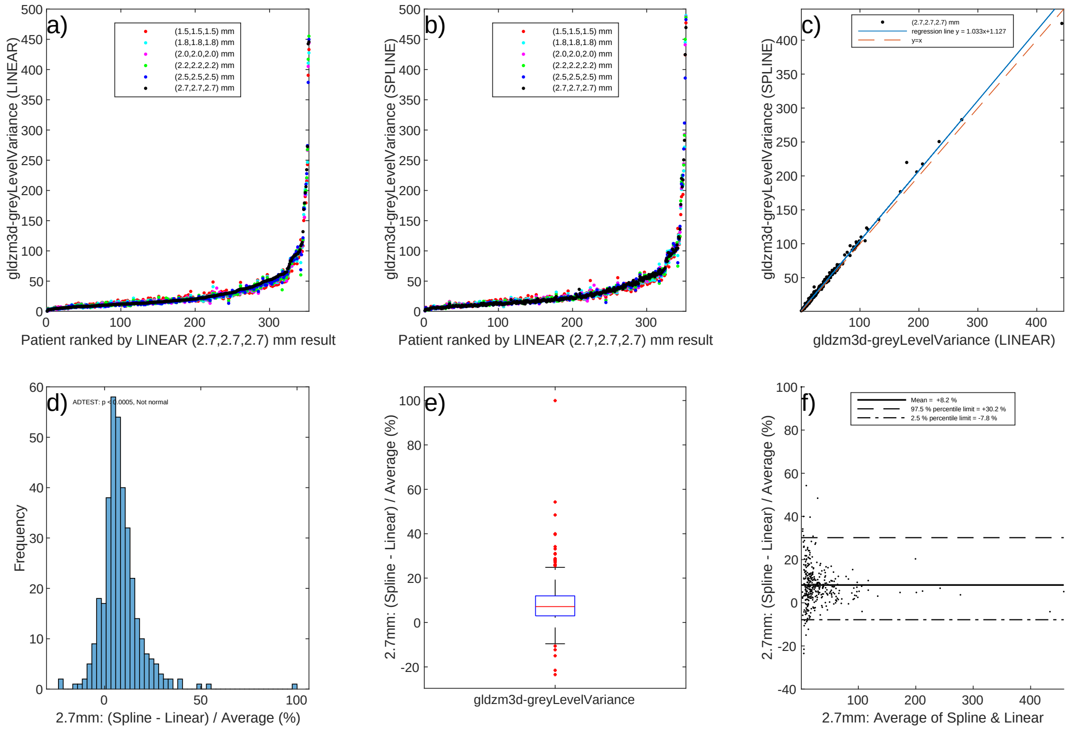

Figure 32: gldzm3d-greyLevelVariance

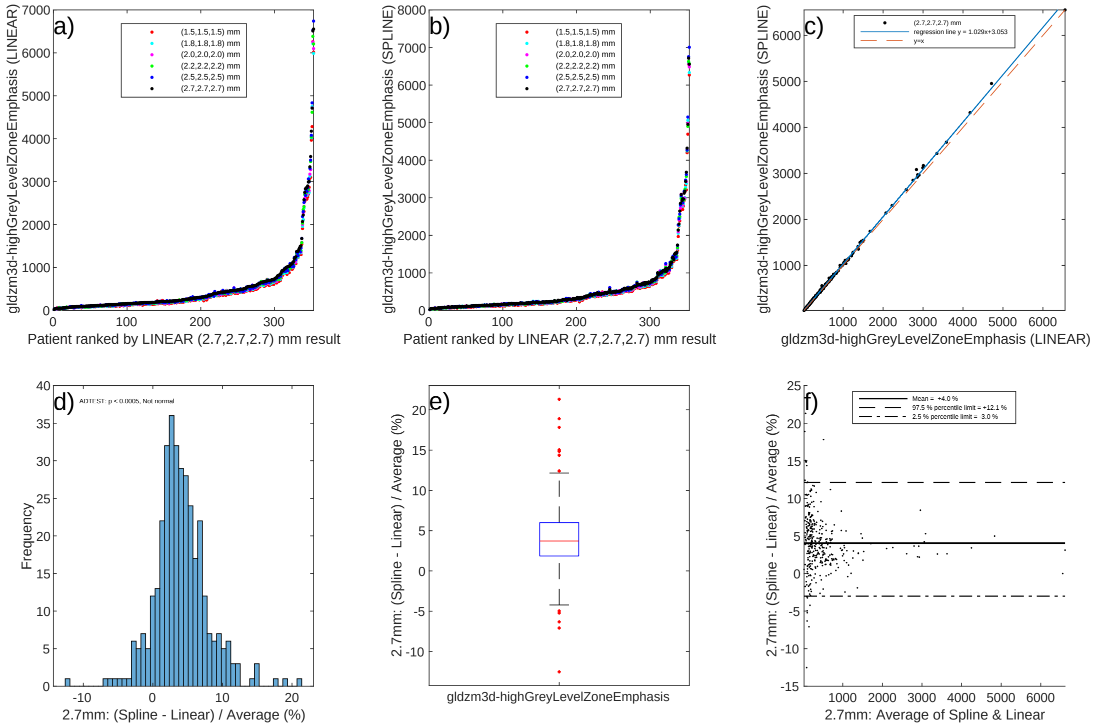

Figure 33: gldzm3d-highGreyLevelZoneEmphasis

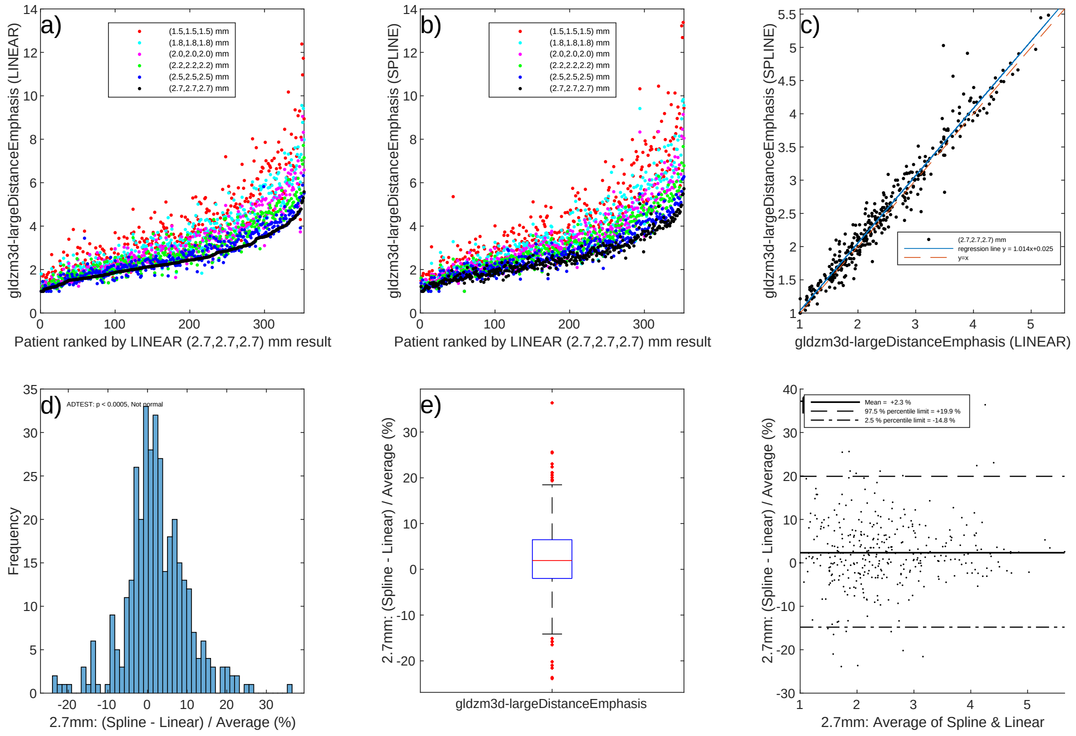

Figure 34: gldzm3d-largeDistanceEmphasis

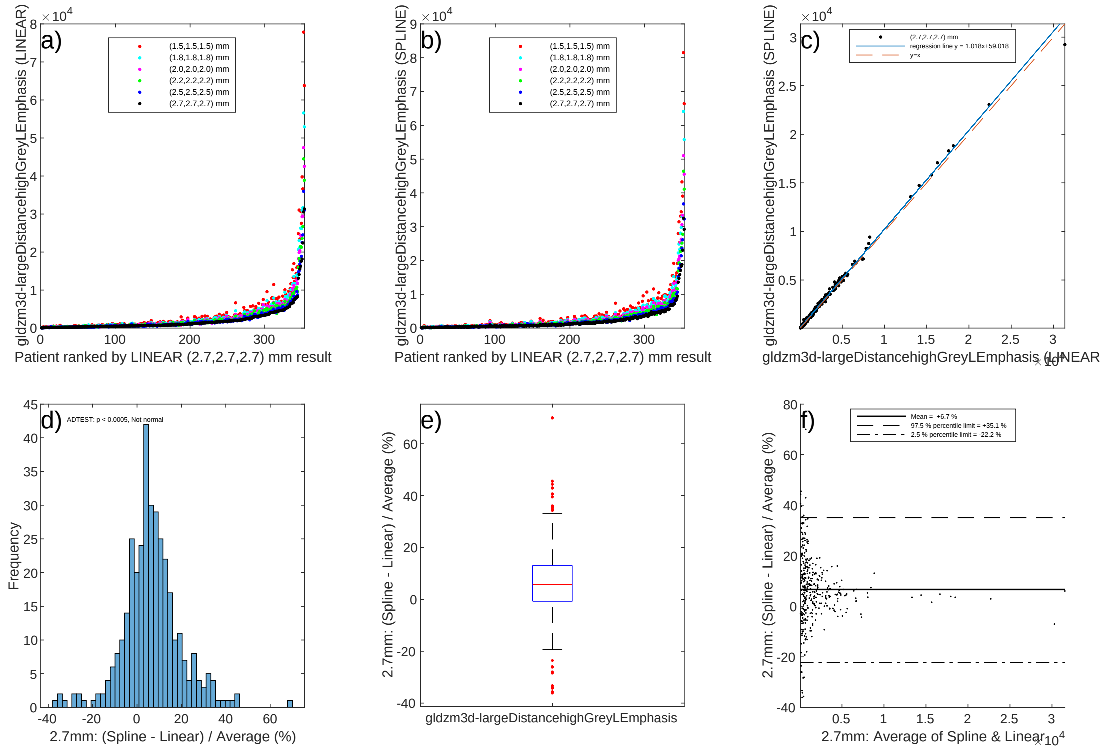

Figure 35: gldzm3d-largeDistancehighGreyLEmphasis

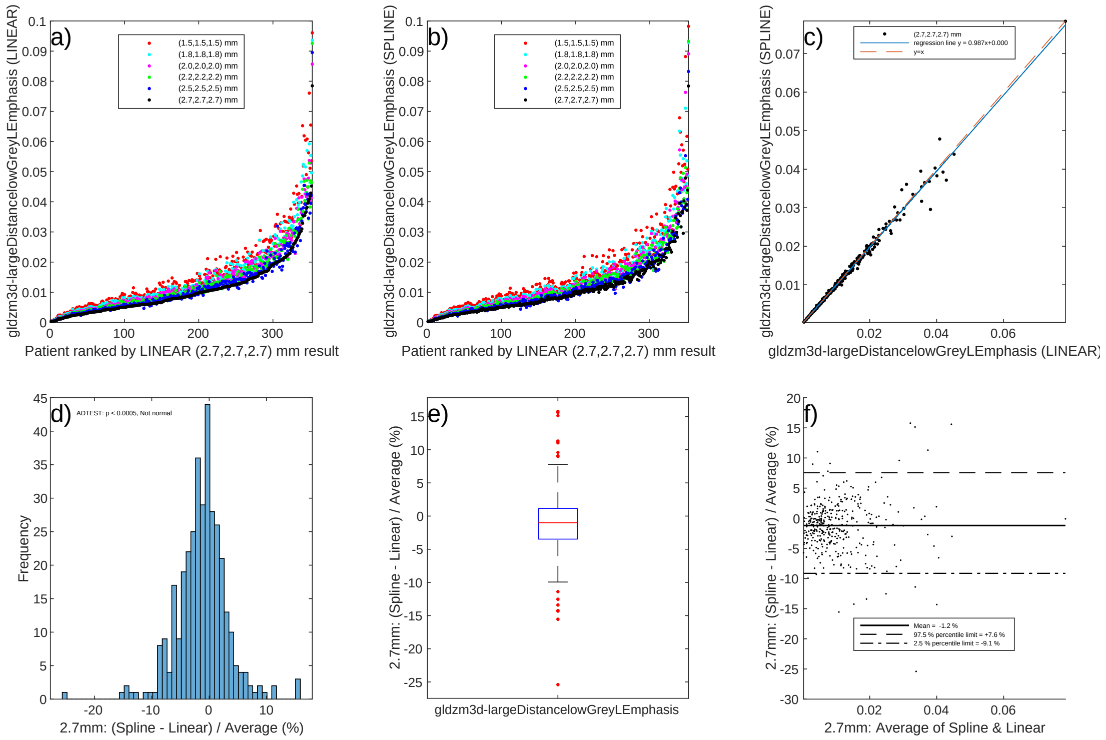

Figure 36: gldzm3d-largeDistancelowGreyLEmphasis

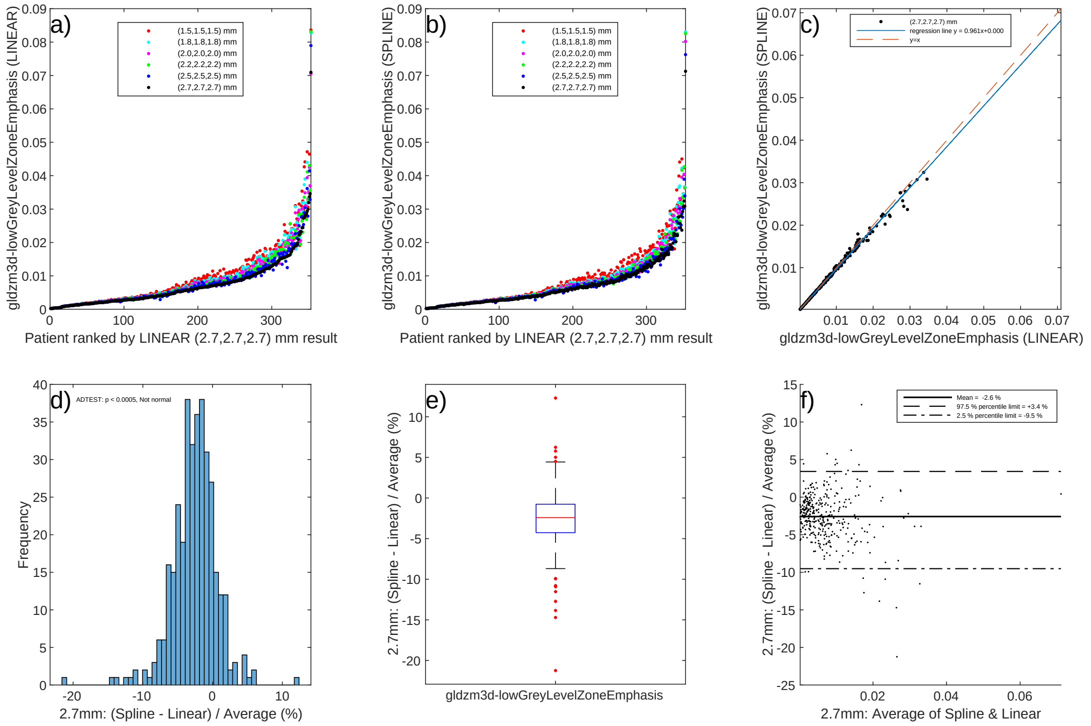

Figure 37: gldzm3d-lowGreyLevelZoneEmphasis

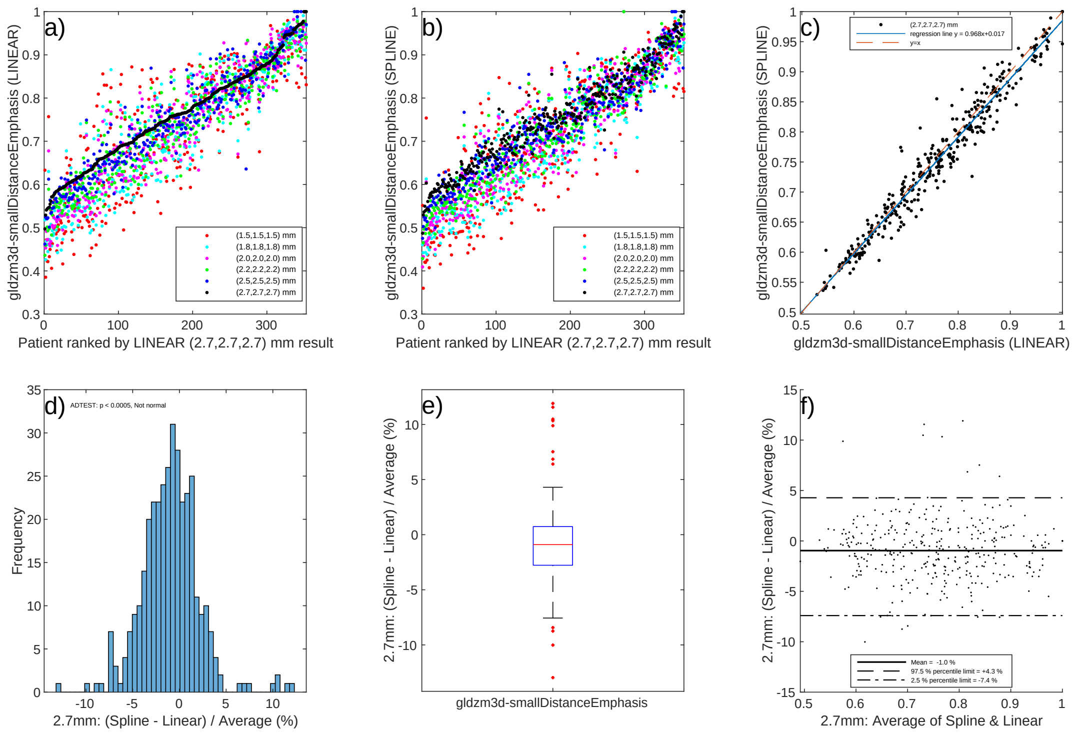

Figure 38: gldzm3d-smallDistanceEmphasis

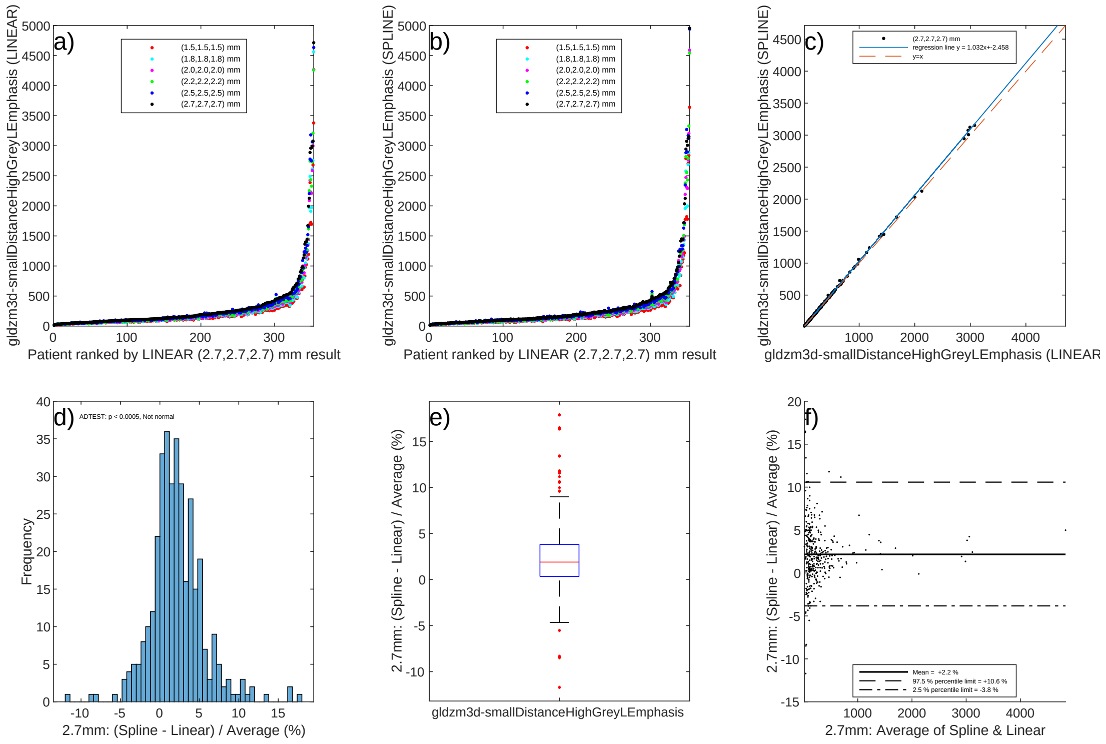

Figure 39: gldzm3d-smallDistanceHighGreyLEmphasis

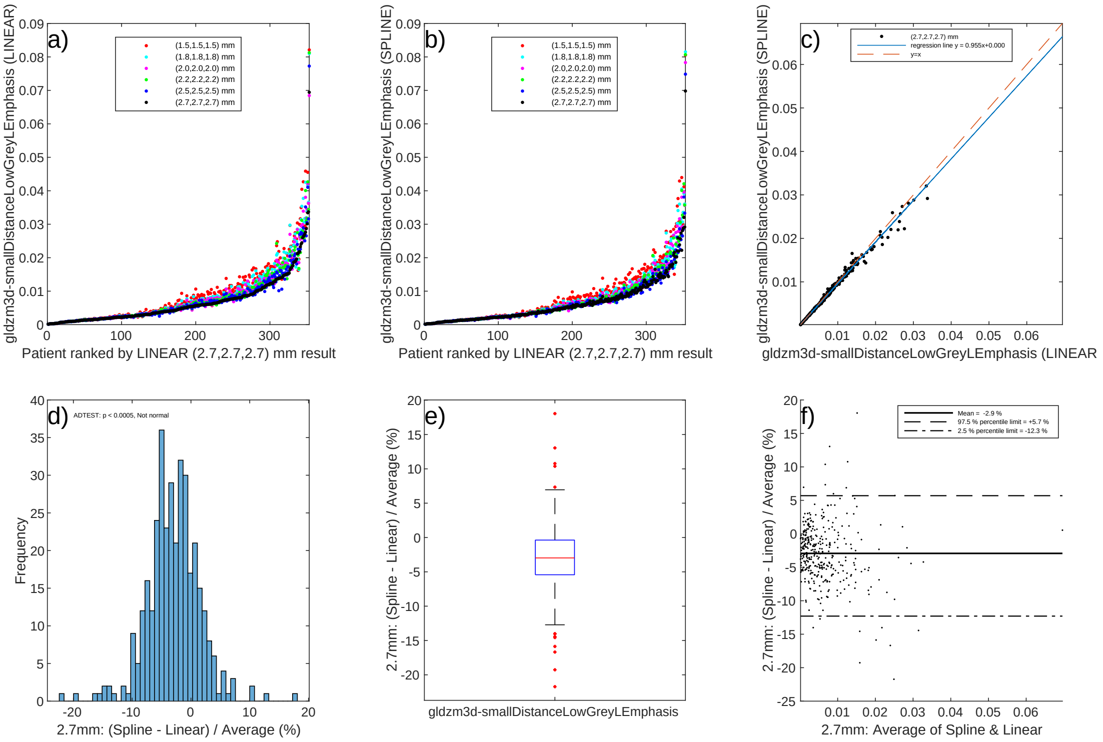

Figure 40: gldzm3d-smallDistanceLowGreyLEmphasis

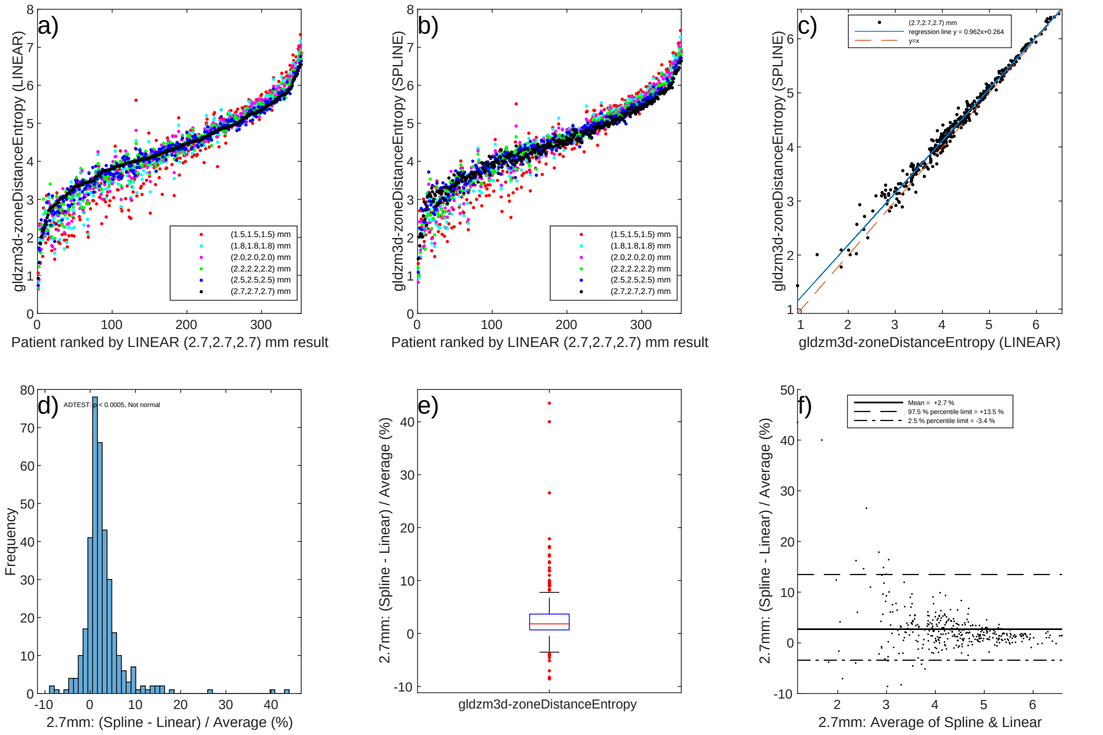

Figure 41: gldzm3d-zoneDistanceEntropy

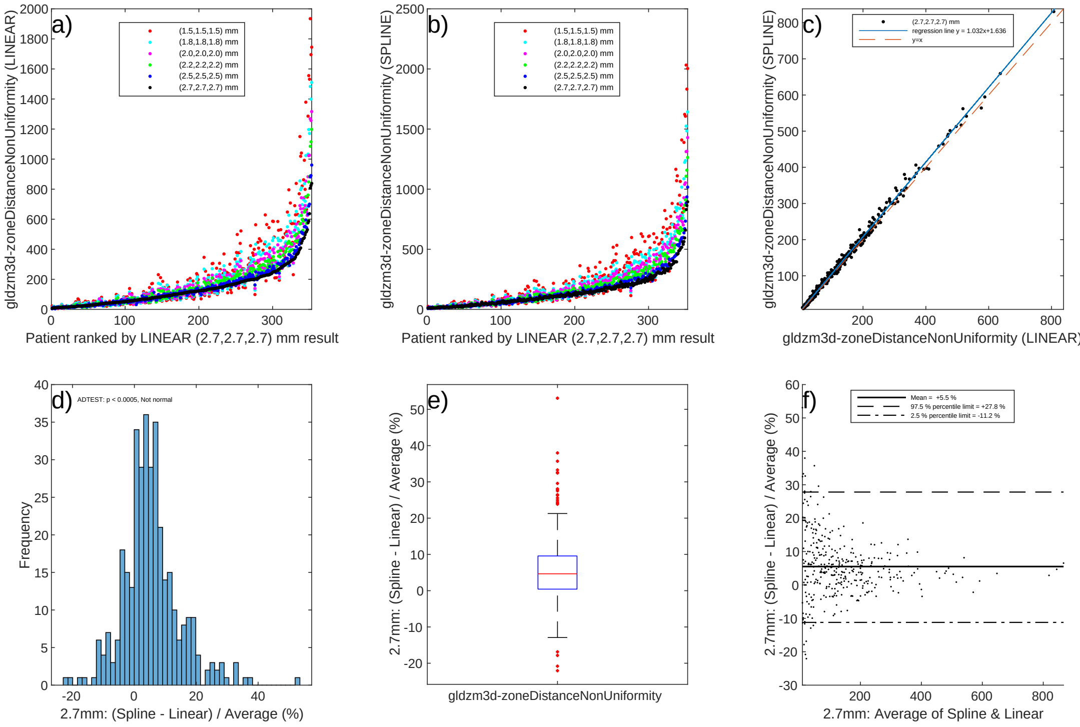

Figure 42: gldzm3d-zoneDistanceNonUniformity

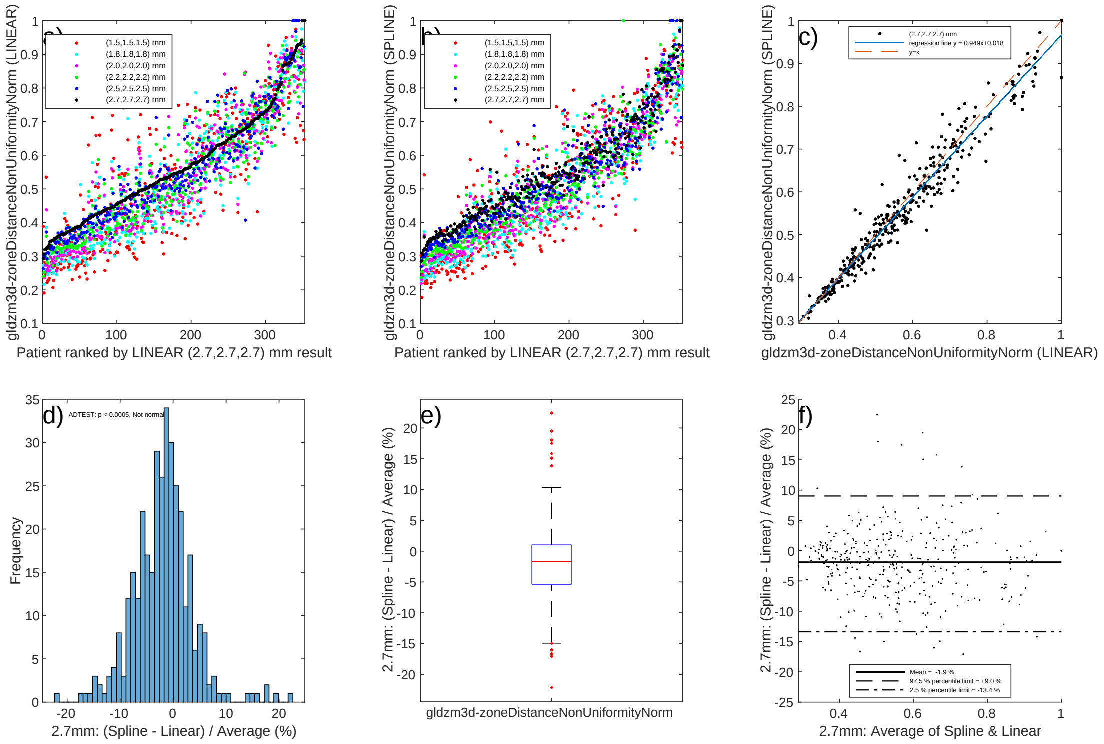

Figure 43: gldzm3d-zoneDistanceNonUniformityNorm

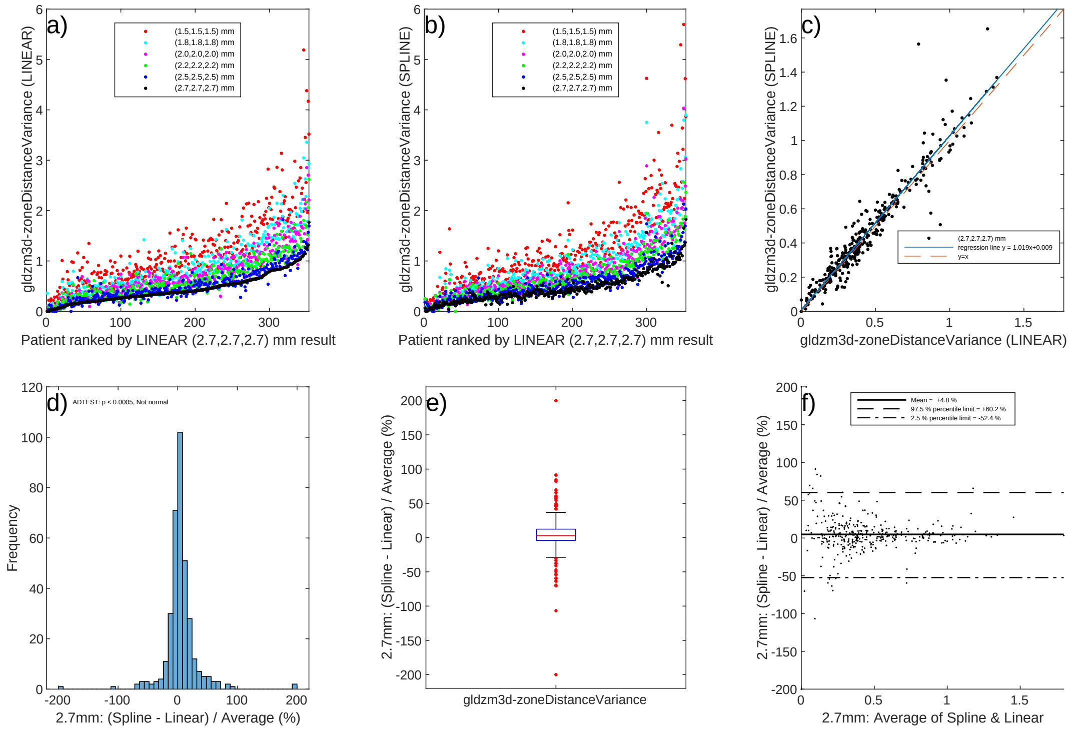

Figure 44: gldzm3d-zoneDistanceVariance

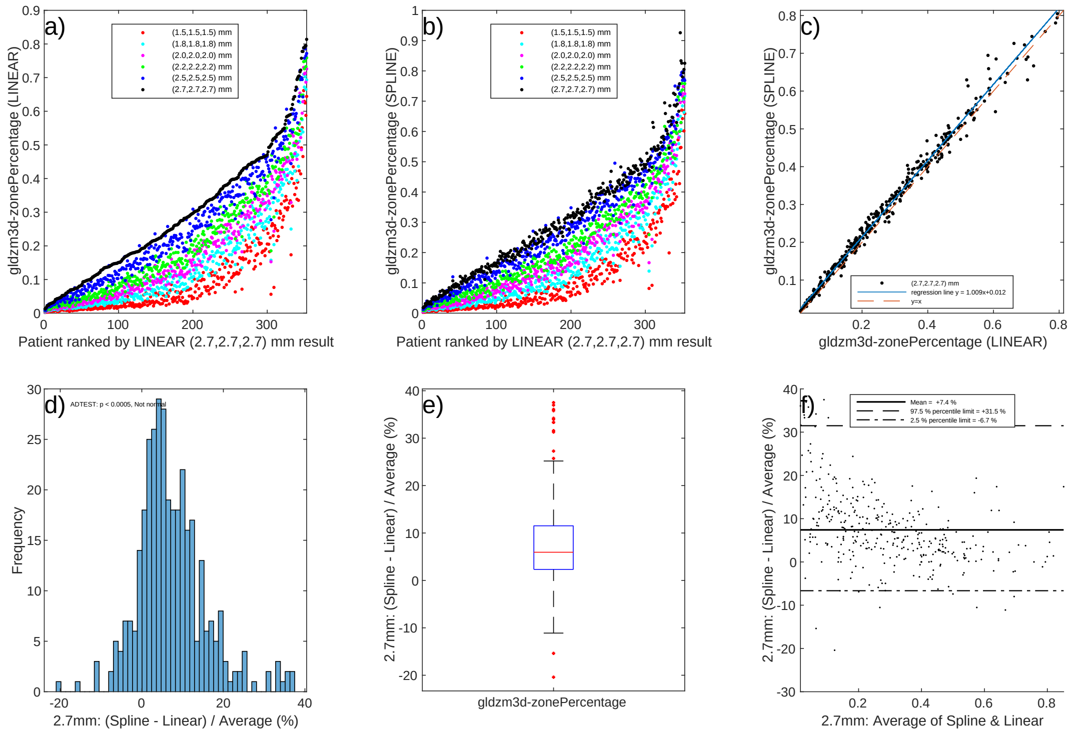

Figure 45: gldzm3d-zonePercentage

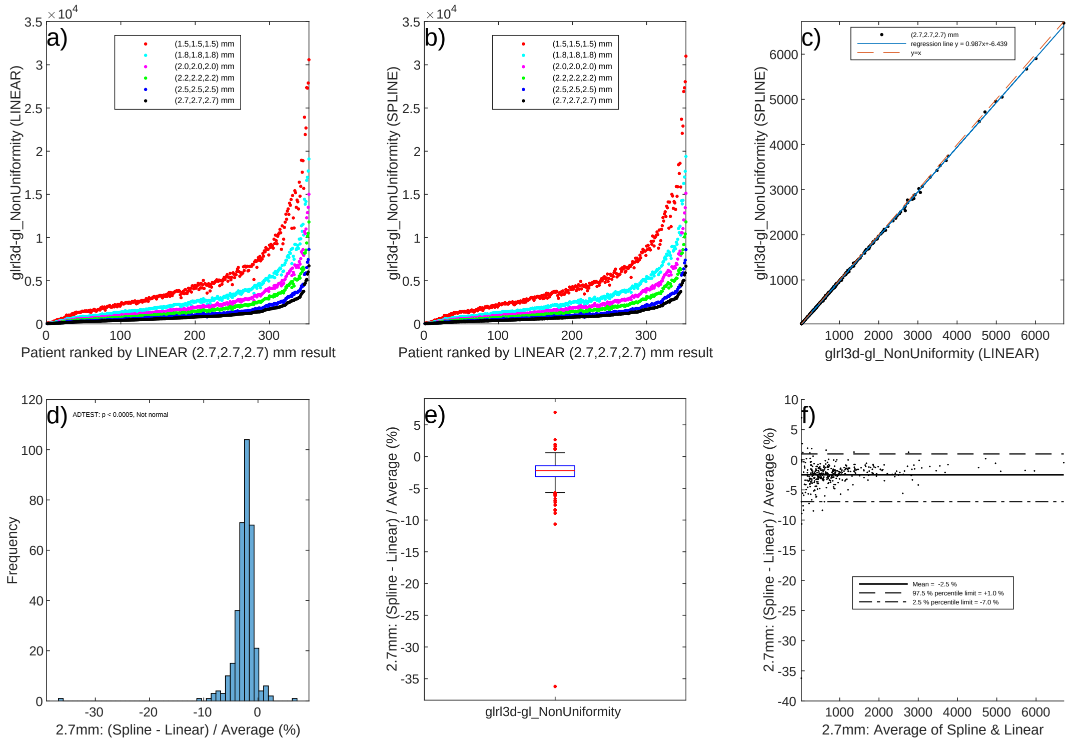

Figure 46: glrl3d-gl-NonUniformity

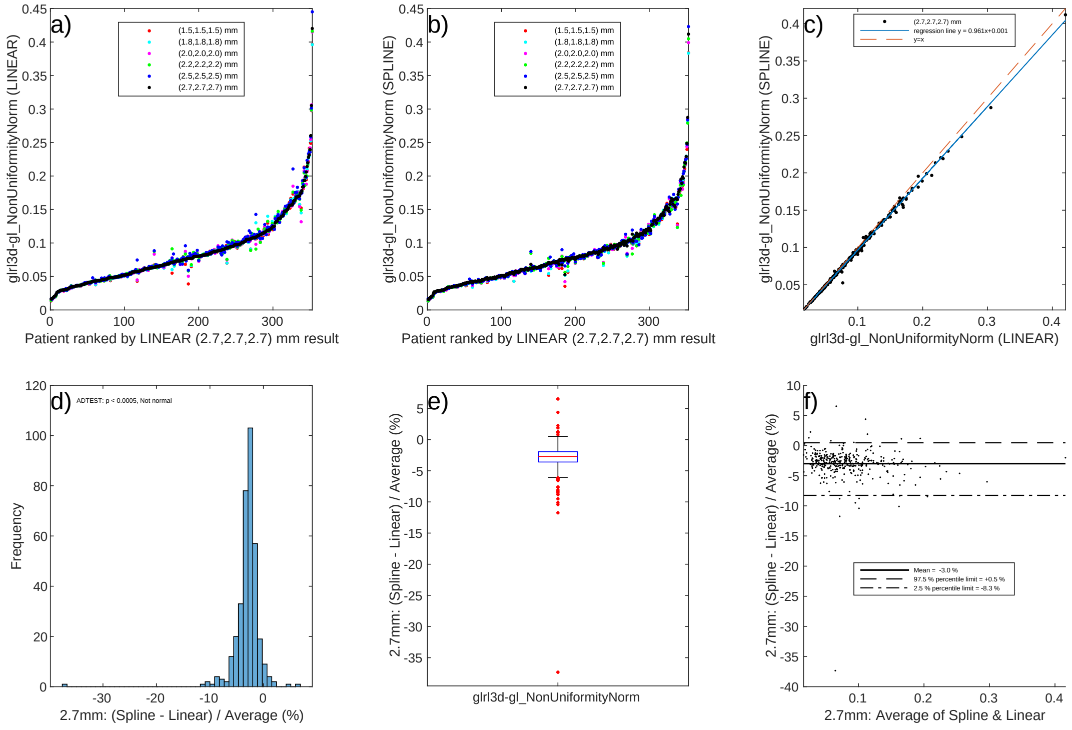

Figure 47: glrl3d-gl-NonUniformityNorm

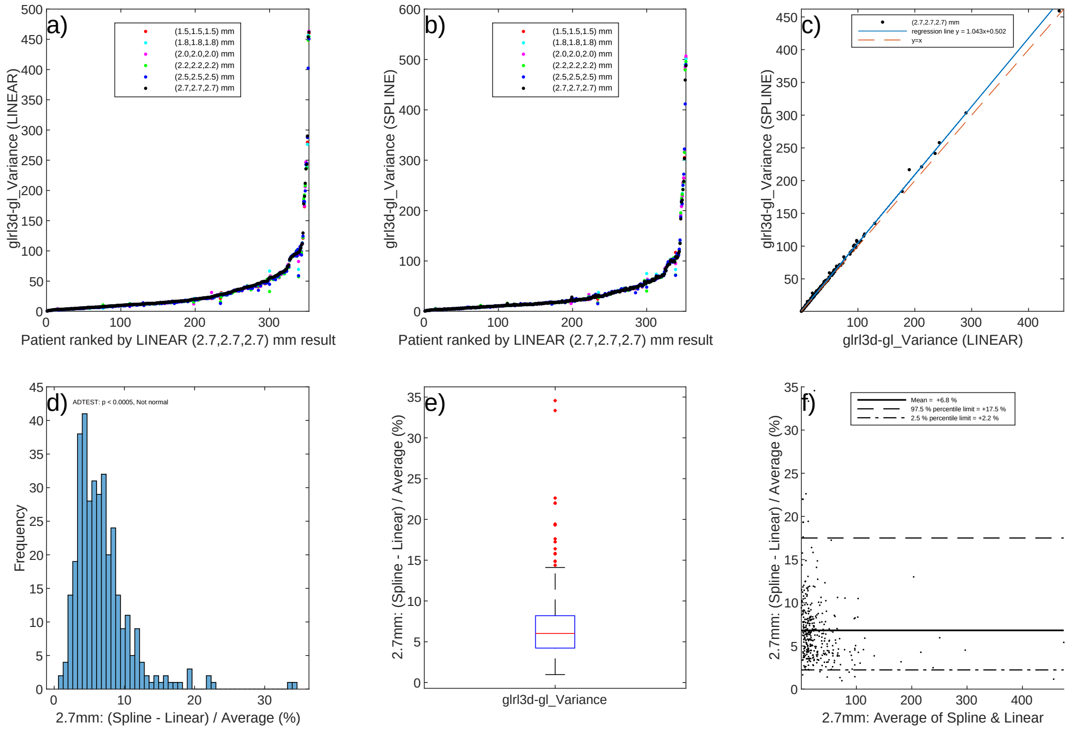

Figure 48: glrl3d-gl-Variance

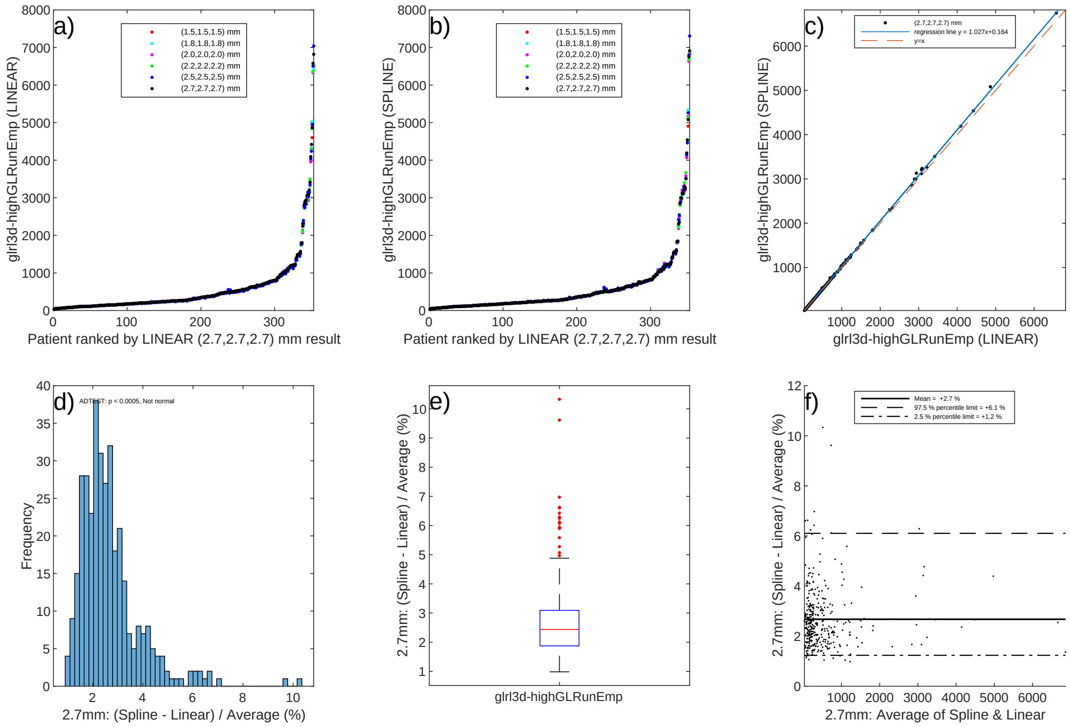

Figure 49: glrl3d-highGLRunEmp

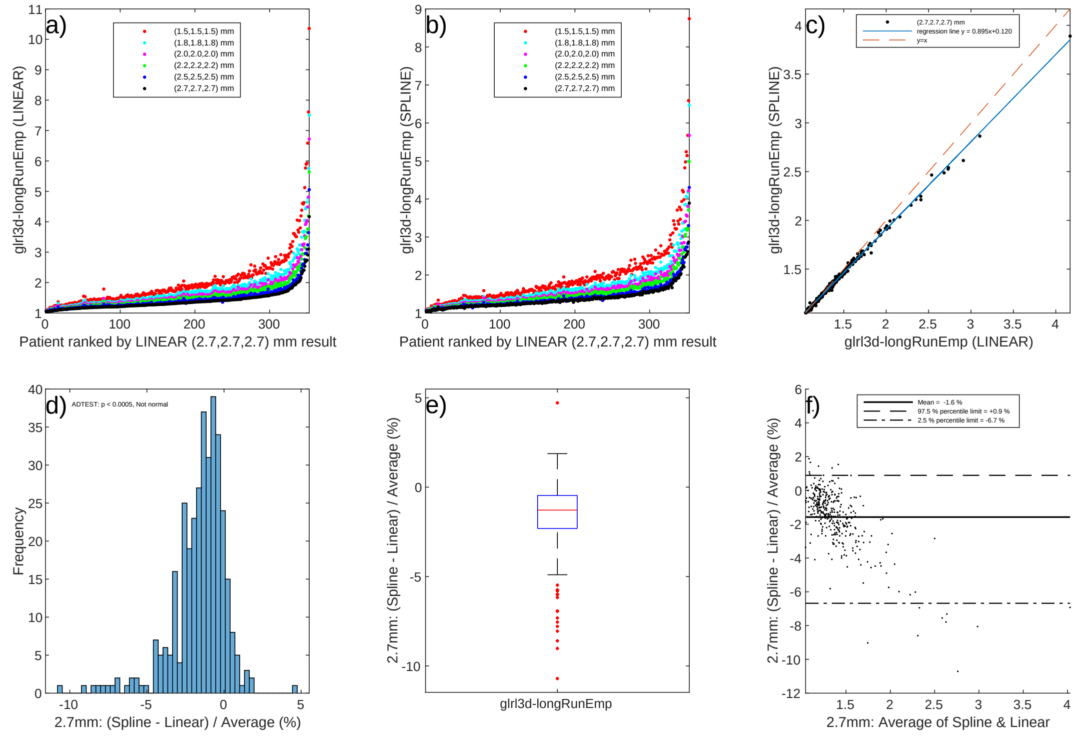

Figure 50: glrl3d-longRunEmp

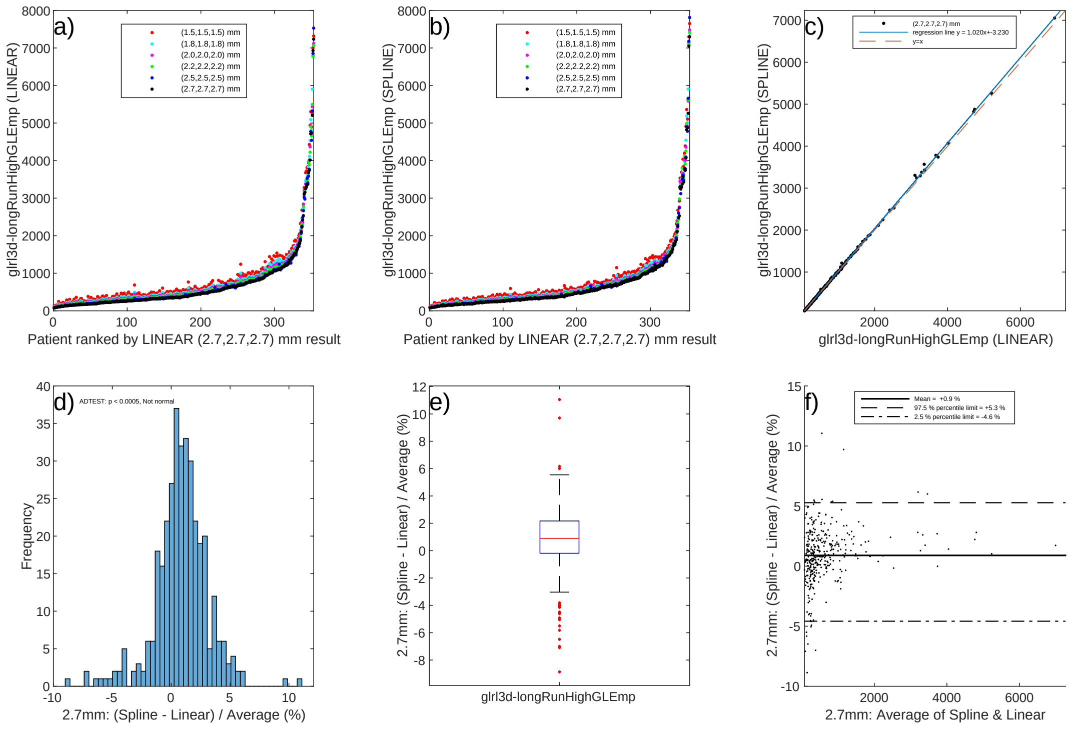

Figure 51: glrl3d-longRunHighGLEmp

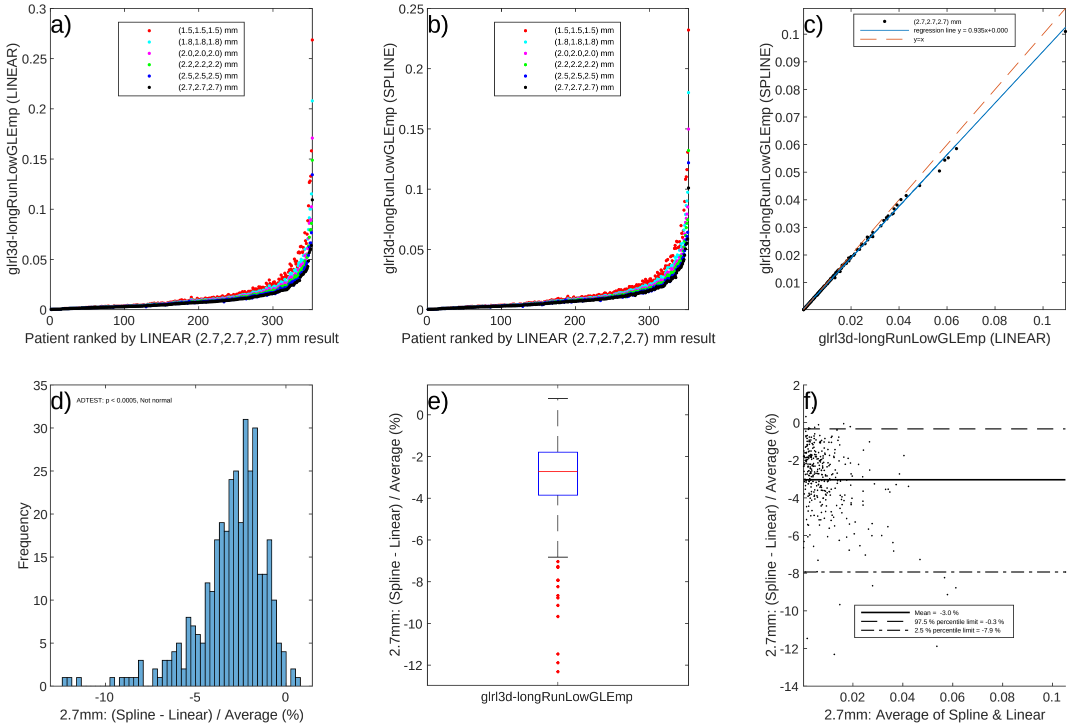

Figure 52: glrl3d-longRunLowGLEmp

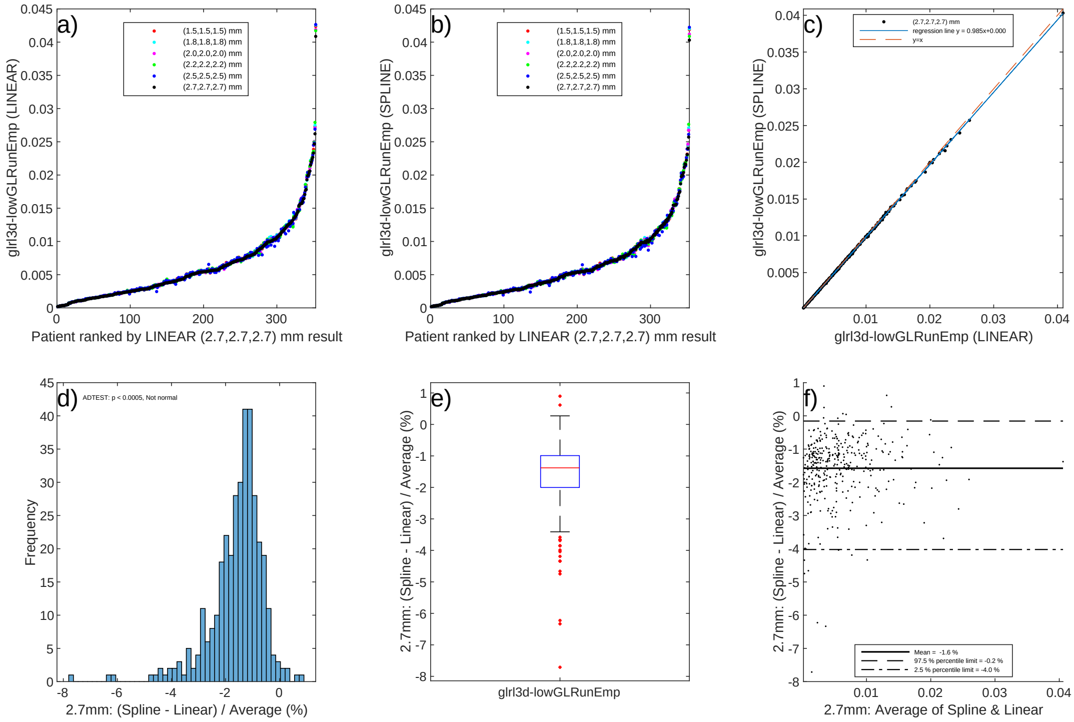

Figure 53: glrl3d-lowGLRunEmp

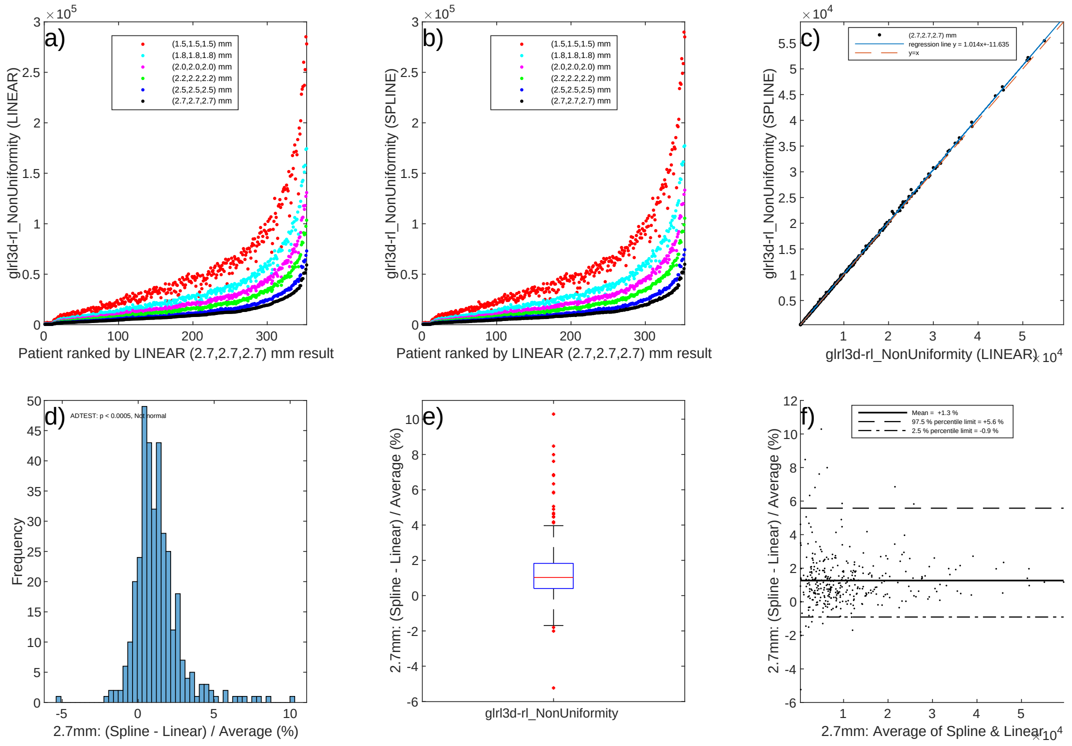

Figure 54: glrl3d-rl-NonUniformity

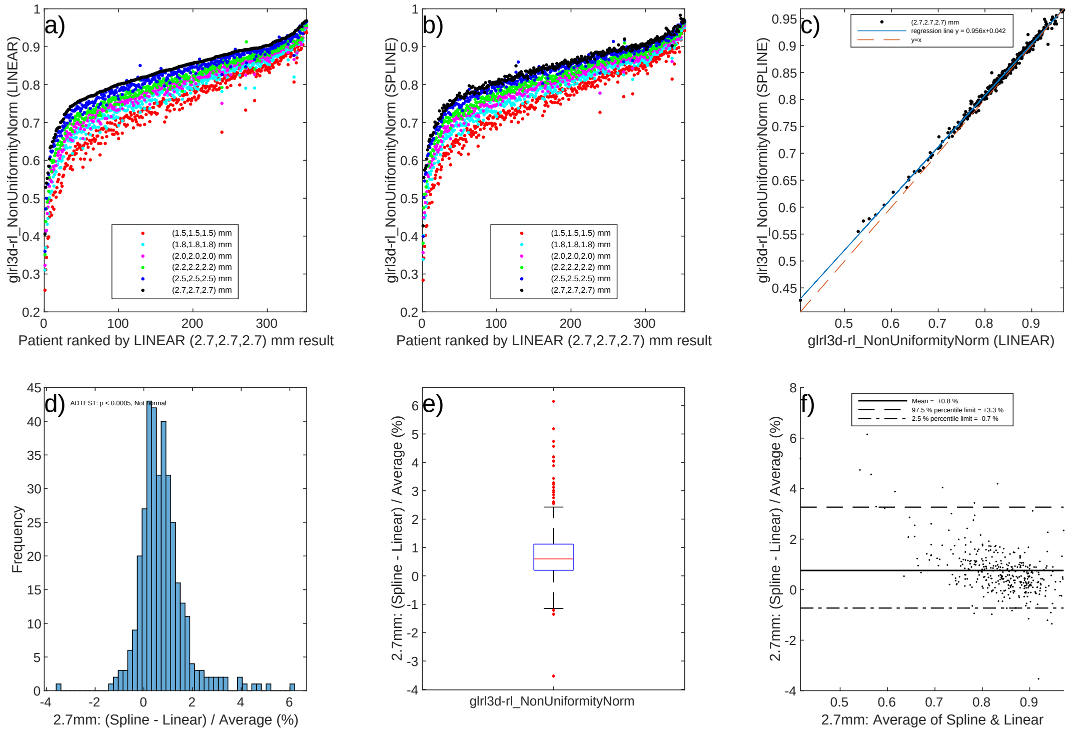

Figure 55: glrl3d-rl-NonUniformityNorm

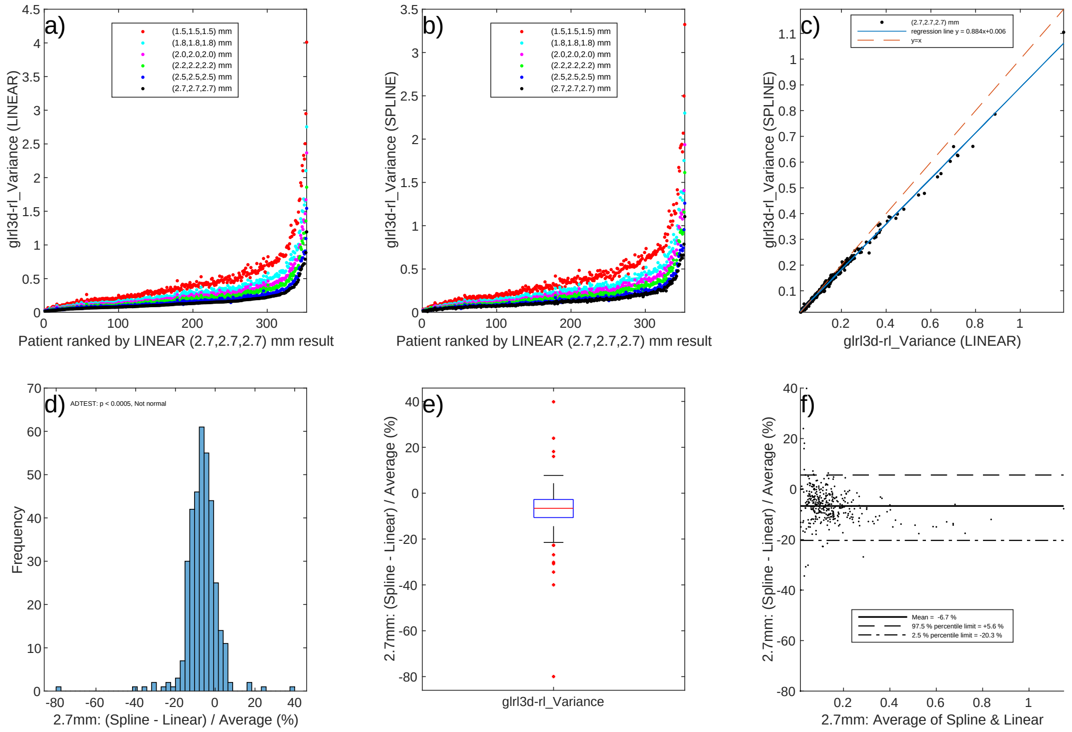

Figure 56: glrl3d-rl-Variance

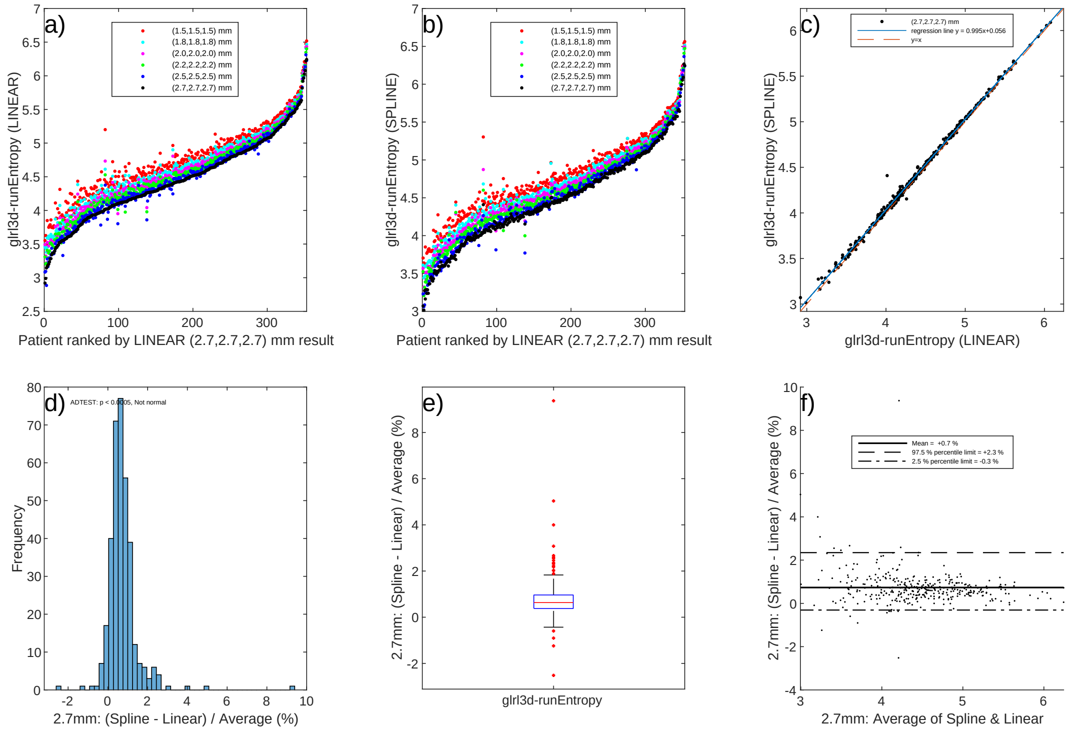

Figure 57: glrl3d-runEntropy

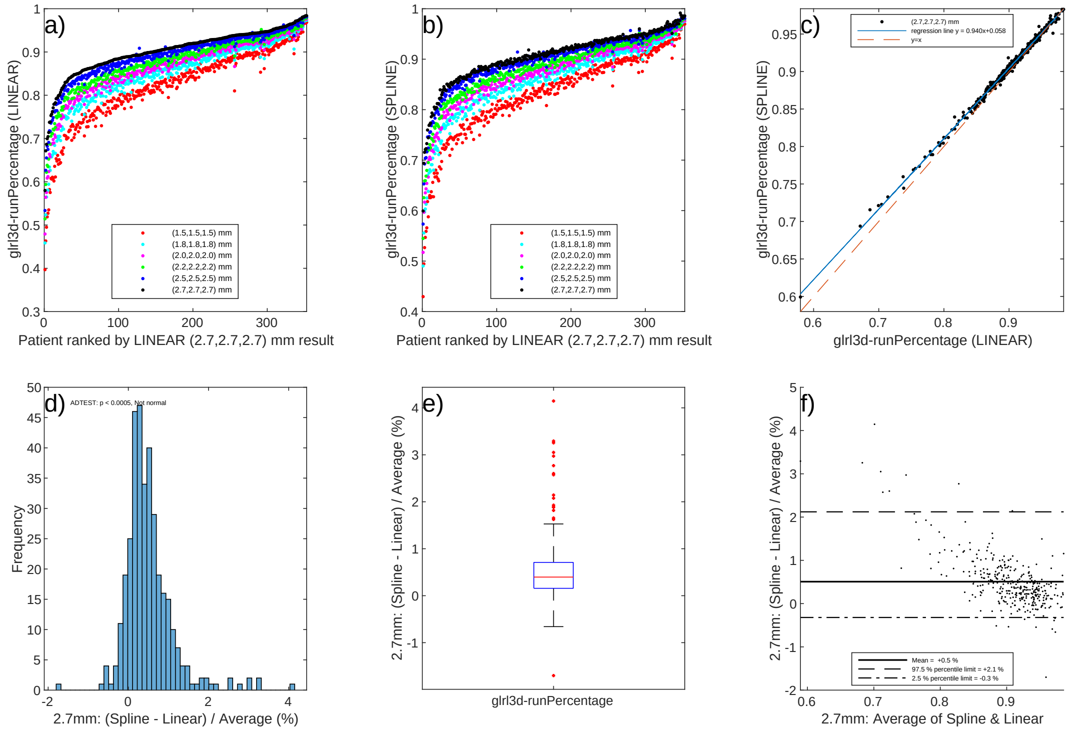

Figure 58: glrl3d-runPercentage

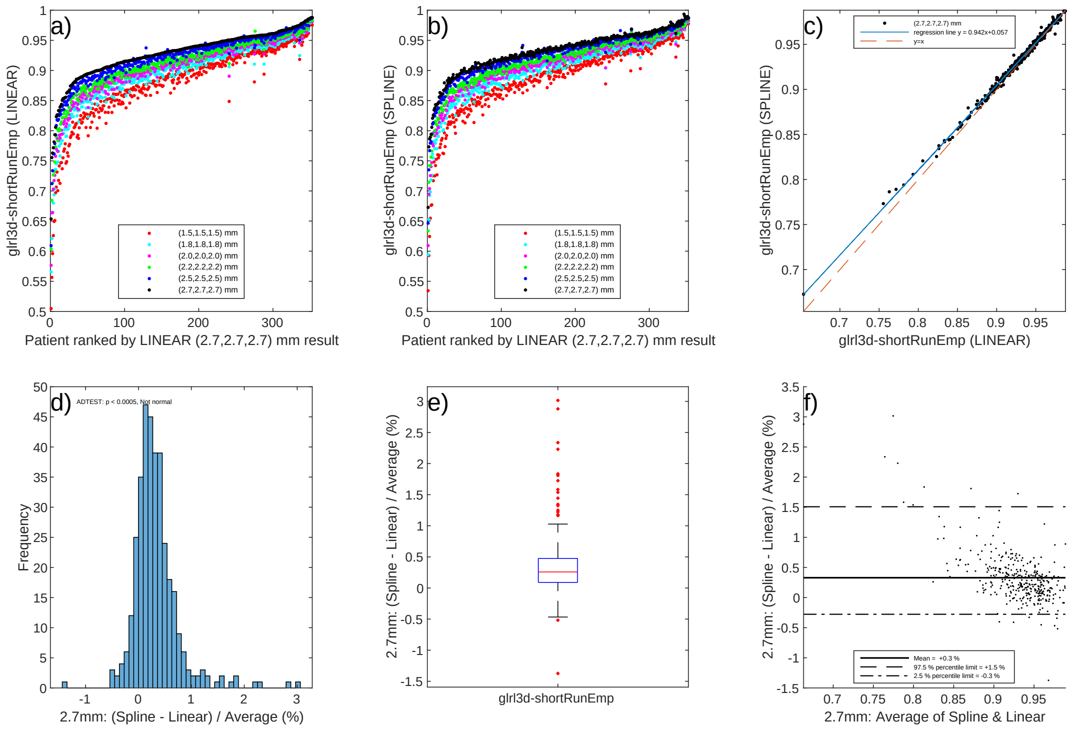

Figure 59: glrl3d-shortRunEmp

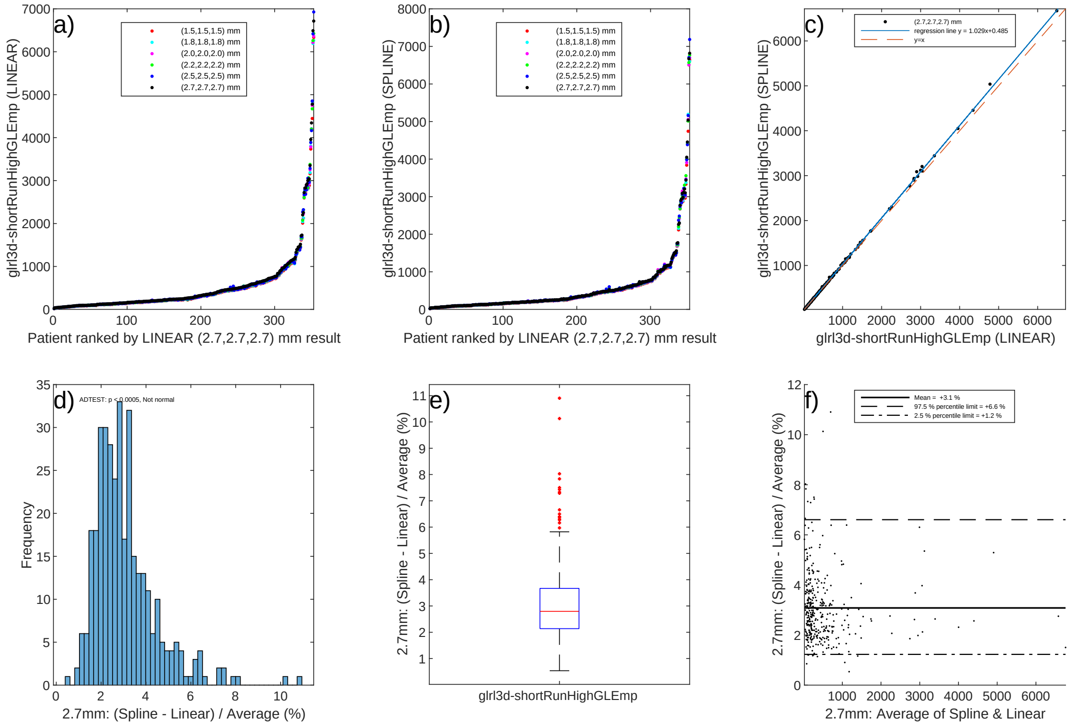

Figure 60: glrl3d-shortRunHighGLEmp

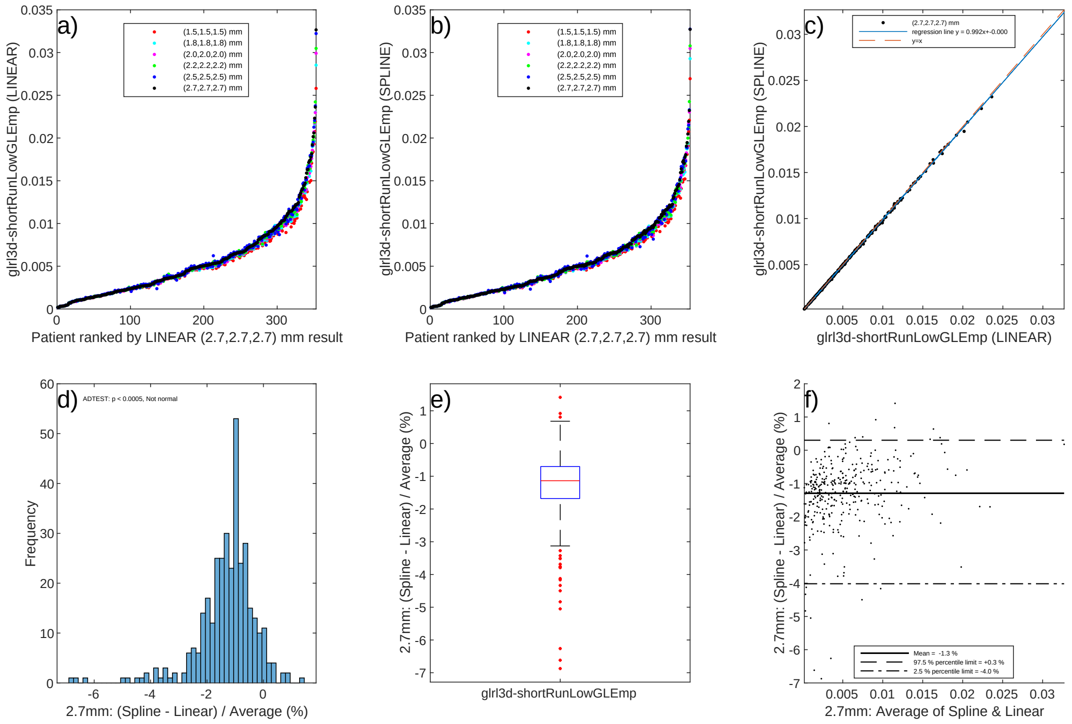

Figure 61: glrl3d-shortRunLowGLEmp

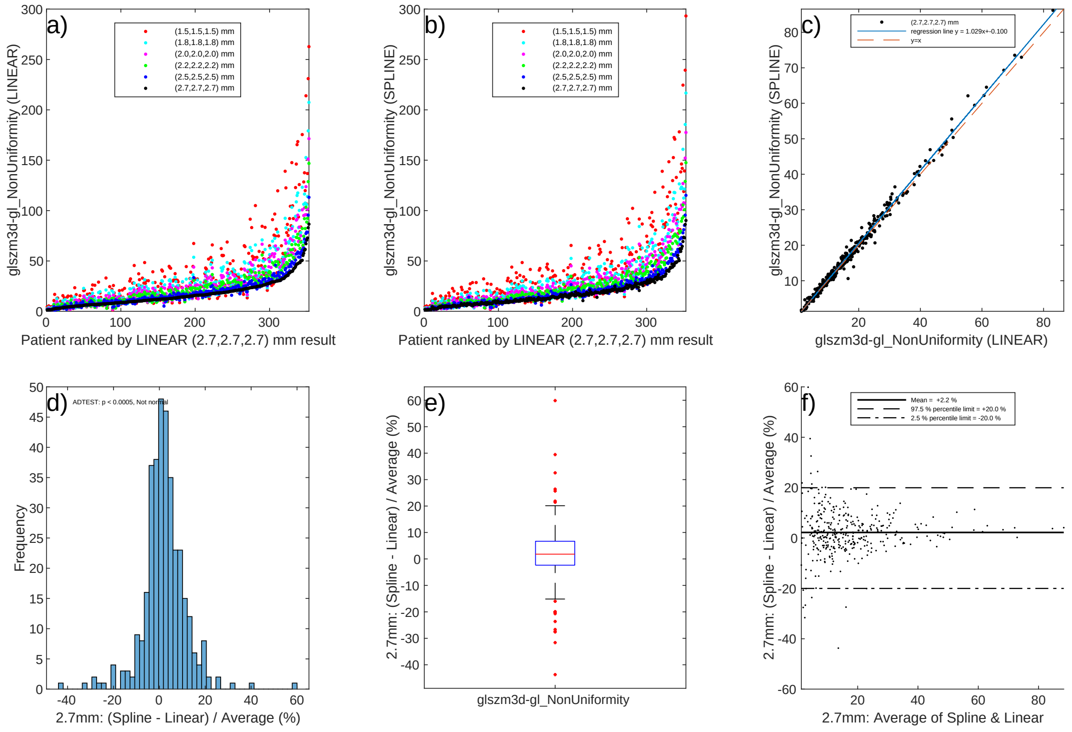

Figure 62: glszm3d-gl-NonUniformity

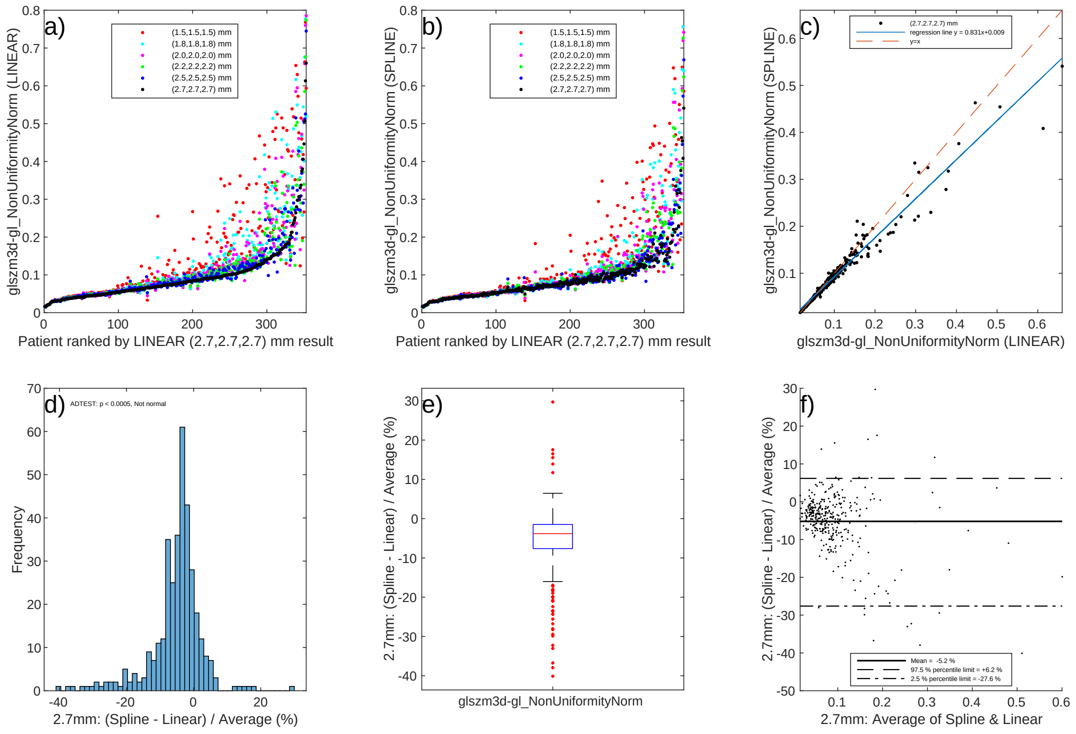

Figure 63: glszm3d-gl-NonUniformityNorm

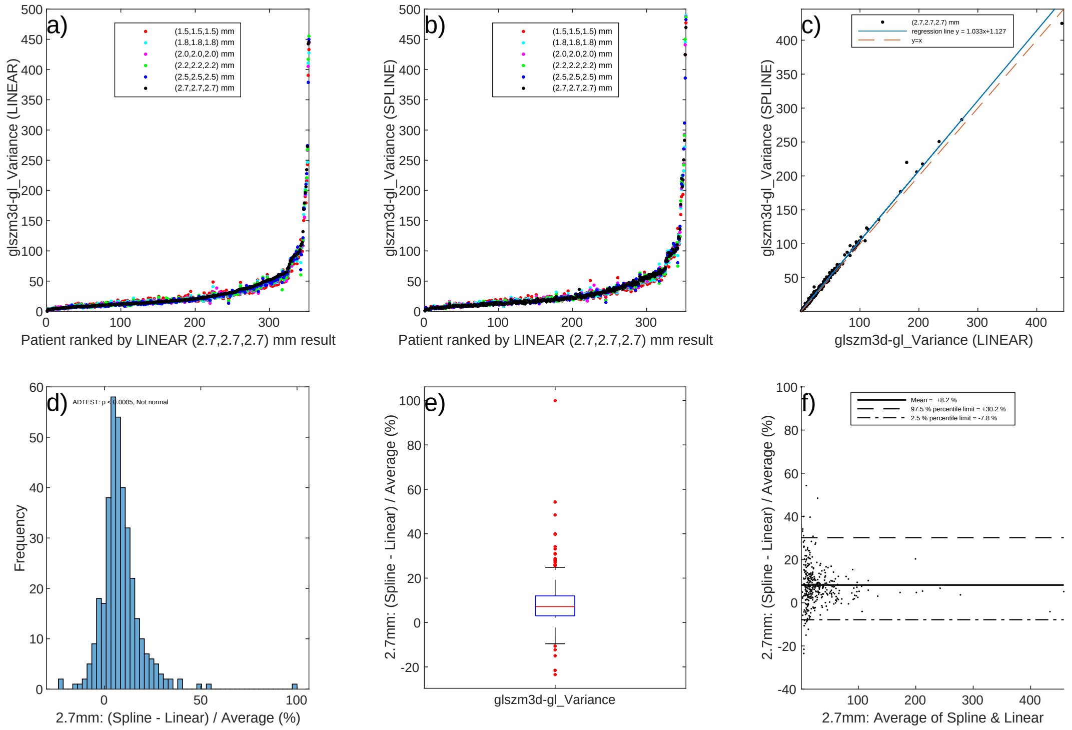

Figure 64: glszm3d-gl-Variance

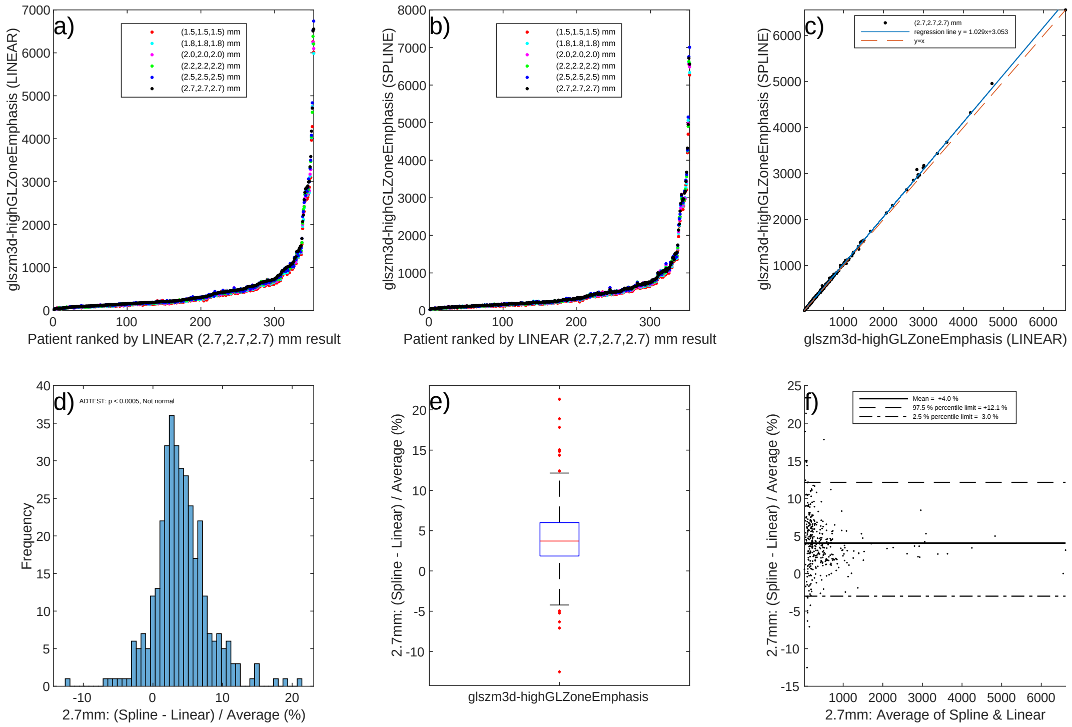

Figure 65: glszm3d-highGLZoneEmphasis

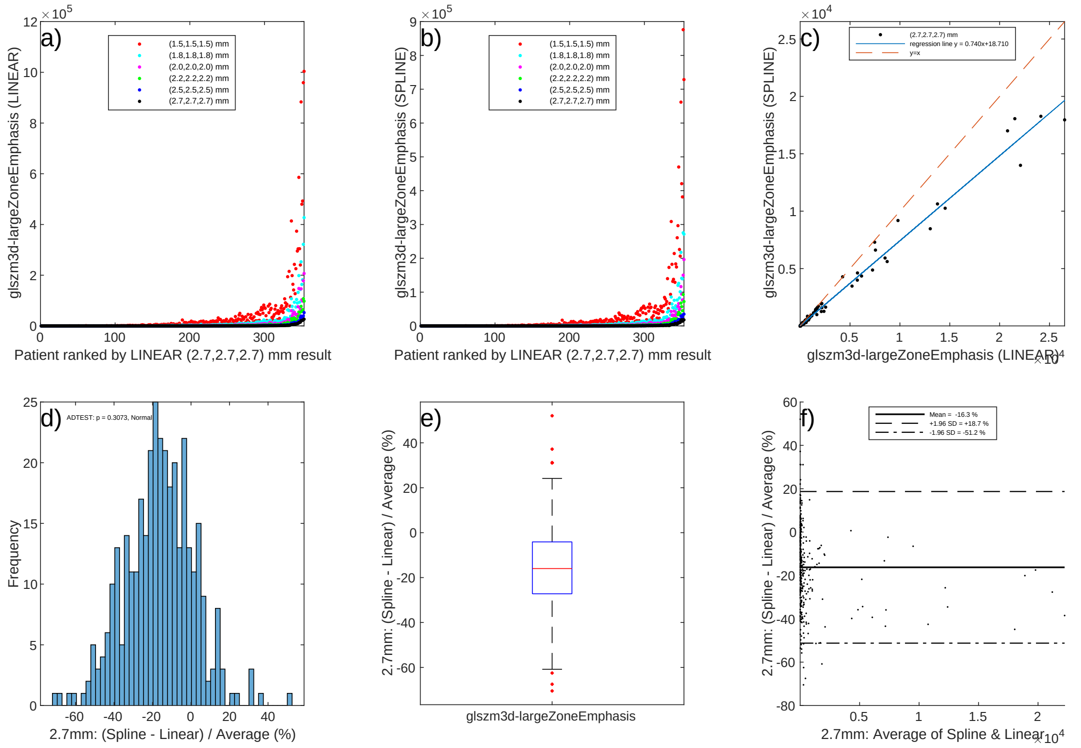

Figure 66: glszm3d-largeZoneEmphasis

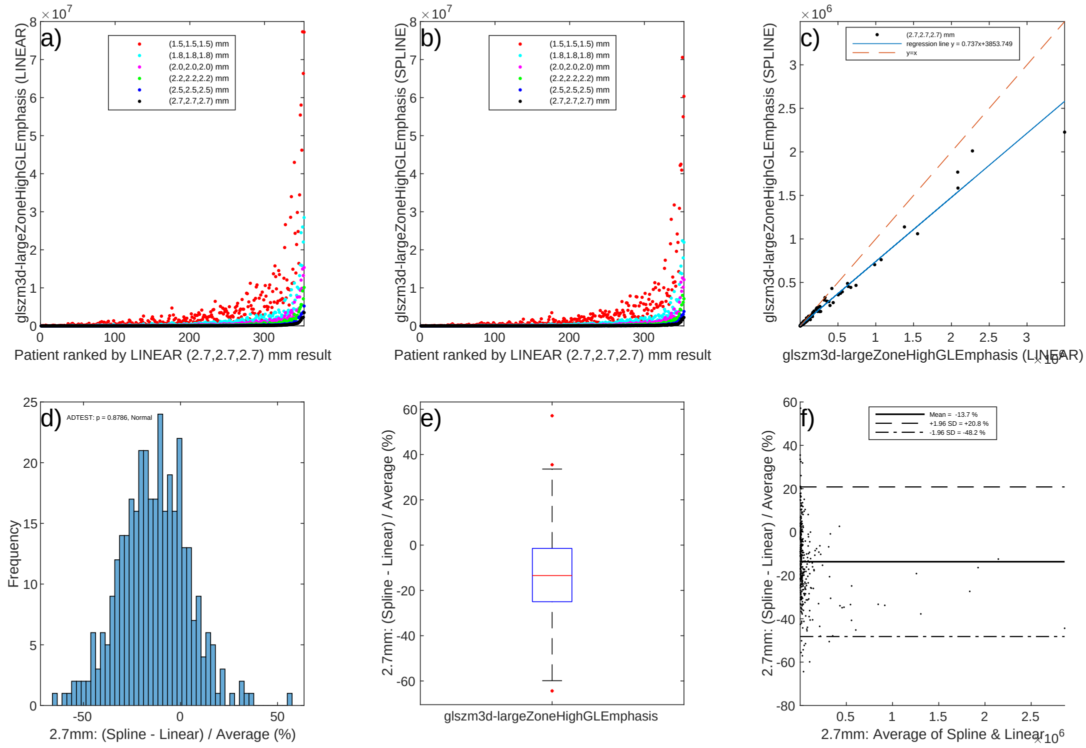

Figure 67: glszm3d-largeZoneHighGLEmphasis

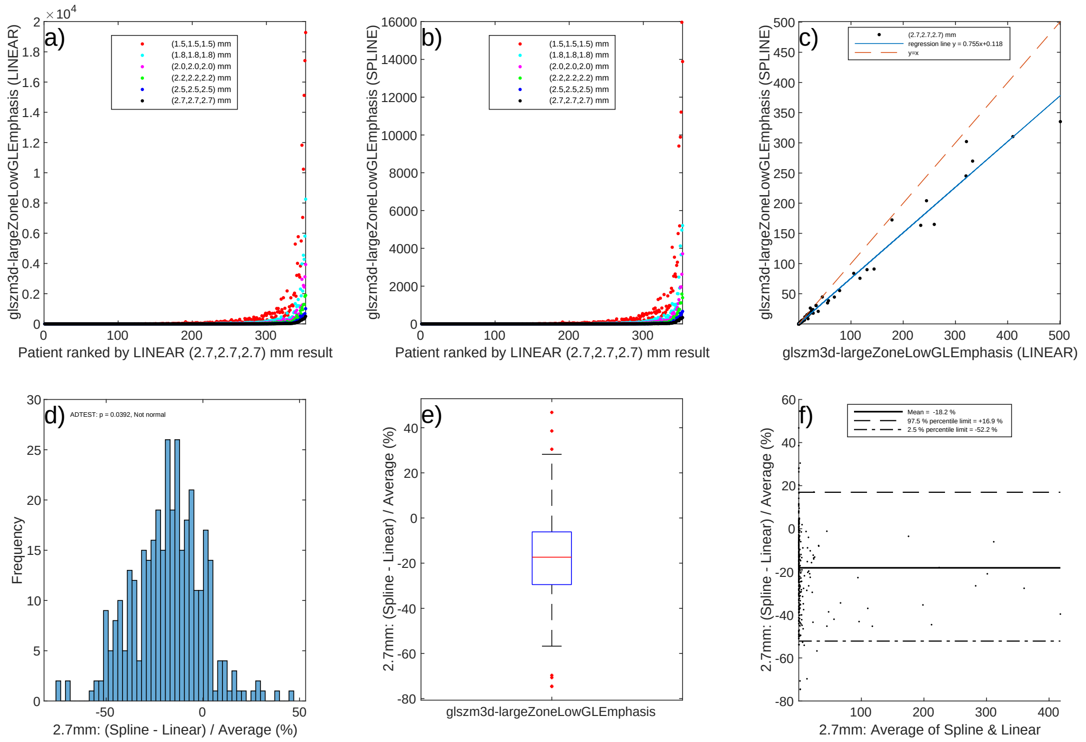

Figure 68: glszm3d-largeZoneLowGLEmphasis

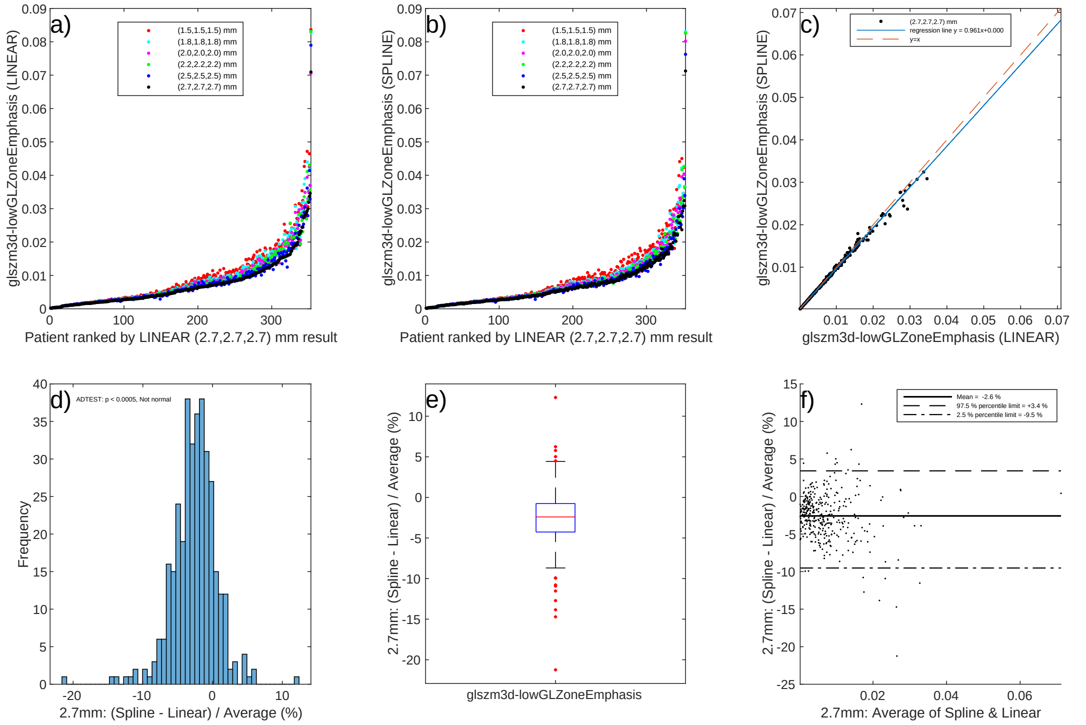

Figure 69: glszm3d-lowGLZoneEmphasis

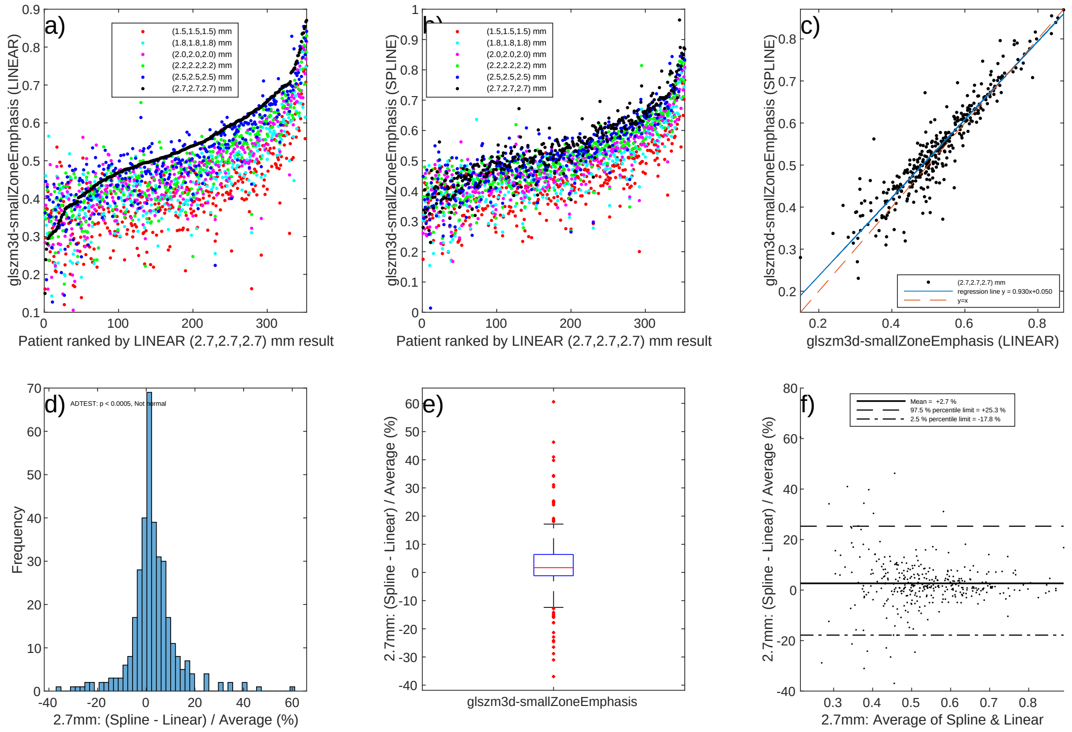

Figure 70: glszm3d-smallZoneEmphasis

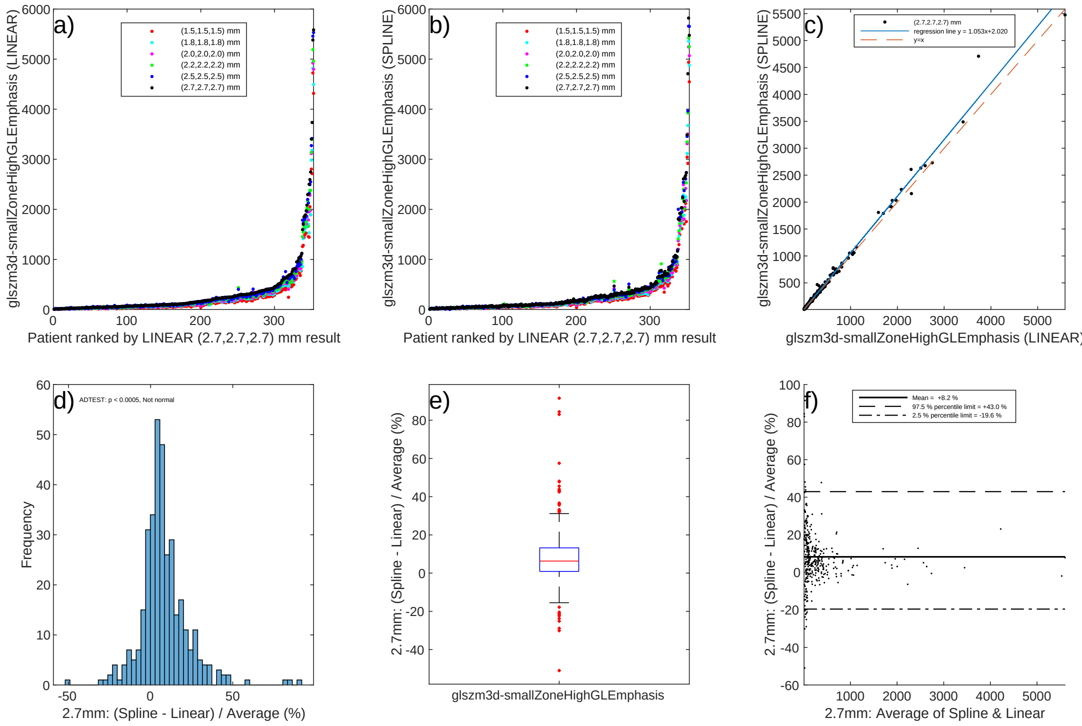

Figure 71: glszm3d-smallZoneHighGLEmphasis

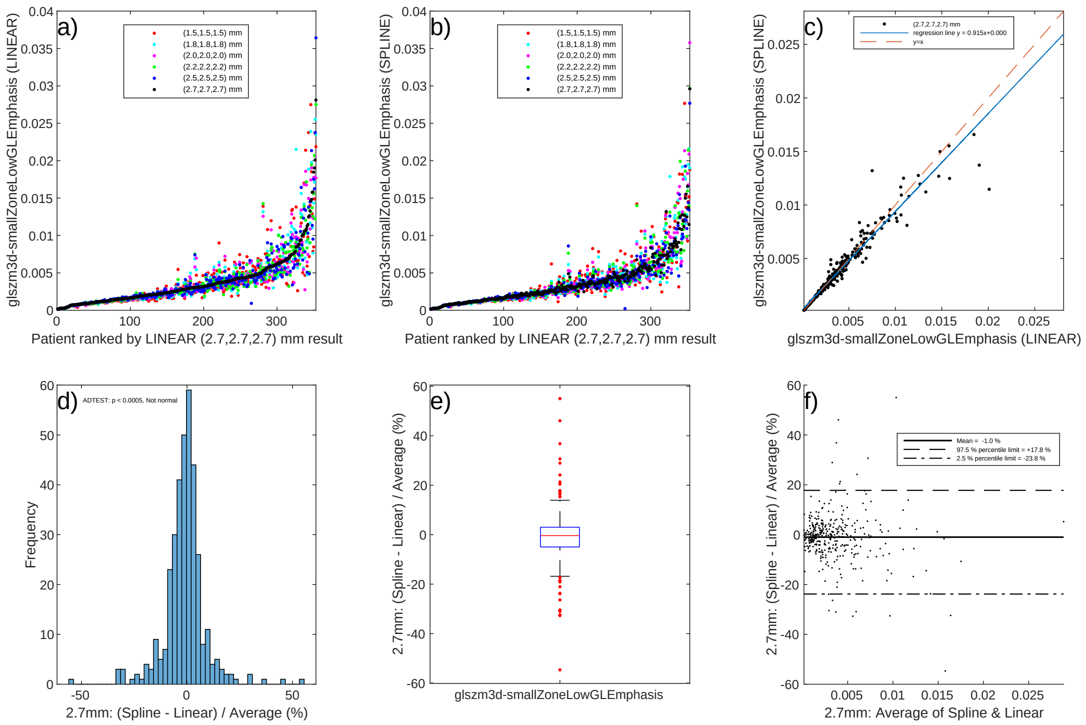

Figure 72: glszm3d-smallZoneLowGLEmphasis

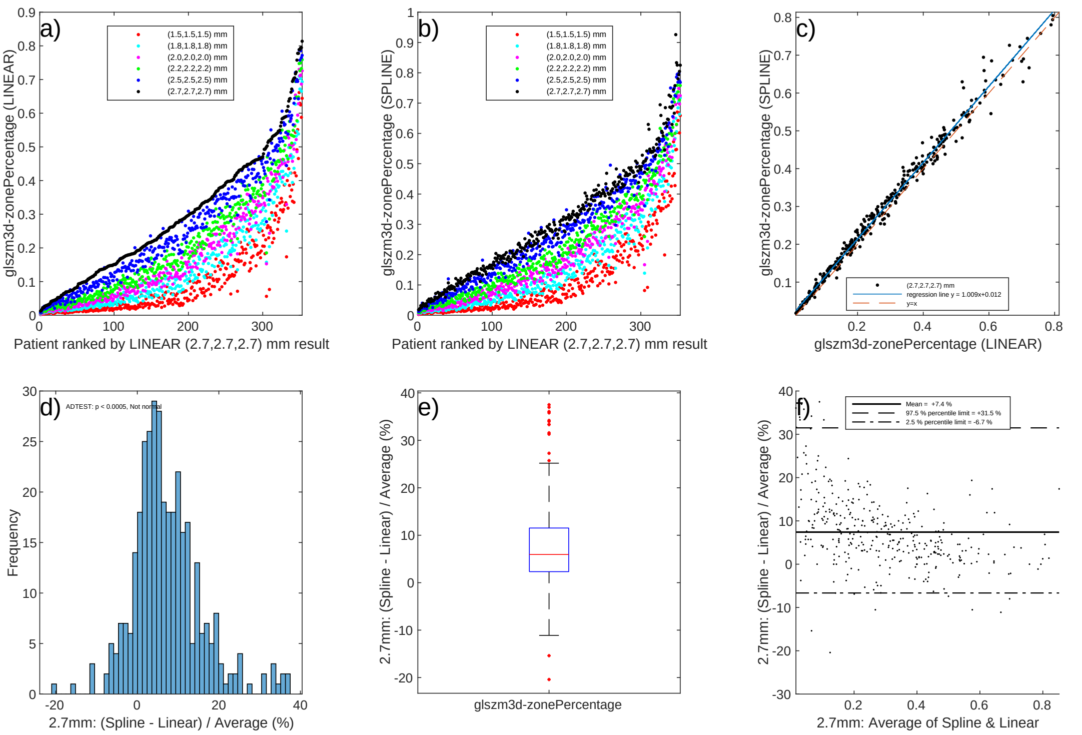

Figure 73: glszm3d-zonePercentage

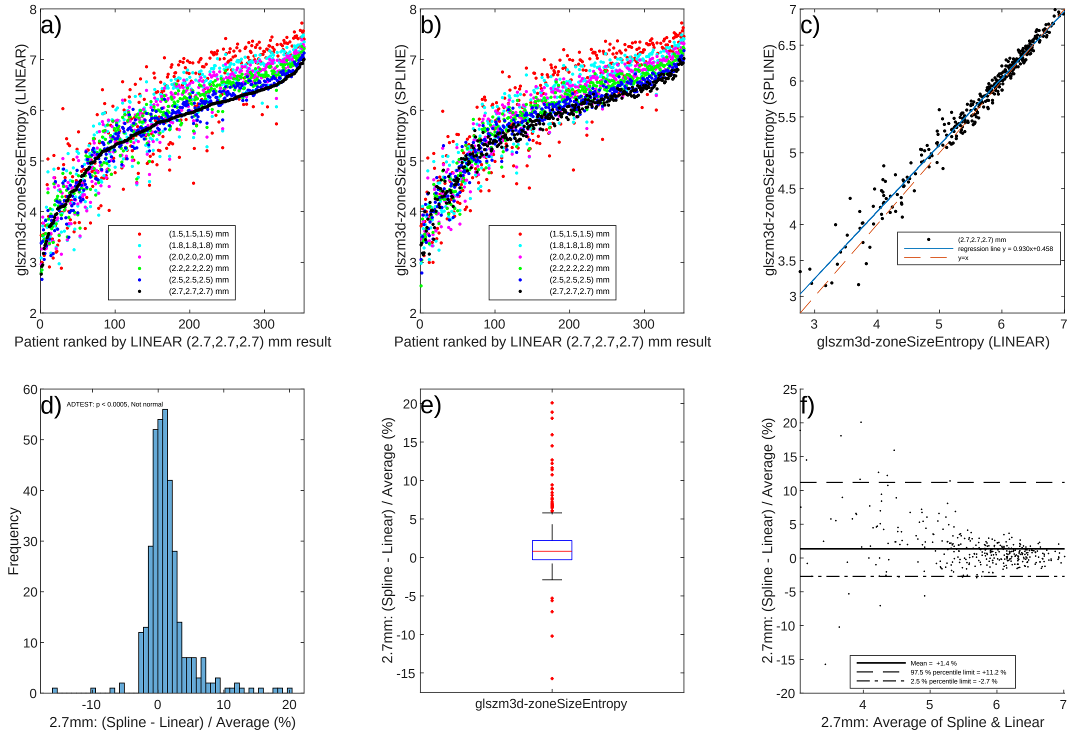

Figure 74: glszm3d-zoneSizeEntropy

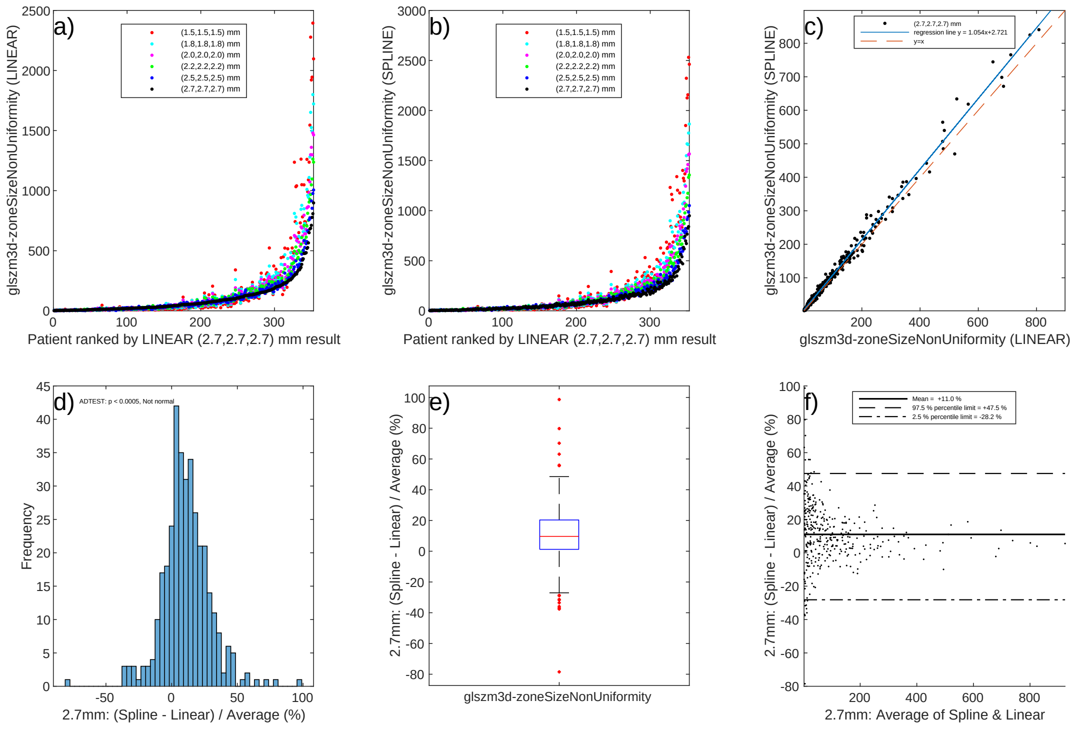

Figure 75: glszm3d-zoneSizeNonUniformity

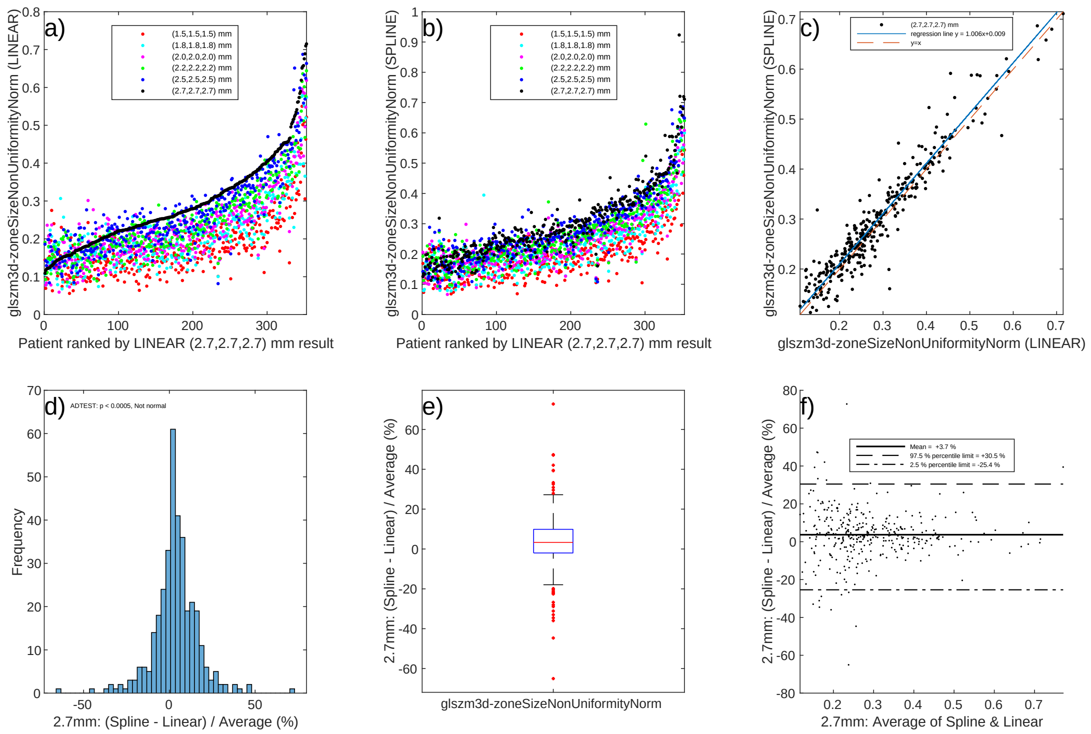

Figure 76: glszm3d-zoneSizeNonUniformityNorm

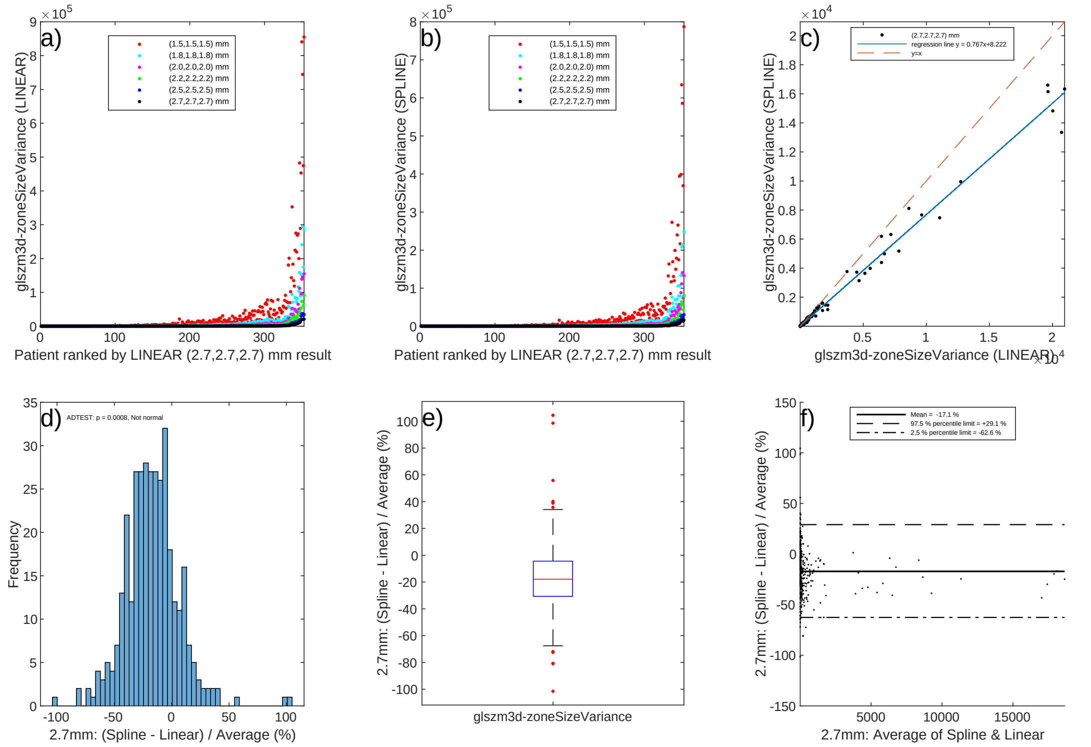

Figure 77: glszm3d-zoneSizeVariance

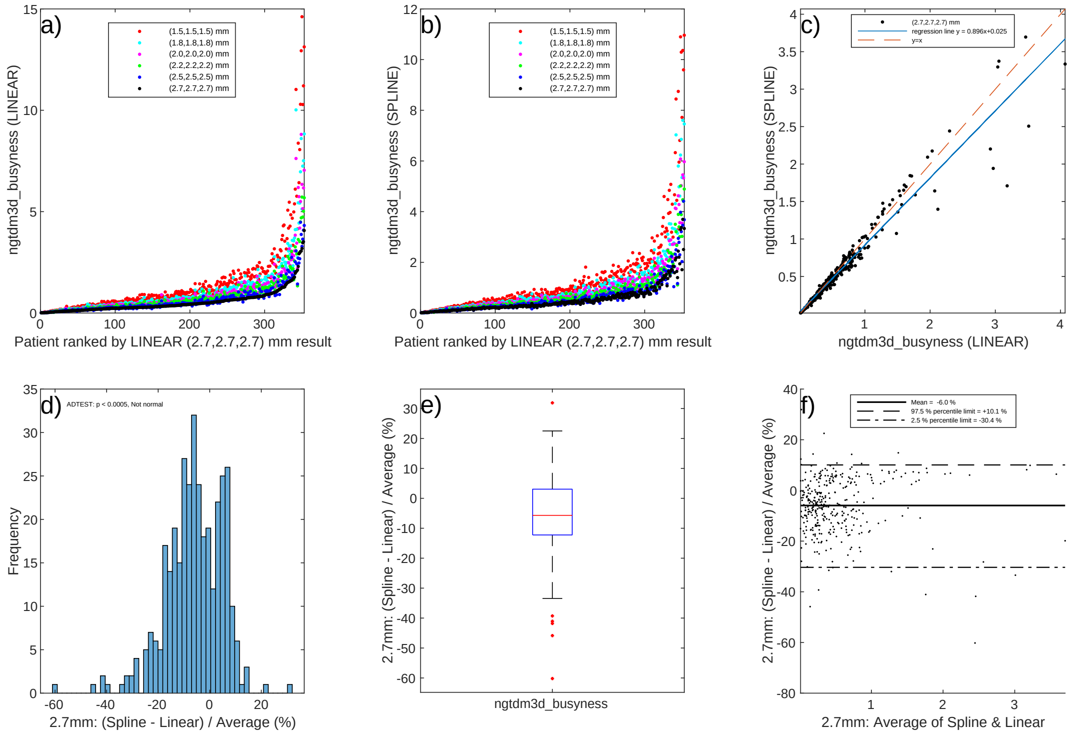

Figure 78: ngtdm3d-busyness

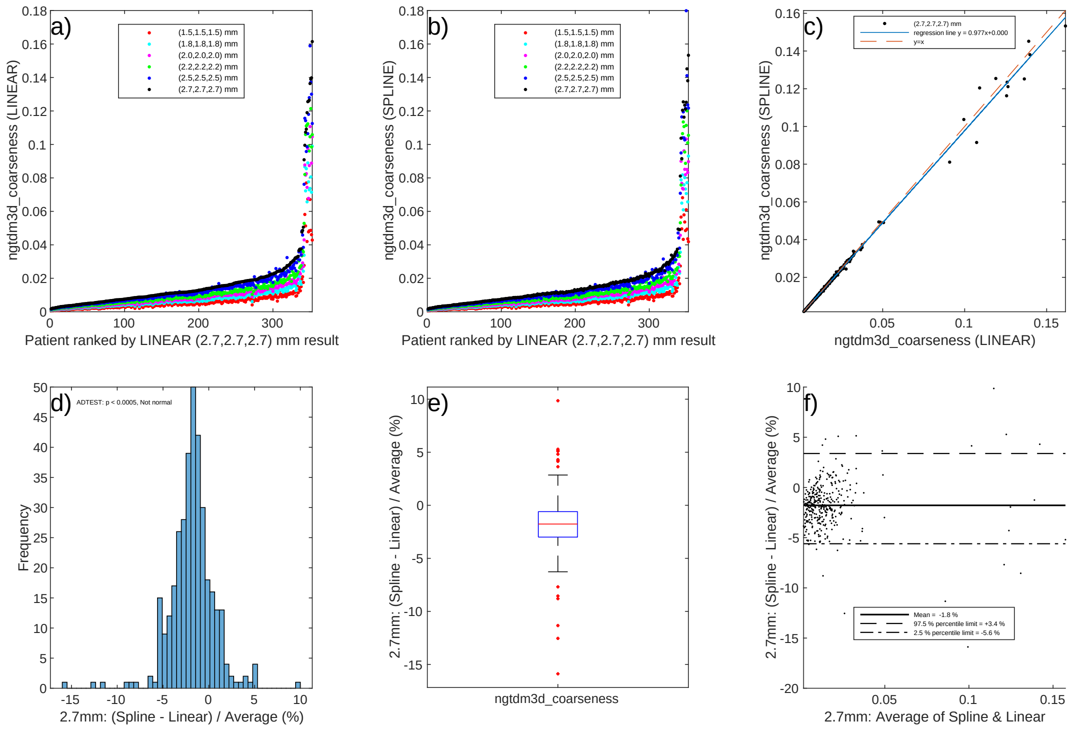

Figure 79: ngtdm3d-coarseness

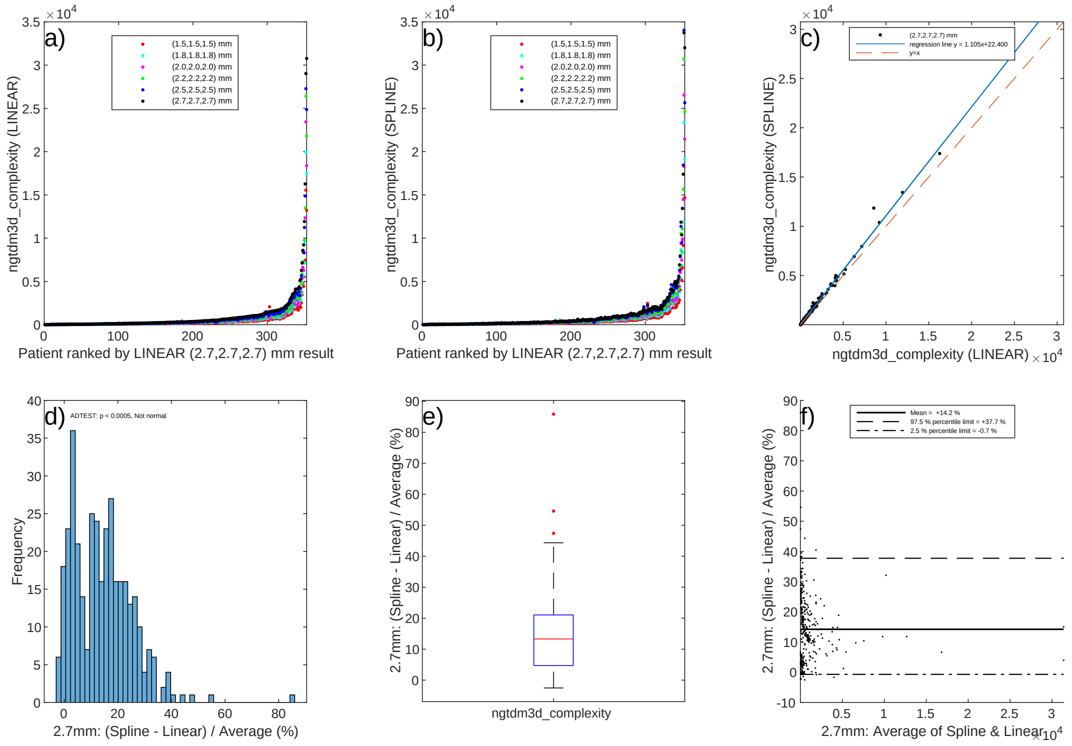

Figure 80: ngtdm3d-complexity

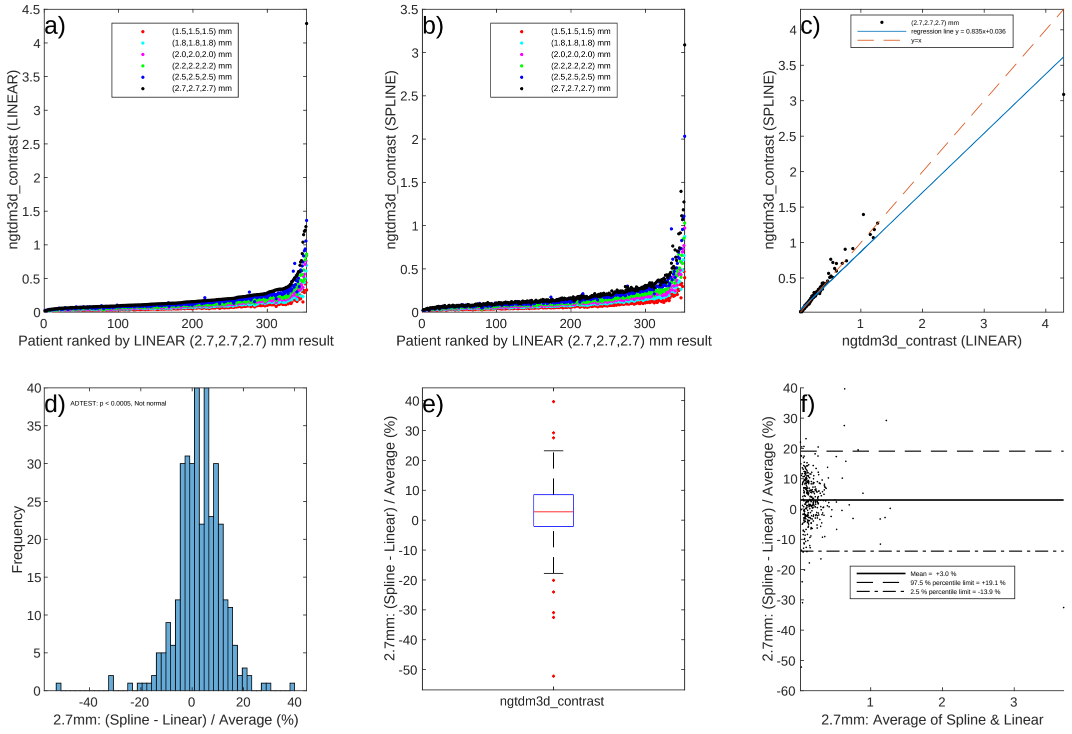

Figure 81: ngtdm3d-contrast

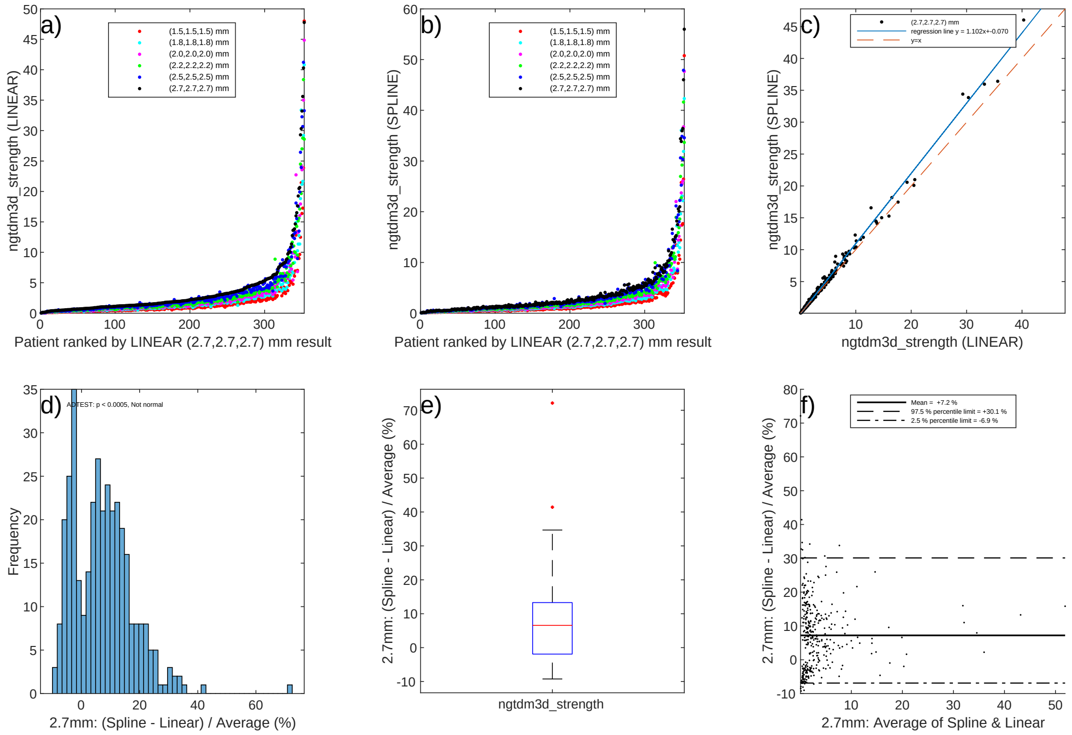

Figure 82: ngtdm3d-strength

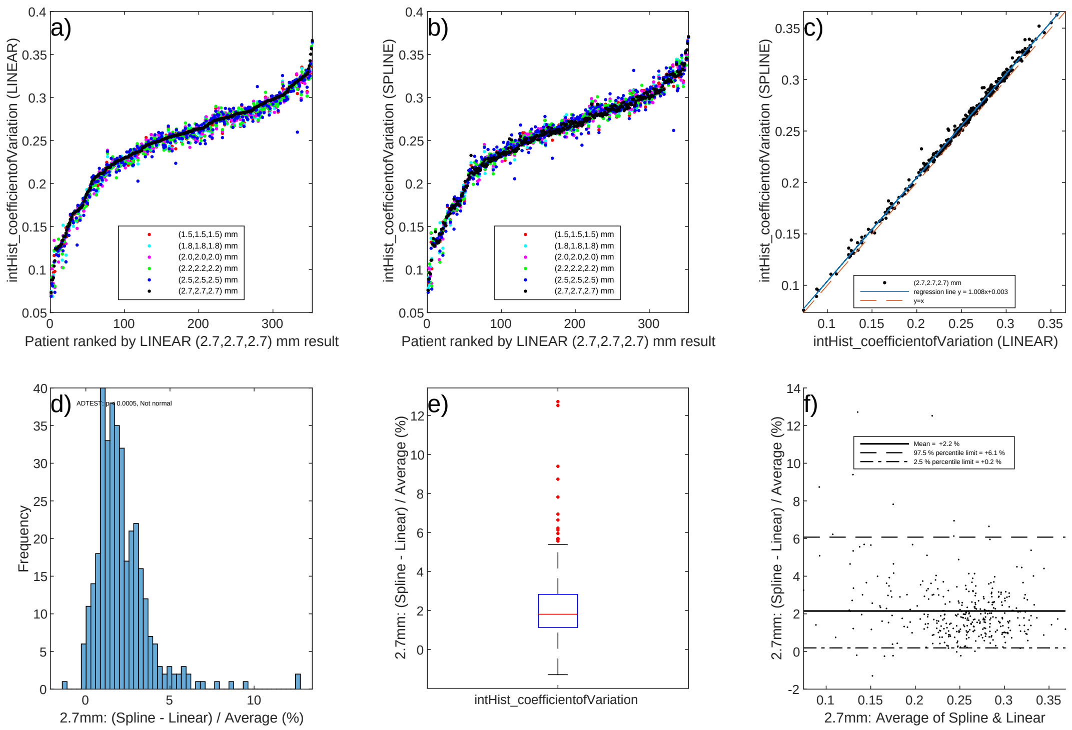

Figure 83: intHist-coefficientofVariation

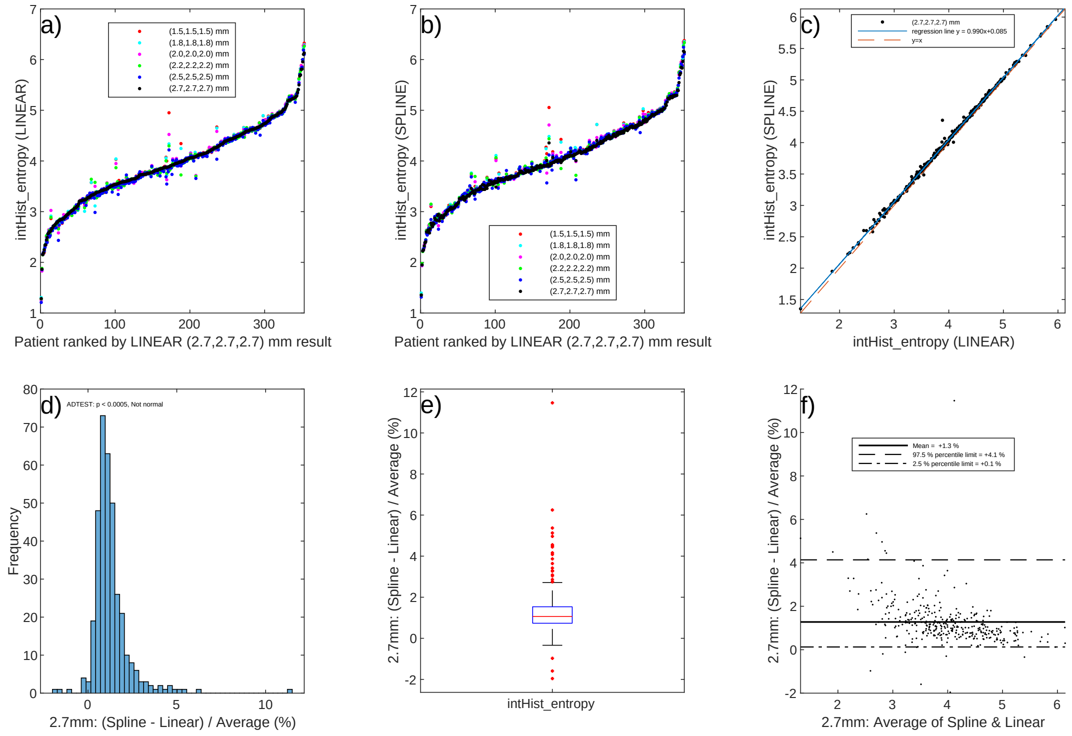

Figure 84: intHist-entropy

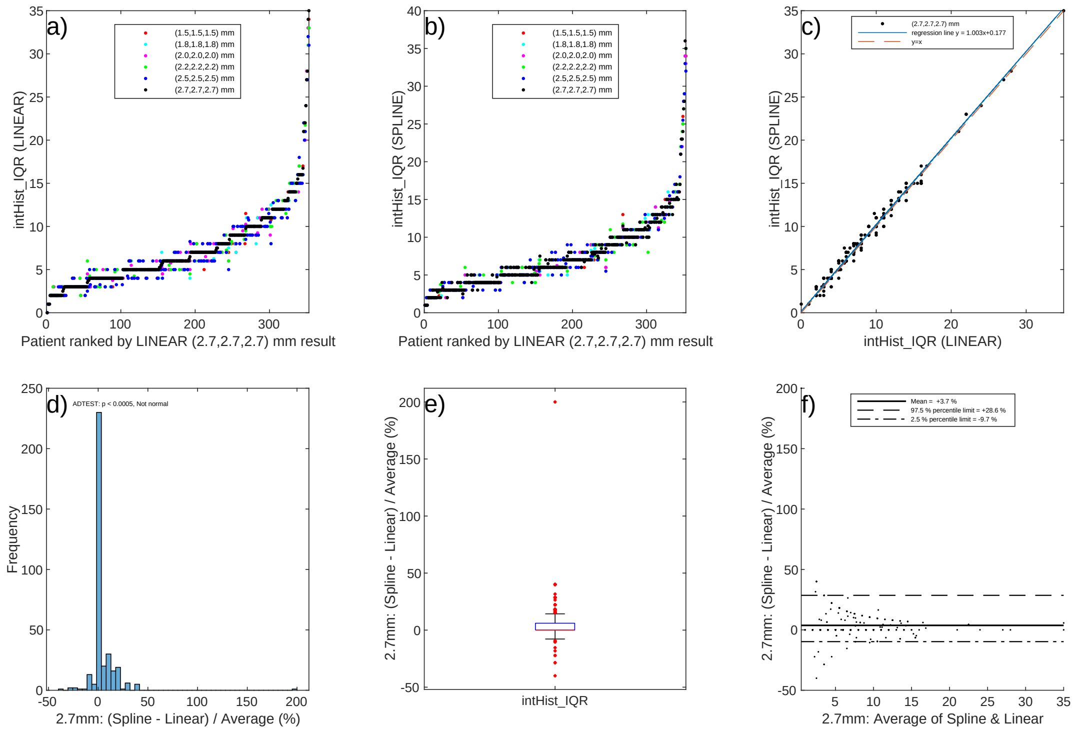

Figure 85: intHist-IQR

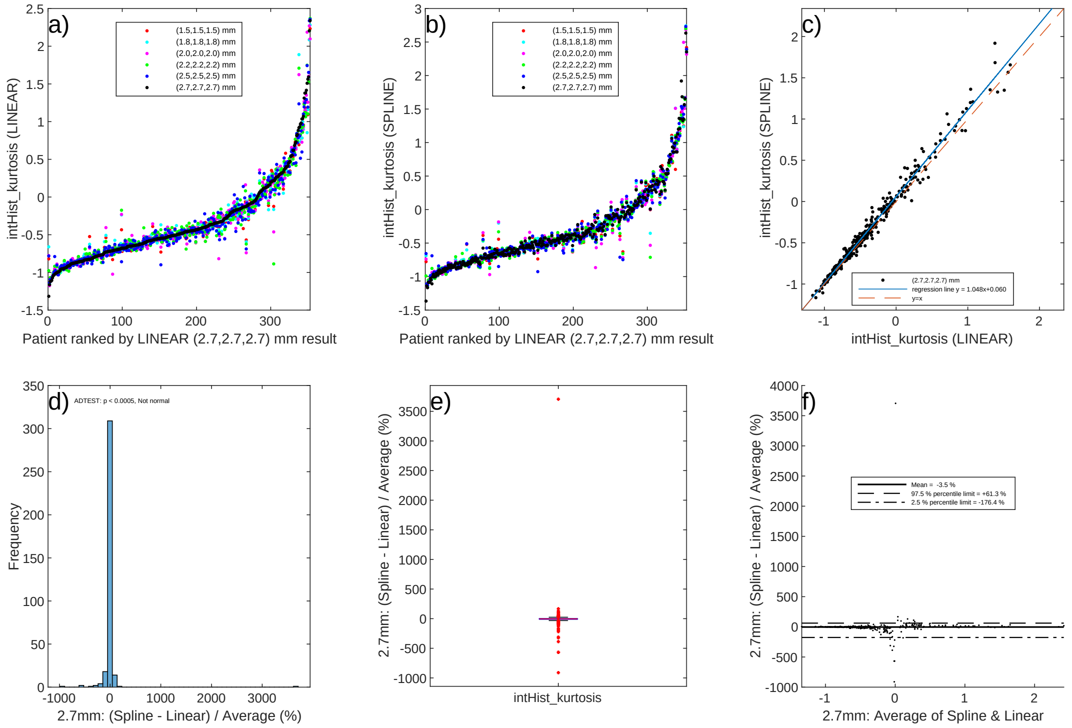

Figure 86: intHist-kurtosis

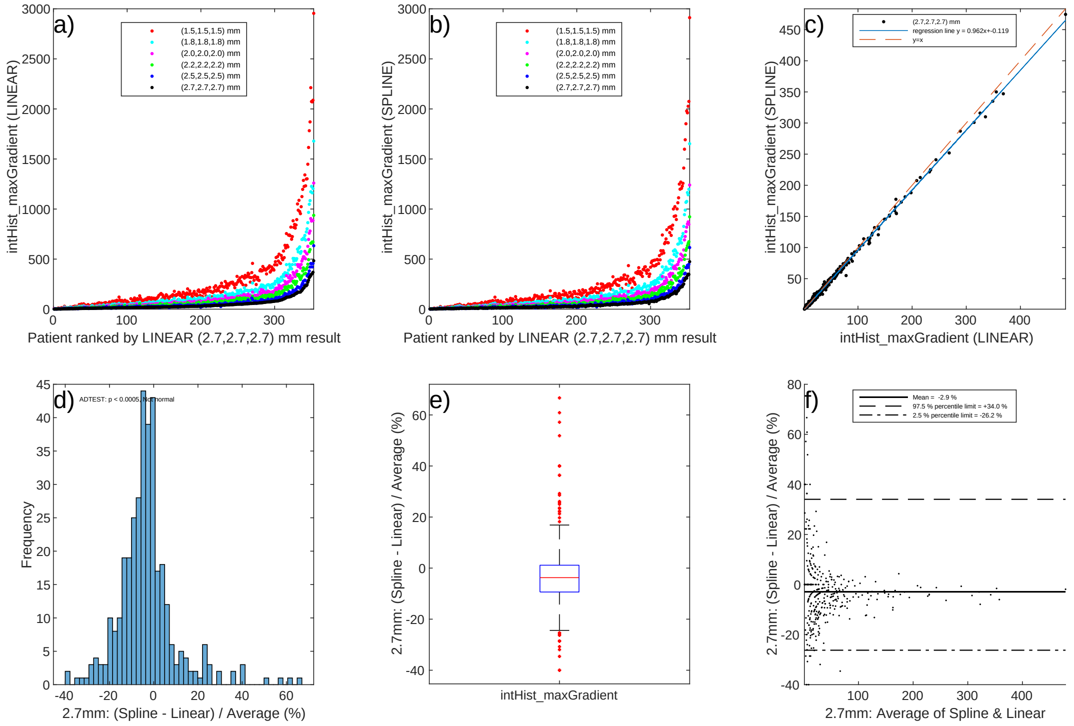

Figure 87: intHist-maxGradient

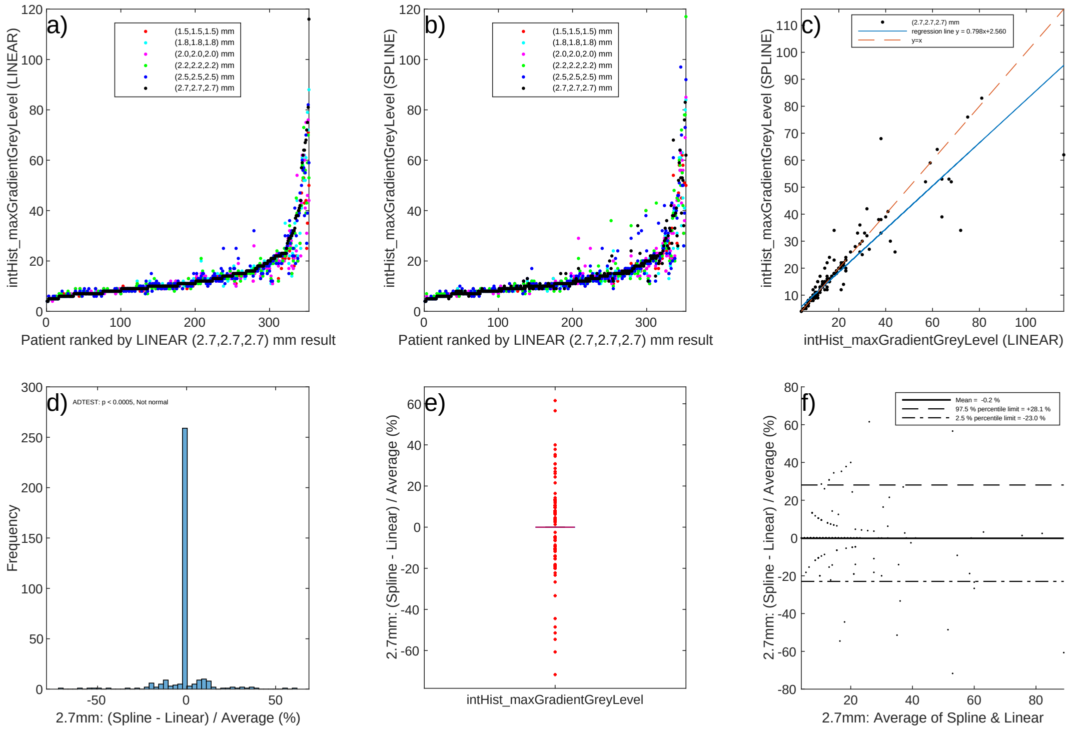

Figure 88: intHist-maxGradientGreyLevel

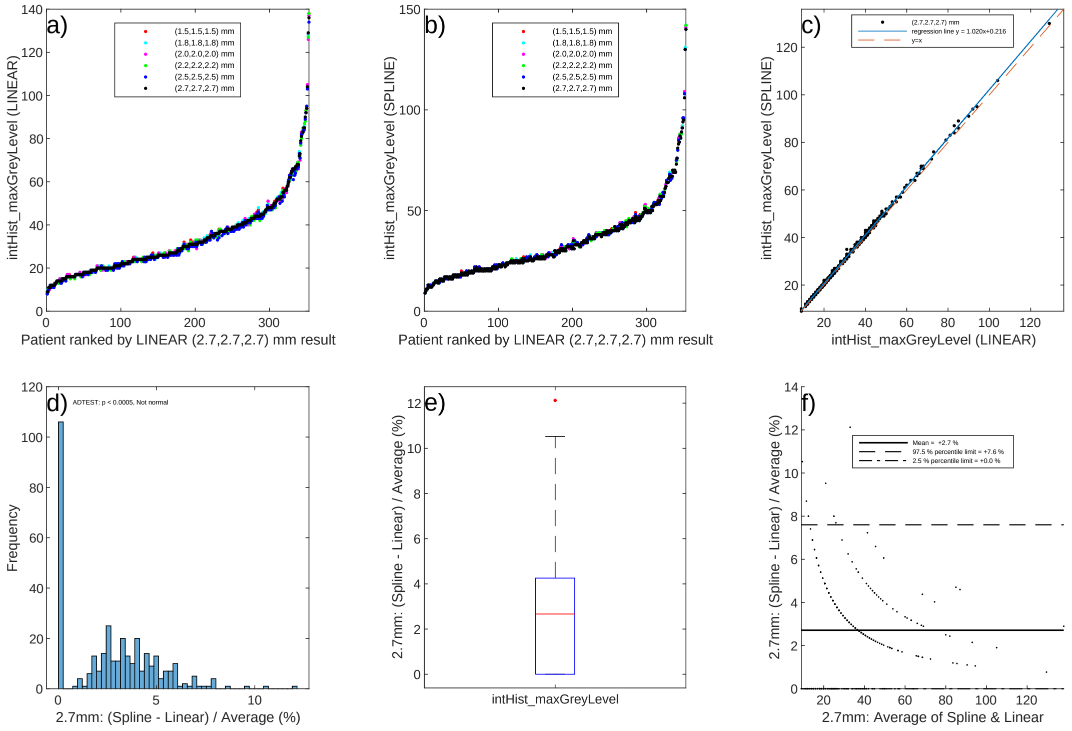

Figure 89: intHist-maxGreyLevel

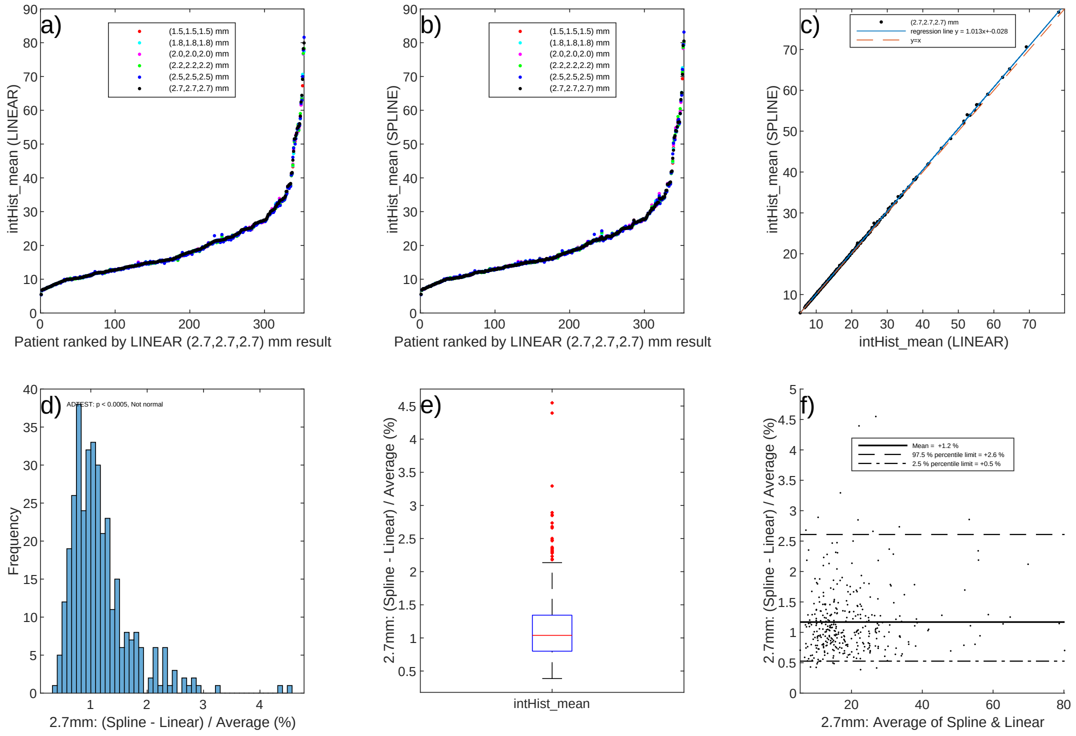

Figure 90: intHist-mean

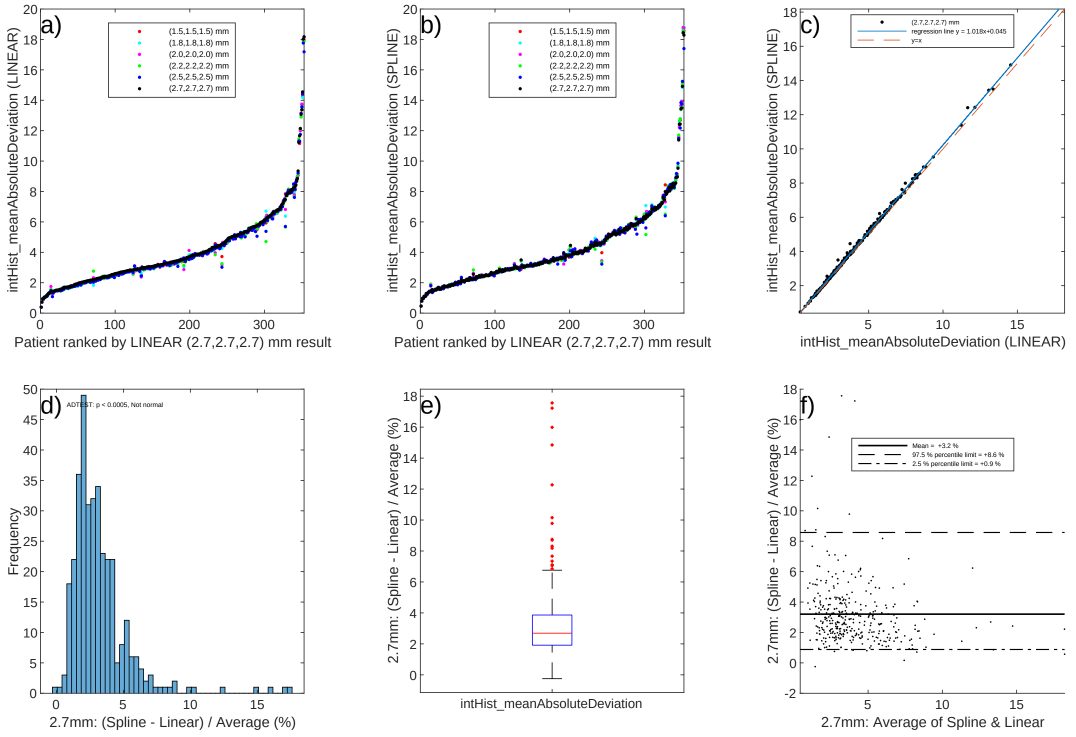

Figure 91: intHist-meanAbsoluteDeviation

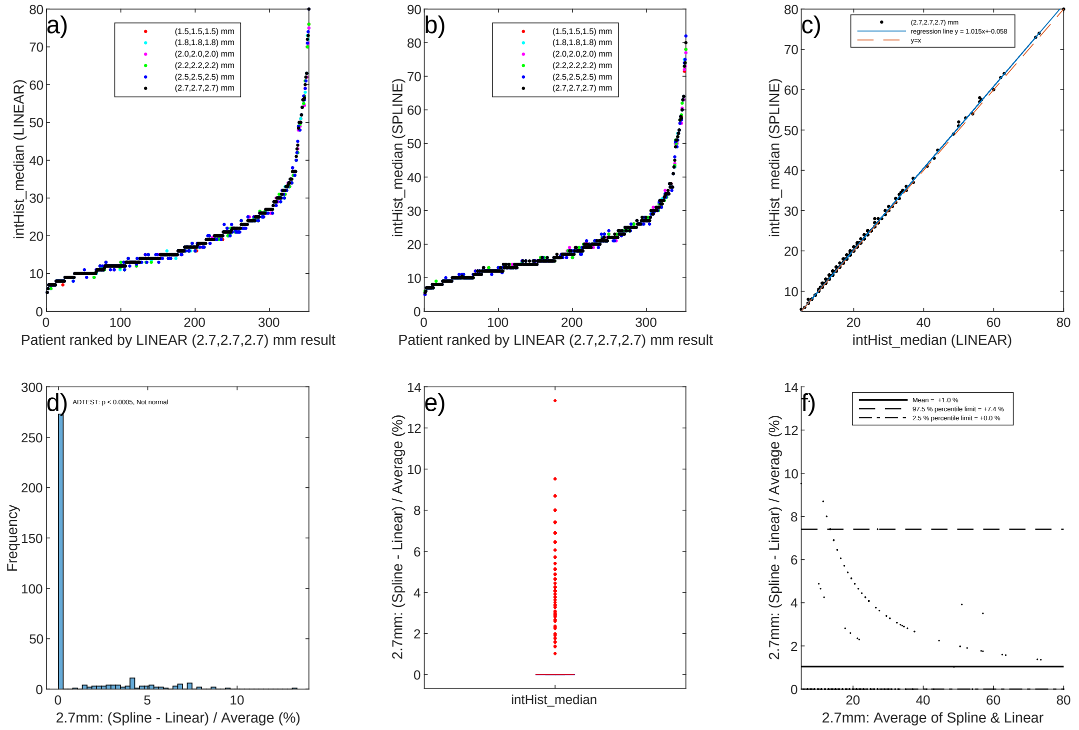

Figure 92: intHist-median

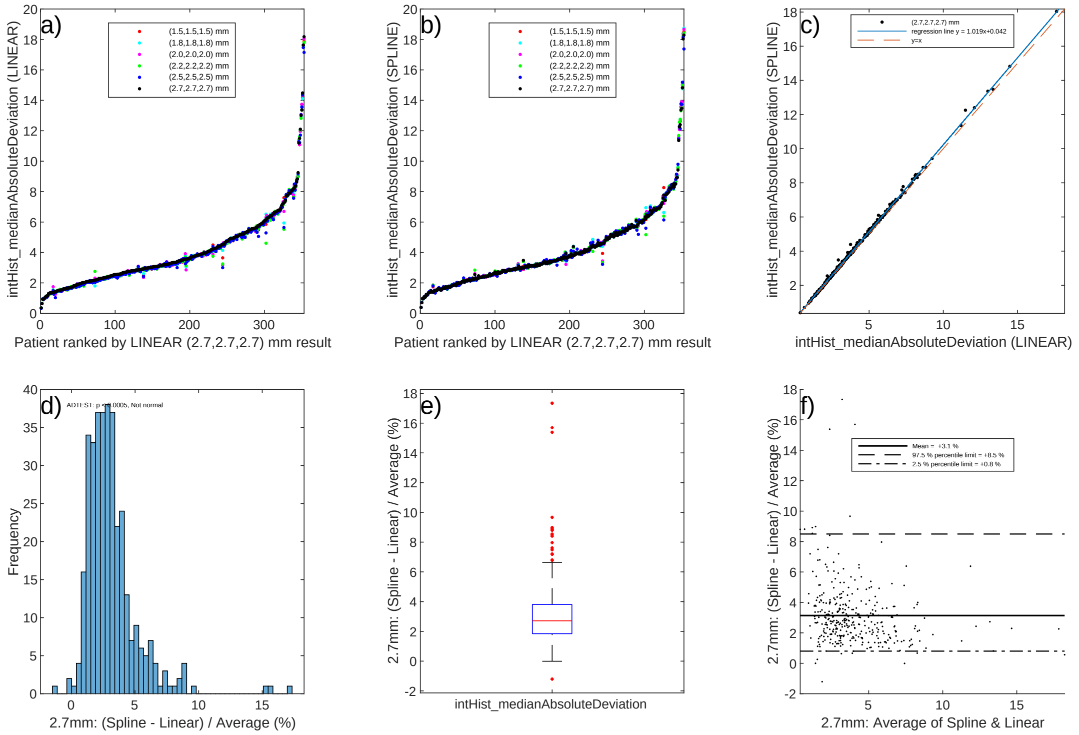

Figure 93: intHist-medianAbsoluteDeviation

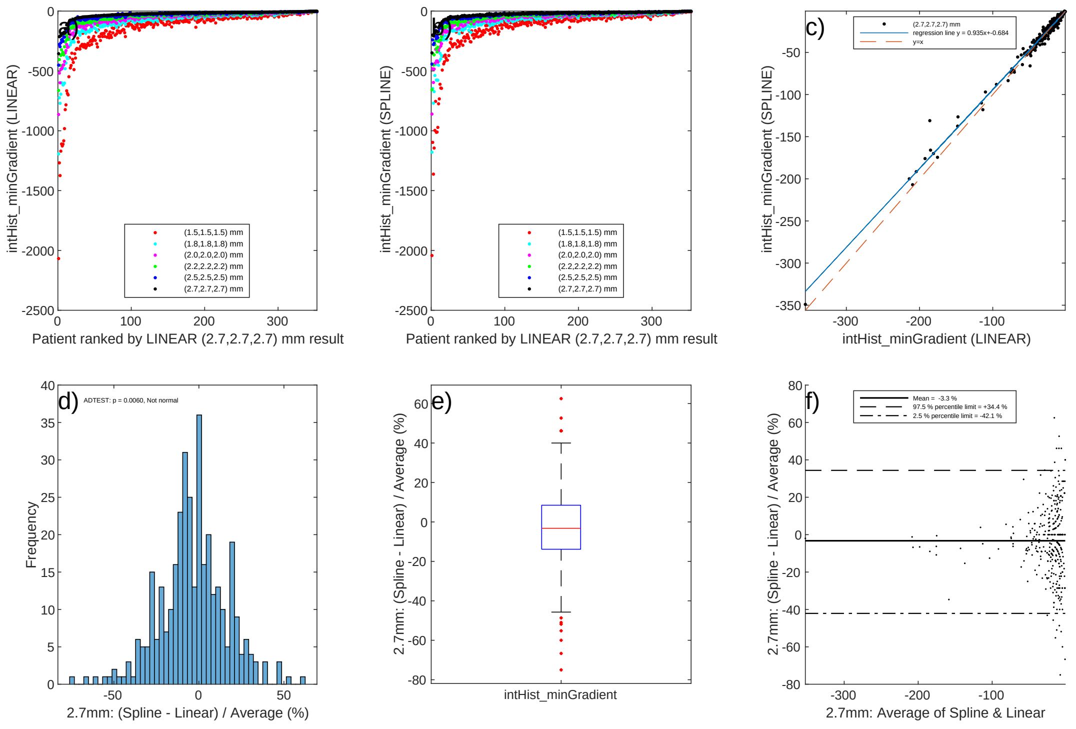

Figure 94: intHist-minGradient

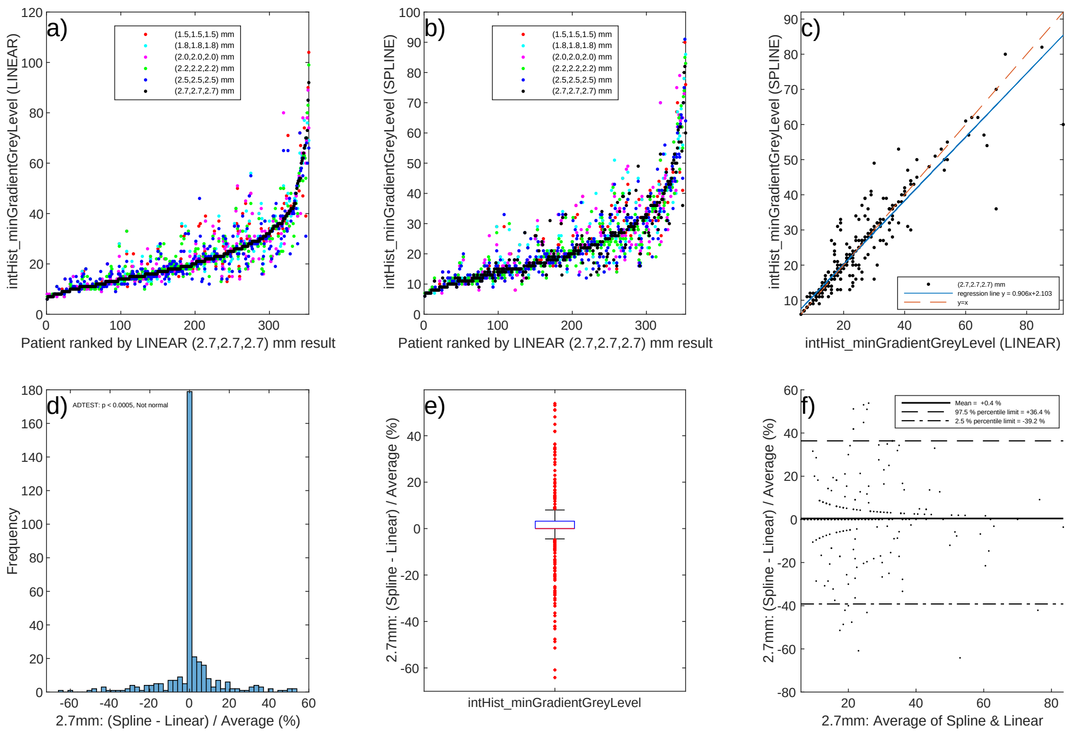

Figure 95: intHist-minGradientGreyLevel

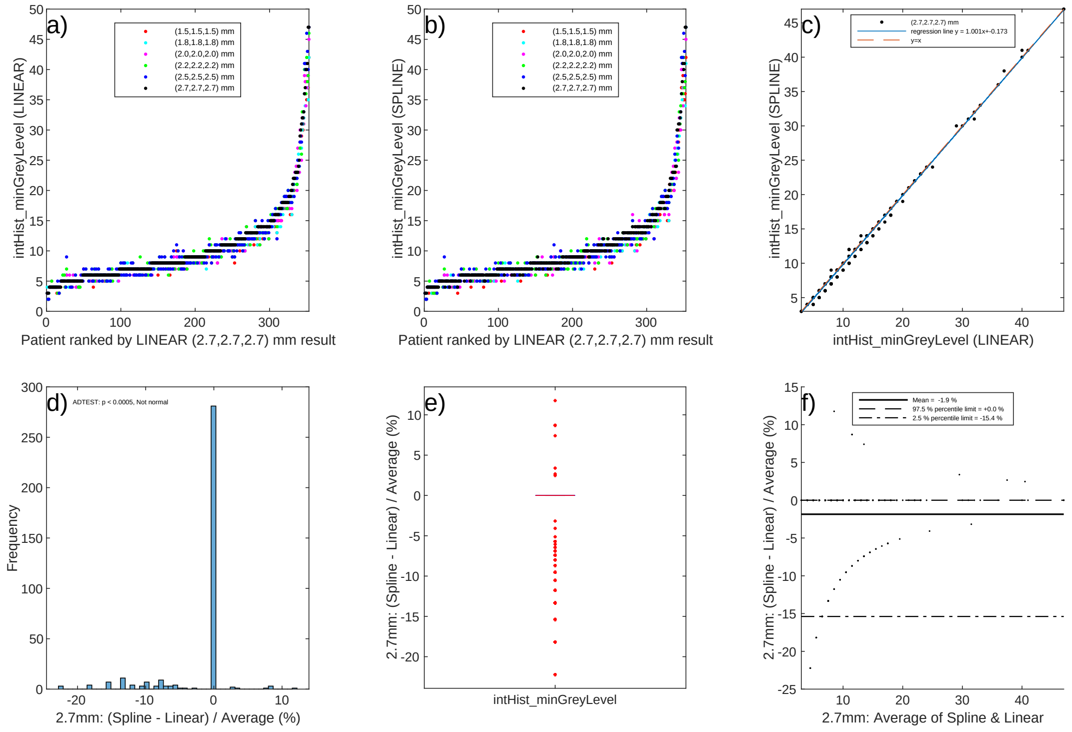

Figure 96: intHist-minGreyLevel

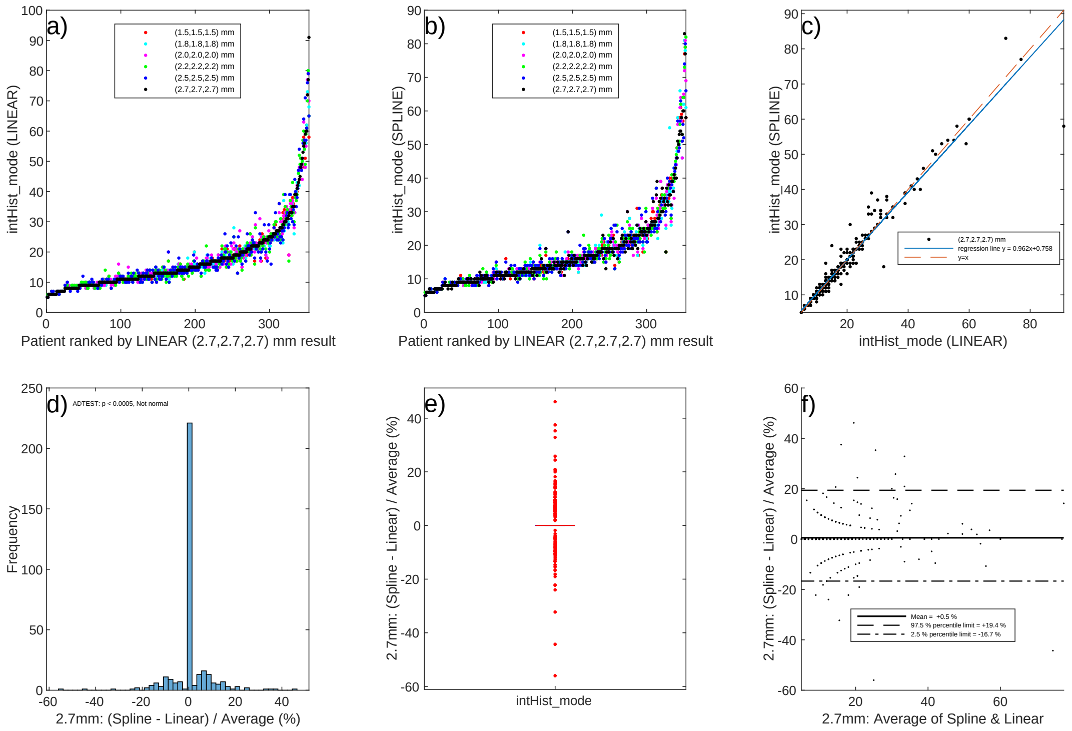

Figure 97: intHist-mode

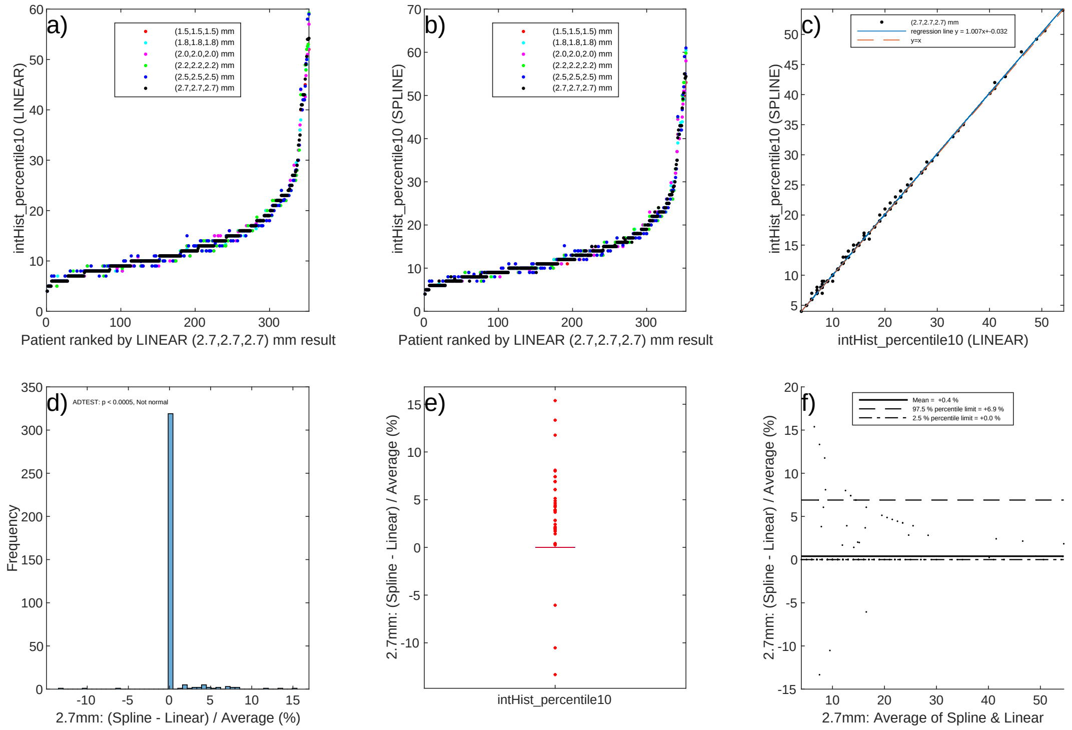

Figure 98: intHist-percentile10

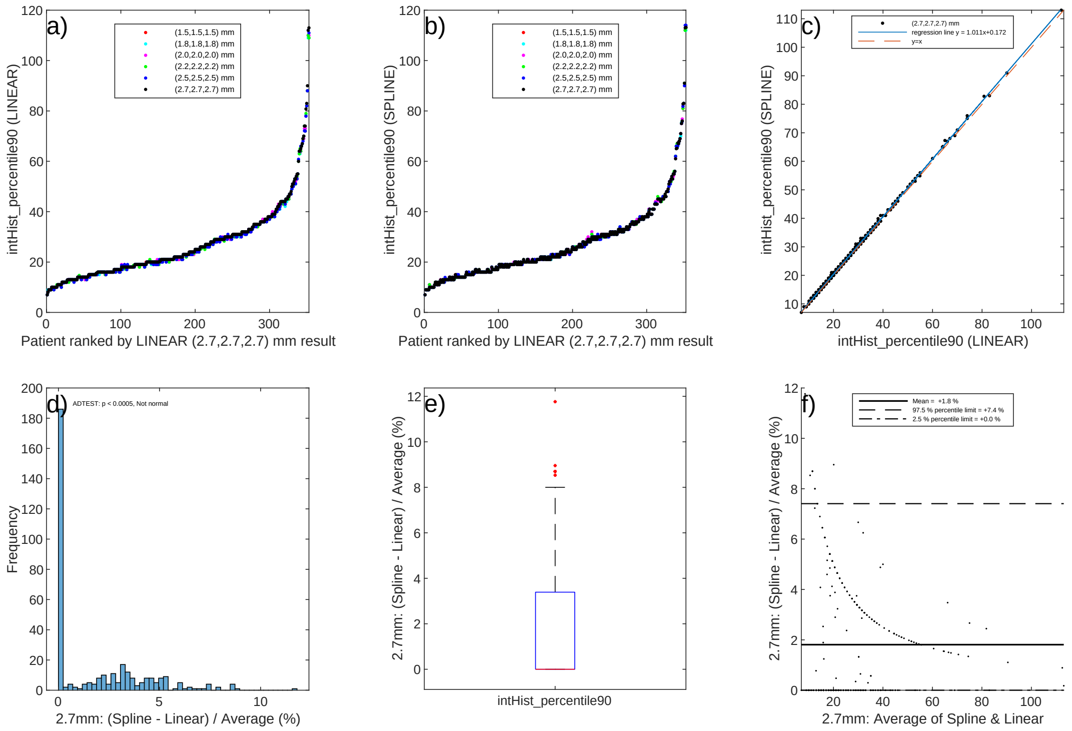

Figure 99: intHist-percentile90

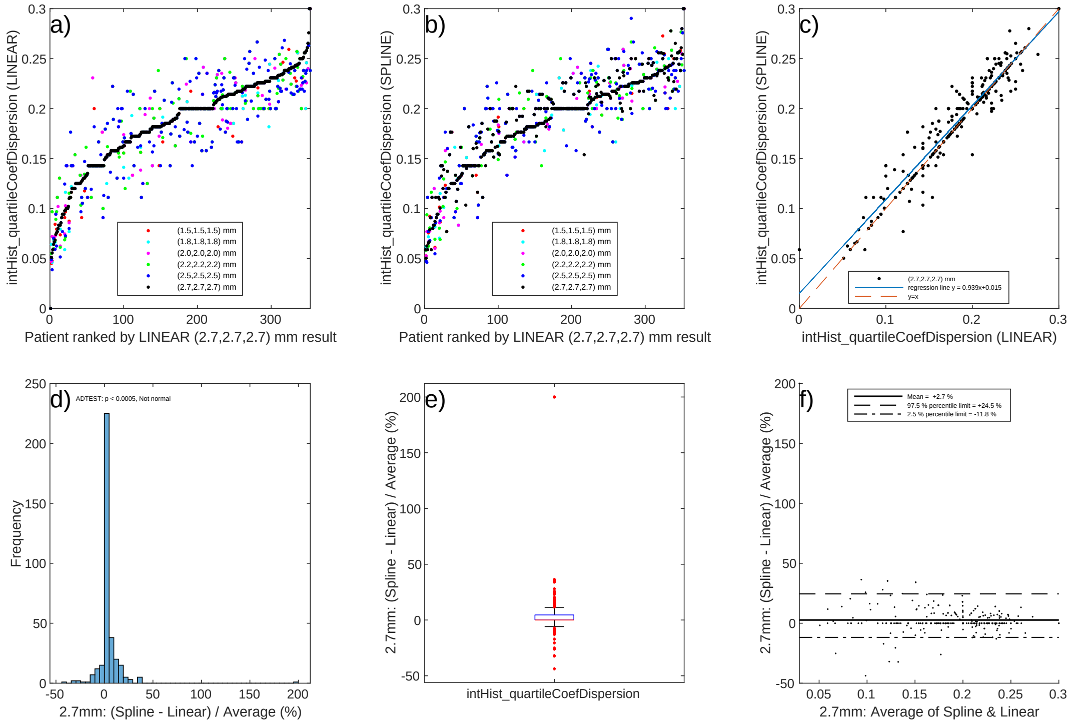

Figure 100: intHist-quartileCoefDispersion

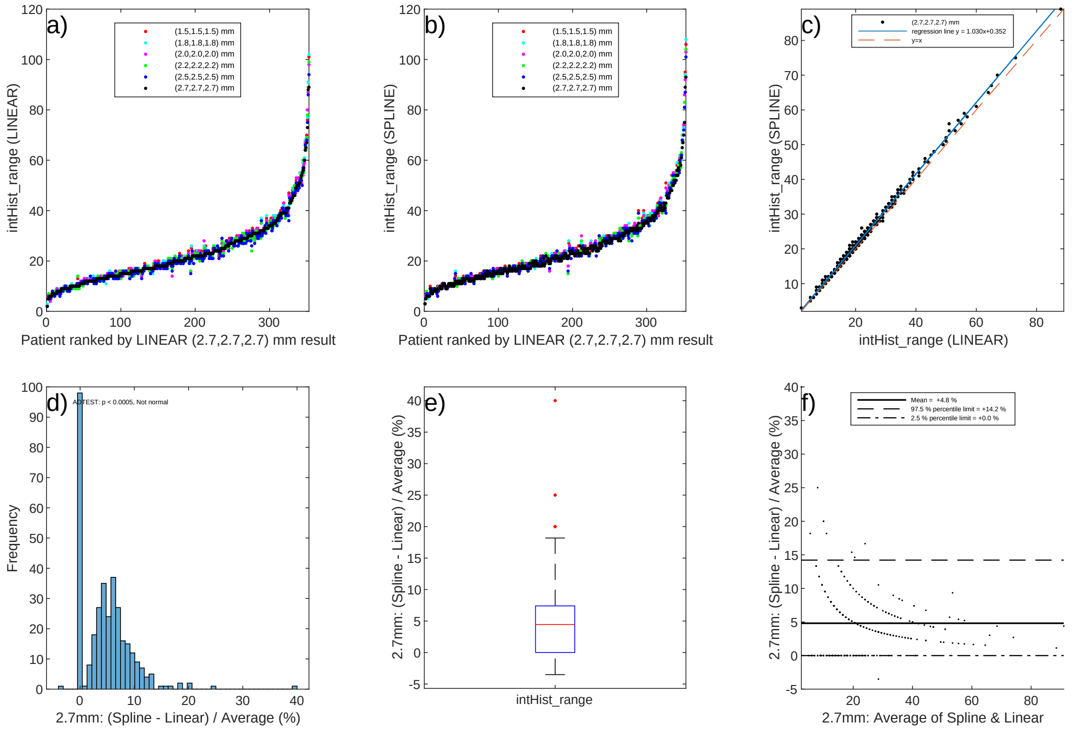

Figure 101: intHist-range

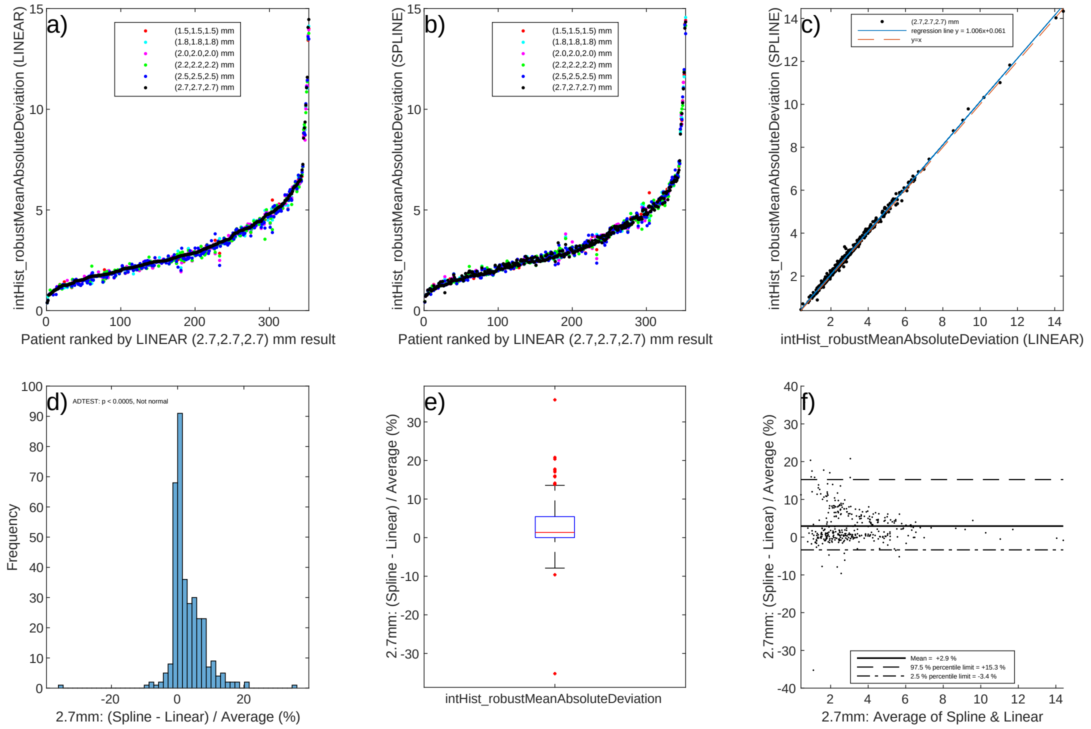

Figure 102: intHist-robustMeanAbsoluteDeviation

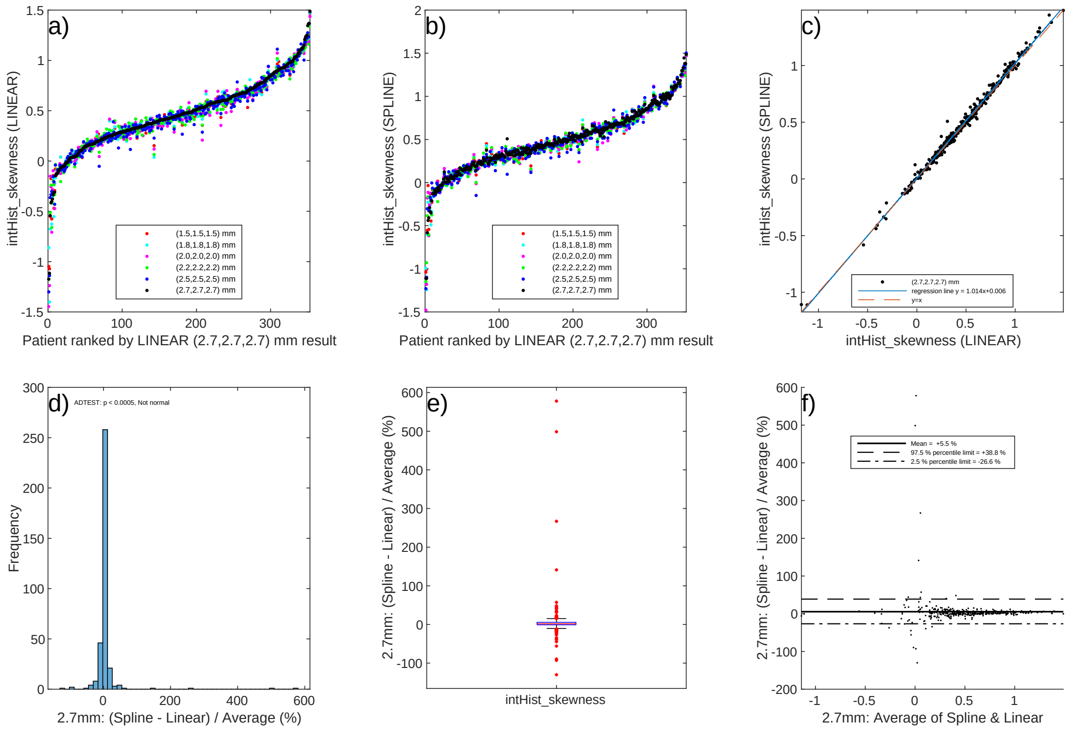

Figure 103: intHist-skewness

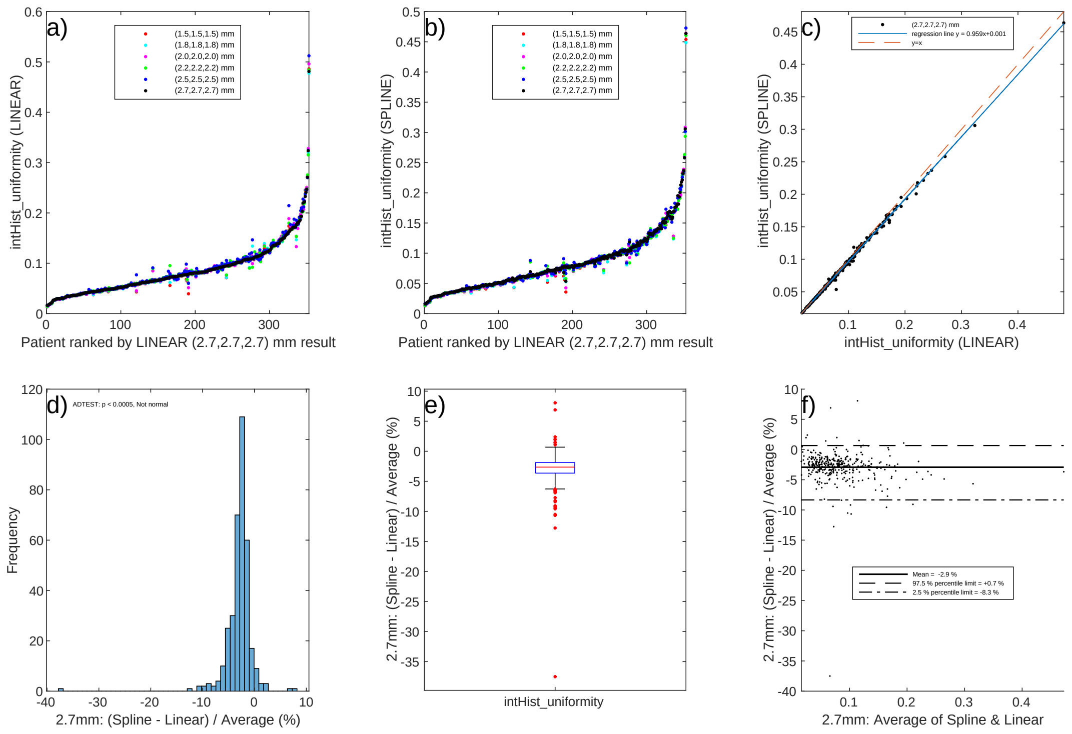

Figure 104: intHist-uniformity

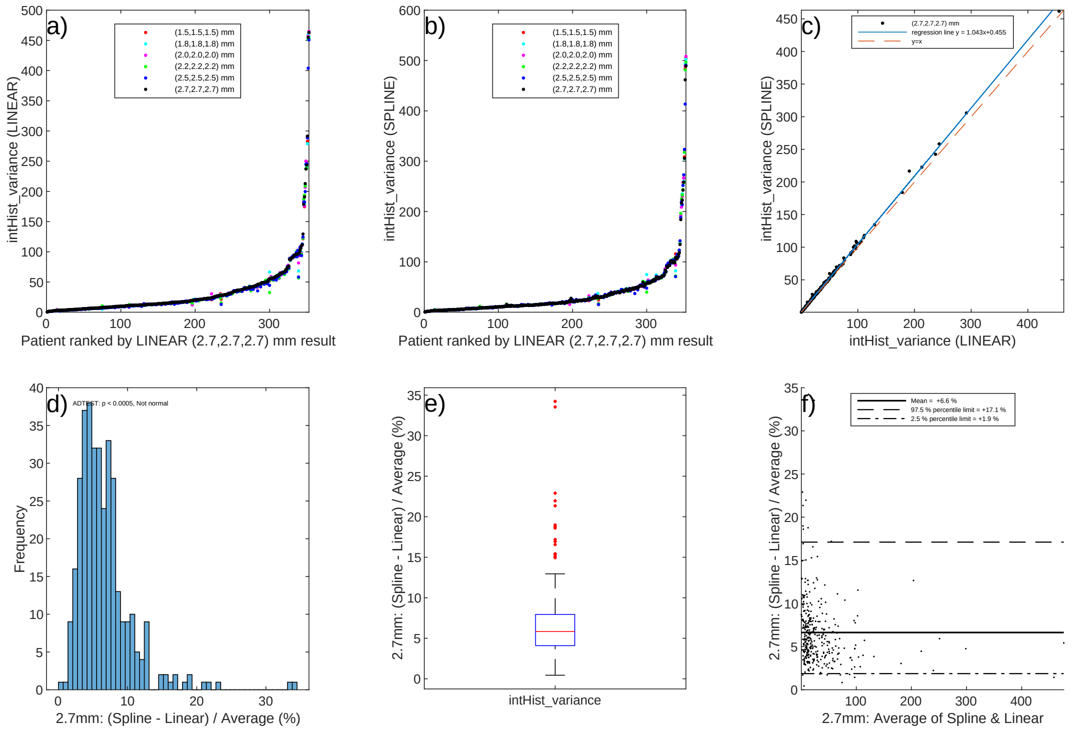

Figure 105: intHist-variance

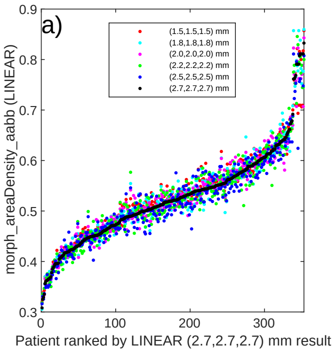

morph_areaDensity_aabb feature values only depend on mask and not the scan values.

Masks are always interpolated linearly.

Figure 106: morph-areaDensity-aabb

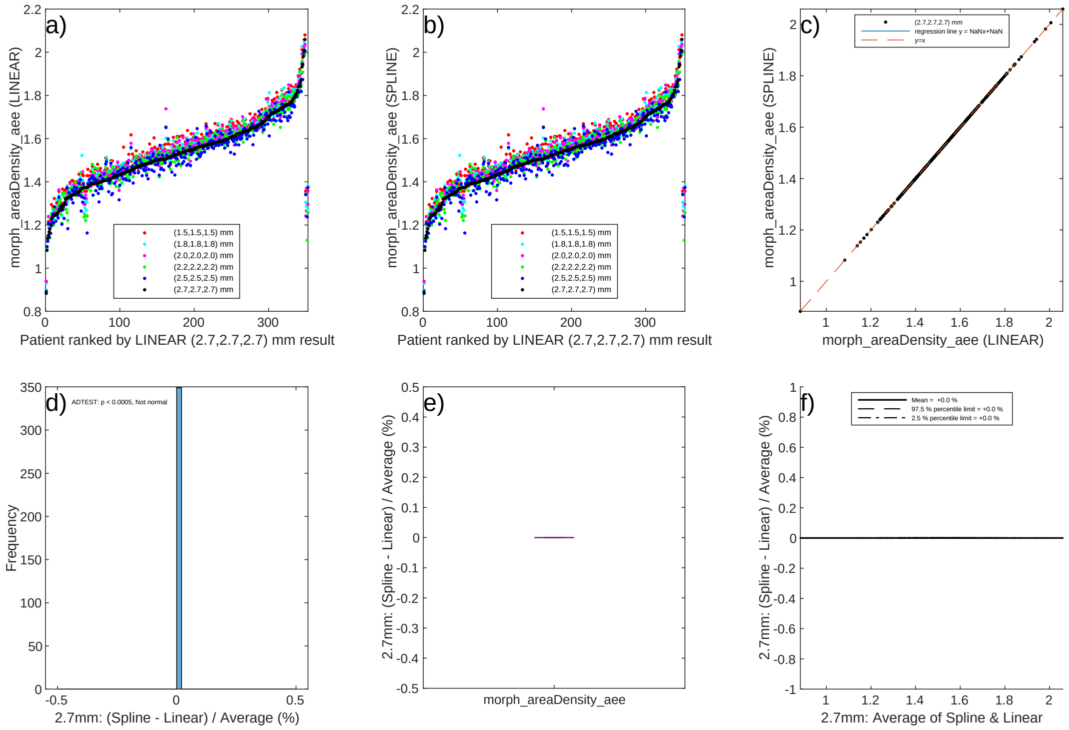

Figure 107: morph-areaDensity-aee

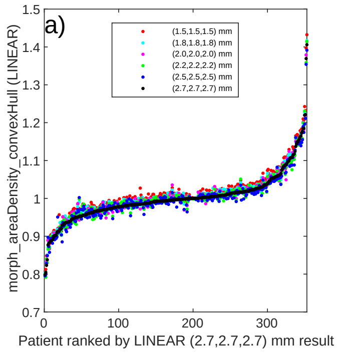

morph_areaDensity_convexHull feature values only depend on mask and not the scan values.

Masks are always interpolated linearly.

Figure 108: morph-areaDensity-convexHull

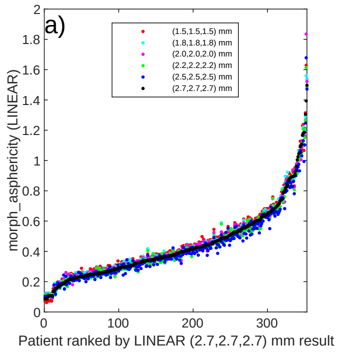

morph_asphericity feature values only depend on mask and not the scan values.

Masks are always interpolated linearly.

Figure 109: morph-asphericity

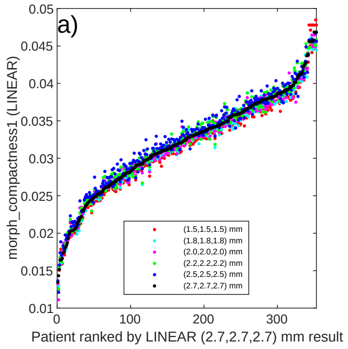

morph_compactness1 feature values only depend on mask and not the scan values.

Masks are always interpolated linearly.

Figure 110: morph-compactness1

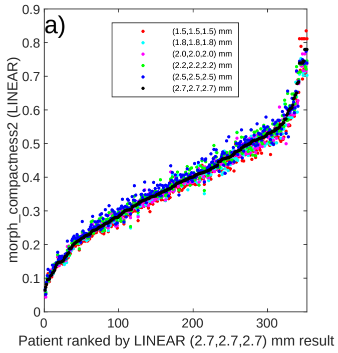

morph_compactness2 feature values only depend on mask and not the scan values.

Masks are always interpolated linearly.

Figure 111: morph-compactness2

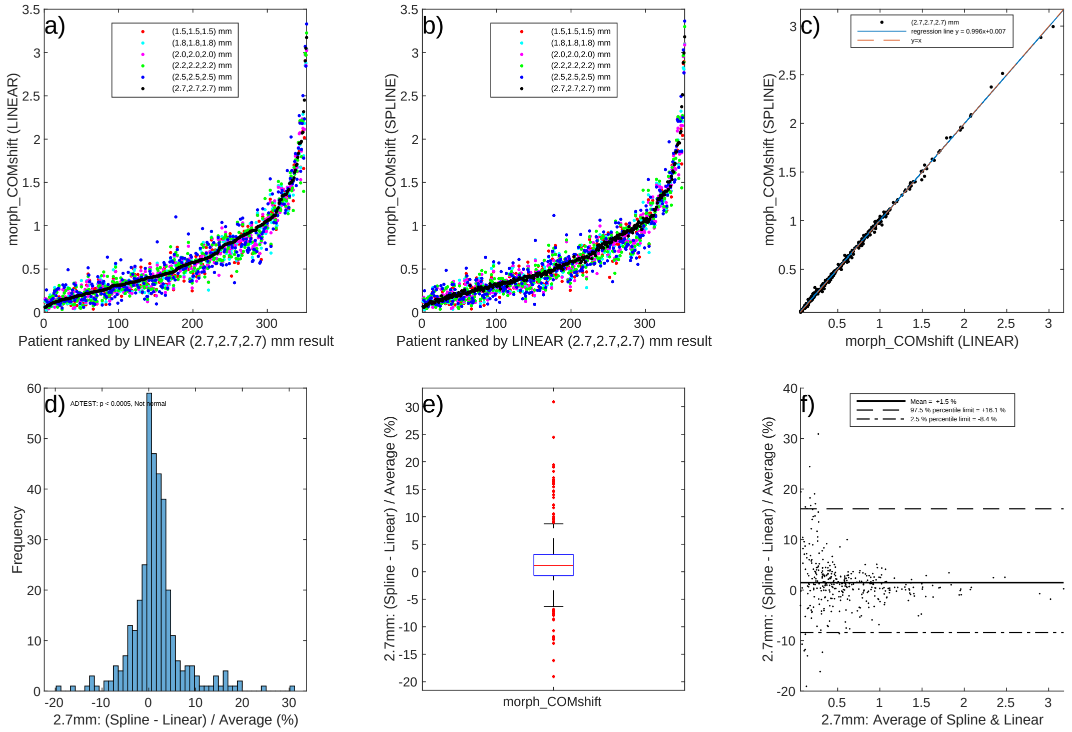

Figure 112: morph-COMshift

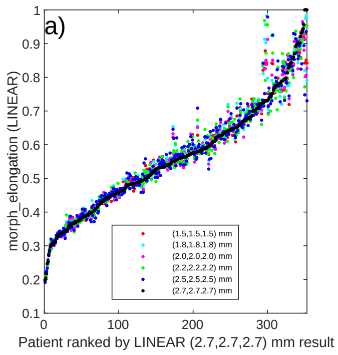

morph_elongation feature values only depend on mask and not the scan values.

Masks are always interpolated linearly.

Figure 113: morph-elongation

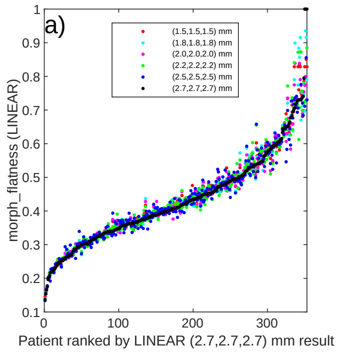

morph_flatness feature values only depend on mask and not the scan values.

Masks are always interpolated linearly.

Figure 114: morph-flatness

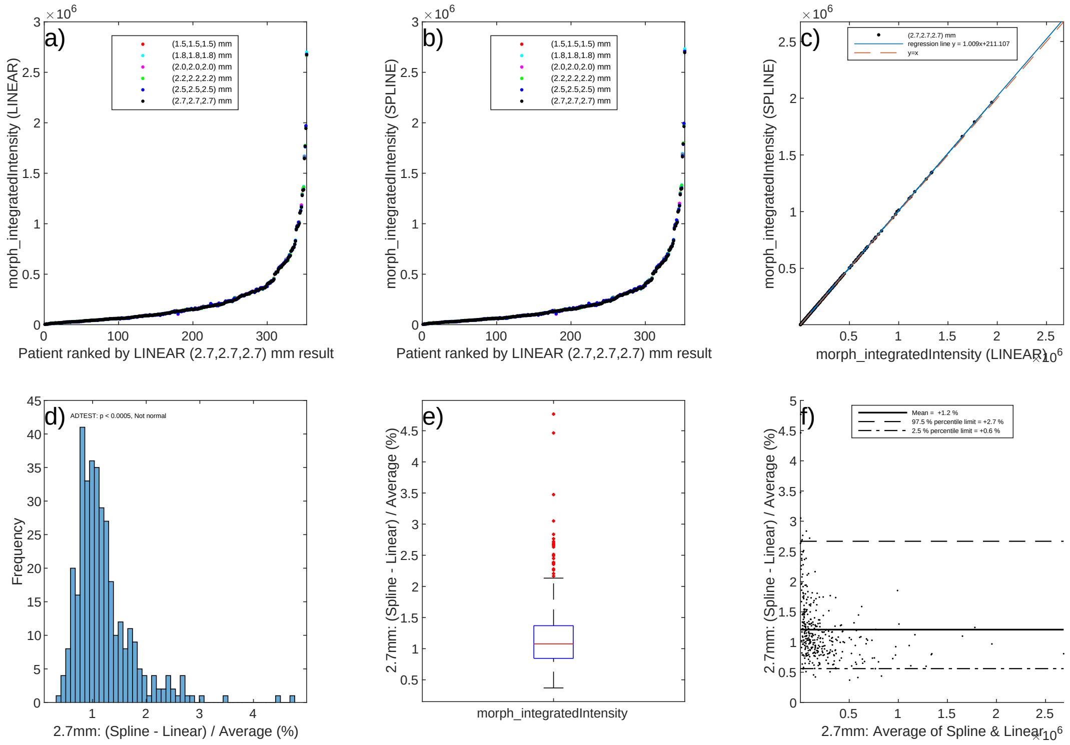

Figure 115: morph-integratedIntensity

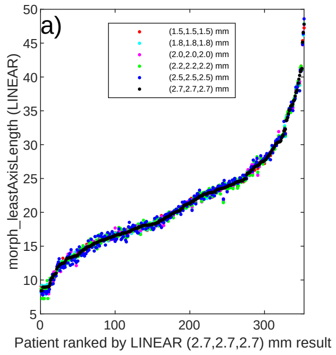

morph_leastAxisLength feature values only depend on mask and not the scan values.

Masks are always interpolated linearly.

Figure 116: morph-leastAxisLength

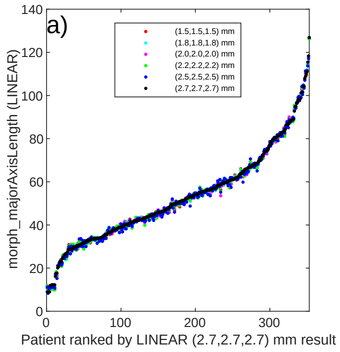

morph_majorAxisLength feature values only depend on mask and not the scan values.

Masks are always interpolated linearly.

Figure 117: morph-majorAxisLength

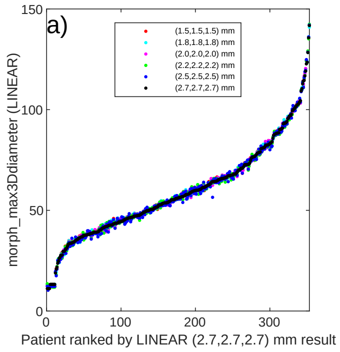

morph_max3Ddiameter feature values only depend on mask and not the scan values.

Masks are always interpolated linearly.

Figure 118: morph-max3Ddiameter

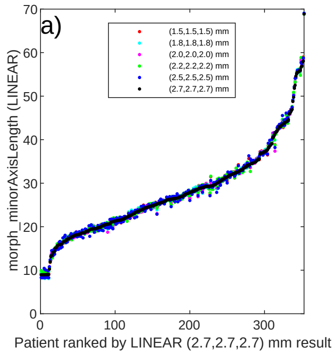

morph_minorAxisLength feature values only depend on mask and not the scan values.

Masks are always interpolated linearly.

Figure 119: morph-minorAxisLength

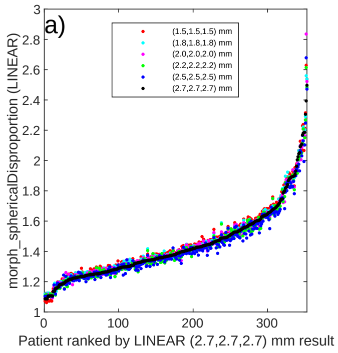

morph_sphericalDisproportion feature values only depend on mask and not the scan values.

Masks are always interpolated linearly.

Figure 120: morph-sphericalDisproportion

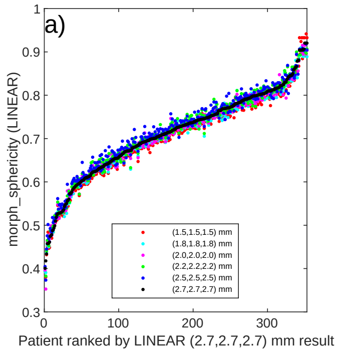

morph_sphericity feature values only depend on mask and not the scan values.

Masks are always interpolated linearly.

Figure 121: morph-sphericity

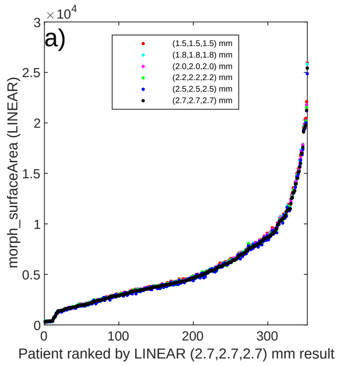

morph_surfaceArea feature values only depend on mask and not the scan values.

Masks are always interpolated linearly.

Figure 122: morph-surfaceArea

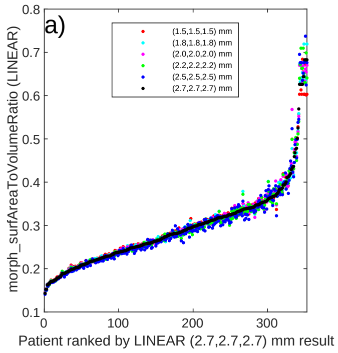

morph_surfAreaToVolumeRatio feature values only depend on mask and not the scan values.

Masks are always interpolated linearly.

Figure 123: morph-surfAreaToVolumeRatio

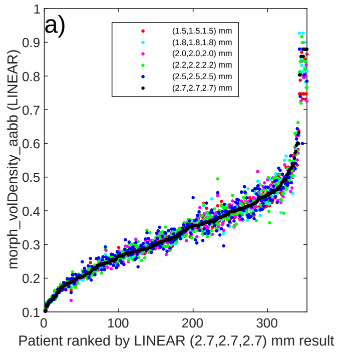

morph_volDensity_aabb feature values only depend on mask and not the scan values.

Masks are always interpolated linearly.

Figure 124: morph-volDensity-aabb

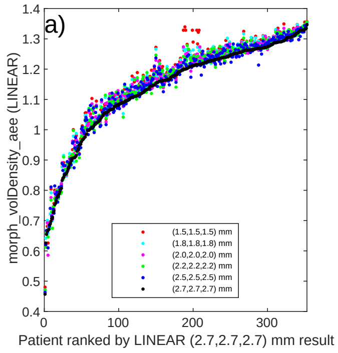

morph_volDensity_aee feature values only depend on mask and not the scan values.

Masks are always interpolated linearly.

Figure 125: morph-volDensity-aee

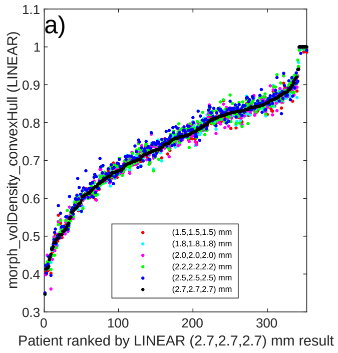

morph_volDensity_convexHull feature values only depend on mask and not the scan values.

Masks are always interpolated linearly.

Figure 126: morph-volDensity-convexHull

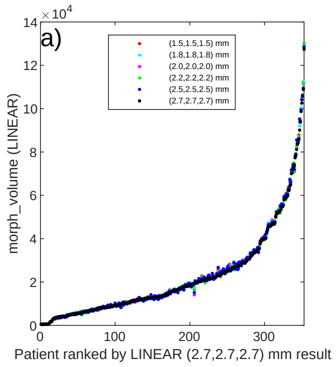

morph_volume feature values only depend on mask and not the scan values.

Masks are always interpolated linearly.

Figure 127: morph-volume

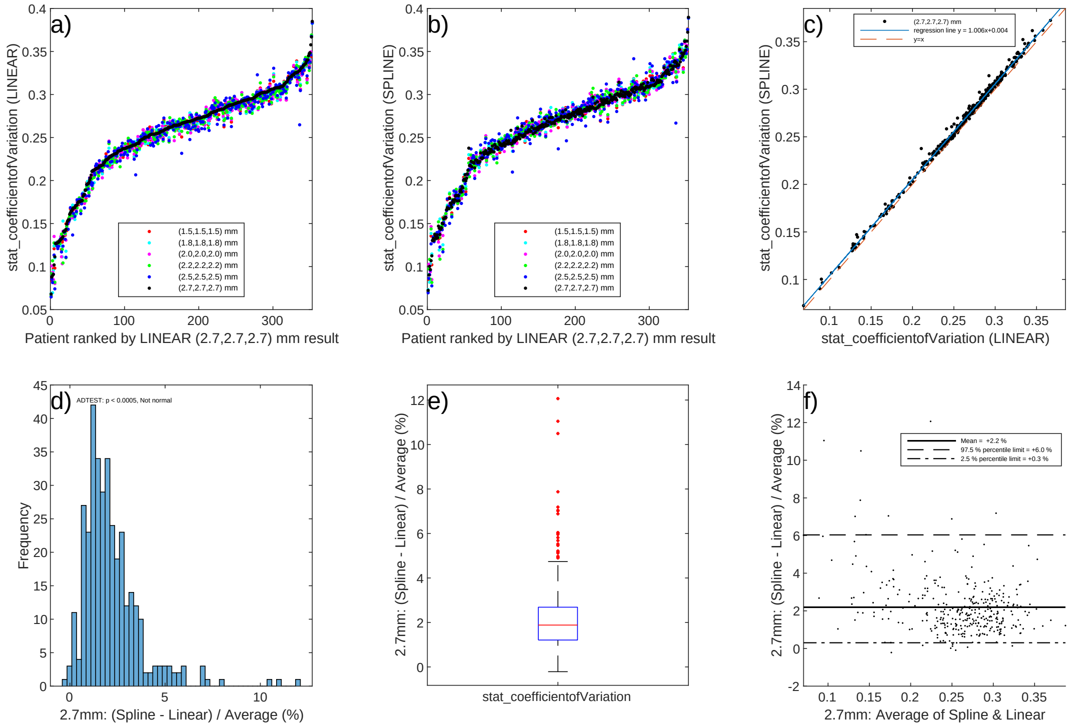

Figure 128: stat-coefficientofVariation

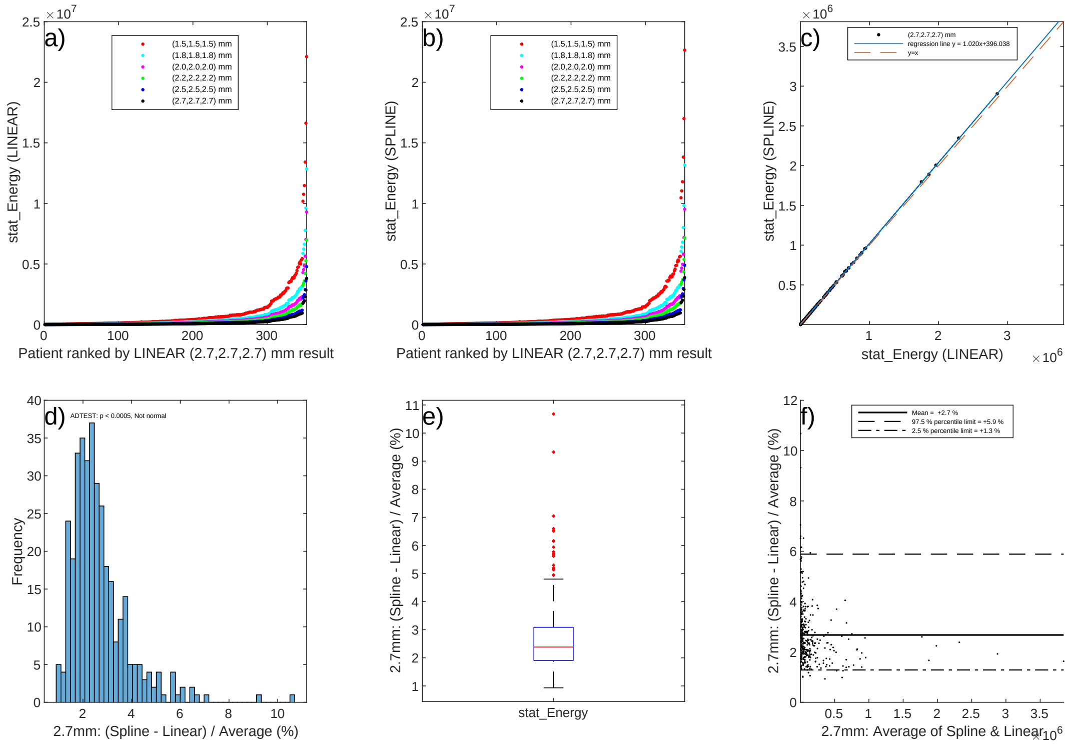

Figure 129: stat-Energy

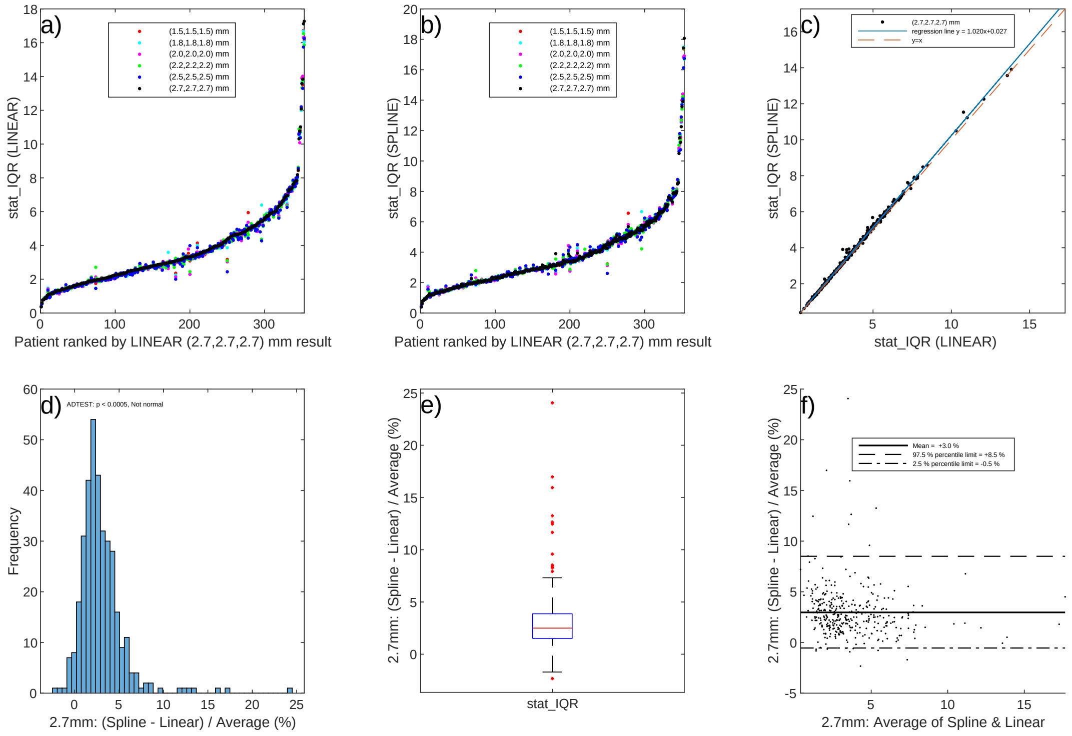

Figure 130: stat-IQR

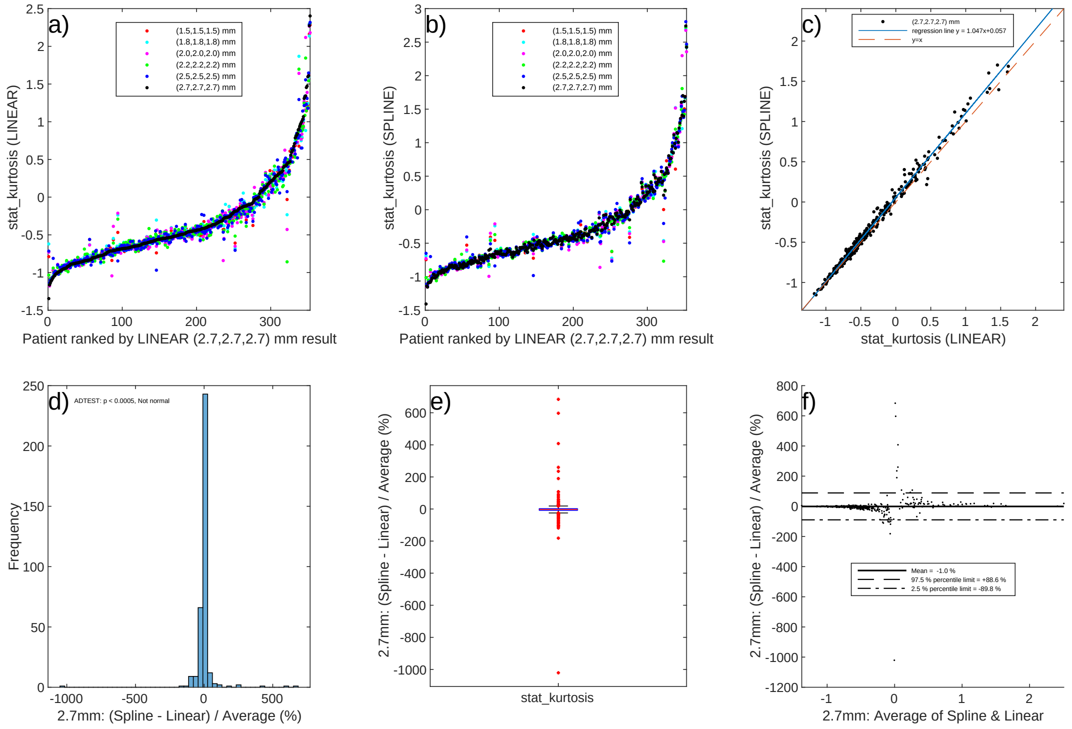

Figure 131: stat-kurtosis

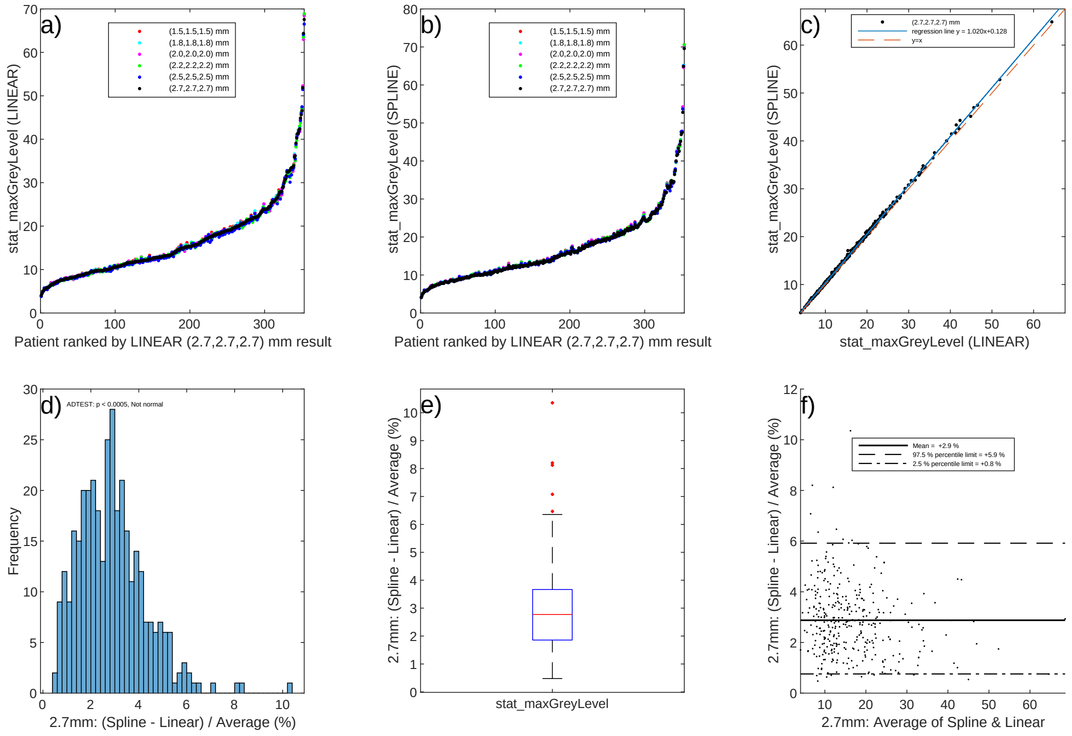

Figure 132: stat-maxGreyLevel

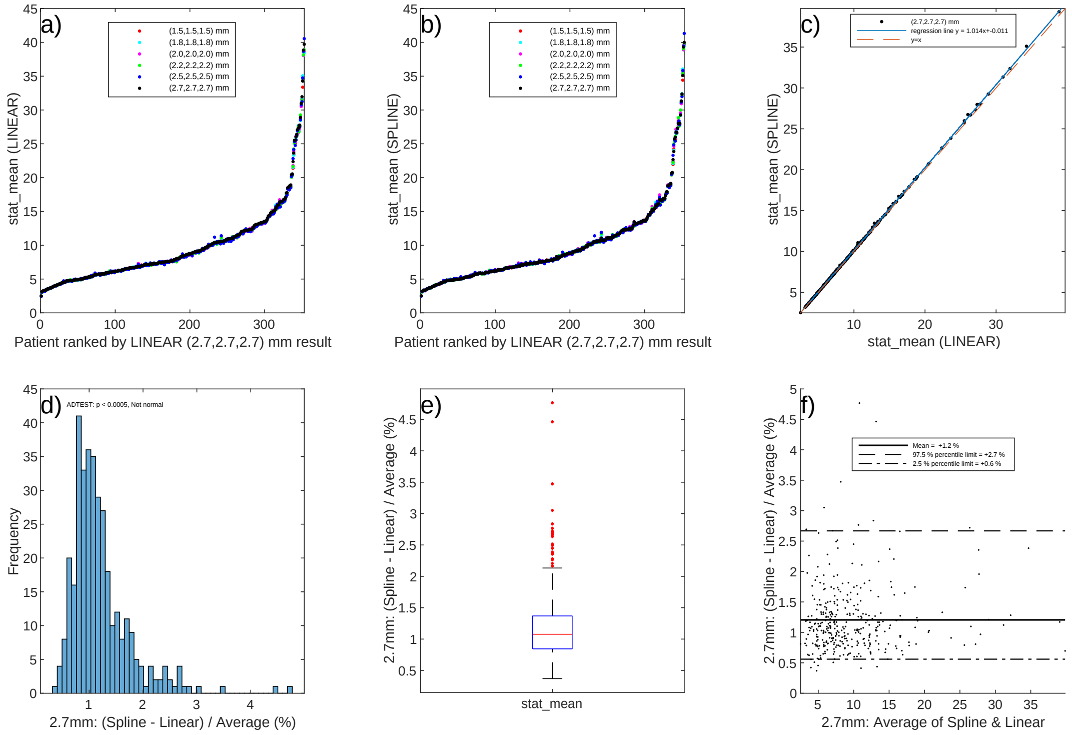

Figure 133: stat-mean

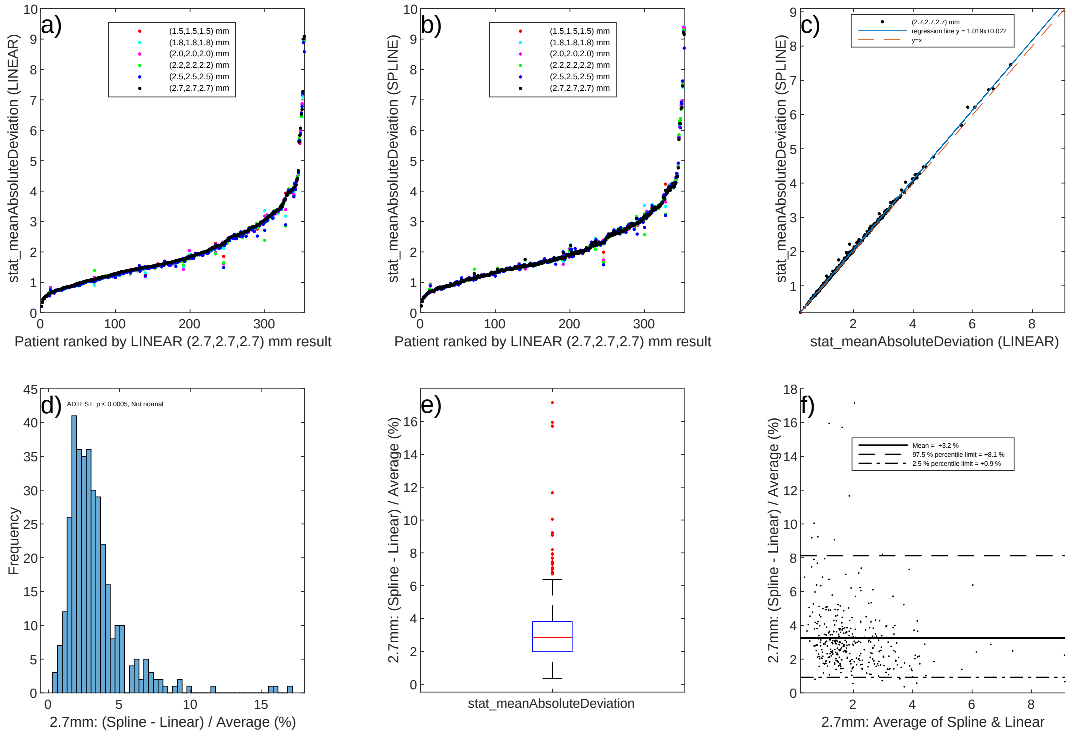

Figure 134: stat-meanAbsoluteDeviation

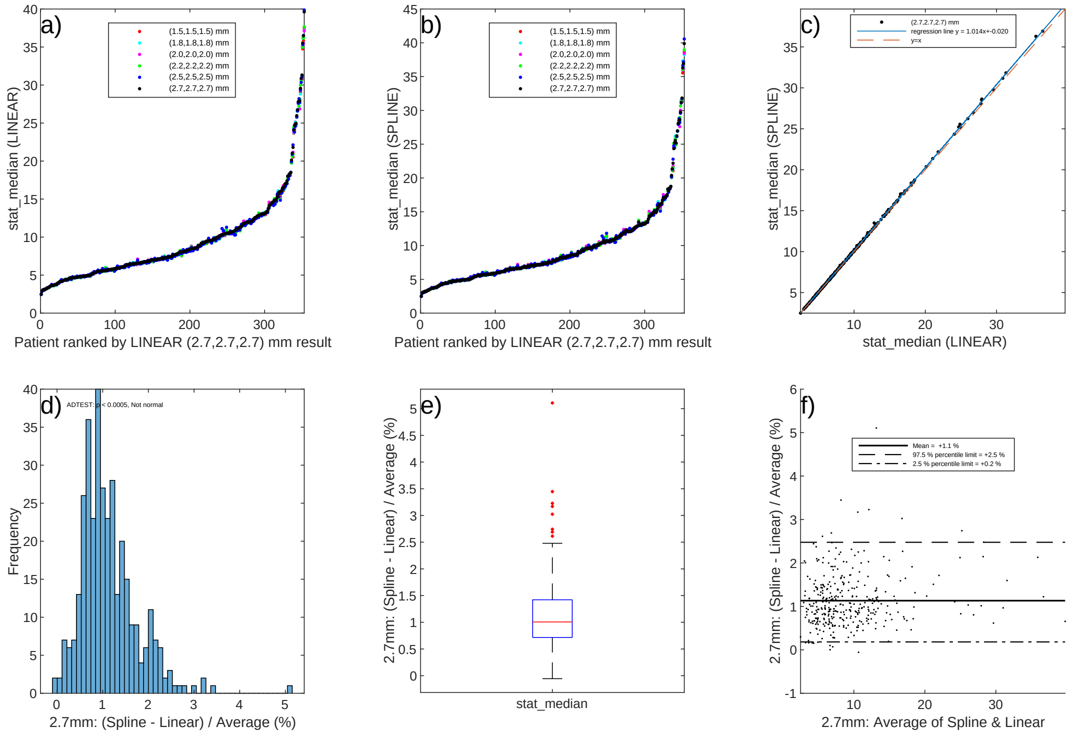

Figure 135: stat-median

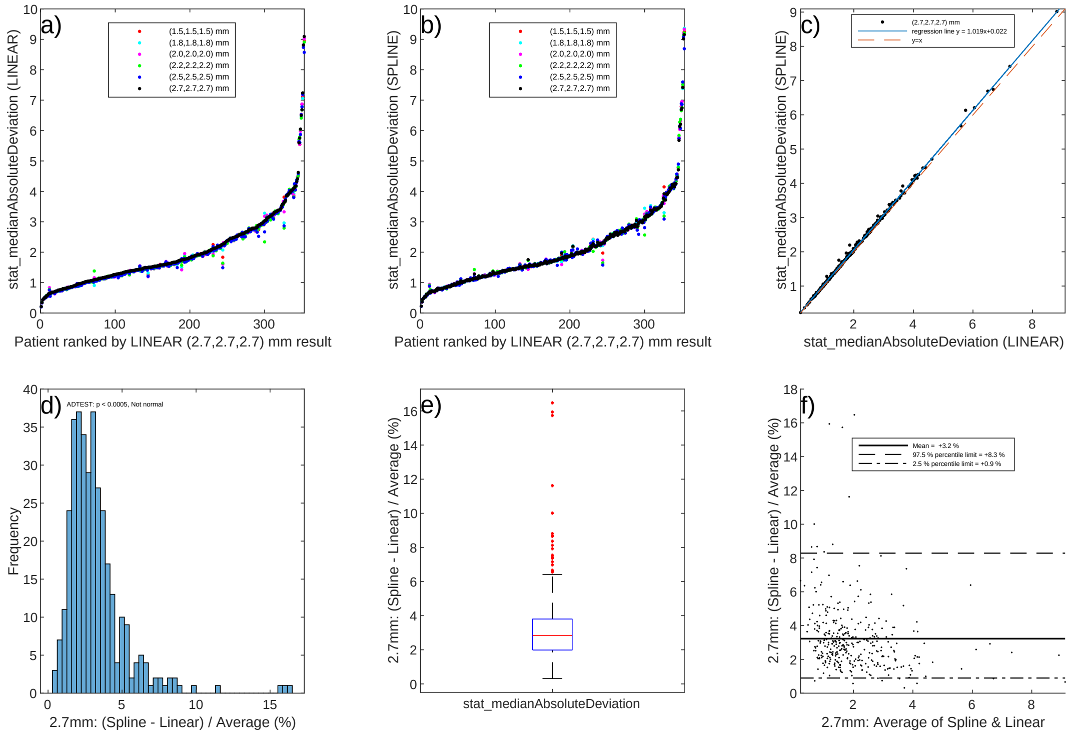

Figure 136: stat-medianAbsoluteDeviation

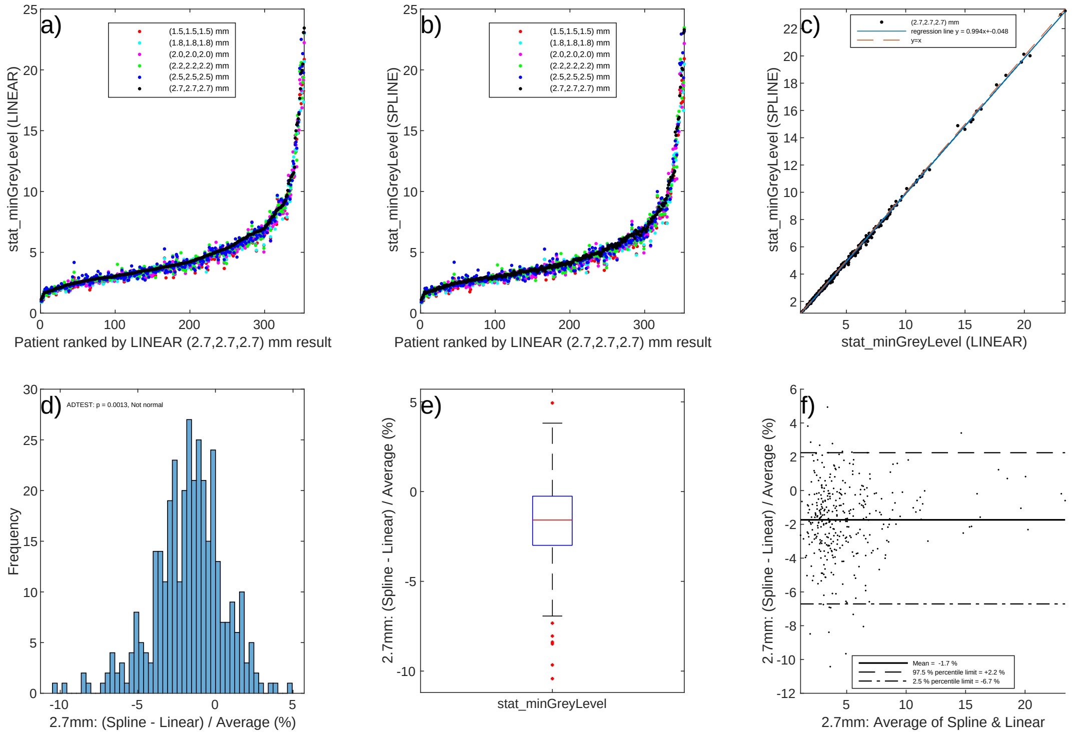

Figure 137: stat-minGreyLevel

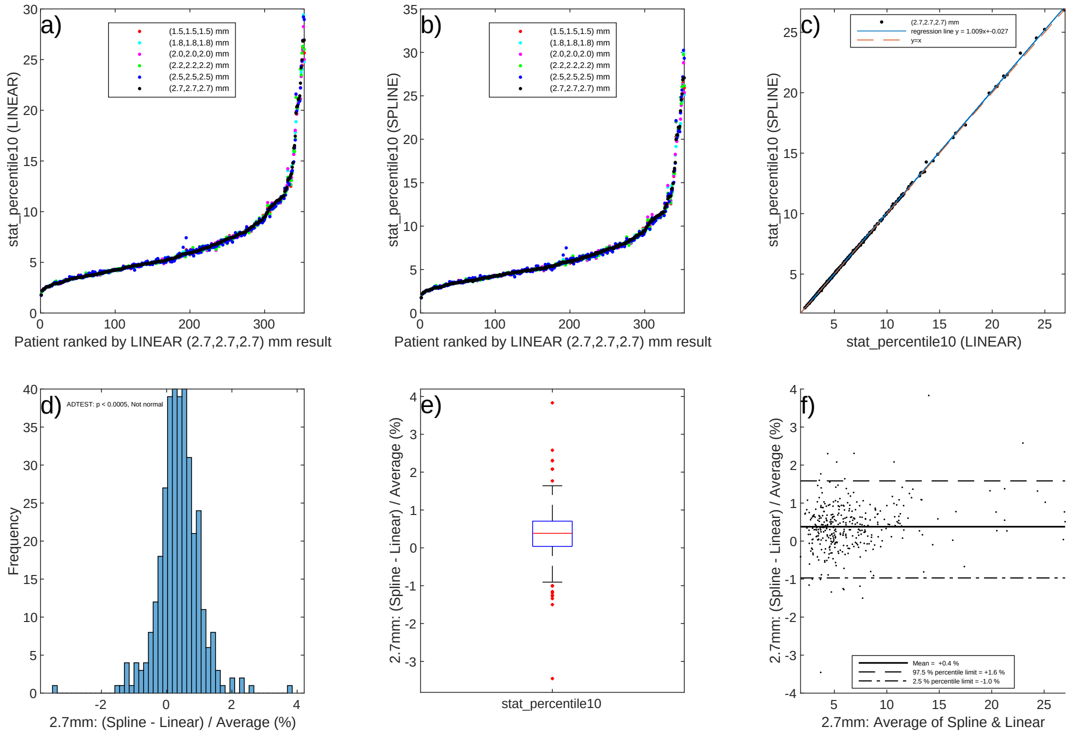

Figure 138: stat-percentile10

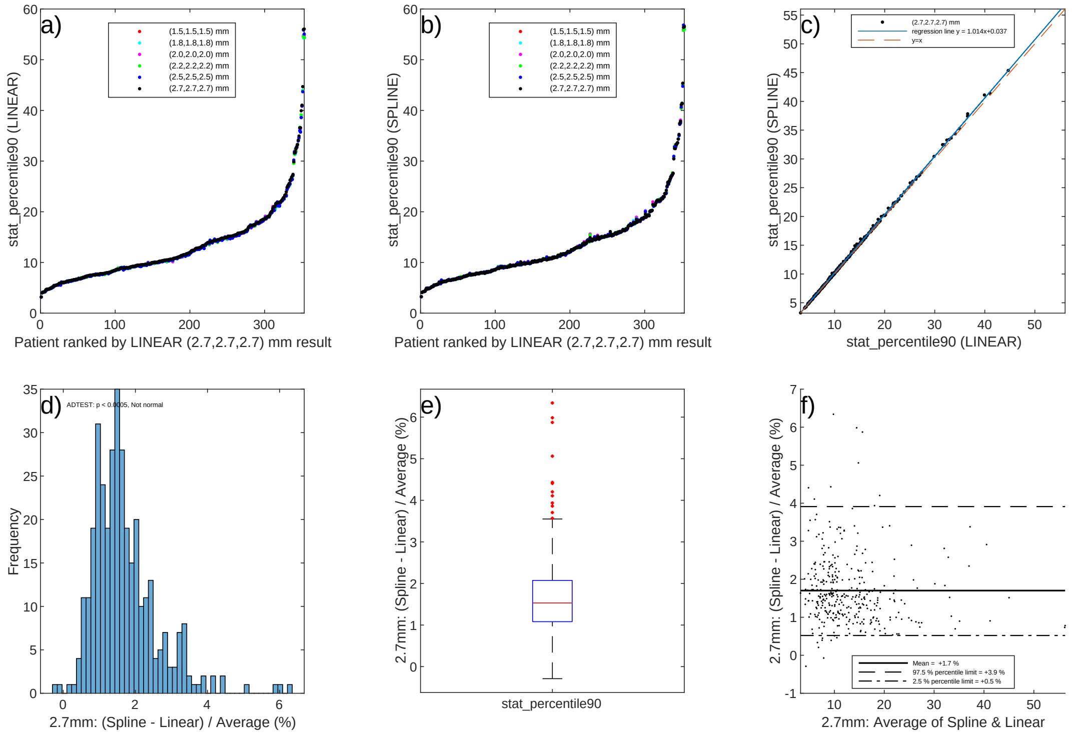

Figure 139: stat-percentile90

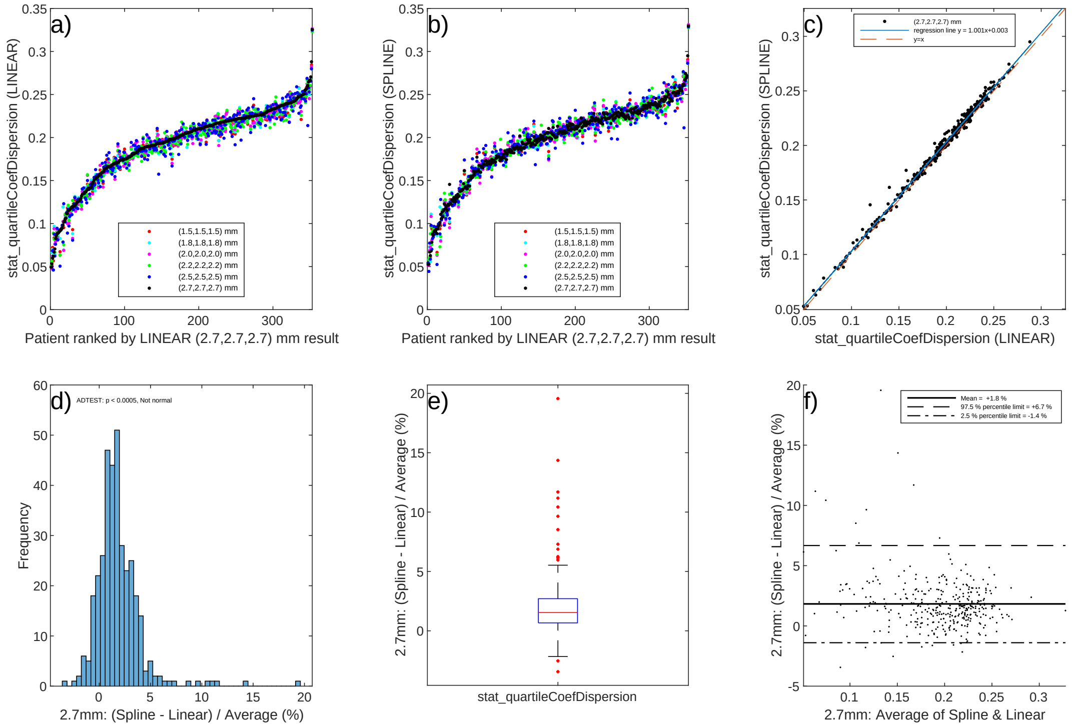

Figure 140: stat-quartileCoefDispersion

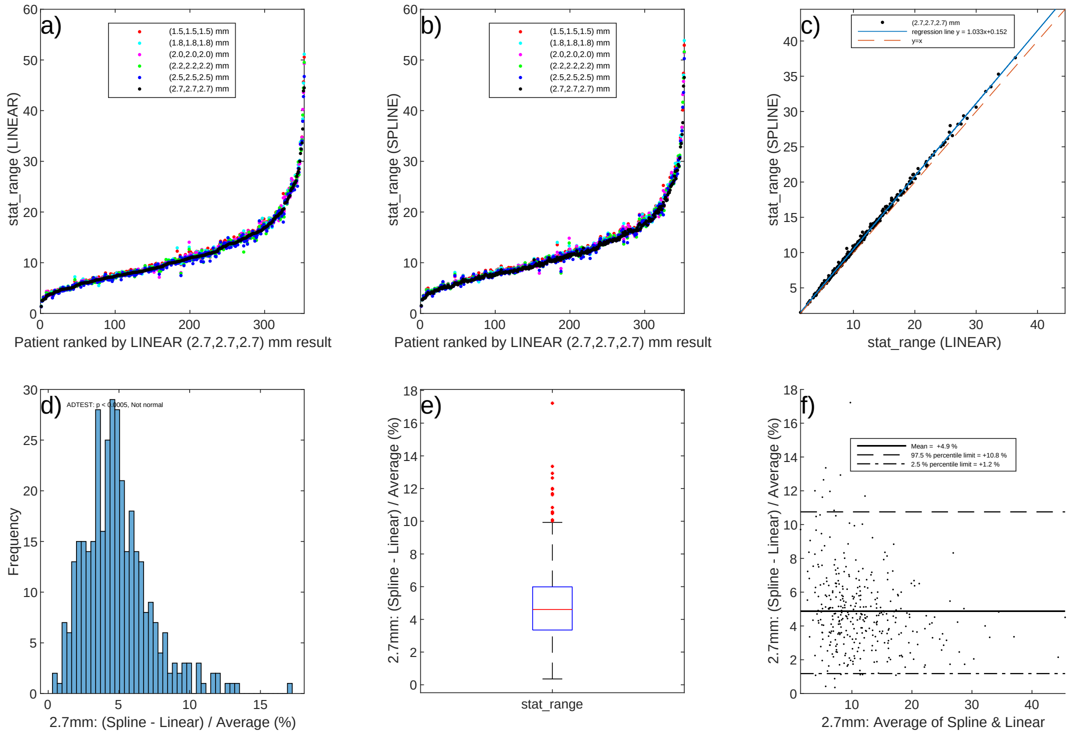

Figure 141: stat-range

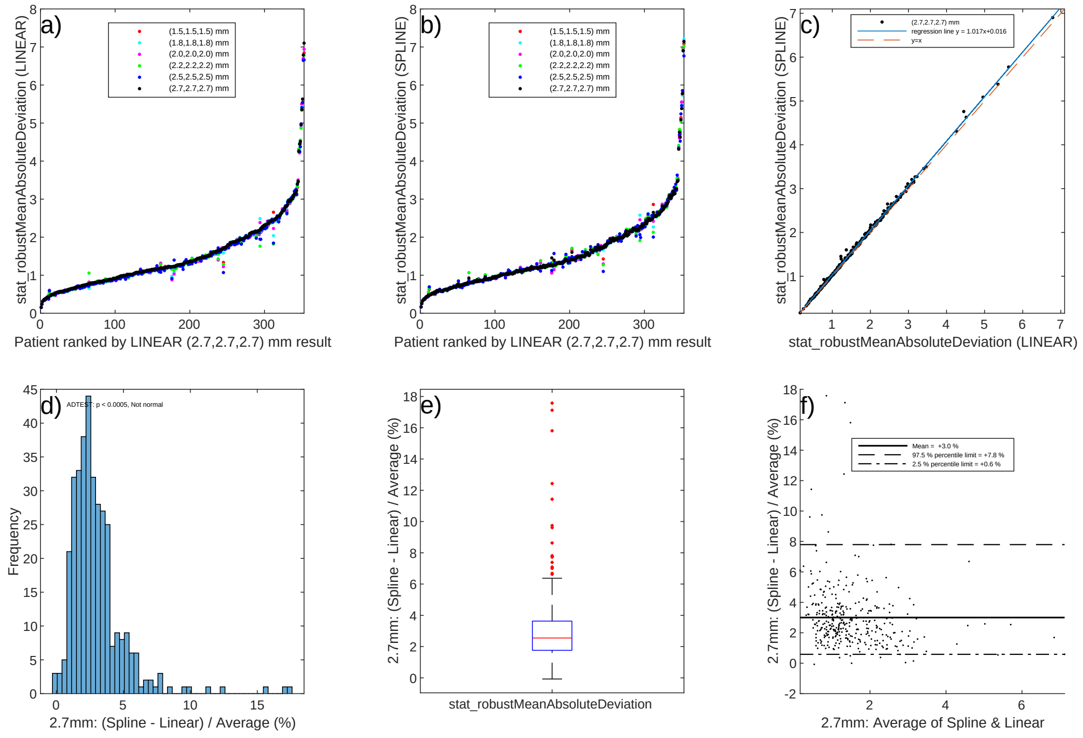

Figure 142: stat-robustMeanAbsoluteDeviation

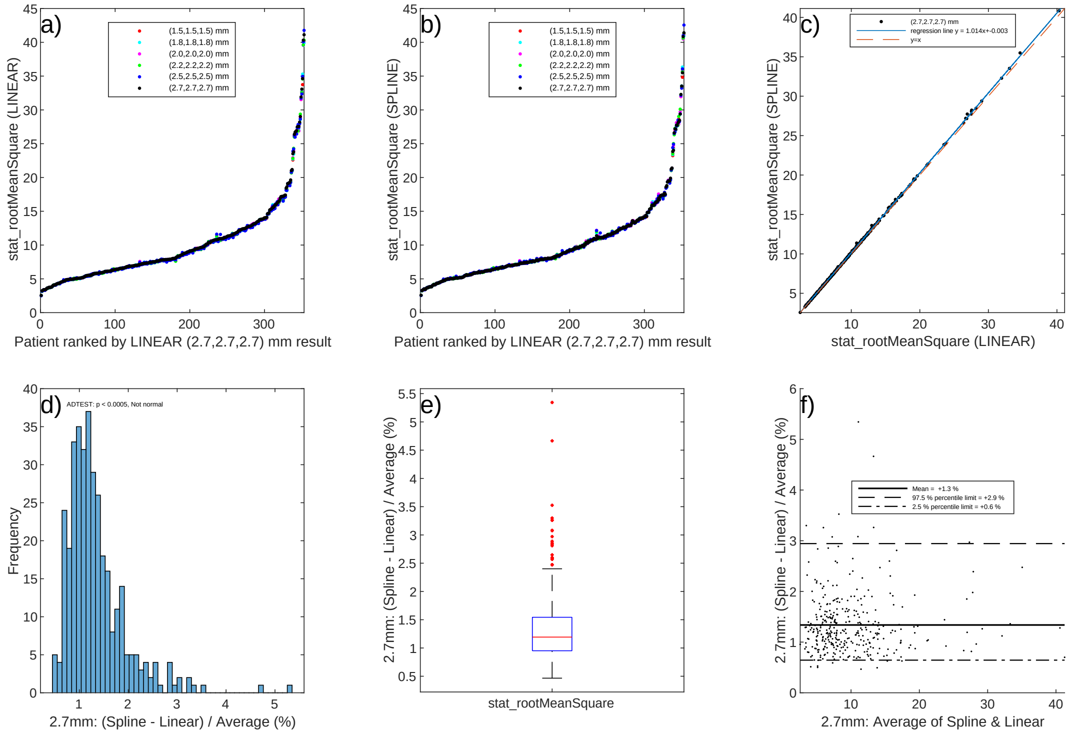

Figure 143: stat-rootMeanSquare

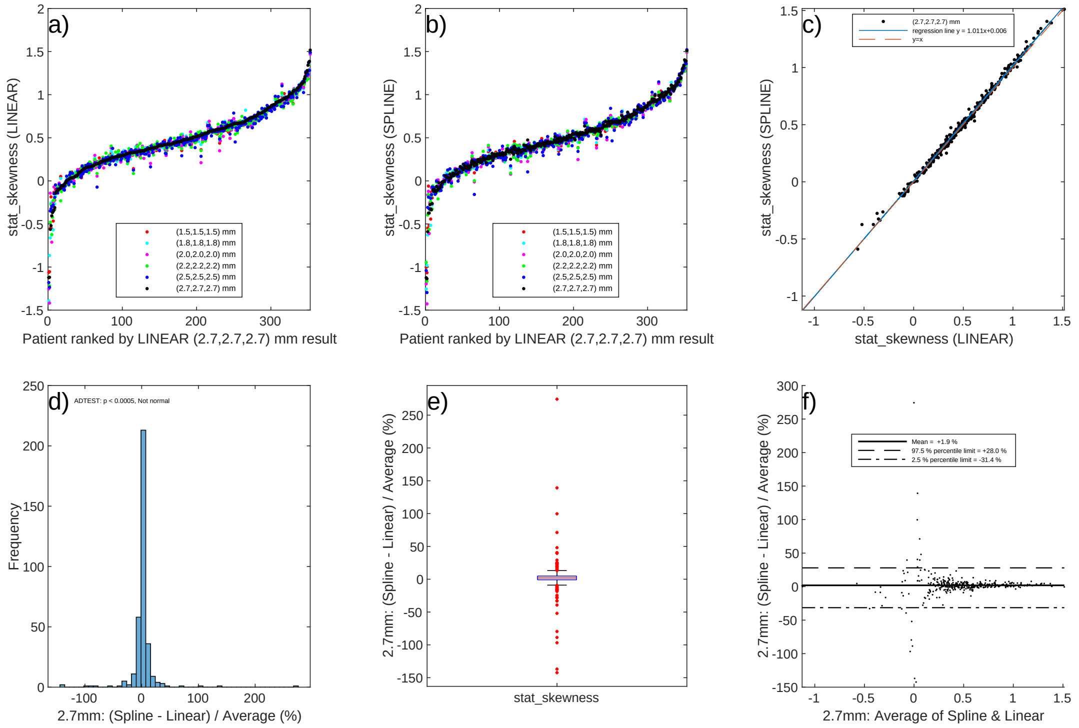

Figure 144: stat-skewness

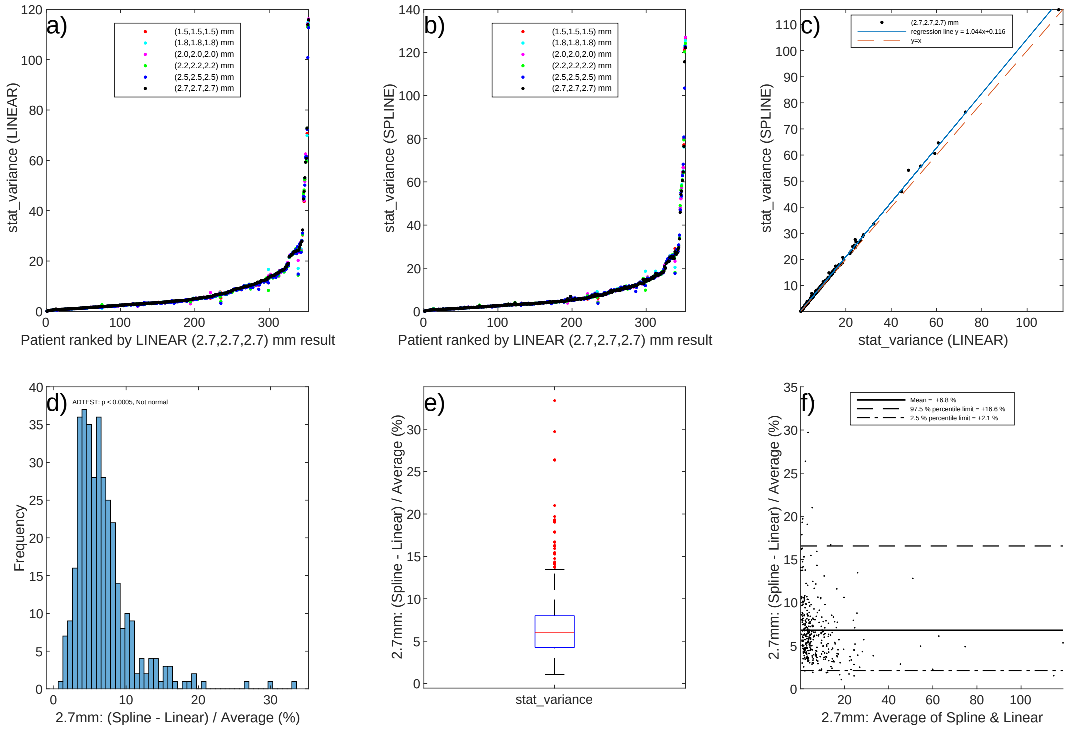

Figure 145: stat-variance

5 Modelling Interpolation response

5.1 Plot explanation

The 34 features marked as correctable due to systematic behaviour underwent the surface fitting method for correction as described in the methods section of the main document. The surfaces, developed using the testing cohort, were evaluated by rescaling feature values in the validation cohort. For each VOI, feature value and voxel size were plotted against the percentage change in feature value compared to the 2.7mm voxel dimension result. Surfaces were generated using the fit function from MATLAB's curve fitting toolbox (MathWorks, Natick, MA, USA). For VOIs with the 2.7mm voxel dimension, the percentage change in feature value compared to the 2.7mm result is clearly zero.

This process was demonstrated in the main manuscript in Fig.4 for the feature glrl feature run length Non uniformity. Below we plot the same visualisations for all 34 features that we attempted to correct.

For each figure, plot a) is the testing dataset feature values and voxel sizes plotted against the percentage change in feature values. Plot b) is a ranked plot of the validation dataset for a feature before applying a correction. Plot c) is the validation dataset after applying the correction based on equation (1) in the main manuscript.

5.2 Surface fit corrections

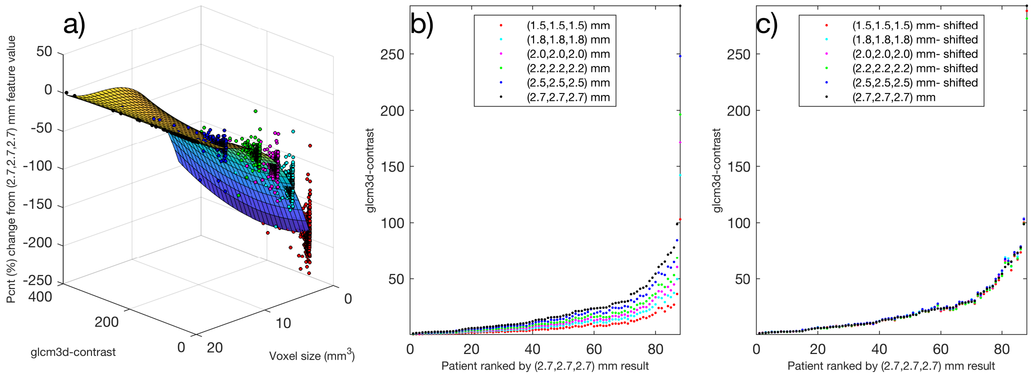

Figure 146: Modelling response: glcm3d-contrast

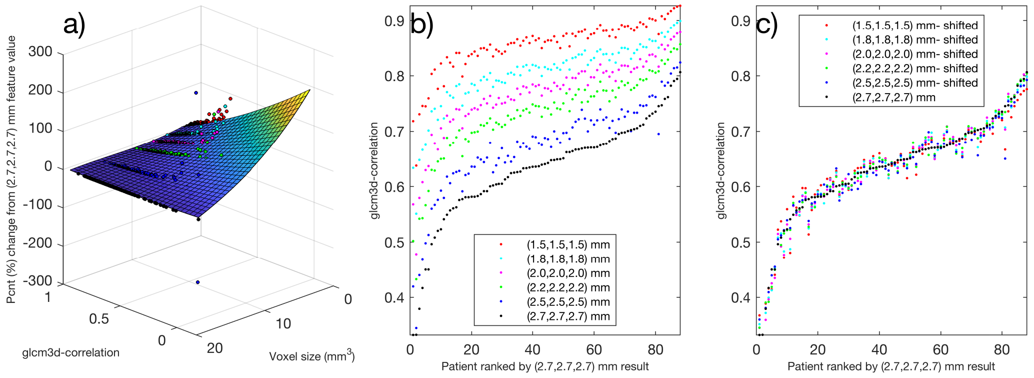

Figure 147: Modelling response: glcm3d-correlation

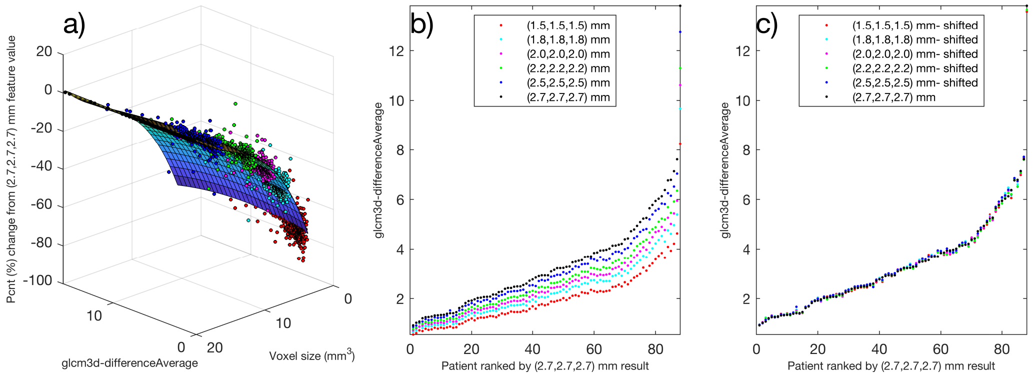

Figure 148: Modelling response: glcm3d-differenceAverage

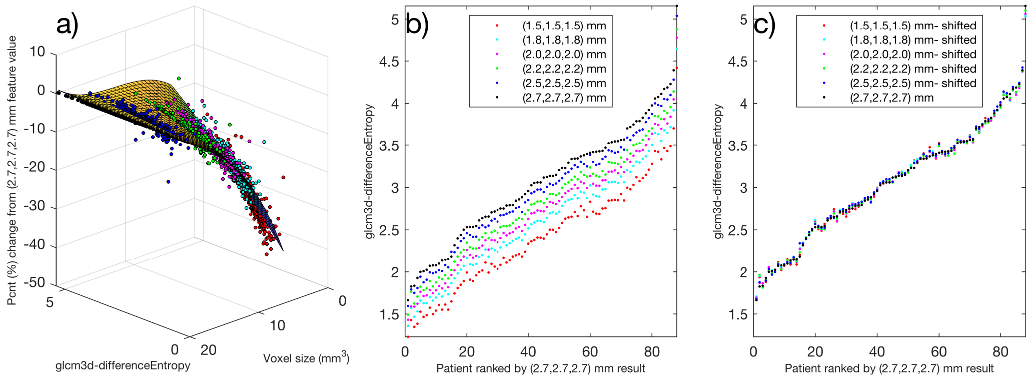

Figure 149: Modelling response: glcm3d-differenceEntropy

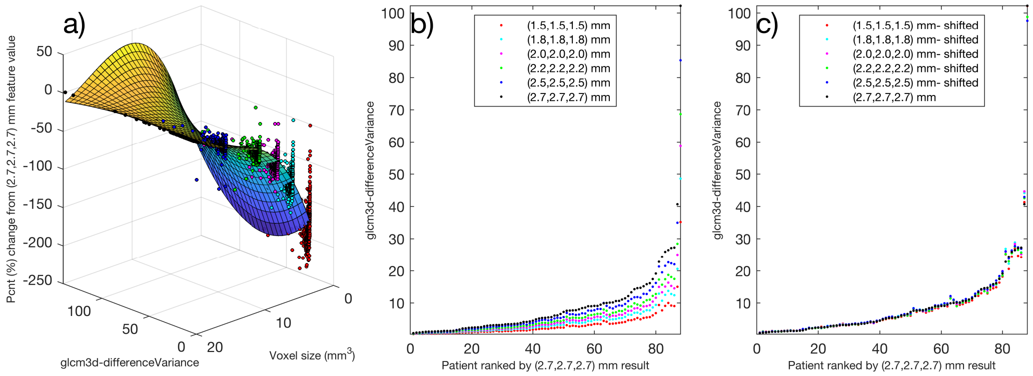

Figure 150: Modelling response: glcm3d-differenceVariance

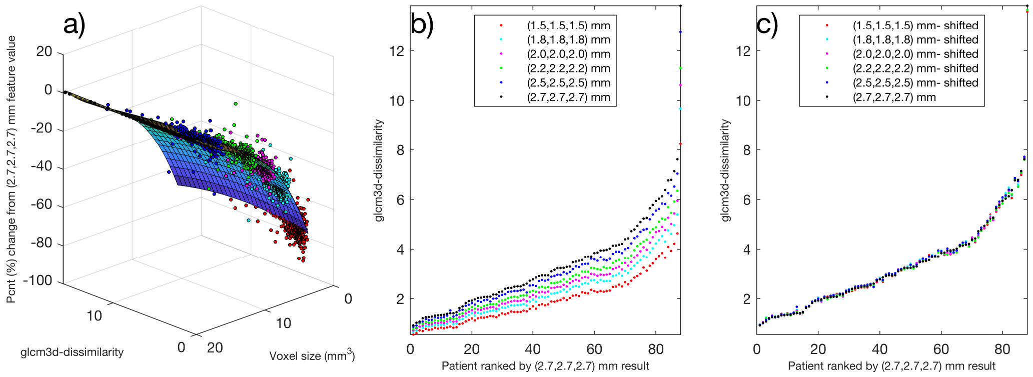

Figure 151: Modelling response: glcm3d-dissimilarity

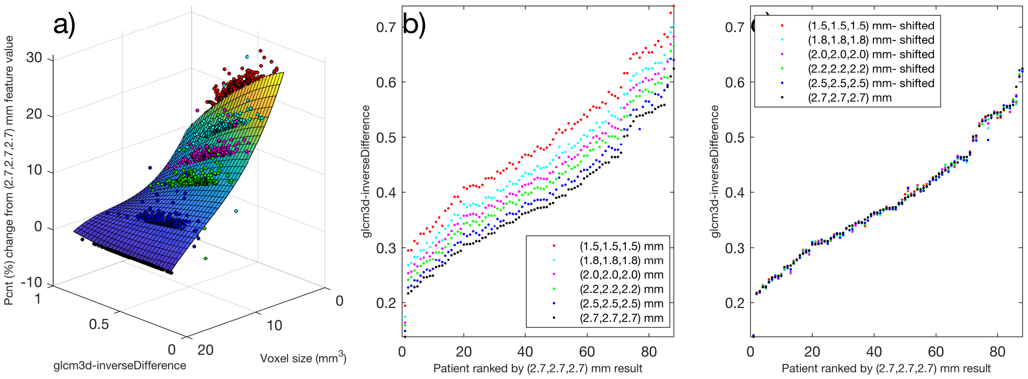

Figure 152: Modelling response: glcm3d-inverseDifference

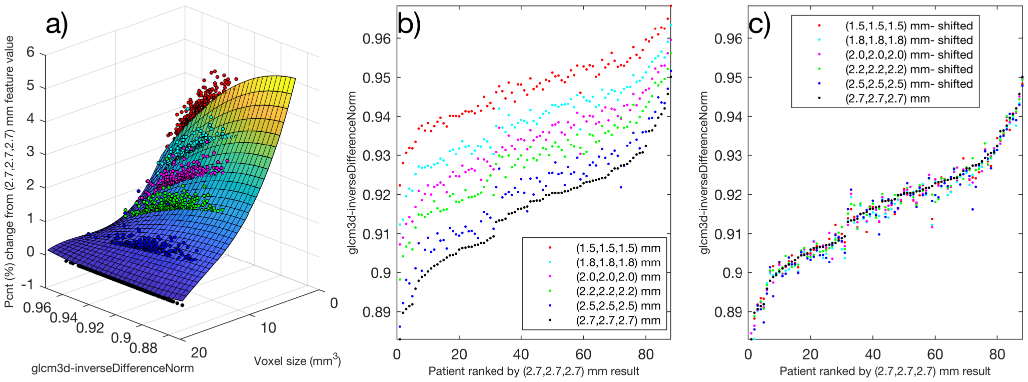

Figure 153: Modelling response: glcm3d-inverseDifferenceNorm

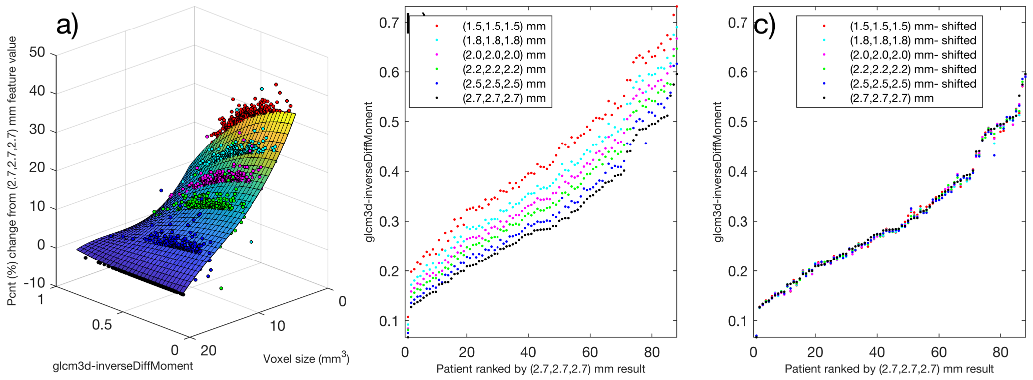

Figure 154: Modelling response: glcm3d-inverseDiffMoment

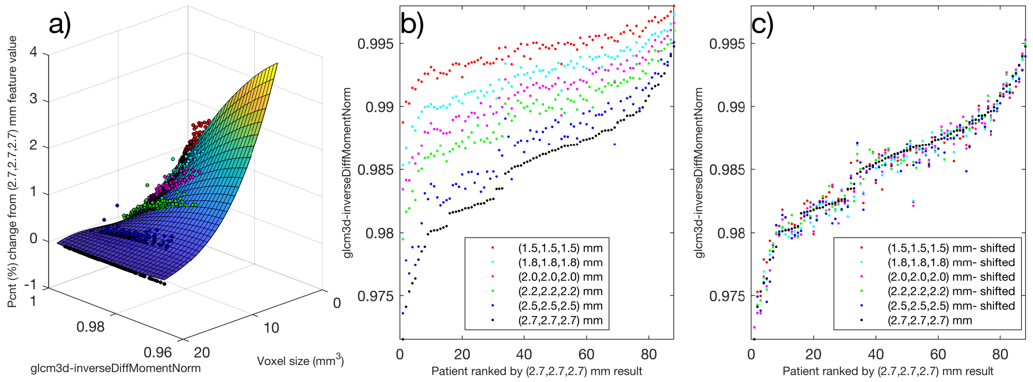

Figure 155: Modelling response: glcm3d-inverseDiffMomentNorm

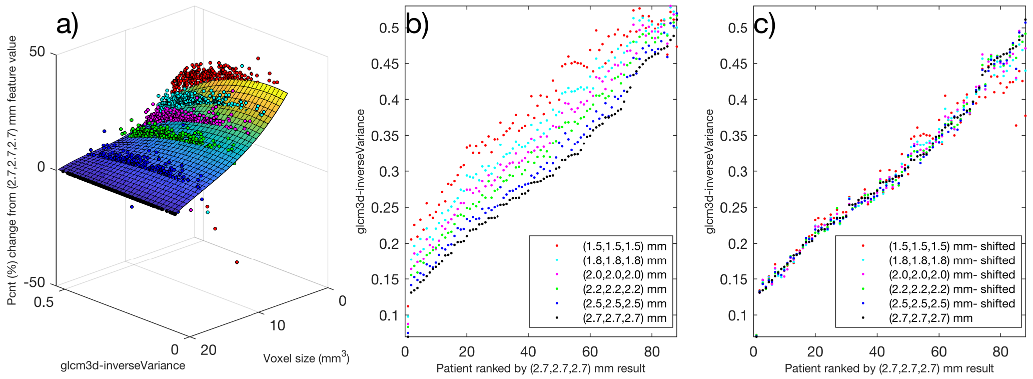

Figure 156: Modelling response: glcm3d-inverseVariance

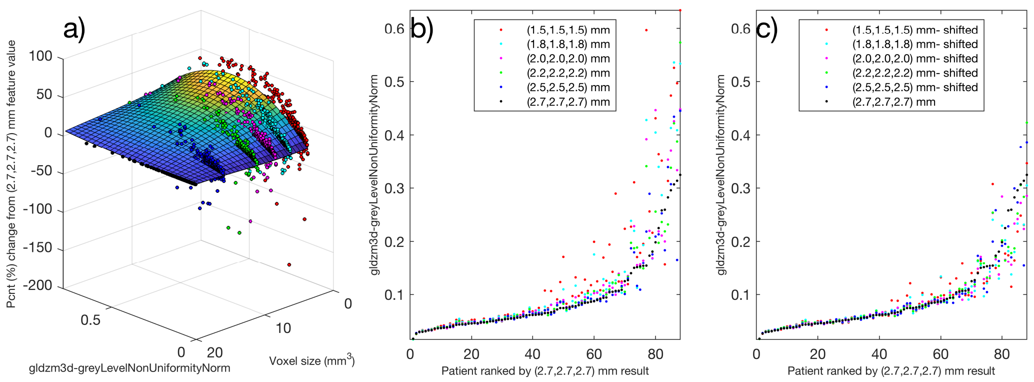

Figure 157: Modelling response: gldzm3d-greyLevelNonUniformityNorm

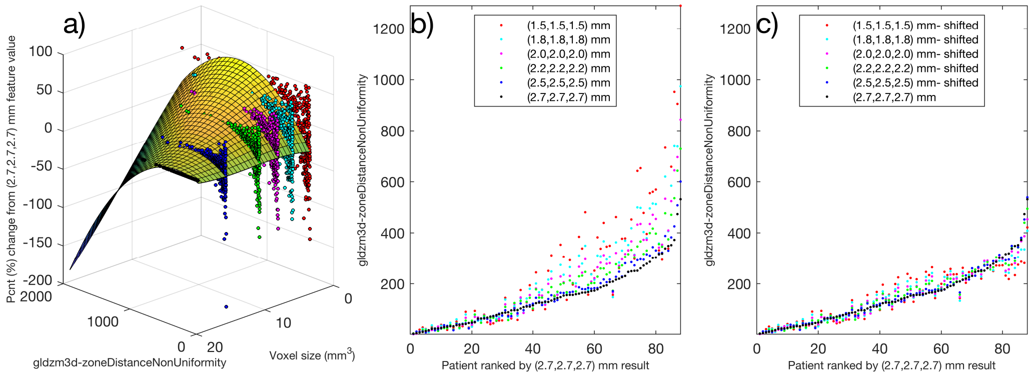

Figure 158: Modelling response: gldzm3d-zoneDistanceNonUniformity

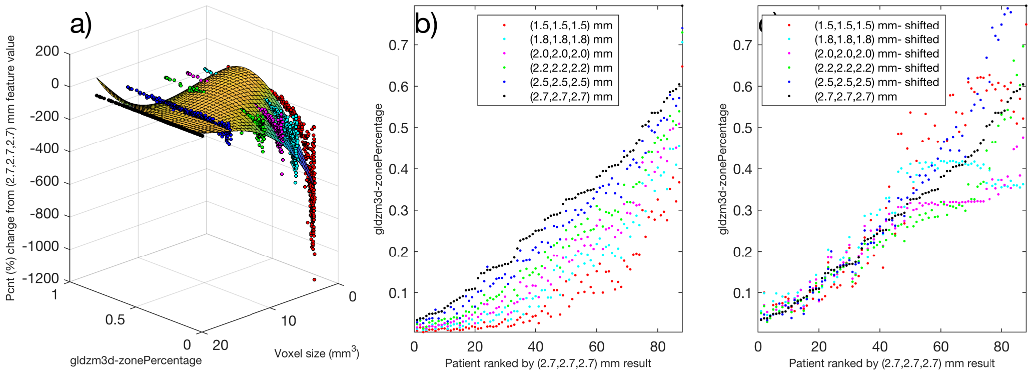

Figure 159: Modelling response: gldzm3d-zonePercentage

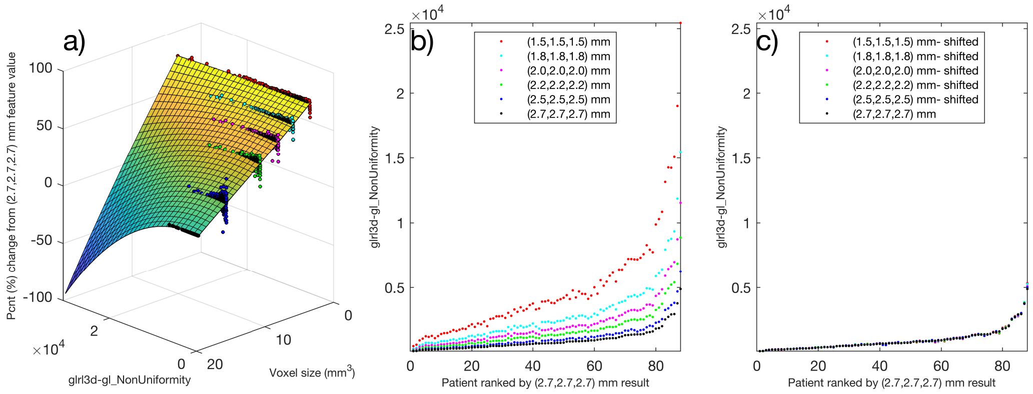

Figure 160: Modelling response: glrl3d-gl-NonUniformity

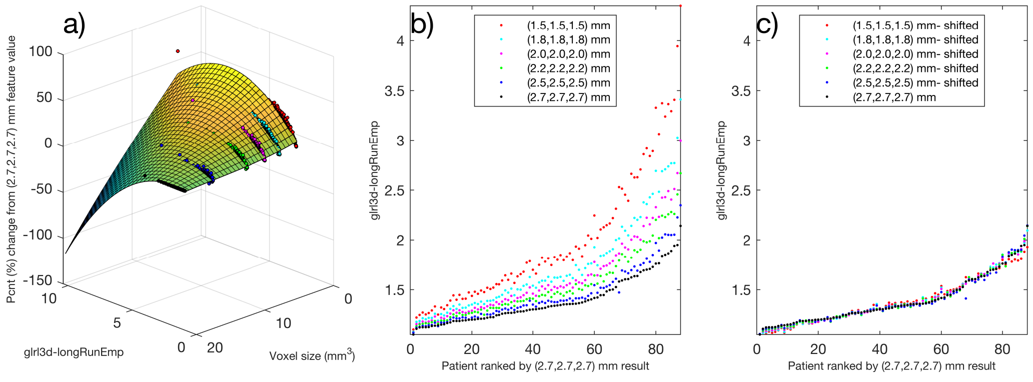

Figure 161: Modelling response: glrl3d-longRunEmp

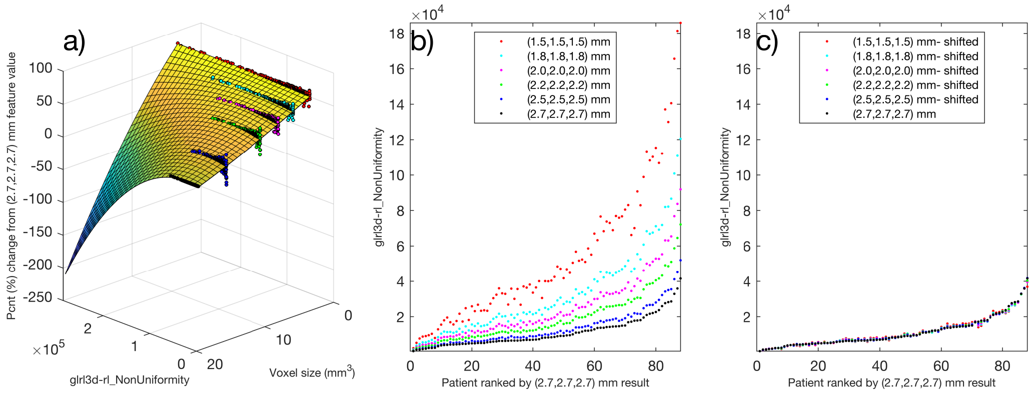

Figure 162: Modelling response: glrl3d-rl-NonUniformity

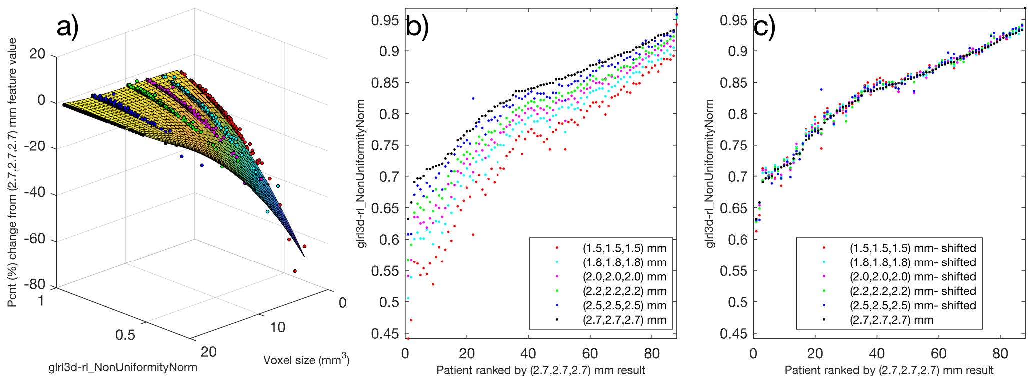

Figure 163: Modelling response: glrl3d-rl-NonUniformityNorm

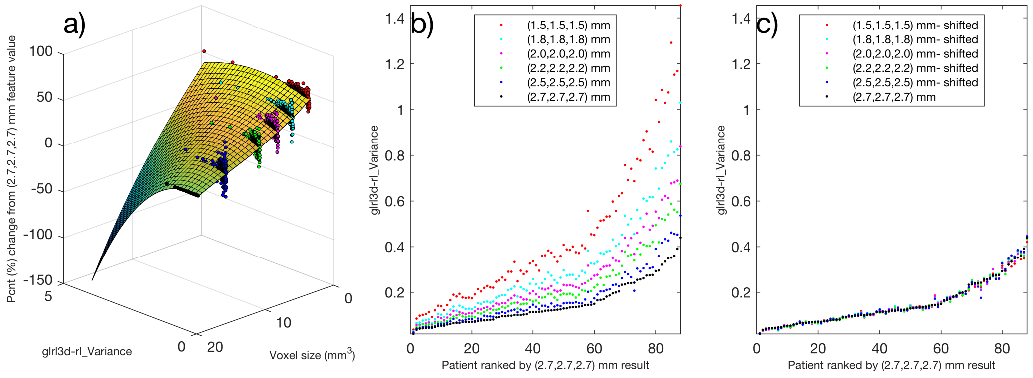

Figure 164: Modelling response: glrl3d-rl-Variance

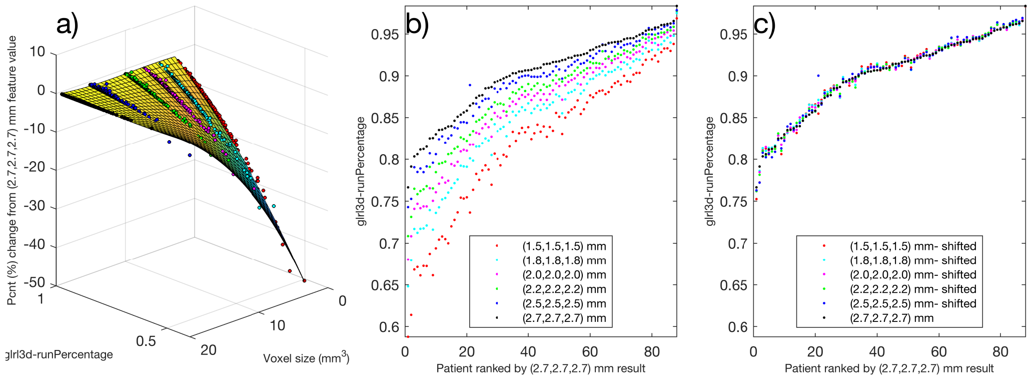

Figure 165: Modelling response: glrl3d-runPercentage

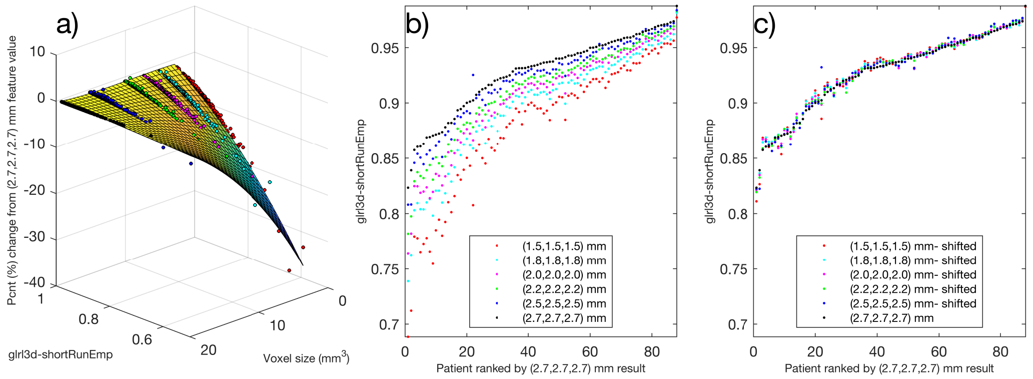

Figure 166: Modelling response: glrl3d-shortRunEmp

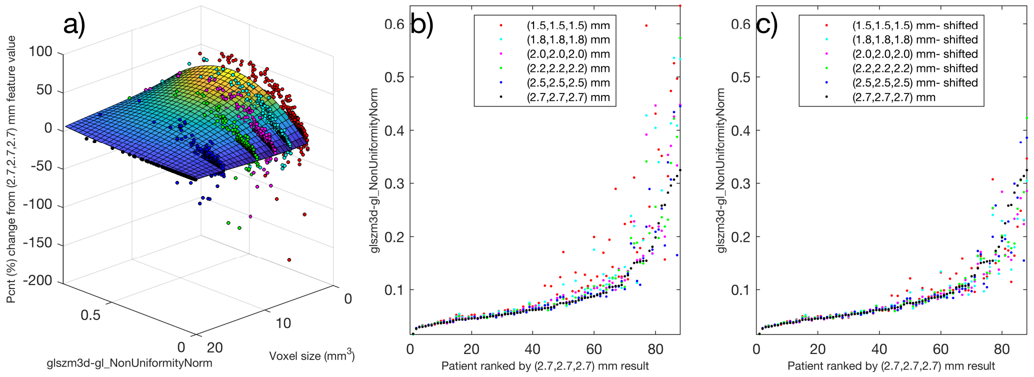

Figure 167: Modelling response: glszm3d-gl-NonUniformityNorm

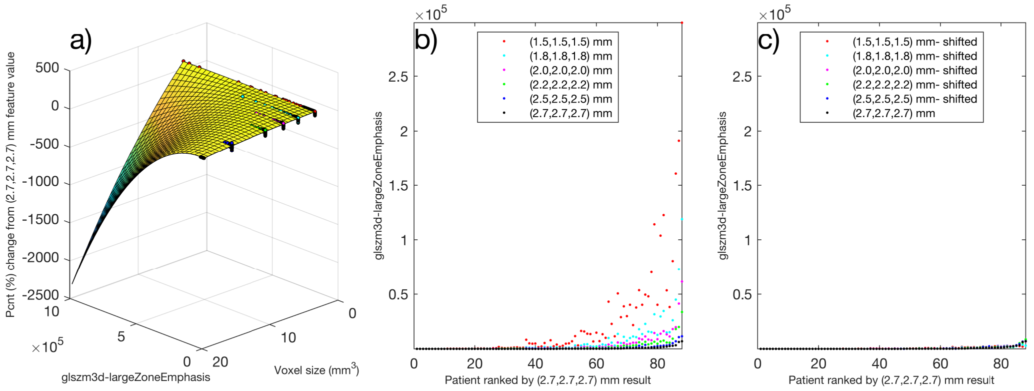

Figure 168: Modelling response: glszm3d-largeZoneEmphasis

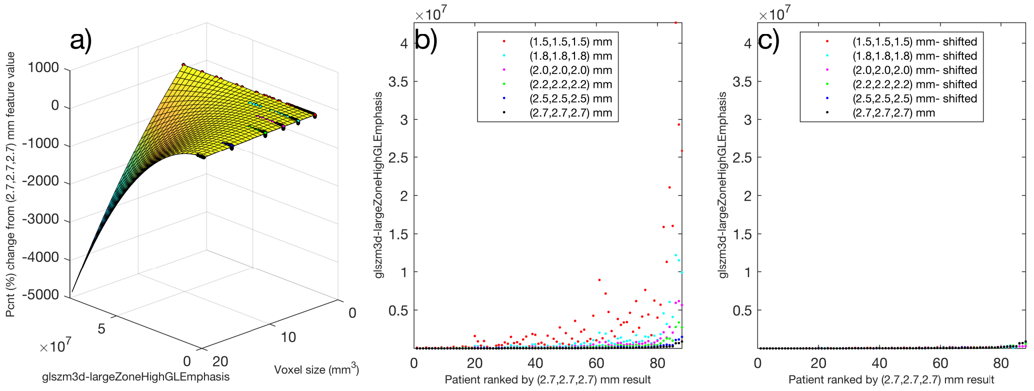

Figure 169: Modelling response: glszm3d-largeZoneHighGLEmphasis

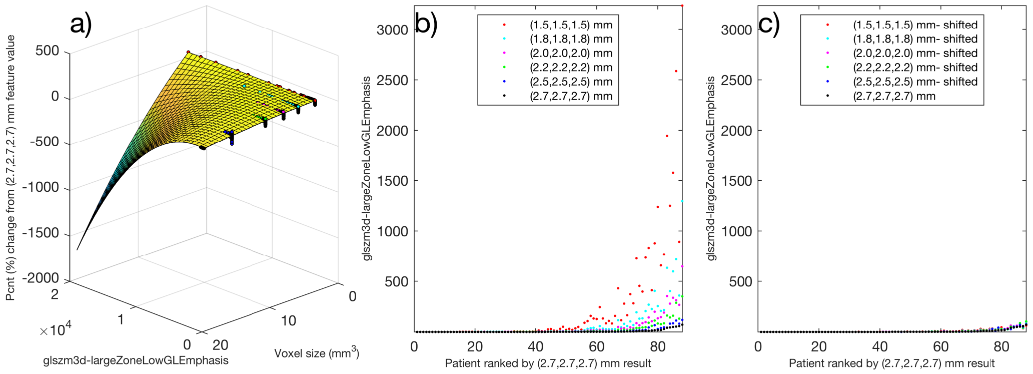

Figure 170: Modelling response: glszm3d-largeZoneLowGLEmphasis

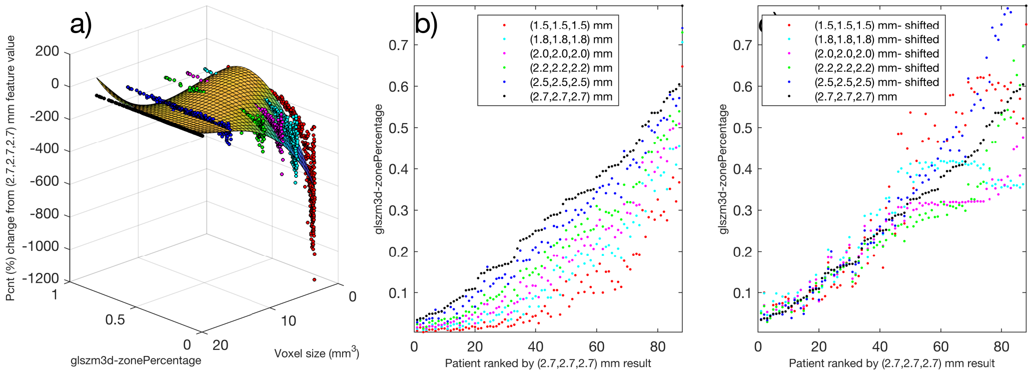

Figure 171: Modelling response: glszm3d-zonePercentage

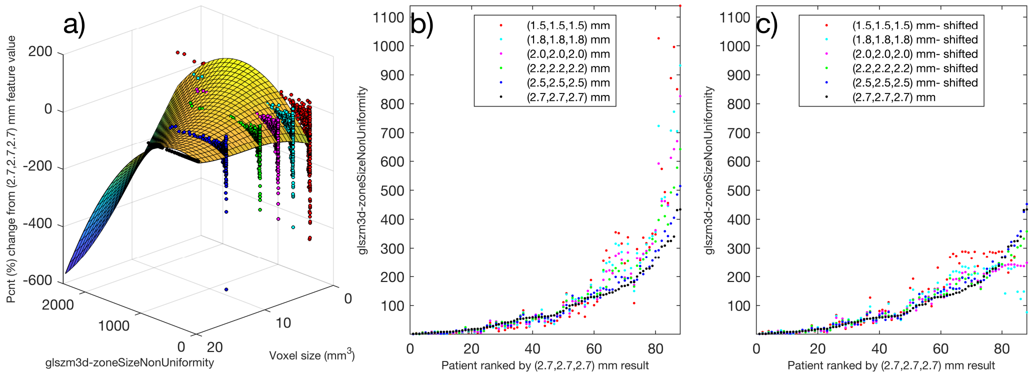

Figure 172: Modelling response: glszm3d-zoneSizeNonUniformity

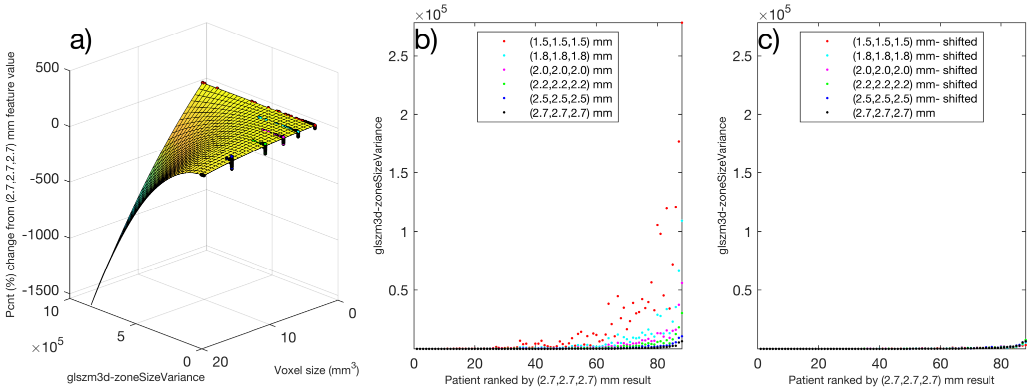

Figure 173: Modelling response: glszm3d-zoneSizeVariance

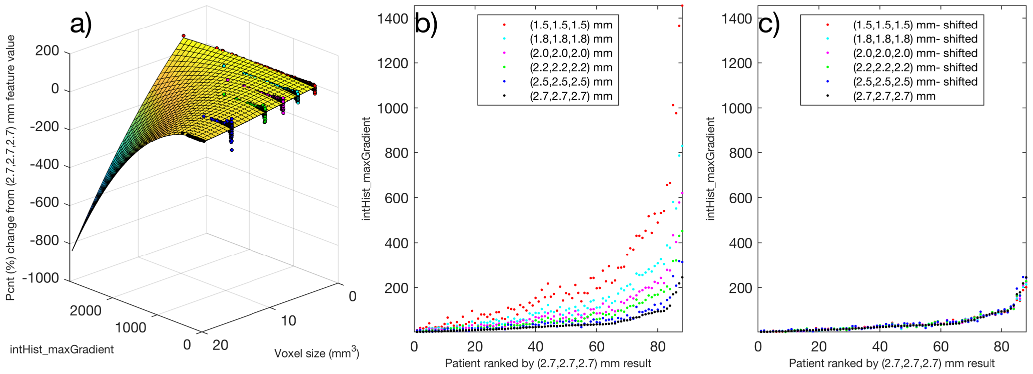

Figure 174: Modelling response: intHist-maxGradient

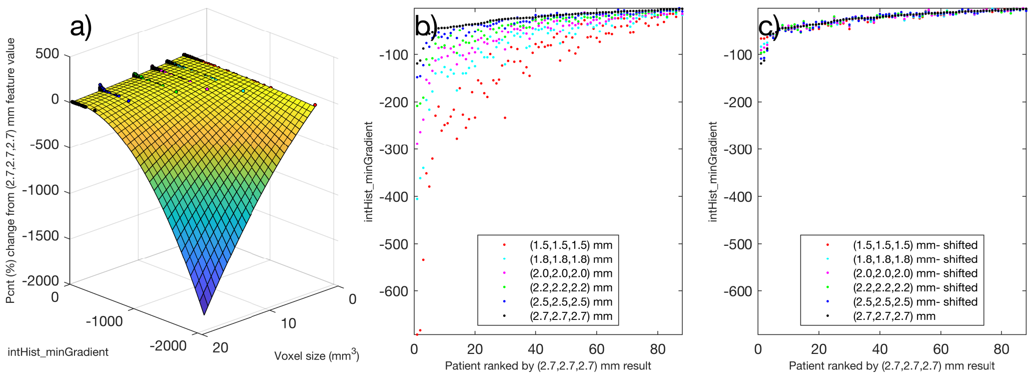

Figure 175: Modelling response: intHist-minGradient

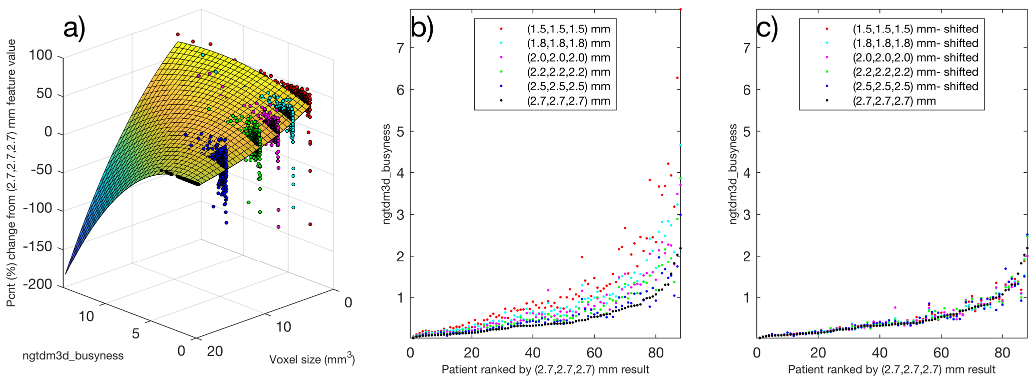

Figure 176: Modelling response: ngtdm3d-busyness

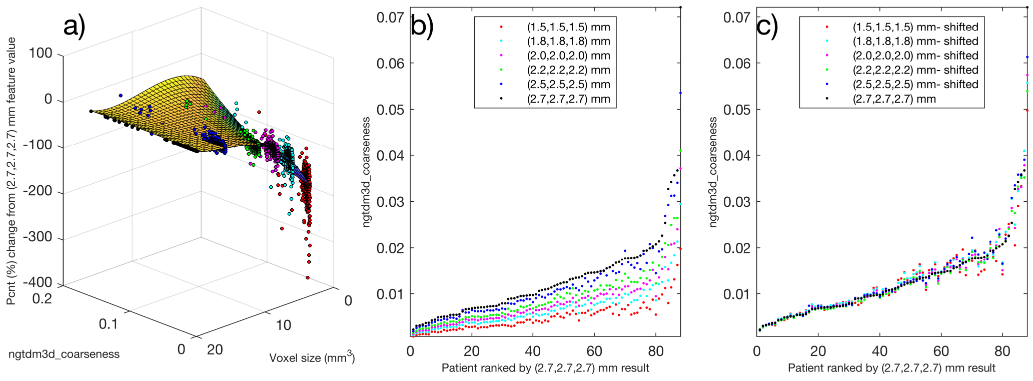

Figure 177: Modelling response: ngtdm3d-coarseness

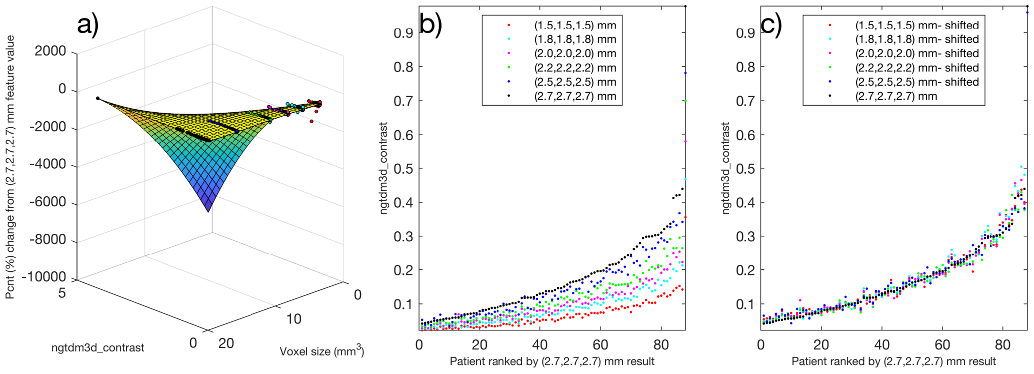

Figure 178: Modelling response: ngtdm3d-contrast

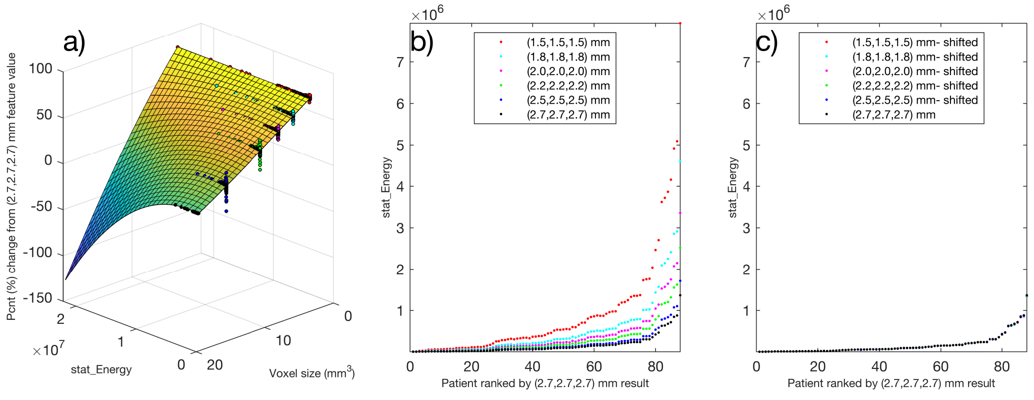

Figure 179: Modelling response: stat-Energy

References

[1] K. G. Foley, R. K. Hills, B. Berthon, C. Marshall, C. Parkinson, W. G. Lewis, T. D. Crosby, E. Spezi, and S. A. Roberts, "Development and validation of a prognostic model incorporating texture analysis derived from standardised segmentation of PET in patients with oesophageal cancer," *European Radiology*, vol. 28, pp. 428–436, jan 2018.

[2] J. O. Deasy, A. I. Blanco, and V. H. Clark, "CERR: A computational environment for radiotherapy research," *Medical Physics*, vol. 30, no. 5, pp. 979–985, 2003.

[3] B. Berthon, C. Marshall, M. Evans, and E. Spezi, "ATLAAS: an automatic decision tree-based learning algorithm for advanced image segmentation in positron emission tomography," *Physics in Medicine and Biology*, vol. 61, no. 13, pp. 4855–4869, 2016.

[4] A. Zwanenburg, S. Leger, M. Vallières, and S. Löck, "Image biomarker standardisation initiative," *arXiv:1612.07003.*, dec 2016.
